# Physiological and transcriptomic responses of Lanzhou Lily (*Lilium davidii*, var. unicolor) to cold stress

**DOI:** 10.1371/journal.pone.0227921

**Published:** 2020-01-23

**Authors:** Xuehui Tian, Jianming Xie, Jihua Yu

**Affiliations:** 1 College of Horticulture, Gansu Agricultural University, Lanzhou, Gansu, China; 2 Department of Ecological Environment and Engineering, Yangling Vocational and Technical College, Yangling, Shanxi, China; Institute for Horticultural Plants, China Agricultural University, CHINA

## Abstract

Low temperature induces changes in plants at physiological and molecular levels, thus affecting growth and development. The Lanzhou lily (*Lilium davidii*, var. unicolor) is an important medicinal plant with high economic value. However, the molecular mechanisms underlying its photosynthetic and antioxidation responses to low temperature still remain poorly understood. This study subjected the Lanzhou lily to the two temperatures of 20°C (control) and 4°C (low temperature) for 24 h. Physiological parameters related to membrane integrity, photosynthesis, antioxidant system, and differentially expressed genes were investigated. Compared with control, low temperature increased the relative electrical conductivity by 43.2%, while it decreased net photosynthesis rate, ratio of variable to maximal fluorescence, and catalase activity by 47.3%, 10.1%, and 11.1%, respectively. In addition, low temperature significantly increased the content of soluble protein, soluble sugar, and proline, as well as the activity of superoxide dismutase and peroxidase. Comparative transcriptome profiling showed that a total of 238,109 differentially expressed genes were detected. Among these, 3,566 were significantly upregulated while 2,982 were significantly downregulated in response to low temperature. Gene Ontology enrichment analysis indicated that in response to low temperature, the mostly significantly enriched differentially expressed genes were mainly involved in phosphorylation, membrane and protein kinase activity, as well as photosynthesis, light harvesting, light reaction, and alpha,alpha-trehalose-phosphate synthase activity. Kyoto Encyclopedia of Genes and Genomes enrichment analysis also indicated that the most significantly enriched pathways involved ribosome biogenesis in eukaryotes, phenylalanine metabolism, circadian rhythm, porphyrin and chlorophyll metabolism, photosynthesis of antenna proteins, photosynthesis, and carbon fixation in photosynthetic organisms. Moreover, the expression patterns of 10 randomly selected differentially expressed genes confirmed the RNA-Seq results. These results expand the understanding of the physiological and molecular mechanisms underlying the response of the Lanzhou lily to low temperature stress.

## Introduction

Under natural conditions, plants often suffer from a variety of biotic and abiotic stresses, such as drought [1–4], salt stress [5], high temperature [6], and chilling injury [7–8]. Temperature is the main determinant that affects the geographical distribution and the length of the growing season for most plants [9–11]. Low temperature is one of the most common abiotic stresses and severely affects plant growth, development, production, and quality [12–15], especially during late autumn, winter, and early spring in northwest China. Low temperature induces a series of morphological, physiological, biochemical, and molecular changes [16]. How the combination of these factors affects plant growth and production has attracted increasing research attention.

Exposure to LT results in several acclimation responses [12], e.g., shortening of the vegetation period [17], osmotic regulation [18], and antioxidant system response [19], which are natural defense mechanisms against possible injury [20]. Relative electrical conductivity (Rec) and activity of antioxidant enzymes are typically measured as physiological indicators of cold resistance [21]. Photosynthesis is widely considered to be significantly affected by temperature [22]. Chlorophyll fluorescence analysis is one of the most powerful and popular techniques for the investigation of the influence of various stresses on the leaf gas exchange [23]. Several studies reported that LT significantly reduced net photosynthesis rate (Pn) and the ratio of variable to maximal fluorescence (Fv/Fm) [24–27] in plants; it also increased the activity of antioxidant enzymes such as superoxide dismutase (SOD, EC 1.15.1.1) and catalase (CAT, EC 1.11.1.6) [28] to resist possible injury. However, if the production rate of reactive oxygen species exceeds the scavenging ability of antioxidants, reactive oxygen species would excessively accumulate and damage plant tissues [29–30]. Moreover, cold-specific responses have been observed in more comprehensive pathways, including the regulation of gene expression [31], redox state [32], and complex signaling [33–34]. Investigating the physiological and molecular mechanisms underlying the cold tolerance may provide information for both the environmental regulation and the genetic improvement of plant growth and production.

Lilies have curative effects on diseases such as chronic bronchitis, chronic gastritis, pertussis, and tuberculosis, and are therefore regarded as an important traditional medicine in China for many years [35]. More than 100 lily species have been described worldwide, 55 of which originated in China [36]. The responses of individual species to cold stress may differ, since they are native to different climatic environments. The Lanzhou lily (*Lilium davidii*, var. unicolor) is a medicinal and ornamental plant with high economic value [37]; it is a mutation of *L*. *davidii* Duchartre and one of the best edible lilies in China [38]. It grows in the Qilihe District of Lanzhou City, Gansu province, China, at an altitude exceeding 2,000 m [31,39]. This area often suffers from low temperature during winter and early spring, when the highest average daytime temperature does not exceed 4°C. However, the osmotic regulation and oxidative damage of lily leaves to cold stress has not received significant research attention [40]. Although temperature is an important environmental factor that affects lily bulb dormancy, most research regarding chilling effects in lilies focused on the cold period requirement of dormancy postharvest physiology (vernalization) of lily bulbs, while more or less ignoring the vegetative growth stage [8,41–44]. A previous study showed that temperatures below 10°C inhibited the growth of lily bulbs [45]. Since the bulb is the production organ of the lily plant, current studies on the cold resistance of lilies mainly focus on its quality [46] and vernalization process [42–43]. Most lily species developed dormancy to survive the cold season (e.g., late fall, winter, and early spring), thus leaving the bulb live in the soil [46–47]. Vegetative growth is central to accumulate saccharides for the growth of bulbs, thus it is important to study the impact of cold stress on physiological parameters to provide insight into the physiological mechanisms underlying growth during cold stress.

Transcriptome sequencing is a powerful and popular modern genetic research tool. The main advantages are its low cost, high accuracy, high efficiency, and sensitive profiling characteristics [37]. This tool is increasingly applied to analyze the mRNA expressions of genes in response to a specific biological process, which enables the identification of their inner molecular mechanisms [48–50]. By using RNA sequencing technology, stress-induced key genes can be identified and analyzed. These can then be manipulated by genetic methods to improve stress tolerance in crops [49], and provide new insights into physiological changes at the transcriptional level [51]. To date, considerable research focused on the transcriptional responses to cold stress in a number of lily species, including *L*. *lancifolium* [4,44,51], *L*. *longiflorum* [41], *L*. *sorbonne* [42,46], and *L*. *pumilum* [43]. These studies identified a large number of genes related to cold stress that are involved in a variety of biological processes [4], indicating that lilies have the ability to induce a series of transcriptome changes during the vernalization process [42–43,46]. However, no transcriptomic characterization of the photosynthesis and cold stress induced gene expression is available for the Lanzhou lily to, especially during the early vegetative stage.

This study used the Lanzhou lily as material and normal temperature as control (CK, 20°C) to investigate the physiological (including photosynthesis, osmoregulation, antioxidant, and fluorescence parameters) and transcriptome responses to low temperature (LT, 4°C). The objectives of this study were to: (1) investigate the physiological response to LT; (2) identify key genes that respond to LT; and (3) explain the resulting physiological changes at the transcriptome level. We hypothesized that LT would mainly affect the expression of genes involved in photosynthesis, carbon assimilation, and antioxidant pathways at the transcriptional level, thus influencing plant growth and development. Studying the physiological and transcriptomic responses of the Lanzhou lily to LT will further increase the understanding of the underlying mechanism of its adaption to cold stress.

## Materials and methods

### Materials

Forty three-year-old Lanzhou lily bulbs (*Lilium davidii* var. unicolor) were purchased from a local flower market in Qilihe District (103°54'E, 35°56'N, altitude 2,638 m), Lanzhou city, Gansu province, China, and were stored for 110 days in a refrigerator at 2°C. After vernalization, the bulbs were planted in 15 cm diameter flower pots filled with 2 L of commercial cultivation substrate (Mengda, Yufeng Co., Ltd., Xianyang, China), and were placed in an intelligent artificial climate box (RXZ-0288, Ningbo Jiangnan Instrument Factory, Ningbo, China) at the Northwest Agriculture and Forestry University, Yangling, Shaanxi, China.

### Experimental design

The growth environment was set to a temperature of 20/15°C (day/night), a relative air humidity of 50–65%, a photoperiod of 16/8 h (day/night), and an average light intensity of 350 ± 20 μmo·m^-2^·s^-1^. Pots were watered every 3–5 days with 300 mL half-strength Hoagland nutrient solution to keep plants well fertilized. The seedlings were grown for 40 d. After that, uniformly sized healthy lily seedlings with a height of about 15 cm were transplanted into plastic pots (upper diameter of 20 cm and height of 16 cm, one plant per pot) in two intelligent artificial climate chambers (RXZ-0288; Ningbo, China) for the different treatments (12 seedlings per chamber). One chamber was set to 20°C and was used as CK, while the other was set to 4°C and was used as LT treatment. All other environmental parameters were the same as those for the pre-treatment described above. The treatments lasted for 24 h. Then, the leaves from the same-leaf position (the 7^th^ to 10^th^ leaves counted from the top) were sampled and immediately frozen in liquid nitrogen and stored at -80°C until further processing except for Pn and Fv/Fm.

### Data analysis

#### Rec, Pn, Fv/Fm, osmoregulation substance, and antioxidant enzyme activities

Rec in lily leaves was measured according to the method of Gomes et al. [1] with minor modification. Ten pieces of leaves were completely immersed in 15 mL of deionized water for 60 min. After that, the electrical conductivity (EC_60_) was measured for five replicates with a conductivity meter (DDSJ-308A; Shanghai, China). Then, the leaves were transferred to 15 mL of boiling water for 30 min, and the EC_30_ were assessed after the water was cooled to room temperature. Then, Rec was calculated as the ratio of EC_60_ to EC_30_ (%).

During daytime, photosynthesis was measured using a portable photosynthesis analyzer (Li-6400, Li-Cor Inc., Lincoln NE, USA) equipped with a narrow-leaf chamber. For both treatments, the environment of the leaf chamber was set to light intensity of 350 ± 10 μmol·m^-2^·s^-1^, a CO_2_ concentration of 400 μmol·mol^-1^, and a relative humidity range of 50–70%. The temperatures differed and 4°C was used for LT treatment and 20°C was used for CK. Furthermore, the Fv/Fm of leaves was determined by the Open FluorCam FC 800-O and analyzed using Fluorcam7 software (PSI, Brno, Czech Republic) [52].

The content of soluble protein (SP) was measured following the method of Bates et al. [53] with minor modification. About 0.3 g of frozen leaflet tissue was extracted in 80% ethanol and measured with a UV-spectrophotometer (UV-1800, Shimadzu, Kyoto, Japan) at A_520_ using the nyhnidrin method with four replicates. In addition, the soluble sugar (SS) content was determined according to the method of Pan et al. [54] with minor modifications. About 2 g of fresh leaves was homogenized with 5 mL of 80% ethyl alcohol and measured with a UV-spectrophotometer at A_620_ using the anthrone sulfuric acid method with four replicates. Furthermore, the content of proline (Pro) was measured according to the method of Bates et al. [53] via the sulfosalicylic acid-ninhydrin reaction. The absorbance was determined at 532 nm by a UV-spectrophotometer and the Pro content was calculated by a standard curve of absorbance vs a series of standard Pro contents gradients.

SOD activities were determined using the nitro blue tetrazolium (NBT) method as described by Giannopolitis et al. [55]. Moreover, the activity of peroxidase (POD, EC 1.11.1.7) was measured according to the method of Yang et al. [56] with minor modifications. A leaf sample of about 0.5 g was ground on ice and filled to a constant volume of 50 mL with distilled water. Then, the absorbance of the reaction mixture was automatically recorded by a UV-spectrophotometer at A_470_ for 3 min, and a change of 0.01 in absorbance per minute presented one unit of enzyme activity. In addition, the CAT activity was determined by recording the changes of H_2_O_2_ at A_240_ within 2 min [57].

#### RNA extraction, library construction, and quality control

Transcriptome sequencing and analysis were completed by Allwegene Technology Co., Ltd., Beijing, China. Each treatment had three biological replicates. Total lily RNA in leaves was extracted using the TRIzol method [58]. RNA concentration, purity, and integrity were tested via spectrophotometry and agarose gel electrophoresis (BIO-RAD, USA) to ensure the accuracy of the data [49].

After that, the mRNA was enriched by magnetic beads with oligo-dT and a fragmentation buffer was added to break mRNAs into short fragments. Then, these mRNA fragments were used as templates to synthesize one strand of cDNA, and then two strands of cDNA. After that, AMPure XP beads were used to purify the synthetic two-stranded cDNA, followed successively by end repair, addition of polyA tails, connection of sequencing joints, and selection of fragment size. Finally, PCR amplification was performed, and the PCR products were purified to obtain the final library [15].

Then, Qubit 2.0 was used for preliminary quantification, and Agilent 2100 was used for the detection of the inserted fragment size of the library. PCR was performed to accurately quantify the effective concentration of the library, and the Illumina 4000 high-throughput sequencing platform (HiSeq^TM^2500, Allwegene Technology Co., Ltd., Beijing, China) was used for sequencing after quality inspection. The data of the Illumina 4000 high-throughput sequencing platform were defined as raw reads or raw data. To improve the quality of sequences, Trimmomatic software (v0.33) was used to remove reads that contained adapters, as well as reads that contained N (where N stands for unascertained base information) at a rate exceeding 10%. Low-quality reads (where the base number of the mass value Q ≤ 20 accounted for more than 50% of the whole read) were also removed.

#### Assembly and functional annotation

After the adapter sequences and low-quality sequences of raw reads were deleted, clean reads were assembled without reference genome [59]. Then, these sequences were assessed by seven commonly used databases, including NCBI protein non-redundant database (NR), NCBI nucleic acid sequence database (NT) (http://www.ncbi.nlm.nih.gov/), Kegg Ortholog (KO), Swiss-Prot, Pfam (database) protein family, eukaryotes orthologous genes database (KOG), and Gene Ontology (GO) (http://www.geneontology.org/). The results were obtained according to methods of Nie et al. [15]. Then, the annotated unigenes involved in GO, Kyoto Encyclopedia of Genes and Genomes (KEGG, http://www.genome.jp/kegg/), and KOG were classified to evaluate the function of assembled unigenes. The clean data were deposited in the Short Read Archive (SRA) database of NCBI under the accession number PRJNA565864.

#### Identification of DEGs, as well as GO and KEGG enrichment analyses

The expression level of each gene was normalized by Fragments Per Kilobase of transcript per Million fragments mapped (FPKM) values. DESeq (v1.10.1) was used to screen DEGs, with a threshold criteria of *Qvalues* < 0.005 and |log_2_(fold change)| > 1. Compared results were statistically analyzed by RNA-Seq by Expectation-Maximization (RSEM) (http://deweylab.github.io/RSEM/). DEGs were further assessed using GO and KEGG annotation and significant enrichment analysis. Goseq (v1.22.0) software was used to identify the significantly enriched pathways in DEGs for the GO pathway enrichment analysis, using the corrected *P-value < 0*.*05* as threshold value. In addition, KOBAS (v2.0) software was used for KEGG enrichment analysis with corrected *P-value < 0*.*05* as threshold value, to identify significantly enriched pathways.

#### Real-time quantitative PCR verification

Ten key DEGs, associated with photosynthesis, and carbon fixation in the photosynthetic process, were randomly selected for qRT-PCR analysis to verify high-throughput data. Using the same samples tested in transcriptome profiling, total RNA of CK and LT were extracted by Plant RNA Kit (OmegaBio-Tek, Doraville, GA, USA) and were then reverse-transcribed by a PrimeScript TM RT reagent kit with a gDNA Eraser (Takara, Shiga, Japan), following the manufacturer’s protocol. The actin gene was used as internal reference. The primers used for key DEGs and actin and their function are presented in S1 Table. The PCR reactions were performed on a StepOnePlus Real-Time PCR System (Applied Biosystems, Carlsbad, CA, USA) using SYBR Premix Ex Taq (Takara). Each treatment was repeated with three biological replicates and each sample was assayed in triplicate. The data were calculated with the 2^-ΔΔCT^ method [60].

#### Statistical analysis

All physiological data were analyzed by one-way analysis of variance (ANOVA) using SPSS 20.0 (IBM Corp., Armonk, NY, USA) to detect significant differences between both treatments (*P* < 0.05) and results were plotted with Graphpad Prism 6 (GraphPad software Inc., La Jolla, CA, USA). Vertical bars in figures represent the means ± SD (n = 3–5). The transcriptome data were analyzed by Allwegene Technology Co., Ltd., Beijing, China.

## Results

### Rec, Pn, Fv/Fm, osmoregulation substances, and antioxidant enzyme system

**Fig 1:**
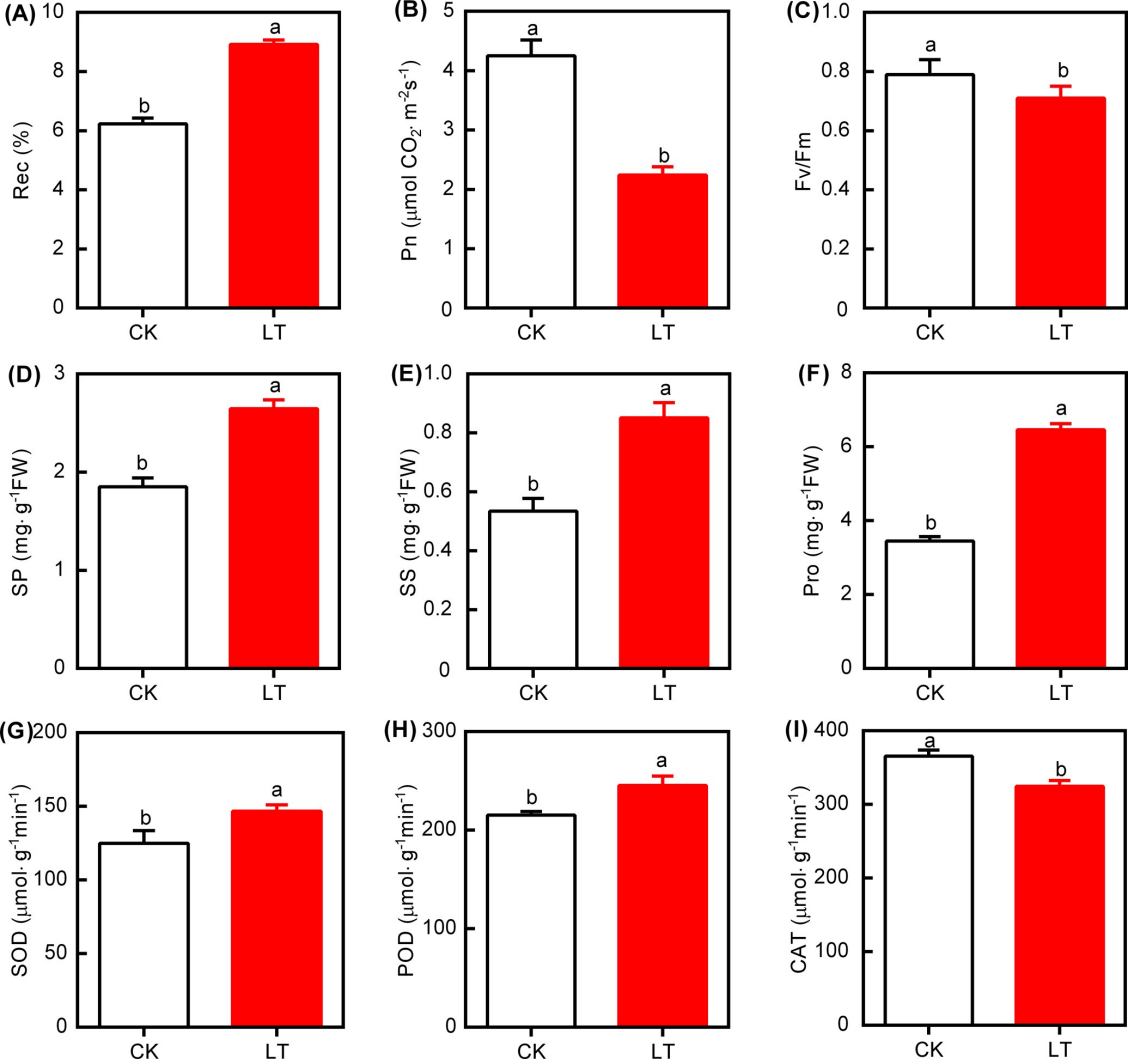
Responses of relative electrical conductivity (Rec, A), net photosynthesis (Pn, B), maximal fluorescence (Fv/Fm, C), soluble protein (SP, D), soluble sugar (SS, E), and proline (Pro, F), superoxide dismutase (SOD, G), peroxidases (POD, H), and catalase (CAT, I) in leaves of the Lanzhou lily to different temperatures. White bars and red bars represent treatments of CK and LT, respectively. Error bars indicate ± SD (n = 3–5). Different letters indicate significant differences between both treatments (*P* < 0.05). CK: control (20°C); LT: low temperature (4°C).

LT significantly affected indicators related to photosynthesis, osmotic regulation, and antioxidant system in leaves of the Lanzhou lily (Fig 1). Compared with CK, LT significantly increased Rec by 43.2% (Fig 1A). However, it decreased both Pn and Fv/Fv by 47.3% and 10.1%, respectively (Fig 1B and 1C). In addition, LT significantly increased SP (Fig 1D), SS (Fig 1E), and Pro (Fig 1F) contents by 42.9%, 59.4%, and 87.3%, respectively. Moreover, it significantly increased both SOD activity (17.5%, Fig 1G) and POD activity (13.9%, Fig 1H), but decreased CAT activity by 11.1% (Fig 1I).

### Sequencing results and trinity splicing

To understand the molecular mechanism underlying the physiological response of the Lanzhou lily to LT stress, transcriptome sequencing was conducted using three biological replicates. A total of 38.57 million and 38.66 million raw reads were obtained from the transcriptome libraries of lily leaves under CK and LT treatments, respectively (S2 Table). The clean reads ratio of CK to LT exceeded 94.52% and 95.89%, respectively. Additionally, Q20 of CK and LT exceeded 98.81% and 98.86%, respectively. Moreover, all GC contents of CK and LT exceeded 49.95%. The results of trinity transcript splicing and cd-hit de-redundant sequences are shown in S3 Table, yielding a total number of 168,637,667 sequences, with a median length of 341 bp, a shortest length of 201 bp, a longest length of 21,703 bp, and an N50 length of 829 bp in transcript sequence. With regard to the unigene sequence, a total of 133,179,110 bp (about 79% that of transcript) were yielded, with shortest, median, longest, and N50 lengths of 201, 327, 21,073, and 757 bp, respectively. The unigene value exceeded 90% of that of the transcript sequence (except for the total number of nucleotides). Overall, these results indicated that transcriptome sequencing of the Lanzhou lily was of high quality and high purity and thus, suitable for downstream analyses.

### Gene function annotation

The annotation statistics of all unigenes were aligned to seven protein databases including NR, NT, KO, Swiss-Prot, Pfam, GO, and COG/KOG, and are shown in S4 Table. Overall, a total of 238,301 unigenes were functionally annotated. Among these, about 27.41% (65,314 unigenes) were annotated by using NR (NCBI protein non-redundant database), 17.68% (42,138 unigenes) by COG/KOG (eukaryotes orthologous genes database), 14.79% (35,244 unigenes) by GO annotation, 15.91% (37,916 unigenes) by Nt (nucleic acid sequence database), 19.44% (46,337 unigenes) by Swiss-Prot annotation, and 24.59% (58,605 unigenes) by the Pfam (database) protein family. Moreover, several other genes could not be functionally annotated, which may be due to unigene fragments that were too short and/or a lack of gene annotation information in the database.

**Fig 2:**
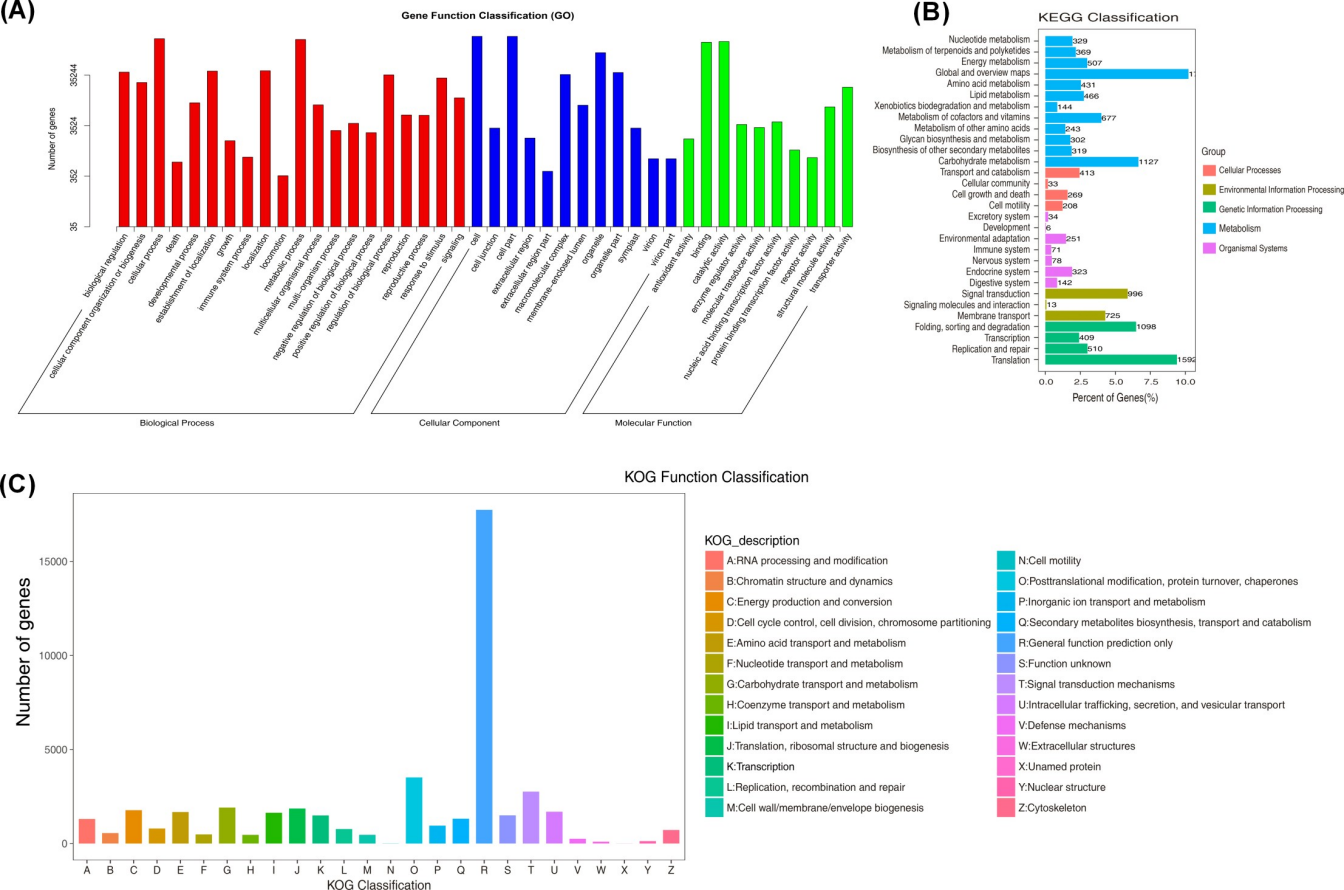
**Gene Ontology (GO) categorization (A), Kyoto Encyclopedia of Genes and Genomes (KEGG) categorization (B), and Eukaryotic Ortholog Groups (KOG) functional classification (C) of the unigenes of leaves of the Lanzhou lily exposed to different temperatures.** Each annotated sequence is assigned at least to one GO term. CK: control (20°C); LT: low temperature (4°C).

The annotated unigenes involved in GO, KEGG, and KOG were classified to evaluate the function of assembled unigenes (Fig 2). For GO classification, genes were annotated to the following three groups: biological process, cellular component, and molecular process. In the molecular function category, the clusters of “binding” and “catalytic activity” occupied the largest groups. In addition, the clusters for “cellular process” and “metabolic process” were the largest in the biological process category (Fig 2A). For KEGG classification, genes were annotated to five groups. Among the metabolism group, the clusters “global and overview maps”, “carbohydrate metabolism”, and “metabolism of cofactors and vitamins” were significantly enriched (Fig 2B). Moreover, for KOG functional classification, all unigenes were classified into 26 categories. Among these, the cluster “general function prediction only” was the largest, followed by “posttranslational modification, protein turnover, chaperones” and “signal transduction mechanisms” (Fig 2C). This implied that LT induced a series of changes in physiological and metabolic processes in leaves, either in response to or as an adaptation to LT stress.

### DEGs

**Fig 3:**
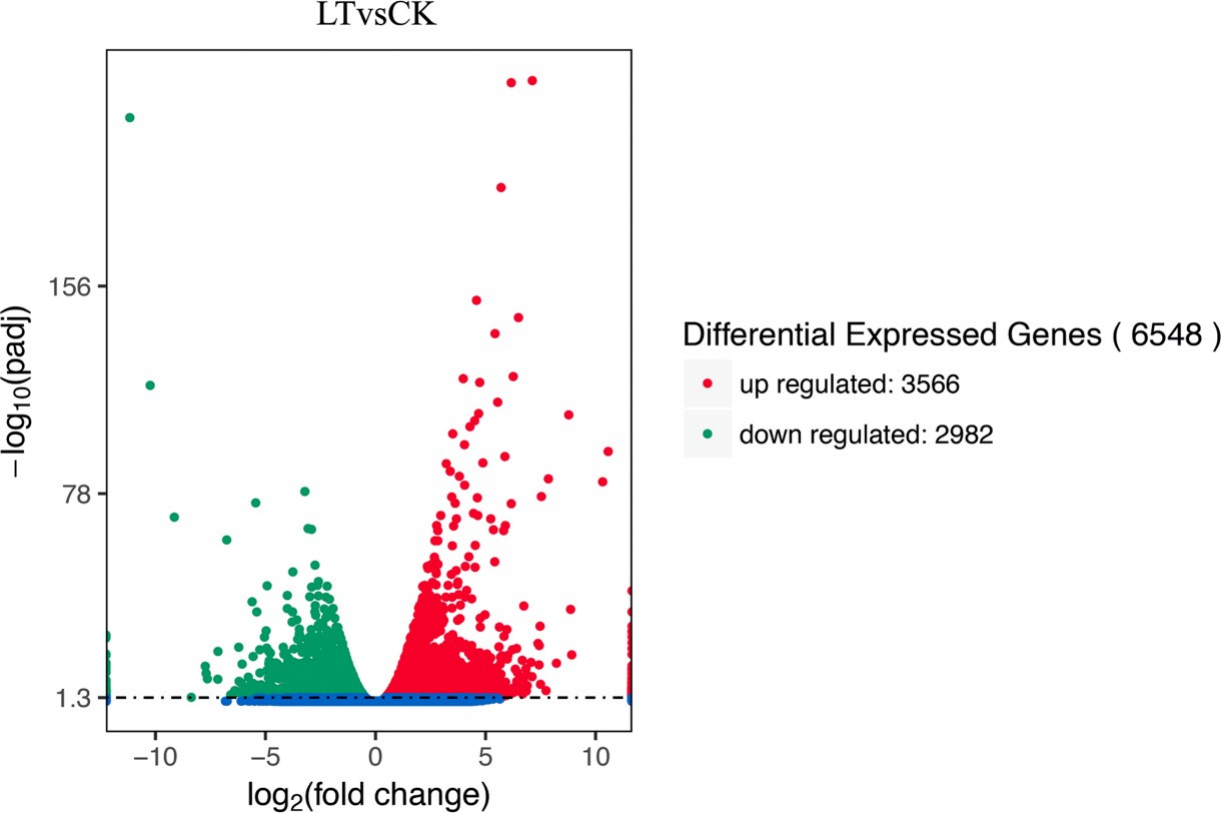
Analysis of differences in gene expression in leaves of the Lanzhou Lily exposed to different temperatures. Red points represent up-regulated genes, green points represent down-regulated genes, and blue points represent no differences. CK: control (20°C); LT: low temperature (4°C).

Fig 3 shows that LT induced a total of 6,548 DEGs, 3,566 of which were significantly up-regulated, while 2,982 were significantly down-regulated, compared with CK (Fig 3, S5 Table and S6 Table). This indicated the overall transcription difference between LT and CK during the early vegetative stage. Moreover, all DEGs were assigned to various GO terms as well as different up- (S1 Zip) or down-regulated (S2 Zip) KEGG pathways for their downstream function analysis. According to the principle of “*P*-Value < 0.05 and corrected *P*-Value <0.05” in DEGs, 26 DEGs related to ribosome biogenesis in eukaryotes, 17 DEGs related to circadian rhythm–plant, and 13 DEGs related to phenylalanine metabolism were significantly up regulated (LinkS2) in LT (S1 Zip). However, 30 DEGs related to photosynthesis pathway, 13 DEGs related to photosynthesis-antenna,24 DEGs regulating carbon fixation in photosynthetic organisms, and 18 genes regulating porphyrin and chlorophyll metabolism were significantly down regulated in LT (S1 Zip).

### GO enrichment analysis of DEGs

**Fig 4:**
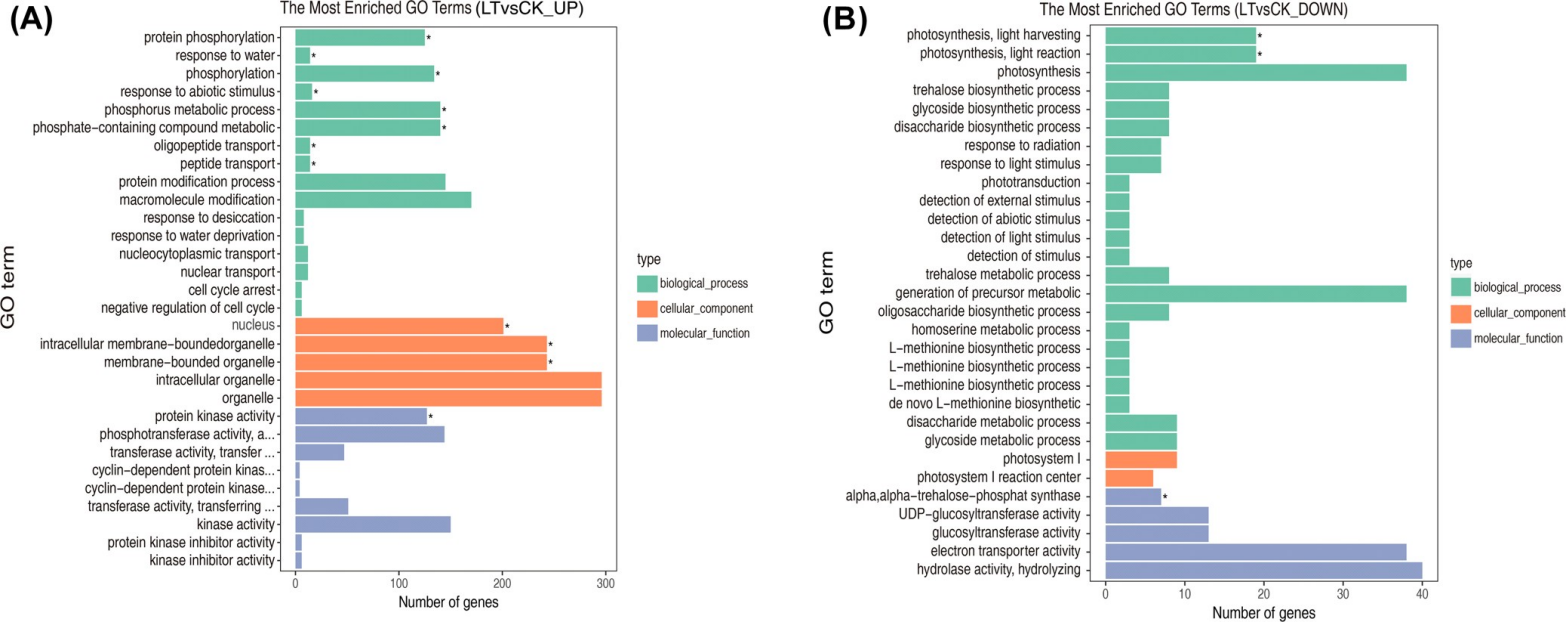
GO assignment of unigenes of leaves exposed to different temperatures. (A) Most enriched up-regulated GO terms of LT, compared with CK; B, most enriched down-regulated GO terms of LT, compared with CK. * represents the significantly expressed genes in the GO database. Green, orange, and blue bars represent categories of biological process, cellular component, and molecular function, respectively. CK: control (20°C); LT: low temperature (4°C).

LT significant up-regulated genes involved in oxidation resistance (e.g., membrane), while it significantly down-regulated genes involved in photosynthesis. (Fig 4, S7 Table and S8 Table). GO terms were significantly up-regulated in the “biological process” category, which were related to eight terms including protein phosphorylation, response to water, phosphorylation, response to abiotic stimulus, phosphorus metabolic process, phosphate-containing compound metabolic process, oligopeptide transport, and peptide transport (GO:0006468, GO:0009415, GO:0016310, GO:0009628, GO:0006793, GO:0006796, GO:0006857, and GO:0015833). In the “cell components” category, they were involved in membrane-bounded organelle (GO:0043227), intracellular membrane-bounded organelles (GO:0043231), and nucleus (GO:0005634). In the molecular category, they were associated with protein kinase activity (GO:0004672). (Fig 4A, S7 Table). Significantly down-regulated genes were mainly related to photosynthesis, light harvesting (GO:0009765), and photosynthesis light reaction (GO:0019684) in the “biological process” category and to alpha,alpha-trehalose-phosphate (GO:0003825) in the “molecular function” category (Fig 4B, S8 Table). These results implied that LT resulted in the upregulation of metabolic activity (e.g., phosphorylation and peptide transport), cellular structure change, and protein kinase activity, while it decreased the expression of genes involved in photosynthesis.

### Significantly enriched KEGG pathways in DEGs

**Fig 5:**
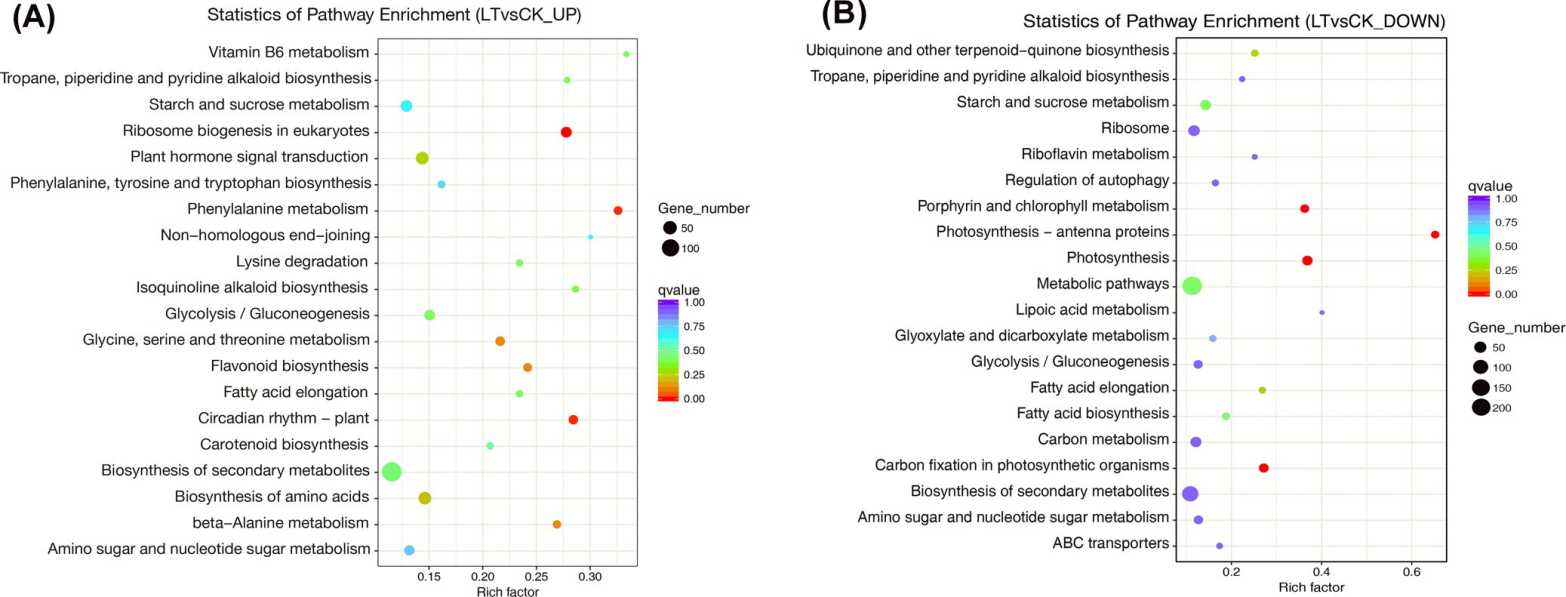
KEGG analysis of DEGs induced by LT treatment in the Lanzhou Lily. (A) Most up-regulated gene clusters of LT, compared with CK; (B) most down-regulated gene clusters of LT, compared with CK. The size of the dots indicates the number of enriched genes, while the color scale indicates the Q-value. Darker colors indicate more significant enrichment. CK: control (20°C); LT: low temperature (4°C).

KEGG enrichment analysis of DEGs was conducted to identify its pathways and molecule function (Fig 5, S9 and S10 Tables). The significantly upregulated genes identified via pathway enrichment analysis were related to ribosome biogenesis in eukaryotes (egu03008), phenylalanine metabolism (egu00360), and circadian rhythm in plants (egu04712), (Fig 5A, S9 Table). However, the pathways with significant enrichment of downregulated genes were related to porphyrin and chlorophyll metabolism (egu00860), photosynthetic-antenna protein (egu00196), photosynthesis (egu00195), and carbon fixation in photosynthetic organisms (egu00710) (Fig 5B, S10 Table). These results implied that LT stress may induce DEGs involved in the upregulation of intracellular biochemical material, change of biological rhythm, and downregulation related to photosynthesis and carbon fixation. Thus DEGs that are important for plant survival.

### RT-qPCR verification

**Fig 6:**
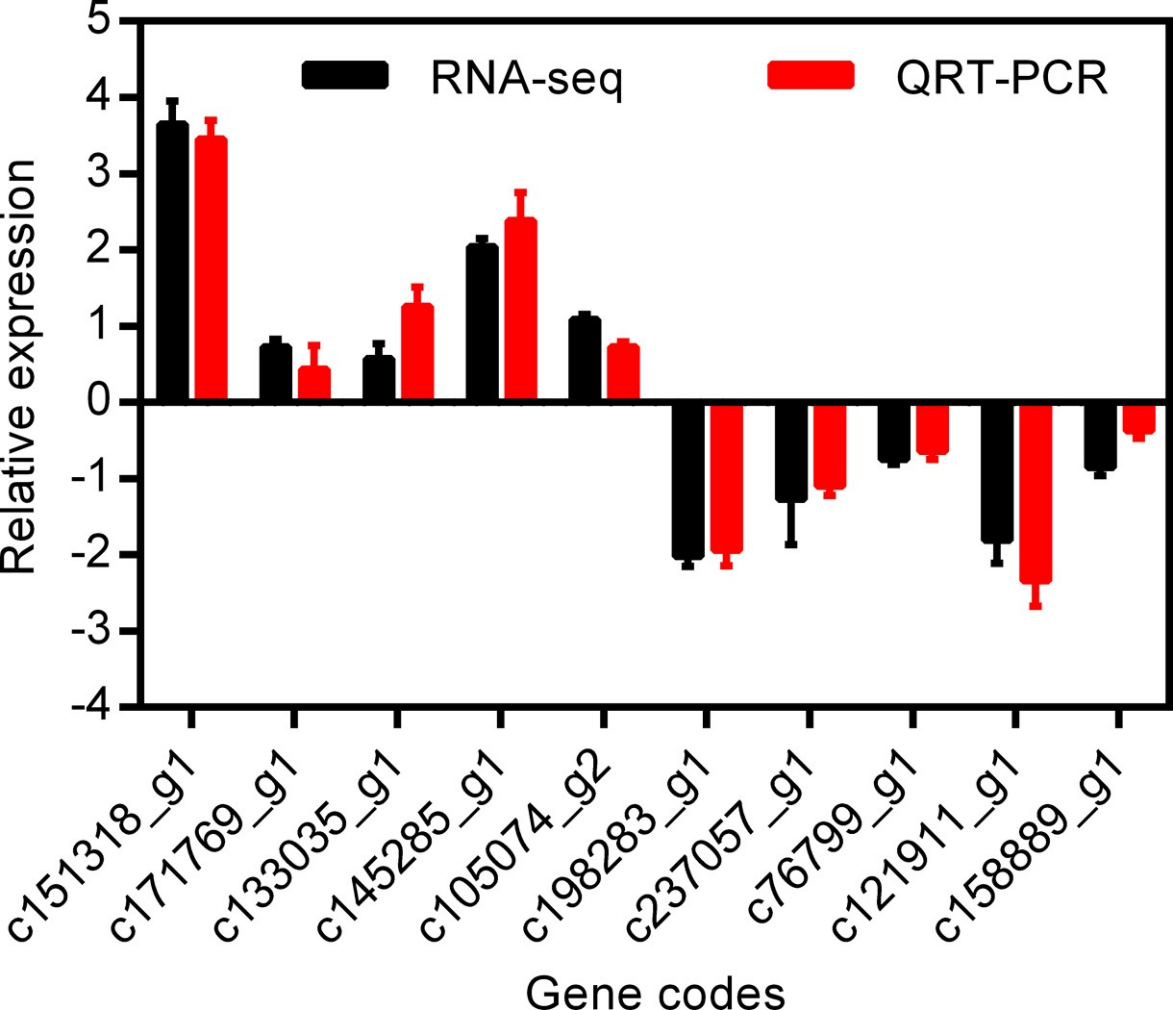
Validation of DEGs data by RT-qPCR. The relative expression levels of the selected genes were normalized to the expression level of the actin gene. The black bars and red bars represent relative expression level of LT to CK with RNA-seq and RT-qPCR methods, respectively. CK: control (20°C); LT: low temperature (4°C).

To verify the reliability of the RNA-seq results, 10 DEGs were randomly selected and their expression levels were measured using the qRT-PCR method (Fig 6). The results showed that these 10 genes were differentially expressed under LT, which confirmed the results of high-throughput sequencing. This indicated the existence of a close association between the expression changes determined by qRT-PCR and those by RNA-Seq methods. Consequently, the sequencing results are authentic and reliable.

### Gene clustering analysis of the 10 selected DEGs

**Fig 7:**
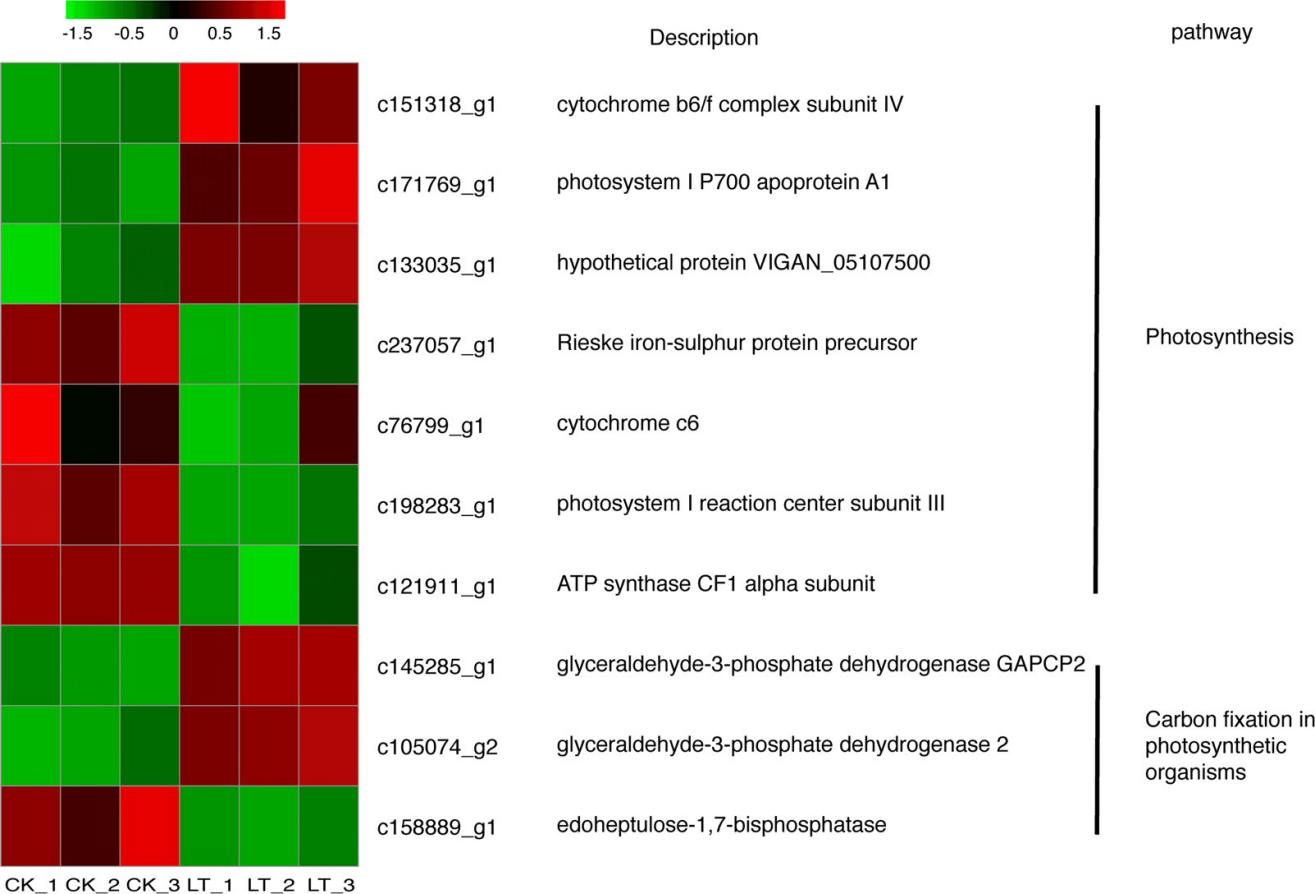
Heat-map representing the relative transcript abundance of 10 randomly selected DEGs in leaves of the Lanzhou Lily under both CK and LT. **These 10 DEGs are involved in photosynthesis and carbon fixation in photosynthetic organisms pathways.** The relative transcript abundance ranged from blue color (low, downregulated) to red color (high, upregulated). Expression ratios are calculated from log2 fragments per kilobase of transcript per million mapped reads (FPKM) values, where each vertical column represents one sample (CK-1, CK-2, and CK-3; LT-1, LT-2, and LT-3), and each horizontal row represents for a single gene. CK: control (20°C); LT: low temperature (4°C).

To further understand the function of the 10 randomly selected DEGs, gene function analysis and clustering analysis were conducted (Fig 7). The function of these genes is described in S1 Table. They mainly belonged to two common pathways: photosynthesis (including *c151318_g1*, *c171769_g1*, *c133035_g1*, *c237057_g1*, *c76799_g1*, *c121911_g1*, and *c198283_g1*) and carbon fixation in the photosynthetic process (including *c145285_g1*, *c105074_g2*, and *c158889_g1*). In addition, LT up-regulated the expression levels of *c151318_g1*, *c171769_g1*, and *c133035_g1*, while it down-regulated the expressions levels of *c237057_g1*, *c76799_g1*, *c121911_g1*, and *c198283_g1* in the photosynthesis pathway. Furthermore, LT up-regulated the expression levels of *c145285_g1* and *c105074_g2*, while it down-regulated the expression levels of *c158889_g1* in the carbon fixation in photosynthetic organisms pathway. This indicated that one pathway may be regulated by many genes, and one gene may participate in many pathways where it exerts different functions. Thus, such genes can mutually affect the plant response and lead to an adaptation to a stressful the outside environment, e.g., LT stress.

## Discussion

Many previous studies reported that lilies respond to cold stress via changes in cellular structure, production of resistance substances, or changes in physical activities [7,8,18]; however, little information is available on transcriptome level [4,44], especially at the early stage of vegetative growth of Lanzhou lily. Based on physiological investigation and transcriptome sequencing analysis, this study demonstrated that LT significantly changed both physiological function and biochemical contents. Moreover, it induced a series of related changes at the oranscriptome level (including the expression of related genes and pathways), which could explain physiological changes at the molecular level. The results lay a foundation for the understanding of the physiological and molecular mechanism of the Lanzhou Lily to LT stress.

The membrane is the first site for plants to perceive and respond to a variety of abiotic stresses, such as LT. It participates in signal transduction, energy conversion, metabolic regulation, and other physiological processes [50]. Rec represents the degree of membrane integrity injury, which is assumed to be affected first by chilling injury [61]. Rec increased under LT (Fig 1A), which was consistent with previous research [40], and indicated that LT treatment induced membrane injury. Photosynthesis, a key physiological process for plant growth and development, is affected by a variety of environmental factors, with temperature in particular [22]. In line with previous studies [24,31], low temperature significantly decreased Pn (Fig 1B). In addition, Fv/Fm represents the maximum quantum efficiency of photosystem II (PSII) photochemistry in the light-adapted state, and significantly decreased under LT treatment (Fig 1C). This indicates that LT induced photoinhibition of PSII [26,62]. In line with several studies on cold stress on growth of bulbs of the Lanzhou Lily, the contents of SS increased under LT stress (Fig 1E). This may be due to the degradation of sucrose into SS [18,63], thus decreasing the leaf water potential, while increasing its osmotic potential to resist chilling injury [8]. Pro, one of the key organic osmolytes, responds to a variety of stresses [64]. Wang et al. [4] reported that LT induced the expression of genes related to proline synthesis, which could explain the increased Pro concentration in the present study (Fig 1F). Furthermore, the increased SOD and POD activities, identified in this study (Fig 1G and 1H), are consistent with previous research on *Hemerocallis* [40] and Kiwifruit [55,57]. This has been considered to be a response to reversible injury, which could increase the defense capability of plants to a variety of abiotic stresses.

LT induced an abundance of DEGs (6,548; 3,566 were up-regulated, while 2,982 were down-regulated) (Fig 3). This indicates that LT may result in changes of many metabolic processes on a molecule level, thus providing important information to reveal the molecular mechanism of the physiological process under LT [4]. To classify the function of those DEGs, they were assigned to different categories including GO, KEGG, and KOG, and consequently, different groups were classified (Fig 2), which matches previous reports [36,41,44,48]. With regard to gene function classification based on the GO database, all DEGs were classified into 20 categories related to biological processes, 12 categories genes related to cellular components, and 10 categories genes related to molecular functions. This indicates that LT could induce dramatic metabolic changes in the leaves of the Lanzhou Lily on a molecular basis. In the molecular function, the “binding” and “catalytic activity” were the two most common groups (Fig 2A), which is consistent with the results of Liu et al. [46] and Hu et al. [37]. These genes covered the functions of electron carrier activity, structural molecule activity, and transporter activity [46], thus affecting metabolic processes in plants. Moreover, in the metabolic group on the KEGG database, the most enriched genes were related to “global and overview maps”, “carbohydrate metabolism”, “metabolism of cofactors and vitamins”, and “energy metabolism”. This partly matched the results of a previous study [43], since LT is widely considered to affect the growth and development by inhibiting the cell metabolism (e.g., carbon metabolism and energy transformation, which require the participation of energy and enzymes). In summary, the enriched genes in the KEGG database showed that an abundance of genes respond to LT, which present valuable targets for further investigations aimed to uncover the underlying molecular mechanisms.

LT resulted in a series of biochemical changes that involved antioxidants to alleviate LT stress. These included increases of Rec, antioxidant substances (SP, SS, and Pro), and activities of antioxidant enzymes (SOD and POD) (Fig 1), which could be explained at the transcriptome level. In the GO term enrichment analysis, upregulation of the phosphorylation metabolism, including eight related biological processes, indicated that LT increased cell metabolic activity, thus helped to resist LT stress. In addition, LT significantly upregulated genes related to membrane and protein kinase activity (Fig 4A, S7 Table). Moreover, the significantly enriched DEGs involved in phosphorylation may explain the upregulated activity of membranes (Fig 4A), since phosphorus is an important component of membranes, which partly matched Moellering et al. [65]. These results implied that LT could improve the membrane structure and the expression levels of related genes, thus improving oxidation resistance. KEGG enrichment analysis identified significant upregulation of genes related to ribosome biogenesis, phenylalanine metabolism, and circadian rhythm, which implied that LT may increase the availability of pathways related to changes in biochemical activities (Fig 5A, S9 Table).

As discussed above, this study clearly demonstrated that LT significantly decreased Pn and Fv/Fm (Fig 1B and 1C), which agrees with a previous study [25]. Moreover, this study showed that these decreases could be perfectly explained by both the significantly enriched GO terms and KEGG terms as indicated by transcriptome analysis. On the one hand, the GO terms showed that LT mainly significantly down-regulated genes associated with photosynthesis, light harvesting (GO:0009765), and photosynthesis, light reaction (GO:0019684) in the “biological process” category. Consequently, absorption and utilization of light energy are obstructed to decrease Pn (Fig 4B, S8 Table). On the other hand, the KEGG terms showed that the main significantly down-regulated pathways were involved in porphyrin and chlorophyll metabolism (egu00860), photosynthetic-antenna protein (egu00196), photosynthesis (egu00195), and carbon fixation in photosynthetic organisms (egu00710). Porphyrin is the key synthetic precursor of chlorophyll, and chlorophyll and photosynthetic-antenna protein are key pigments in the light capture system required for plant growth and photosynthesis; thus, LT downregulated the syntheses and significantly decreased Pn (Fig 5B, S10 Table). This implied that LT could induce a series of changes in genes related to photosynthesis and pathways, thus decreasing Pn.

The relative expression of 10 randomly selected genes (using qPCR) agreed well with the RNA-seq data, showing the same trends or no significant differences (three of them had significant differences, namely, c133035_g1, c105074_g2, and c158889_g1, which may be due to their low expression or other unknow factors). This indicates that the RNA-seq data are reliable and could be used to identify the molecular mechanism of the response of the Lanzhou Lily to LT stress. This study mainly focused on the molecule mechanism based on photosynthesis and antioxidant levels. However, additional relevant genes, as well as metabolites and pathways related to cold resistance in the Lanzhou Lily still need to be investigated to uncover the applied cold resistance mechanism.

## Conclusions

LT significantly decreased cell membrane stability, Pn, and Fv/Fm, while it increased the contents of osmotic conditional substances and the activities of antioxidant enzymes (SOD and POD) in the Lanzhou Lily, thus resisting LT stress. In addition, key genes and pathways related to photosynthesis and oxidation resistance were obtained via transcriptome analysis, to identify the underlying molecular mechanism of the physiological responses to LT stress. The physiological response of the Lanzhou Lily to LT stress could be explained through transcription sequencing on the molecular level. Moreover, the validation of 10 randomly selected DEGs by qPCR implied that the results of transcription sequencing were reliable, which provides a valuable reference for the development of SSR markers and for the molecular breeding of Lanzhou Lily varieties with high cold resistance.

## Supporting information

S1 Table Primers used for qRT-PCR analysis. (XLSX)

S2 Table Quality evaluation of RNA-seq data. CK: control (20°C); LT: low temperature (4°C).
(XLS)

S3 Table Sequence number, length of N50, and total number of bases by different data processing methods. (XLS)

S4 Table Annotation statistics for all unigenes aligned to seven protein databases. (XLS)

S5 Table Significantly enriched up-regulated DEGs of LTvsCK. CK: control (20°C); LT: low temperature (4°C).
(XLS)

S6 Table Significantly enriched down-regulated DEGs of LTvsCK. CK: control (20°C); LT: low temperature (4°C).
(XLS)

S7 Table Up-regulated GO enrichment DEGs of LTvsCK. CK: control (20°C); LT: low temperature (4°C).
(XLS)

S8 Table Down-regulated GO enrichment DEGs of LTvsCK. CK: control (20°C); LT: low temperature (4°C).
(XLS)

S9 Table Up-regulated KEGG pathways of LTvsCK. CK: control (20°C); LT: low temperature (4°C).
(XLS)

S10 Table Down-regulated KEGG pathways of LTvsCK. CK: control (20°C); LT: low temperature (4°C).
(XLS)

S1 Zip Most enriched up-regulated KEGG terms with the list of DEGs of LTvsCK. (Zip). CK: control (20°C); LT: low temperature (4°C).
(ZIP)

S2 Zip Most enriched down-regulated KEGG terms with the list of DEGs of LTvsCK. (Zip). CK: control (20°C); LT: low temperature (4°C).
(ZIP)
