# Supplementary material for: Physiological and transcriptomic responses of Lanzhou Lily (Lilium davidii, var. unicolor) to cold stress
Source: PLoS One. 2020 Jan 23;15(1):e0227921. doi: 10.1371/journal.pone.0227921 (PMC6977731; doi:10.1371/journal.pone.0227921)
Supplement: S1 Zip — (Zip). CK: control (20°C); LT: low temperature (4°C). (ZIP) [file pone.0227921.s011.zip › S1 Zip/LTvsCK_UP.html]

Pathway Enrichment

  

# The most enriched pathway terms

Statistic method: hypergeometric test

FDR correction method: Benjamini and Hochberg

| Term | Sample number | Background number | P-value | Corrected P-value | Gene\_id | KEGG\_ID/KO | Entrez ID | Gene name |
| --- | --- | --- | --- | --- | --- | --- | --- | --- |
| Ribosome biogenesis in eukaryotes | 26 | 94 | 1.73499201071e-05 | 0.001943191052 | c173926\_g1 c158781\_g1 c152959\_g1 c165011\_g1 c164010\_g2 c143131\_g1 c173393\_g1 c170819\_g1 c160458\_g1 c167244\_g1 c174677\_g1 c155970\_g1 c145725\_g1 c141305\_g1 c155498\_g1 c172223\_g1 c174699\_g1 c168172\_g1 c170751\_g1 c168781\_g1 c134306\_g1 c159038\_g1 c146280\_g1 c168253\_g1 c173905\_g2 c166893\_g1 | egu:105035524 egu:105058060 egu:105059424 egu:105032038 egu:105058060 egu:105053596 egu:105032124 egu:105037954 egu:105042460 egu:105054834 egu:105043717 egu:105045325 egu:105043116 egu:105058749 egu:105040095 egu:105054755 egu:105053627 egu:105045184 egu:105051030 egu:105046077 egu:105049533 egu:105051885 egu:105061517 egu:105059853 egu:105047430 egu:105042999 | 105035524 105058060 105059424 105032038 105058060 105053596 105032124 105037954 105042460 105054834 105043717 105045325 105043116 105058749 105040095 105054755 105053627 105045184 105051030 105046077 105049533 105051885 105061517 105059853 105047430 105042999 |  |
| Circadian rhythm - plant | 17 | 60 | 0.000378917408583 | 0.0212193748806 | c152959\_g1 c151401\_g1 c174283\_g3 c186285\_g1 c171563\_g1 c170144\_g1 c146896\_g1 c121976\_g1 c164886\_g1 c169857\_g1 c171275\_g1 c161583\_g1 c146896\_g3 c169999\_g5 c174513\_g3 c155146\_g1 c174513\_g1 | egu:105059424 egu:105050962 egu:105041113 egu:105040182 egu:105036385 egu:105040132 egu:105050962 egu:105040182 egu:105045005 egu:105035716 egu:105047555 egu:105040294 egu:105050962 egu:105035907 egu:105050962 egu:105036364 egu:105050962 | 105059424 105050962 105041113 105040182 105036385 105040132 105050962 105040182 105045005 105035716 105047555 105040294 105050962 105035907 105050962 105036364 105050962 |  |
| Phenylalanine metabolism | 13 | 40 | 0.000627987797823 | 0.0234448777854 | c167137\_g1 c168304\_g1 c134164\_g1 c168406\_g1 c168304\_g3 c168304\_g2 c167006\_g1 c148234\_g1 c166080\_g1 c162887\_g1 c168951\_g1 c165806\_g1 c170271\_g1 | egu:105055420 egu:105035781 egu:105053813 egu:105037948 egu:105055673 egu:105055673 egu:105044125 egu:105054501 egu:105055673 egu:105039619 egu:105055420 egu:105045995 egu:105044125 | 105055420 105035781 105053813 105037948 105055673 105055673 105044125 105054501 105055673 105039619 105055420 105045995 105044125 |  |
| Glycine, serine and threonine metabolism | 17 | 79 | 0.00477623910927 | 0.109486576551 | c175256\_g1 c167137\_g1 c157181\_g1 c148031\_g1 c167006\_g1 c134944\_g1 c151470\_g3 c119816\_g1 c166123\_g1 c153848\_g1 c173425\_g1 c116327\_g1 c156718\_g1 c163681\_g1 c168951\_g1 c151470\_g2 c170271\_g1 | egu:105052340 egu:105055420 egu:105046041 egu:105042090 egu:105044125 egu:105049537 egu:105046041 egu:105038209 egu:105058884 egu:105033631 egu:105053256 egu:105038419 egu:105056873 egu:105060274 egu:105055420 egu:105052340 egu:105044125 | 105052340 105055420 105046041 105042090 105044125 105049537 105046041 105038209 105058884 105033631 105053256 105038419 105056873 105060274 105055420 105052340 105044125 |  |
| beta-Alanine metabolism | 11 | 41 | 0.00555903687754 | 0.109486576551 | c167137\_g1 c173974\_g3 c149000\_g1 c148031\_g1 c167006\_g1 c159016\_g1 c151510\_g1 c163847\_g1 c158241\_g1 c168951\_g1 c170271\_g1 | egu:105055420 egu:105035697 egu:105058904 egu:105042090 egu:105044125 egu:105045148 egu:105041902 egu:105032618 egu:105035498 egu:105055420 egu:105044125 | 105055420 105035697 105058904 105042090 105044125 105045148 105041902 105032618 105035498 105055420 105044125 |  |
| Flavonoid biosynthesis | 13 | 54 | 0.00586535231524 | 0.109486576551 | c151401\_g1 c167553\_g1 c155053\_g1 c174513\_g3 c146896\_g1 c169857\_g1 c146719\_g1 c146896\_g3 c154616\_g1 c165806\_g1 c166729\_g1 c155146\_g1 c174513\_g1 | egu:105050962 egu:105035984 egu:105035842 egu:105050962 egu:105050962 egu:105035716 egu:105058232 egu:105050962 egu:105058071 egu:105045995 egu:105054663 egu:105036364 egu:105050962 | 105050962 105035984 105035842 105050962 105050962 105035716 105058232 105050962 105058071 105045995 105054663 105036364 105050962 |  |
| Biosynthesis of amino acids | 42 | 290 | 0.0139135780801 | 0.222617249281 | c175256\_g1 c158821\_g1 c134112\_g1 c188298\_g1 c134944\_g1 c170991\_g2 c166123\_g1 c129527\_g1 c156756\_g2 c156756\_g1 c140061\_g1 c167493\_g1 c157181\_g1 c159520\_g1 c145285\_g1 c170886\_g3 c119816\_g1 c156235\_g1 c165885\_g1 c116327\_g1 c156718\_g1 c151470\_g3 c151470\_g2 c152936\_g1 c105074\_g2 c172074\_g1 c155751\_g1 c173425\_g1 c161769\_g1 c170819\_g2 c168406\_g1 c155351\_g1 c148031\_g1 c155351\_g2 c157258\_g1 c164239\_g2 c163051\_g1 c162772\_g1 c157432\_g1 c163681\_g1 c103295\_g1 c153848\_g1 | egu:105052340 egu:105053882 egu:105040155 egu:105035292 egu:105049537 egu:105059450 egu:105058884 egu:105036609 egu:105058982 egu:105042489 egu:105032793 egu:105060774 egu:105046041 egu:105047057 egu:105059882 egu:105050474 egu:105038209 egu:105057764 egu:105041687 egu:105038419 egu:105056873 egu:105046041 egu:105052340 egu:105036609 egu:105051363 egu:105057280 egu:105058186 egu:105053256 egu:105038179 egu:105044276 egu:105037948 egu:105032793 egu:105042090 egu:105032793 egu:105039187 egu:105060182 egu:105053561 egu:105060382 egu:105059872 egu:105060274 egu:105048637 egu:105033631 | 105052340 105053882 105040155 105035292 105049537 105059450 105058884 105036609 105058982 105042489 105032793 105060774 105046041 105047057 105059882 105050474 105038209 105057764 105041687 105038419 105056873 105046041 105052340 105036609 105051363 105057280 105058186 105053256 105038179 105044276 105037948 105032793 105042090 105032793 105039187 105060182 105053561 105060382 105059872 105060274 105048637 105033631 |  |
| Plant hormone signal transduction | 41 | 288 | 0.0188242151146 | 0.263539011605 | c158029\_g1 c163360\_g1 c87233\_g1 c150290\_g1 c76349\_g1 c164404\_g1 c146802\_g3 c142660\_g1 c156282\_g1 c159509\_g4 c162387\_g1 c172818\_g4 c132627\_g1 c170101\_g1 c152357\_g1 c155948\_g1 c162356\_g1 c164022\_g1 c169345\_g1 c103312\_g1 c167605\_g2 c113301\_g1 c147612\_g1 c163407\_g1 c138921\_g1 c174574\_g2 c160379\_g1 c157415\_g1 c147464\_g1 c149381\_g1 c146567\_g1 c154443\_g1 c147714\_g1 c142840\_g2 c170704\_g1 c140543\_g1 c96913\_g1 c138925\_g1 c146349\_g1 c165448\_g1 c157217\_g1 | egu:105038536 egu:105045549 egu:105052243 egu:105059132 egu:105059035 egu:105040525 egu:105052748 egu:105061364 egu:105057151 egu:105043050 egu:105033019 egu:105040420 egu:105041307 egu:105032276 egu:105058608 egu:105050174 egu:105056896 egu:105032167 egu:105048226 egu:105041307 egu:105043524 egu:105043078 egu:105043791 egu:105051062 egu:105035162 egu:105042113 egu:105032167 egu:105060874 egu:105047258 egu:105035319 egu:105060907 egu:105032733 egu:105046557 egu:105055597 egu:105043422 egu:105044079 egu:105056896 egu:105043524 egu:105055850 egu:105046997 egu:105041307 | 105038536 105045549 105052243 105059132 105059035 105040525 105052748 105061364 105057151 105043050 105033019 105040420 105041307 105032276 105058608 105050174 105056896 105032167 105048226 105041307 105043524 105043078 105043791 105051062 105035162 105042113 105032167 105060874 105047258 105035319 105060907 105032733 105046557 105055597 105043422 105044079 105056896 105043524 105055850 105046997 105041307 |  |
| Isoquinoline alkaloid biosynthesis | 6 | 21 | 0.0293751538403 | 0.365557470012 | c167137\_g1 c168406\_g1 c167006\_g1 c172129\_g1 c168951\_g1 c170271\_g1 | egu:105055420 egu:105037948 egu:105044125 egu:105048962 egu:105055420 egu:105044125 | 105055420 105037948 105044125 105048962 105055420 105044125 |  |
| Lysine degradation | 7 | 30 | 0.043334513098 | 0.394860183864 | c170170\_g1 c148031\_g1 c158212\_g1 c173126\_g1 c159372\_g1 c165534\_g2 c165534\_g1 | egu:105049737 egu:105042090 egu:105034748 egu:105045520 egu:105053701 egu:105043130 egu:105043130 | 105049737 105042090 105034748 105045520 105053701 105043130 105043130 |  |
| Fatty acid elongation | 7 | 30 | 0.043334513098 | 0.394860183864 | c166224\_g1 c161171\_g2 c161171\_g1 c163278\_g1 c159288\_g1 c162055\_g1 c168511\_g1 | egu:105041077 egu:105053416 egu:105053416 egu:105042699 egu:105058603 egu:105045727 egu:105052984 | 105041077 105053416 105053416 105042699 105058603 105045727 105052984 |  |
| Glycolysis / Gluconeogenesis | 23 | 154 | 0.0444731514022 | 0.394860183864 | c175256\_g1 c170590\_g5 c170590\_g8 c156756\_g2 c156756\_g1 c162039\_g1 c167493\_g1 c157181\_g1 c158409\_g1 c119816\_g1 c145285\_g1 c156718\_g1 c151470\_g3 c151470\_g2 c105074\_g2 c172074\_g1 c188298\_g1 c161769\_g1 c172556\_g1 c148031\_g1 c158821\_g1 c163051\_g1 c157432\_g1 | egu:105052340 egu:105039431 egu:105039431 egu:105058982 egu:105042489 egu:105059487 egu:105060774 egu:105046041 egu:105050719 egu:105038209 egu:105059882 egu:105056873 egu:105046041 egu:105052340 egu:105051363 egu:105057280 egu:105035292 egu:105038179 egu:105045855 egu:105042090 egu:105053882 egu:105053561 egu:105059872 | 105052340 105039431 105039431 105058982 105042489 105059487 105060774 105046041 105050719 105038209 105059882 105056873 105046041 105052340 105051363 105057280 105035292 105038179 105045855 105042090 105053882 105053561 105059872 |  |
| Vitamin B6 metabolism | 4 | 12 | 0.0487200973084 | 0.394860183864 | c161228\_g1 c224416\_g1 c173425\_g1 c173447\_g1 | egu:105047312 egu:105035898 egu:105053256 egu:105047312 | 105047312 105035898 105053256 105047312 |  |
| Tropane, piperidine and pyridine alkaloid biosynthesis | 5 | 18 | 0.0493575229831 | 0.394860183864 | c167137\_g1 c168951\_g1 c170271\_g1 c167006\_g1 c168406\_g1 | egu:105055420 egu:105055420 egu:105044125 egu:105044125 egu:105037948 | 105055420 105055420 105044125 105044125 105037948 |  |
| Biosynthesis of secondary metabolites | 134 | 1184 | 0.0598186577764 | 0.446645978064 | c170590\_g5 c170590\_g8 c146169\_g1 c162039\_g1 c168403\_g1 c173509\_g1 c170819\_g2 c121963\_g1 c162655\_g1 c162165\_g1 c161769\_g1 c166080\_g1 c166861\_g1 c166224\_g1 c169830\_g1 c133447\_g2 c161281\_g1 c169649\_g1 c140061\_g1 c167553\_g1 c155351\_g1 c163278\_g1 c133817\_g1 c165048\_g1 c148234\_g1 c166548\_g2 c163051\_g1 c162772\_g1 c114481\_g1 c103295\_g1 c145522\_g1 c155351\_g2 c134164\_g1 c156756\_g2 c172256\_g4 c157258\_g1 c166197\_g1 c164800\_g1 c169857\_g1 c167493\_g1 c159520\_g1 c172938\_g7 c129527\_g1 c168117\_g1 c119816\_g1 c163397\_g1 c165885\_g1 c168575\_g1 c168511\_g1 c156970\_g1 c156718\_g1 c165623\_g2 c105074\_g2 c161779\_g15 c160696\_g1 c167196\_g2 c154095\_g1 c141199\_g1 c117443\_g1 c148031\_g1 c172129\_g1 c156235\_g1 c162055\_g1 c121798\_g1 c162887\_g1 c140986\_g1 c174513\_g3 c174513\_g1 c175256\_g1 c168304\_g3 c168304\_g2 c168304\_g1 c166123\_g1 c173762\_g1 c152294\_g1 c163448\_g1 c167006\_g1 c165806\_g1 c154616\_g1 c170127\_g1 c146896\_g1 c146896\_g3 c152936\_g1 c104905\_g1 c151510\_g1 c170749\_g4 c170749\_g3 c159288\_g1 c167245\_g1 c151855\_g2 c155636\_g1 c170271\_g1 c155636\_g2 c170991\_g2 c172556\_g1 c153824\_g1 c162048\_g1 c158821\_g1 c155146\_g1 c149166\_g1 c135007\_g1 c161171\_g1 c161171\_g2 c134112\_g1 c156756\_g1 c155053\_g1 c153848\_g1 c146719\_g1 c166729\_g1 c150645\_g1 c151401\_g1 c157181\_g1 c159016\_g1 c145285\_g1 c172946\_g1 c116327\_g1 c151470\_g3 c151470\_g2 c167137\_g1 c155459\_g1 c172074\_g1 c151973\_g1 c155751\_g1 c188298\_g1 c163681\_g1 c168951\_g1 c170056\_g1 c168406\_g1 c168307\_g1 c164239\_g2 c170886\_g3 c137525\_g1 c157432\_g1 c165035\_g1 | egu:105039431 egu:105039431 egu:105034095 egu:105059487 egu:105036591 egu:105035937 egu:105044276 egu:105035499 egu:105034258 egu:105056718 egu:105038179 egu:105055673 egu:105060488 egu:105041077 egu:105051975 egu:105032435 egu:105059274 egu:105058113 egu:105032793 egu:105035984 egu:105032793 egu:105042699 egu:105045139 egu:105041644 egu:105054501 egu:105039328 egu:105053561 egu:105060382 egu:105053391 egu:105048637 egu:105058543 egu:105032793 egu:105053813 egu:105058982 egu:105042391 egu:105039187 egu:105047182 egu:105046237 egu:105035716 egu:105060774 egu:105047057 egu:105038037 egu:105036609 egu:105046802 egu:105038209 egu:105060532 egu:105041687 egu:105041372 egu:105052984 egu:105055939 egu:105056873 egu:105033970 egu:105051363 egu:105037657 egu:105046158 egu:105043827 egu:105034598 egu:105040573 egu:105052990 egu:105042090 egu:105048962 egu:105057764 egu:105045727 egu:105034997 egu:105039619 egu:105056224 egu:105050962 egu:105050962 egu:105052340 egu:105055673 egu:105055673 egu:105035781 egu:105058884 egu:105053112 egu:105055883 egu:105055201 egu:105044125 egu:105045995 egu:105058071 egu:105041133 egu:105050962 egu:105050962 egu:105036609 egu:105053391 egu:105041902 egu:105045199 egu:105045199 egu:105058603 egu:105054164 egu:105053370 egu:105042391 egu:105044125 egu:105042391 egu:105059450 egu:105045855 egu:105041633 egu:105046802 egu:105053882 egu:105036364 egu:105060729 egu:105036034 egu:105053416 egu:105053416 egu:105040155 egu:105042489 egu:105035842 egu:105033631 egu:105058232 egu:105054663 egu:105053413 egu:105050962 egu:105046041 egu:105045148 egu:105059882 egu:105038559 egu:105038419 egu:105046041 egu:105052340 egu:105055420 egu:105059350 egu:105057280 egu:105038882 egu:105058186 egu:105035292 egu:105060274 egu:105055420 egu:105037657 egu:105037948 egu:105038499 egu:105060182 egu:105050474 egu:105052944 egu:105059872 egu:105050729 | 105039431 105039431 105034095 105059487 105036591 105035937 105044276 105035499 105034258 105056718 105038179 105055673 105060488 105041077 105051975 105032435 105059274 105058113 105032793 105035984 105032793 105042699 105045139 105041644 105054501 105039328 105053561 105060382 105053391 105048637 105058543 105032793 105053813 105058982 105042391 105039187 105047182 105046237 105035716 105060774 105047057 105038037 105036609 105046802 105038209 105060532 105041687 105041372 105052984 105055939 105056873 105033970 105051363 105037657 105046158 105043827 105034598 105040573 105052990 105042090 105048962 105057764 105045727 105034997 105039619 105056224 105050962 105050962 105052340 105055673 105055673 105035781 105058884 105053112 105055883 105055201 105044125 105045995 105058071 105041133 105050962 105050962 105036609 105053391 105041902 105045199 105045199 105058603 105054164 105053370 105042391 105044125 105042391 105059450 105045855 105041633 105046802 105053882 105036364 105060729 105036034 105053416 105053416 105040155 105042489 105035842 105033631 105058232 105054663 105053413 105050962 105046041 105045148 105059882 105038559 105038419 105046041 105052340 105055420 105059350 105057280 105038882 105058186 105035292 105060274 105055420 105037657 105037948 105038499 105060182 105050474 105052944 105059872 105050729 |  |
| Carotenoid biosynthesis | 7 | 34 | 0.0695870710951 | 0.487109497666 | c173509\_g1 c173485\_g1 c168301\_g1 c168117\_g1 c163397\_g1 c162048\_g1 c154973\_g1 | egu:105035937 egu:105033850 egu:105038273 egu:105046802 egu:105060532 egu:105046802 egu:105058241 | 105035937 105033850 105038273 105046802 105060532 105046802 105058241 |  |
| Starch and sucrose metabolism | 31 | 243 | 0.103911401026 | 0.647695250988 | c163448\_g1 c172256\_g4 c141054\_g1 c159060\_g1 c166197\_g1 c167554\_g2 c169469\_g1 c172045\_g1 c173495\_g2 c148722\_g1 c166861\_g1 c166556\_g1 c174721\_g1 c170749\_g4 c170749\_g3 c155636\_g1 c164911\_g1 c155636\_g2 c149471\_g1 c169649\_g1 c171913\_g1 c169422\_g1 c79219\_g1 c157266\_g1 c156393\_g1 c156393\_g2 c158824\_g1 c133326\_g1 c122873\_g1 c165538\_g1 c123004\_g1 | egu:105055201 egu:105042391 egu:105033487 egu:105043204 egu:105047182 egu:105055983 egu:105045186 egu:105049657 egu:105060892 egu:105046839 egu:105060488 egu:105058580 egu:105043800 egu:105045199 egu:105045199 egu:105042391 egu:105055260 egu:105042391 egu:105040297 egu:105058113 egu:105034870 egu:105035608 egu:105043204 egu:105056682 egu:105039053 egu:105050124 egu:105054034 egu:105035371 egu:105055983 egu:105049657 egu:105056063 | 105055201 105042391 105033487 105043204 105047182 105055983 105045186 105049657 105060892 105046839 105060488 105058580 105043800 105045199 105045199 105042391 105055260 105042391 105040297 105058113 105034870 105035608 105043204 105056682 105039053 105050124 105054034 105035371 105055983 105049657 105056063 |  |
| Non-homologous end-joining | 3 | 10 | 0.104093879623 | 0.647695250988 | c160026\_g38 c163082\_g1 c159313\_g1 | egu:105049145 egu:105046549 egu:105039319 | 105049145 105046549 105039319 |  |
| Phenylalanine, tyrosine and tryptophan biosynthesis | 9 | 56 | 0.124307325674 | 0.732758972395 | c168406\_g1 c134112\_g1 c170819\_g2 c164239\_g2 c129527\_g1 c152936\_g1 c165885\_g1 c163681\_g1 c103295\_g1 | egu:105037948 egu:105040155 egu:105044276 egu:105060182 egu:105036609 egu:105036609 egu:105041687 egu:105060274 egu:105048637 | 105037948 105040155 105044276 105060182 105036609 105036609 105041687 105060274 105048637 |  |
| Amino sugar and nucleotide sugar metabolism | 21 | 161 | 0.137180637843 | 0.76821157192 | c166861\_g1 c133326\_g1 c172397\_g1 c163736\_g1 c167554\_g2 c169422\_g1 c169469\_g1 c172816\_g4 c164458\_g2 c171416\_g1 c159575\_g2 c164111\_g1 c164898\_g1 c164458\_g1 c72023\_g1 c122873\_g1 c164911\_g1 c161991\_g1 c123004\_g1 c166197\_g1 c150645\_g1 | egu:105060488 egu:105035371 egu:105036046 egu:105042572 egu:105055983 egu:105035608 egu:105045186 egu:105060220 egu:105042574 egu:105040562 egu:105047457 egu:105057156 egu:105049059 egu:105042574 egu:105046676 egu:105055983 egu:105055260 egu:105047536 egu:105056063 egu:105047182 egu:105053413 | 105060488 105035371 105036046 105042572 105055983 105035608 105045186 105060220 105042574 105040562 105047457 105057156 105049059 105042574 105046676 105055983 105055260 105047536 105056063 105047182 105053413 |  |
| Sesquiterpenoid and triterpenoid biosynthesis | 2 | 6 | 0.15639767552 | 0.790028252694 | c152294\_g1 c121798\_g1 | egu:105055883 egu:105034997 | 105055883 105034997 |  |
| Cyanoamino acid metabolism | 7 | 43 | 0.156455681247 | 0.790028252694 | c168674\_g1 c116327\_g1 c170749\_g4 c170749\_g3 c172256\_g4 c155636\_g1 c155636\_g2 | egu:105044445 egu:105038419 egu:105045199 egu:105045199 egu:105042391 egu:105042391 egu:105042391 | 105044445 105038419 105045199 105045199 105042391 105042391 105042391 |  |
| Carbon metabolism | 38 | 322 | 0.16223794475 | 0.790028252694 | c175256\_g1 c158821\_g1 c173425\_g1 c134944\_g1 c163723\_g1 c156756\_g2 c156756\_g1 c162039\_g1 c140061\_g1 c167493\_g1 c157181\_g1 c159520\_g1 c170886\_g3 c159016\_g1 c158409\_g1 c119816\_g1 c145285\_g1 c170991\_g2 c116327\_g1 c156718\_g1 c151470\_g3 c166833\_g1 c155459\_g1 c105074\_g2 c133447\_g2 c188298\_g1 c161769\_g1 c151470\_g2 c172074\_g1 c155351\_g1 c162772\_g1 c155351\_g2 c117443\_g1 c135926\_g1 c163051\_g1 c149166\_g1 c157432\_g1 c145522\_g1 | egu:105052340 egu:105053882 egu:105053256 egu:105049537 egu:105058702 egu:105058982 egu:105042489 egu:105059487 egu:105032793 egu:105060774 egu:105046041 egu:105047057 egu:105050474 egu:105045148 egu:105050719 egu:105038209 egu:105059882 egu:105059450 egu:105038419 egu:105056873 egu:105046041 egu:105043985 egu:105059350 egu:105051363 egu:105032435 egu:105035292 egu:105038179 egu:105052340 egu:105057280 egu:105032793 egu:105060382 egu:105032793 egu:105052990 egu:105047511 egu:105053561 egu:105060729 egu:105059872 egu:105058543 | 105052340 105053882 105053256 105049537 105058702 105058982 105042489 105059487 105032793 105060774 105046041 105047057 105050474 105045148 105050719 105038209 105059882 105059450 105038419 105056873 105046041 105043985 105059350 105051363 105032435 105035292 105038179 105052340 105057280 105032793 105060382 105032793 105052990 105047511 105053561 105060729 105059872 105058543 |  |
| Galactose metabolism | 9 | 62 | 0.182348894964 | 0.850961509833 | c167493\_g1 c163736\_g1 c167034\_g1 c158824\_g1 c164267\_g1 c147292\_g1 c141054\_g1 c164911\_g1 c171613\_g1 | egu:105060774 egu:105042572 egu:105057305 egu:105054034 egu:105056490 egu:105038174 egu:105033487 egu:105055260 egu:105034931 | 105060774 105042572 105057305 105054034 105056490 105038174 105033487 105055260 105034931 |  |
| Tyrosine metabolism | 6 | 39 | 0.211905776981 | 0.876832931953 | c167137\_g1 c168406\_g1 c167006\_g1 c172129\_g1 c168951\_g1 c170271\_g1 | egu:105055420 egu:105037948 egu:105044125 egu:105048962 egu:105055420 egu:105044125 | 105055420 105037948 105044125 105048962 105055420 105044125 |  |
| Taurine and hypotaurine metabolism | 3 | 15 | 0.214642924987 | 0.876832931953 | c151510\_g1 c168674\_g1 c162417\_g1 | egu:105041902 egu:105044445 egu:105047825 | 105041902 105044445 105047825 |  |
| Arachidonic acid metabolism | 3 | 15 | 0.214642924987 | 0.876832931953 | c168674\_g1 c159727\_g1 c159549\_g1 | egu:105044445 egu:105042890 egu:105044026 | 105044445 105042890 105044026 |  |
| Mismatch repair | 7 | 48 | 0.219208232988 | 0.876832931953 | c173300\_g1 c167546\_g1 c172733\_g8 c169225\_g4 c139006\_g1 c168792\_g1 c160857\_g1 | egu:105054823 egu:105057784 egu:105053216 egu:105036598 egu:105040945 egu:105044402 egu:105046296 | 105054823 105057784 105053216 105036598 105040945 105044402 105046296 |  |
| Arginine and proline metabolism | 8 | 58 | 0.236310837017 | 0.884333454915 | c173974\_g3 c149000\_g1 c148031\_g1 c157509\_g1 c170577\_g2 c154095\_g1 c163847\_g1 c158241\_g1 | egu:105035697 egu:105058904 egu:105042090 egu:105050758 egu:105035222 egu:105034598 egu:105032618 egu:105035498 | 105035697 105058904 105042090 105050758 105035222 105034598 105032618 105035498 |  |
| Glycerophospholipid metabolism | 14 | 112 | 0.236875032567 | 0.884333454915 | c164783\_g1 c165623\_g2 c133817\_g1 c171054\_g2 c160696\_g1 c162877\_g1 c170127\_g1 c167245\_g1 c123587\_g1 c168575\_g1 c152491\_g2 c152491\_g1 c172120\_g1 c164800\_g1 | egu:105055852 egu:105033970 egu:105045139 egu:105050243 egu:105046158 egu:105033167 egu:105041133 egu:105054164 egu:105057482 egu:105041372 egu:105061179 egu:105061179 egu:105044829 egu:105046237 | 105055852 105033970 105045139 105050243 105046158 105033167 105041133 105054164 105057482 105041372 105061179 105061179 105044829 105046237 |  |
| Regulation of autophagy | 6 | 43 | 0.272875683758 | 0.985873438093 | c126406\_g1 c155868\_g1 c173414\_g1 c148972\_g1 c170446\_g1 c159535\_g1 | egu:105048117 egu:105060190 egu:105060190 egu:105048117 egu:105057639 egu:105045507 | 105048117 105060190 105060190 105048117 105057639 105045507 |  |
| Ubiquinone and other terpenoid-quinone biosynthesis | 5 | 36 | 0.304101347422 | 0.999951598671 | c162887\_g1 c137525\_g1 c165806\_g1 c141199\_g1 c134164\_g1 | egu:105039619 egu:105052944 egu:105045995 egu:105040573 egu:105053813 | 105039619 105052944 105045995 105040573 105053813 |  |
| Phenylpropanoid biosynthesis | 19 | 168 | 0.316049181018 | 0.999951598671 | c155636\_g2 c169830\_g1 c172938\_g7 c168304\_g2 c168304\_g1 c170749\_g4 c170749\_g3 c161779\_g15 c168304\_g3 c148234\_g1 c162887\_g1 c140986\_g1 c172256\_g4 c166080\_g1 c155636\_g1 c170056\_g1 c165806\_g1 c165035\_g1 c134164\_g1 | egu:105042391 egu:105051975 egu:105038037 egu:105055673 egu:105035781 egu:105045199 egu:105045199 egu:105037657 egu:105055673 egu:105054501 egu:105039619 egu:105056224 egu:105042391 egu:105055673 egu:105042391 egu:105037657 egu:105045995 egu:105050729 egu:105053813 | 105042391 105051975 105038037 105055673 105035781 105045199 105045199 105037657 105055673 105054501 105039619 105056224 105042391 105055673 105042391 105037657 105045995 105050729 105053813 |  |
| Nitrogen metabolism | 5 | 38 | 0.340525675612 | 0.999951598671 | c168315\_g1 c170868\_g1 c169807\_g1 c157258\_g1 c167975\_g1 | egu:105060899 egu:105041350 egu:105046269 egu:105039187 egu:105035363 | 105060899 105041350 105046269 105039187 105035363 |  |
| Pyruvate metabolism | 12 | 104 | 0.344380396023 | 0.999951598671 | c188298\_g1 c148031\_g1 c158821\_g1 c163723\_g1 c172074\_g1 c165048\_g1 c156756\_g2 c156756\_g1 c166833\_g1 c162039\_g1 c161769\_g1 c145522\_g1 | egu:105035292 egu:105042090 egu:105053882 egu:105058702 egu:105057280 egu:105041644 egu:105058982 egu:105042489 egu:105043985 egu:105059487 egu:105038179 egu:105058543 | 105035292 105042090 105053882 105058702 105057280 105041644 105058982 105042489 105043985 105059487 105038179 105058543 |  |
| Ascorbate and aldarate metabolism | 6 | 48 | 0.353783764166 | 0.999951598671 | c167554\_g2 c168307\_g1 c148031\_g1 c122873\_g1 c135007\_g1 c150645\_g1 | egu:105055983 egu:105038499 egu:105042090 egu:105055983 egu:105036034 egu:105053413 | 105055983 105038499 105042090 105055983 105036034 105053413 |  |
| Glutathione metabolism | 12 | 105 | 0.35544634875 | 0.999951598671 | c173732\_g2 c168674\_g1 c165975\_g1 c152293\_g1 c163752\_g1 c161851\_g3 c160646\_g1 c133447\_g2 c132043\_g1 c163232\_g1 c159727\_g1 c172086\_g3 | egu:105037855 egu:105044445 egu:105037855 egu:105032151 egu:105058473 egu:105042120 egu:105056588 egu:105032435 egu:105058893 egu:105048525 egu:105042890 egu:105032431 | 105037855 105044445 105037855 105032151 105058473 105042120 105056588 105032435 105058893 105048525 105042890 105032431 |  |
| RNA transport | 22 | 205 | 0.381135797606 | 0.999951598671 | c147937\_g1 c171650\_g1 c155321\_g1 c169995\_g2 c172809\_g1 c147291\_g1 c167332\_g2 c158591\_g1 c169217\_g3 c168229\_g1 c837\_g1 c141122\_g1 c145725\_g1 c174078\_g4 c166802\_g1 c158413\_g1 c164695\_g7 c72097\_g1 c169579\_g1 c165981\_g1 c159038\_g1 c167428\_g1 | egu:105041107 egu:105048848 egu:105046248 egu:105058622 egu:105056865 egu:105056889 egu:105046005 egu:105058399 egu:105045408 egu:105056837 egu:105035788 egu:105056426 egu:105043116 egu:105052955 egu:105050885 egu:105047630 egu:105050873 egu:105051987 egu:105035083 egu:105040546 egu:105051885 egu:105049252 | 105041107 105048848 105046248 105058622 105056865 105056889 105046005 105058399 105045408 105056837 105035788 105056426 105043116 105052955 105050885 105047630 105050873 105051987 105035083 105040546 105051885 105049252 |  |
| Zeatin biosynthesis | 3 | 22 | 0.390693314036 | 0.999951598671 | c170100\_g2 c166291\_g1 c173887\_g1 | egu:105034779 egu:105053404 egu:105036092 | 105034779 105053404 105036092 |  |
| Pentose phosphate pathway | 9 | 80 | 0.403101256048 | 0.999951598671 | c170590\_g5 c167493\_g1 c155459\_g1 c159520\_g1 c162772\_g1 c158409\_g1 c170590\_g8 c133447\_g2 c170991\_g2 | egu:105039431 egu:105060774 egu:105059350 egu:105047057 egu:105060382 egu:105050719 egu:105039431 egu:105032435 egu:105059450 | 105039431 105060774 105059350 105047057 105060382 105050719 105039431 105032435 105059450 |  |
| Ether lipid metabolism | 4 | 32 | 0.405186637478 | 0.999951598671 | c167245\_g1 c133817\_g1 c165623\_g2 c164800\_g1 | egu:105054164 egu:105045139 egu:105033970 egu:105046237 | 105054164 105045139 105033970 105046237 |  |
| Glycosylphosphatidylinositol(GPI)-anchor biosynthesis | 3 | 23 | 0.415622947507 | 0.999951598671 | c167649\_g1 c170429\_g1 c150889\_g1 | egu:105059739 egu:105039157 egu:105048800 | 105059739 105039157 105048800 |  |
| Glycosphingolipid biosynthesis - ganglio series | 1 | 5 | 0.430567397756 | 0.999951598671 | c172397\_g1 | egu:105036046 | 105036046 |  |
| Sulfur relay system | 2 | 15 | 0.457937521487 | 0.999951598671 | c162305\_g1 c158107\_g1 | egu:105033260 egu:105048348 | 105033260 105048348 |  |
| RNA polymerase | 5 | 45 | 0.468157787962 | 0.999951598671 | c171631\_g6 c166229\_g1 c174522\_g5 c107073\_g1 c171895\_g1 | egu:105034397 egu:105047342 egu:12079509 egu:105039834 egu:105060985 | 105034397 105047342 12079509 105039834 105060985 |  |
| Biotin metabolism | 3 | 26 | 0.488099891968 | 0.999951598671 | c167962\_g1 c173502\_g2 c163639\_g1 | egu:105056688 egu:105051431 egu:105039255 | 105056688 105051431 105039255 |  |
| Linoleic acid metabolism | 2 | 16 | 0.48834934605 | 0.999951598671 | c173762\_g1 c162165\_g1 | egu:105053112 egu:105056718 | 105053112 105056718 |  |
| Valine, leucine and isoleucine biosynthesis | 2 | 17 | 0.517666964887 | 0.999951598671 | c155751\_g1 c156235\_g1 | egu:105058186 egu:105057764 | 105058186 105057764 |  |
| DNA replication | 6 | 59 | 0.531989896019 | 0.999951598671 | c167546\_g1 c169225\_g4 c139006\_g1 c168792\_g1 c171707\_g2 c160857\_g1 | egu:105057784 egu:105036598 egu:105040945 egu:105044402 egu:105052111 egu:105046296 | 105057784 105036598 105040945 105044402 105052111 105046296 |  |
| Cutin, suberine and wax biosynthesis | 3 | 28 | 0.533900153678 | 0.999951598671 | c167289\_g1 c160216\_g1 c160204\_g2 | egu:105044187 egu:105061386 egu:105046261 | 105044187 105061386 105046261 |  |
| C5-Branched dibasic acid metabolism | 1 | 8 | 0.57034533389 | 0.999951598671 | c156235\_g1 | egu:105057764 | 105057764 |  |
| One carbon pool by folate | 2 | 19 | 0.572844662258 | 0.999951598671 | c135926\_g1 c116327\_g1 | egu:105047511 egu:105038419 | 105047511 105038419 |  |
| Homologous recombination | 5 | 52 | 0.587122444949 | 0.999951598671 | c168792\_g1 c160026\_g38 c139006\_g1 c161001\_g1 c171499\_g1 | egu:105044402 egu:105049145 egu:105040945 egu:105056344 egu:105042050 | 105044402 105049145 105040945 105056344 105042050 |  |
| Synthesis and degradation of ketone bodies | 1 | 9 | 0.608854127987 | 0.999951598671 | c146169\_g1 | egu:105034095 | 105034095 |  |
| Purine metabolism | 17 | 181 | 0.60976497489 | 0.999951598671 | c169556\_g1 c168403\_g1 c174522\_g5 c171631\_g6 c188298\_g1 c121963\_g1 c172074\_g1 c158821\_g1 c161851\_g3 c171895\_g1 c151895\_g1 c107073\_g1 c161769\_g1 c156756\_g1 c166229\_g1 c172086\_g3 c156756\_g2 | egu:105058802 egu:105036591 egu:12079509 egu:105034397 egu:105035292 egu:105035499 egu:105057280 egu:105053882 egu:105042120 egu:105060985 egu:105060687 egu:105039834 egu:105038179 egu:105042489 egu:105047342 egu:105032431 egu:105058982 | 105058802 105036591 12079509 105034397 105035292 105035499 105057280 105053882 105042120 105060985 105060687 105039834 105038179 105042489 105047342 105032431 105058982 |  |
| Sulfur metabolism | 4 | 43 | 0.617278906135 | 0.999951598671 | c140061\_g1 c155351\_g1 c155351\_g2 c151895\_g1 | egu:105032793 egu:105032793 egu:105032793 egu:105060687 | 105032793 105032793 105032793 105060687 |  |
| N-Glycan biosynthesis | 5 | 55 | 0.633435638073 | 0.999951598671 | c79137\_g1 c158910\_g1 c134078\_g1 c94583\_g1 c158181\_g1 | egu:105044631 egu:105040617 egu:105051251 egu:105046530 egu:105039124 | 105044631 105040617 105051251 105046530 105039124 |  |
| Spliceosome | 21 | 227 | 0.640090523398 | 0.999951598671 | c173699\_g2 c162615\_g1 c165965\_g3 c174104\_g1 c164201\_g2 c172808\_g4 c167816\_g1 c155331\_g2 c141698\_g1 c160307\_g1 c163181\_g5 c164662\_g3 c141698\_g2 c173165\_g1 c167228\_g1 c161568\_g3 c165278\_g1 c139076\_g1 c162711\_g2 c162711\_g1 c168415\_g1 | egu:105050579 egu:105038935 egu:105045020 egu:105058570 egu:105053664 egu:105060553 egu:105052957 egu:105048742 egu:105056954 egu:105058818 egu:105045690 egu:105055498 egu:105056954 egu:105053817 egu:105047594 egu:105045761 egu:105045262 egu:105058479 egu:105048592 egu:105061041 egu:105048227 | 105050579 105038935 105045020 105058570 105053664 105060553 105052957 105048742 105056954 105058818 105045690 105055498 105056954 105053817 105047594 105045761 105045262 105058479 105048592 105061041 105048227 |  |
| Flavone and flavonol biosynthesis | 1 | 10 | 0.64391415432 | 0.999951598671 | c154616\_g1 | egu:105058071 | 105058071 |  |
| Butanoate metabolism | 2 | 22 | 0.646712580566 | 0.999951598671 | c151510\_g1 c146169\_g1 | egu:105041902 egu:105034095 | 105041902 105034095 |  |
| Carbon fixation in photosynthetic organisms | 8 | 89 | 0.649044153747 | 0.999951598671 | c159520\_g1 c105074\_g2 c145285\_g1 c163723\_g1 c157432\_g1 c166833\_g1 c170991\_g2 c145522\_g1 | egu:105047057 egu:105051363 egu:105059882 egu:105058702 egu:105059872 egu:105043985 egu:105059450 egu:105058543 | 105047057 105051363 105059882 105058702 105059872 105043985 105059450 105058543 |  |
| Glycosphingolipid biosynthesis - globo series | 1 | 11 | 0.67583403804 | 0.999951598671 | c172397\_g1 | egu:105036046 | 105036046 |  |
| alpha-Linolenic acid metabolism | 5 | 58 | 0.676424960206 | 0.999951598671 | c153824\_g1 c173762\_g1 c172946\_g1 c166548\_g2 c162165\_g1 | egu:105041633 egu:105053112 egu:105038559 egu:105039328 egu:105056718 | 105041633 105053112 105038559 105039328 105056718 |  |
| Nucleotide excision repair | 6 | 70 | 0.686125192601 | 0.999951598671 | c167546\_g1 c172314\_g3 c169225\_g4 c139006\_g1 c168792\_g1 c160857\_g1 | egu:105057784 egu:105051054 egu:105036598 egu:105040945 egu:105044402 egu:105046296 | 105057784 105051054 105036598 105040945 105044402 105046296 |  |
| Biosynthesis of unsaturated fatty acids | 3 | 36 | 0.691252162799 | 0.999951598671 | c168511\_g1 c153824\_g1 c162055\_g1 | egu:105052984 egu:105041633 egu:105045727 | 105052984 105041633 105045727 |  |
| Valine, leucine and isoleucine degradation | 4 | 48 | 0.696513447569 | 0.999951598671 | c148031\_g1 c159016\_g1 c155751\_g1 c146169\_g1 | egu:105042090 egu:105045148 egu:105058186 egu:105034095 | 105042090 105045148 105058186 105034095 |  |
| Phosphatidylinositol signaling system | 6 | 71 | 0.698344442946 | 0.999951598671 | c101051\_g1 c166855\_g1 c152208\_g2 c104329\_g1 c162261\_g1 c135007\_g1 | egu:105057333 egu:105057333 egu:105056668 egu:105055908 egu:105048729 egu:105036034 | 105057333 105057333 105056668 105055908 105048729 105036034 |  |
| Fatty acid metabolism | 7 | 85 | 0.728030620942 | 0.999951598671 | c161647\_g2 c153824\_g1 c167962\_g1 c159016\_g1 c162055\_g1 c165048\_g1 c168511\_g1 | egu:105047881 egu:105041633 egu:105056688 egu:105045148 egu:105045727 egu:105041644 egu:105052984 | 105047881 105041633 105056688 105045148 105045727 105041644 105052984 |  |
| Endocytosis | 17 | 197 | 0.733894400066 | 0.999951598671 | c147063\_g1 c173338\_g6 c133817\_g1 c165623\_g2 c171004\_g1 c167245\_g1 c173571\_g2 c105465\_g1 c141698\_g1 c163181\_g5 c142610\_g1 c165298\_g1 c142610\_g2 c141698\_g2 c162261\_g1 c161639\_g1 c162333\_g2 | egu:105052346 egu:105058367 egu:105045139 egu:105033970 egu:105038113 egu:105054164 egu:105039433 egu:105049593 egu:105056954 egu:105045690 egu:105044044 egu:105032610 egu:105044044 egu:105056954 egu:105048729 egu:105045676 egu:105043229 | 105052346 105058367 105045139 105033970 105038113 105054164 105039433 105049593 105056954 105045690 105044044 105032610 105044044 105056954 105048729 105045676 105043229 |  |
| Metabolic pathways | 204 | 2161 | 0.744726245379 | 0.999951598671 | c170590\_g5 c174522\_g5 c158181\_g1 c171895\_g1 c146169\_g1 c162039\_g1 c168403\_g1 c173509\_g1 c167554\_g2 c173447\_g1 c172045\_g1 c162165\_g1 c170577\_g2 c170991\_g2 c166080\_g1 c166861\_g1 c121963\_g1 c158824\_g1 c169830\_g1 c167962\_g1 c173502\_g2 c158038\_g1 c167649\_g1 c166229\_g1 c170590\_g8 c158107\_g1 c169649\_g1 c168674\_g1 c140061\_g1 c167553\_g1 c155351\_g1 c161228\_g1 c133817\_g1 c165048\_g1 c148234\_g1 c169214\_g1 c166548\_g2 c163051\_g1 c162048\_g1 c114481\_g1 c172086\_g3 c145522\_g1 c161647\_g2 c155351\_g2 c134164\_g1 c167137\_g1 c134944\_g1 c151318\_g1 c156756\_g2 c172256\_g4 c165623\_g2 c157258\_g1 c170429\_g1 c166197\_g1 c164800\_g1 c169857\_g1 c167493\_g1 c161281\_g1 c159520\_g1 c169469\_g1 c172938\_g7 c129527\_g1 c168117\_g1 c158409\_g1 c161851\_g3 c159060\_g1 c119816\_g1 c163397\_g1 c107073\_g1 c165885\_g1 c168575\_g1 c168511\_g1 c166833\_g1 c156970\_g1 c156718\_g1 c168772\_g1 c174721\_g1 c163681\_g1 c105074\_g2 c161779\_g15 c160696\_g1 c162772\_g1 c154095\_g1 c152491\_g2 c152491\_g1 c173813\_g1 c149471\_g1 c170819\_g2 c141199\_g1 c133447\_g2 c117443\_g1 c148031\_g1 c157266\_g1 c172129\_g1 c156235\_g1 c103295\_g1 c121798\_g1 c162887\_g1 c174249\_g4 c174513\_g3 c174513\_g1 c175256\_g1 c172397\_g1 c140986\_g1 c168304\_g3 c168304\_g2 c168304\_g1 c162417\_g1 c166123\_g1 c173762\_g1 c152294\_g1 c163448\_g1 c167006\_g1 c134078\_g1 c165806\_g1 c162261\_g1 c164898\_g1 c154616\_g1 c170127\_g1 c157509\_g1 c140940\_g1 c171913\_g1 c146896\_g1 c146896\_g3 c170271\_g1 c152936\_g1 c151895\_g1 c163671\_g1 c155053\_g1 c163736\_g1 c104905\_g1 c151510\_g1 c170749\_g4 c170749\_g3 c164458\_g2 c167245\_g1 c171769\_g1 c151855\_g2 c94583\_g1 c173425\_g1 c155636\_g1 c164911\_g1 c155636\_g2 c161769\_g1 c172556\_g1 c153824\_g1 c169422\_g1 c79219\_g1 c158821\_g1 c172816\_g4 c135926\_g1 c155146\_g1 c149166\_g1 c165538\_g1 c135007\_g1 c153848\_g1 c158910\_g1 c134112\_g1 c156756\_g1 c171054\_g2 c163723\_g1 c164239\_g2 c104329\_g1 c146719\_g1 c141054\_g1 c166729\_g1 c150645\_g1 c151401\_g1 c150889\_g1 c158673\_g1 c171631\_g6 c157181\_g1 c173495\_g2 c159016\_g1 c145285\_g1 c172946\_g1 c116327\_g1 c161872\_g2 c151470\_g3 c151470\_g2 c166556\_g1 c155459\_g1 c172074\_g1 c151973\_g1 c155751\_g1 c164458\_g1 c188298\_g1 c164009\_g2 c168951\_g1 c170056\_g1 c163639\_g1 c161991\_g1 c168406\_g1 c168307\_g1 c156393\_g1 c156393\_g2 c170886\_g3 c133326\_g1 c137525\_g1 c122873\_g1 c157432\_g1 c133035\_g1 c165035\_g1 c159549\_g1 | egu:105039431 egu:12079509 egu:105039124 egu:105060985 egu:105034095 egu:105059487 egu:105036591 egu:105035937 egu:105055983 egu:105047312 egu:105049657 egu:105056718 egu:105035222 egu:105059450 egu:105055673 egu:105060488 egu:105035499 egu:105054034 egu:105051975 egu:105056688 egu:105051431 egu:105059341 egu:105059739 egu:105047342 egu:105039431 egu:105048348 egu:105058113 egu:105044445 egu:105032793 egu:105035984 egu:105032793 egu:105047312 egu:105045139 egu:105041644 egu:105054501 egu:105045810 egu:105039328 egu:105053561 egu:105046802 egu:105053391 egu:105032431 egu:105058543 egu:105047881 egu:105032793 egu:105053813 egu:105055420 egu:105049537 egu:12079419 egu:105058982 egu:105042391 egu:105033970 egu:105039187 egu:105039157 egu:105047182 egu:105046237 egu:105035716 egu:105060774 egu:105059274 egu:105047057 egu:105045186 egu:105038037 egu:105036609 egu:105046802 egu:105050719 egu:105042120 egu:105043204 egu:105038209 egu:105060532 egu:105039834 egu:105041687 egu:105041372 egu:105052984 egu:105043985 egu:105055939 egu:105056873 egu:105037452 egu:105043800 egu:105060274 egu:105051363 egu:105037657 egu:105046158 egu:105060382 egu:105034598 egu:105061179 egu:105061179 egu:105055950 egu:105040297 egu:105044276 egu:105040573 egu:105032435 egu:105052990 egu:105042090 egu:105056682 egu:105048962 egu:105057764 egu:105048637 egu:105034997 egu:105039619 egu:12079446 egu:105050962 egu:105050962 egu:105052340 egu:105036046 egu:105056224 egu:105055673 egu:105055673 egu:105035781 egu:105047825 egu:105058884 egu:105053112 egu:105055883 egu:105055201 egu:105044125 egu:105051251 egu:105045995 egu:105048729 egu:105049059 egu:105058071 egu:105041133 egu:105050758 egu:105035694 egu:105034870 egu:105050962 egu:105050962 egu:105044125 egu:105036609 egu:105060687 egu:105035203 egu:105035842 egu:105042572 egu:105053391 egu:105041902 egu:105045199 egu:105045199 egu:105042574 egu:105054164 egu:12079391 egu:105053370 egu:105046530 egu:105053256 egu:105042391 egu:105055260 egu:105042391 egu:105038179 egu:105045855 egu:105041633 egu:105035608 egu:105043204 egu:105053882 egu:105060220 egu:105047511 egu:105036364 egu:105060729 egu:105049657 egu:105036034 egu:105033631 egu:105040617 egu:105040155 egu:105042489 egu:105050243 egu:105058702 egu:105060182 egu:105055908 egu:105058232 egu:105033487 egu:105054663 egu:105053413 egu:105050962 egu:105048800 egu:105059570 egu:105034397 egu:105046041 egu:105060892 egu:105045148 egu:105059882 egu:105038559 egu:105038419 egu:12079488 egu:105046041 egu:105052340 egu:105058580 egu:105059350 egu:105057280 egu:105038882 egu:105058186 egu:105042574 egu:105035292 egu:105049423 egu:105055420 egu:105037657 egu:105039255 egu:105047536 egu:105037948 egu:105038499 egu:105039053 egu:105050124 egu:105050474 egu:105035371 egu:105052944 egu:105055983 egu:105059872 egu:105034012 egu:105050729 egu:105044026 | 105039431 12079509 105039124 105060985 105034095 105059487 105036591 105035937 105055983 105047312 105049657 105056718 105035222 105059450 105055673 105060488 105035499 105054034 105051975 105056688 105051431 105059341 105059739 105047342 105039431 105048348 105058113 105044445 105032793 105035984 105032793 105047312 105045139 105041644 105054501 105045810 105039328 105053561 105046802 105053391 105032431 105058543 105047881 105032793 105053813 105055420 105049537 12079419 105058982 105042391 105033970 105039187 105039157 105047182 105046237 105035716 105060774 105059274 105047057 105045186 105038037 105036609 105046802 105050719 105042120 105043204 105038209 105060532 105039834 105041687 105041372 105052984 105043985 105055939 105056873 105037452 105043800 105060274 105051363 105037657 105046158 105060382 105034598 105061179 105061179 105055950 105040297 105044276 105040573 105032435 105052990 105042090 105056682 105048962 105057764 105048637 105034997 105039619 12079446 105050962 105050962 105052340 105036046 105056224 105055673 105055673 105035781 105047825 105058884 105053112 105055883 105055201 105044125 105051251 105045995 105048729 105049059 105058071 105041133 105050758 105035694 105034870 105050962 105050962 105044125 105036609 105060687 105035203 105035842 105042572 105053391 105041902 105045199 105045199 105042574 105054164 12079391 105053370 105046530 105053256 105042391 105055260 105042391 105038179 105045855 105041633 105035608 105043204 105053882 105060220 105047511 105036364 105060729 105049657 105036034 105033631 105040617 105040155 105042489 105050243 105058702 105060182 105055908 105058232 105033487 105054663 105053413 105050962 105048800 105059570 105034397 105046041 105060892 105045148 105059882 105038559 105038419 12079488 105046041 105052340 105058580 105059350 105057280 105038882 105058186 105042574 105035292 105049423 105055420 105037657 105039255 105047536 105037948 105038499 105039053 105050124 105050474 105035371 105052944 105055983 105059872 105034012 105050729 105044026 |  |
| Pentose and glucuronate interconversions | 7 | 87 | 0.748429869786 | 0.999951598671 | c166556\_g1 c167554\_g2 c172026\_g5 c158038\_g1 c122873\_g1 c164911\_g1 c149471\_g1 | egu:105058580 egu:105055983 egu:105046157 egu:105059341 egu:105055983 egu:105055260 egu:105040297 | 105058580 105055983 105046157 105059341 105055983 105055260 105040297 |  |
| Lysine biosynthesis | 1 | 15 | 0.777369015245 | 0.999951598671 | c148031\_g1 | egu:105042090 | 105042090 |  |
| Diterpenoid biosynthesis | 2 | 29 | 0.779487798587 | 0.999951598671 | c167196\_g2 c162655\_g1 | egu:105043827 egu:105034258 | 105043827 105034258 |  |
| ABC transporters | 2 | 29 | 0.779487798587 | 0.999951598671 | c76811\_g1 c173470\_g3 | egu:105032304 egu:105060172 | 105032304 105060172 |  |
| mRNA surveillance pathway | 12 | 149 | 0.787697977815 | 0.999951598671 | c139798\_g1 c154451\_g2 c172184\_g1 c172927\_g1 c146246\_g2 c169759\_g1 c167848\_g1 c143034\_g2 c163585\_g1 c174709\_g3 c52599\_g1 c141841\_g1 | egu:105040379 egu:105049603 egu:105042580 egu:105050127 egu:105032926 egu:105031982 egu:105034445 egu:105057634 egu:105041936 egu:105034835 egu:105039096 egu:105060065 | 105040379 105049603 105042580 105050127 105032926 105031982 105034445 105057634 105041936 105034835 105039096 105060065 |  |
| Base excision repair | 3 | 44 | 0.805019751658 | 0.999951598671 | c173164\_g1 c167546\_g1 c160857\_g1 | egu:105051450 egu:105057784 egu:105046296 | 105051450 105057784 105046296 |  |
| Plant-pathogen interaction | 18 | 220 | 0.807228204988 | 0.999951598671 | c113196\_g1 c158259\_g1 c147877\_g2 c101051\_g1 c166423\_g2 c163371\_g2 c166855\_g1 c174710\_g5 c158416\_g1 c154003\_g1 c156536\_g2 c152058\_g1 c166605\_g3 c172657\_g2 c161556\_g2 c147918\_g2 c158996\_g1 c153491\_g1 | egu:105057654 egu:105046864 egu:105050781 egu:105057333 egu:105060351 egu:105052035 egu:105057333 egu:105050612 egu:105057222 egu:105048941 egu:105046031 egu:105034665 egu:105044758 egu:105054741 egu:105047530 egu:105052456 egu:105047519 egu:105051034 | 105057654 105046864 105050781 105057333 105060351 105052035 105057333 105050612 105057222 105048941 105046031 105034665 105044758 105054741 105047530 105052456 105047519 105051034 |  |
| RNA degradation | 10 | 130 | 0.815460682956 | 0.999951598671 | c173926\_g1 c162676\_g1 c156321\_g2 c167493\_g1 c163051\_g1 c156792\_g1 c169556\_g1 c167106\_g2 c152859\_g1 c165391\_g1 | egu:105035524 egu:105044893 egu:105046443 egu:105060774 egu:105053561 egu:105033956 egu:105058802 egu:105034266 egu:105055716 egu:105044967 | 105035524 105044893 105046443 105060774 105053561 105033956 105058802 105034266 105055716 105044967 |  |
| Histidine metabolism | 1 | 17 | 0.815509021109 | 0.999951598671 | c148031\_g1 | egu:105042090 | 105042090 |  |
| Steroid biosynthesis | 3 | 45 | 0.816413639248 | 0.999951598671 | c151855\_g2 c152294\_g1 c163671\_g1 | egu:105053370 egu:105055883 egu:105035203 | 105053370 105055883 105035203 |  |
| Citrate cycle (TCA cycle) | 5 | 71 | 0.822342181498 | 0.999951598671 | c170886\_g3 c117443\_g1 c149166\_g1 c162039\_g1 c145522\_g1 | egu:105050474 egu:105052990 egu:105060729 egu:105059487 egu:105058543 | 105050474 105052990 105060729 105059487 105058543 |  |
| Inositol phosphate metabolism | 5 | 72 | 0.830953135564 | 0.999951598671 | c104329\_g1 c162261\_g1 c135007\_g1 c152208\_g2 c164800\_g1 | egu:105055908 egu:105048729 egu:105036034 egu:105056668 egu:105046237 | 105055908 105048729 105036034 105056668 105046237 |  |
| Glycosaminoglycan degradation | 1 | 18 | 0.83205580201 | 0.999951598671 | c172397\_g1 | egu:105036046 | 105036046 |  |
| Terpenoid backbone biosynthesis | 4 | 61 | 0.84552702579 | 0.999951598671 | c161281\_g1 c95088\_g1 c159165\_g1 c146169\_g1 | egu:105059274 egu:105036025 egu:105039667 egu:105034095 | 105059274 105036025 105039667 105034095 |  |
| Fatty acid degradation | 3 | 48 | 0.847249183446 | 0.999951598671 | c148031\_g1 c153824\_g1 c159016\_g1 | egu:105042090 egu:105041633 egu:105045148 | 105042090 105041633 105045148 |  |
| Propanoate metabolism | 2 | 35 | 0.856263994986 | 0.999951598671 | c165048\_g1 c159016\_g1 | egu:105041644 egu:105045148 | 105041644 105045148 |  |
| Brassinosteroid biosynthesis | 1 | 20 | 0.860833420677 | 0.999951598671 | c156970\_g1 | egu:105055939 | 105055939 |  |
| Stilbenoid, diarylheptanoid and gingerol biosynthesis | 1 | 20 | 0.860833420677 | 0.999951598671 | c165806\_g1 | egu:105045995 | 105045995 |  |
| Porphyrin and chlorophyll metabolism | 3 | 50 | 0.865214911433 | 0.999951598671 | c151973\_g1 c104905\_g1 c114481\_g1 | egu:105038882 egu:105053391 egu:105053391 | 105038882 105053391 105053391 |  |
| Glycerolipid metabolism | 5 | 77 | 0.869027463718 | 0.999951598671 | c168575\_g1 c148031\_g1 c140940\_g1 c160696\_g1 c164009\_g2 | egu:105041372 egu:105042090 egu:105035694 egu:105046158 egu:105049423 | 105041372 105042090 105035694 105046158 105049423 |  |
| Fructose and mannose metabolism | 6 | 90 | 0.870545158347 | 0.999951598671 | c170590\_g5 c167493\_g1 c170590\_g8 c158038\_g1 c133326\_g1 c169226\_g1 | egu:105039431 egu:105060774 egu:105039431 egu:105059341 egu:105035371 egu:105040553 | 105039431 105060774 105039431 105059341 105035371 105040553 |  |
| Other glycan degradation | 1 | 21 | 0.873317965439 | 0.999951598671 | c172397\_g1 | egu:105036046 | 105036046 |  |
| SNARE interactions in vesicular transport | 3 | 52 | 0.881293243313 | 0.999951598671 | c155629\_g1 c162457\_g1 c167775\_g2 | egu:105047890 egu:105059832 egu:105059832 | 105047890 105059832 105059832 |  |
| Folate biosynthesis | 1 | 22 | 0.884683396675 | 0.999951598671 | c158107\_g1 | egu:105048348 | 105048348 |  |
| Pyrimidine metabolism | 9 | 134 | 0.903192437859 | 0.999951598671 | c158673\_g1 c174522\_g5 c171631\_g6 c161851\_g3 c171895\_g1 c107073\_g1 c169556\_g1 c166229\_g1 c172086\_g3 | egu:105059570 egu:12079509 egu:105034397 egu:105042120 egu:105060985 egu:105039834 egu:105058802 egu:105047342 egu:105032431 | 105059570 12079509 105034397 105042120 105060985 105039834 105058802 105047342 105032431 |  |
| Alanine, aspartate and glutamate metabolism | 3 | 57 | 0.914248714041 | 0.999951598671 | c151510\_g1 c168403\_g1 c157258\_g1 | egu:105041902 egu:105036591 egu:105039187 | 105041902 105036591 105039187 |  |
| Tryptophan metabolism | 2 | 42 | 0.914545682997 | 0.999951598671 | c148031\_g1 c168772\_g1 | egu:105042090 egu:105037452 | 105042090 105037452 |  |
| Fatty acid biosynthesis | 3 | 59 | 0.924920262986 | 0.999951598671 | c165048\_g1 c167962\_g1 c161647\_g2 | egu:105041644 egu:105056688 egu:105047881 | 105041644 105056688 105047881 |  |
| Cysteine and methionine metabolism | 7 | 114 | 0.925094593236 | 0.999951598671 | c140061\_g1 c155351\_g1 c155351\_g2 c157509\_g1 c155751\_g1 c173813\_g1 c145522\_g1 | egu:105032793 egu:105032793 egu:105032793 egu:105050758 egu:105058186 egu:105055950 egu:105058543 | 105032793 105032793 105032793 105050758 105058186 105055950 105058543 |  |
| 2-Oxocarboxylic acid metabolism | 3 | 60 | 0.929786703484 | 0.999951598671 | c170886\_g3 c155751\_g1 c156235\_g1 | egu:105050474 egu:105058186 egu:105057764 | 105050474 105058186 105057764 |  |
| Protein export | 3 | 60 | 0.929786703484 | 0.999951598671 | c172536\_g1 c138913\_g1 c166038\_g3 | egu:105035090 egu:105053092 egu:105055078 | 105035090 105053092 105055078 |  |
| Pantothenate and CoA biosynthesis | 1 | 32 | 0.954972248414 | 0.999951598671 | c155751\_g1 | egu:105058186 | 105058186 |  |
| Phagosome | 6 | 118 | 0.97103712241 | 0.999951598671 | c157053\_g1 c149749\_g2 c169214\_g1 c160425\_g1 c152208\_g2 c163575\_g2 | egu:105042885 egu:105054846 egu:105045810 egu:105050980 egu:105056668 egu:105059030 | 105042885 105054846 105045810 105050980 105056668 105059030 |  |
| Proteasome | 3 | 75 | 0.975287405611 | 0.999951598671 | c168479\_g2 c156463\_g2 c198607\_g1 | egu:105052320 egu:105050364 egu:105041809 | 105052320 105050364 105041809 |  |
| Photosynthesis | 3 | 82 | 0.985136175168 | 0.999951598671 | c171769\_g1 c133035\_g1 c151318\_g1 | egu:12079391 egu:105034012 egu:12079419 | 12079391 105034012 12079419 |  |
| Glyoxylate and dicarboxylate metabolism | 3 | 83 | 0.986190209651 | 0.999951598671 | c170886\_g3 c116327\_g1 c145522\_g1 | egu:105050474 egu:105038419 egu:105058543 | 105050474 105038419 105058543 |  |
| Protein processing in endoplasmic reticulum | 15 | 257 | 0.987436885841 | 0.999951598671 | c141698\_g2 c171451\_g1 c171446\_g2 c147952\_g2 c159312\_g1 c143179\_g1 c141977\_g1 c163181\_g5 c153315\_g2 c160425\_g1 c141698\_g1 c94583\_g1 c161184\_g2 c159142\_g2 c172657\_g2 | egu:105056954 egu:105051770 egu:105038626 egu:105060095 egu:105053689 egu:105041025 egu:105042707 egu:105045690 egu:105038626 egu:105050980 egu:105056954 egu:105046530 egu:105052833 egu:105057396 egu:105054741 | 105056954 105051770 105038626 105060095 105053689 105041025 105042707 105045690 105038626 105050980 105056954 105046530 105052833 105057396 105054741 |  |
| Basal transcription factors | 1 | 54 | 0.994326870784 | 0.999951598671 | c164781\_g1 | egu:105055048 | 105055048 |  |
| Oxidative phosphorylation | 7 | 163 | 0.995226964315 | 0.999951598671 | c117443\_g1 c140802\_g1 c169214\_g1 c174249\_g4 c154310\_g1 c149166\_g1 c161872\_g2 | egu:105052990 egu:105052009 egu:105045810 egu:12079446 egu:105044465 egu:105060729 egu:12079488 | 105052990 105052009 105045810 12079446 105044465 105060729 12079488 |  |
| Peroxisome | 3 | 113 | 0.998604893396 | 0.999951598671 | c152362\_g1 c160216\_g1 c153824\_g1 | egu:105058041 egu:105061386 egu:105041633 | 105058041 105061386 105041633 |  |
| Ubiquitin mediated proteolysis | 4 | 159 | 0.999824924437 | 0.999951598671 | c168179\_g1 c164685\_g2 c169528\_g2 c114328\_g1 | egu:105044042 egu:105045873 egu:105043171 egu:105045214 | 105044042 105045873 105043171 105045214 |  |
| Ribosome | 16 | 377 | 0.999951598671 | 0.999951598671 | c148511\_g1 c158891\_g1 c115398\_g1 c146787\_g1 c159076\_g1 c165977\_g1 c133240\_g1 c156834\_g1 c224168\_g1 c145296\_g1 c142526\_g1 c131691\_g1 c13257\_g1 c138227\_g1 c157907\_g1 c157164\_g3 | egu:105058445 egu:105048878 egu:105056627 egu:105044506 egu:105059802 egu:105044285 egu:105037979 egu:105042949 egu:105033541 egu:105049840 egu:105059181 egu:105032842 egu:105051463 egu:105055776 egu:105033079 egu:105041276 | 105058445 105048878 105056627 105044506 105059802 105044285 105037979 105042949 105033541 105049840 105059181 105032842 105051463 105055776 105033079 105041276 |  |
