# Supplementary material for: Physiological and transcriptomic responses of Lanzhou Lily (Lilium davidii, var. unicolor) to cold stress
Source: PLoS One. 2020 Jan 23;15(1):e0227921. doi: 10.1371/journal.pone.0227921 (PMC6977731; doi:10.1371/journal.pone.0227921)
Supplement: S1 Zip — (Zip). CK: control (20°C); LT: low temperature (4°C). (ZIP) [file pone.0227921.s011.zip › S1 Zip/src/egu00010.html]

egu00010


- egu:105042090

- Up regulated genes

c148031\_g1(0.6165)

- egu:105045855

- Up regulated genes

c172556\_g1(1.8748)

- egu:105045855

- Up regulated genes

c172556\_g1(1.8748)

- egu:105059487

- Up regulated genes

c162039\_g1(0.48153)

- egu:105058982

- Up regulated genes

c156756\_g2(2.2972)
- egu:105035292

- Up regulated genes

c188298\_g1(2.0535)
- egu:105038179

- Up regulated genes

c161769\_g1(0.81464)
- egu:105057280

- Up regulated genes

c172074\_g1(2.793)
- egu:105053882

- Up regulated genes

c158821\_g1(0.52215)
- egu:105042489

- Up regulated genes

c156756\_g1(2.052)

- egu:105053561

- Up regulated genes

c163051\_g1(1.3984)

- egu:105056873

- Up regulated genes

c156718\_g1(0.83881)

- egu:105059882

- Up regulated genes

c145285\_g1(2.0457)
- egu:105051363

- Up regulated genes

c105074\_g2(1.0862)

- egu:105060774

- Up regulated genes

c167493\_g1(1.2221)

- egu:105059872

- Up regulated genes

c157432\_g1(0.46067)

- egu:105050719

- Up regulated genes

c158409\_g1(0.65101)

- egu:105038209

- Up regulated genes

c119816\_g1(1.47)
- egu:105052340

- Up regulated genes

c175256\_g1(1.4216) c151470\_g2(1.2691)
- egu:105046041

- Up regulated genes

c157181\_g1(1.4428) c151470\_g3(1.9388)

- egu:105039431

- Up regulated genes

c170590\_g5(2.0592) c170590\_g8(1.9734)

Close
