# Supplementary material for: Physiological and transcriptomic responses of Lanzhou Lily (Lilium davidii, var. unicolor) to cold stress
Source: PLoS One. 2020 Jan 23;15(1):e0227921. doi: 10.1371/journal.pone.0227921 (PMC6977731; doi:10.1371/journal.pone.0227921)
Supplement: S1 Zip — (Zip). CK: control (20°C); LT: low temperature (4°C). (ZIP) [file pone.0227921.s011.zip › S1 Zip/src/egu00020.html]

egu00020


- egu:105050474

- Up regulated genes

c170886\_g3(0.98865)

- egu:105058543

- Up regulated genes

c145522\_g1(1.236)

- egu:105059487

- Up regulated genes

c162039\_g1(0.48153)

- egu:105060729

- Up regulated genes

c149166\_g1(1.6337)
- egu:105052990

- Up regulated genes

c117443\_g1(0.9188)

Close
