# Supplementary material for: Physiological and transcriptomic responses of Lanzhou Lily (Lilium davidii, var. unicolor) to cold stress
Source: PLoS One. 2020 Jan 23;15(1):e0227921. doi: 10.1371/journal.pone.0227921 (PMC6977731; doi:10.1371/journal.pone.0227921)
Supplement: S1 Zip — (Zip). CK: control (20°C); LT: low temperature (4°C). (ZIP) [file pone.0227921.s011.zip › S1 Zip/src/egu00030.html]

egu00030


- egu:105059450

- Up regulated genes

c170991\_g2(0.56857)

- egu:105047057

- Up regulated genes

c159520\_g1(1.375)

- egu:105059450

- Up regulated genes

c170991\_g2(0.56857)

- egu:105060774

- Up regulated genes

c167493\_g1(1.2221)

- egu:105032435

- Up regulated genes

c133447\_g2(0.58858)

- egu:105059350

- Up regulated genes

c155459\_g1(0.63442)

- egu:105060382

- Up regulated genes

c162772\_g1(0.74368)

- egu:105050719

- Up regulated genes

c158409\_g1(0.65101)

- egu:105032435

- Up regulated genes

c133447\_g2(0.58858)

- egu:105039431

- Up regulated genes

c170590\_g5(2.0592) c170590\_g8(1.9734)

Close
