# Supplementary material for: Physiological and transcriptomic responses of Lanzhou Lily (Lilium davidii, var. unicolor) to cold stress
Source: PLoS One. 2020 Jan 23;15(1):e0227921. doi: 10.1371/journal.pone.0227921 (PMC6977731; doi:10.1371/journal.pone.0227921)
Supplement: S1 Zip — (Zip). CK: control (20°C); LT: low temperature (4°C). (ZIP) [file pone.0227921.s011.zip › S1 Zip/src/egu00040.html]

egu00040


- egu:105055260

- Up regulated genes

c164911\_g1(0.86235)

- egu:105055983

- Up regulated genes

c167554\_g2(0.95472) c122873\_g1(1.2297)

- egu:105058580

- Up regulated genes

c166556\_g1(0.76938)
- egu:105040297

- Up regulated genes

c149471\_g1(1.7421)

- egu:105046157

- Up regulated genes

c172026\_g5(Inf)

- egu:105059341

- Up regulated genes

c158038\_g1(0.5281)

Close
