# Supplementary material for: Physiological and transcriptomic responses of Lanzhou Lily (Lilium davidii, var. unicolor) to cold stress
Source: PLoS One. 2020 Jan 23;15(1):e0227921. doi: 10.1371/journal.pone.0227921 (PMC6977731; doi:10.1371/journal.pone.0227921)
Supplement: S1 Zip — (Zip). CK: control (20°C); LT: low temperature (4°C). (ZIP) [file pone.0227921.s011.zip › S1 Zip/src/egu00051.html]

egu00051


- egu:105059341

- Up regulated genes

c158038\_g1(0.5281)

- egu:105040553

- Up regulated genes

c169226\_g1(0.65798)

- egu:105035371

- Up regulated genes

c133326\_g1(1.0801)

- egu:105060774

- Up regulated genes

c167493\_g1(1.2221)

- egu:105039431

- Up regulated genes

c170590\_g5(2.0592) c170590\_g8(1.9734)

Close
