# Supplementary material for: Physiological and transcriptomic responses of Lanzhou Lily (Lilium davidii, var. unicolor) to cold stress
Source: PLoS One. 2020 Jan 23;15(1):e0227921. doi: 10.1371/journal.pone.0227921 (PMC6977731; doi:10.1371/journal.pone.0227921)
Supplement: S1 Zip — (Zip). CK: control (20°C); LT: low temperature (4°C). (ZIP) [file pone.0227921.s011.zip › S1 Zip/src/egu00052.html]

egu00052


- egu:105060774

- Up regulated genes

c167493\_g1(1.2221)

- egu:105054034

- Up regulated genes

c158824\_g1(0.69999)

- egu:105054034

- Up regulated genes

c158824\_g1(0.69999)

- egu:105042572

- Up regulated genes

c163736\_g1(1.7307)

- egu:105055260

- Up regulated genes

c164911\_g1(0.86235)

- egu:105034931

- Up regulated genes

c171613\_g1(1.6356)
- egu:105038174

- Up regulated genes

c147292\_g1(1.1081)
- egu:105056490

- Up regulated genes

c164267\_g1(1.3783)

- egu:105057305

- Up regulated genes

c167034\_g1(5.0343)

Close
