# Supplementary material for: Physiological and transcriptomic responses of Lanzhou Lily (Lilium davidii, var. unicolor) to cold stress
Source: PLoS One. 2020 Jan 23;15(1):e0227921. doi: 10.1371/journal.pone.0227921 (PMC6977731; doi:10.1371/journal.pone.0227921)
Supplement: S1 Zip — (Zip). CK: control (20°C); LT: low temperature (4°C). (ZIP) [file pone.0227921.s011.zip › S1 Zip/src/egu00053.html]

egu00053


- egu:105036034

- Up regulated genes

c135007\_g1(0.70355)

- egu:105038499

- Up regulated genes

c168307\_g1(4.6883)

- egu:105053413

- Up regulated genes

c150645\_g1(0.4666)

- egu:105053413

- Up regulated genes

c150645\_g1(0.4666)

- egu:105053413

- Up regulated genes

c150645\_g1(0.4666)

- egu:105055983

- Up regulated genes

c167554\_g2(0.95472) c122873\_g1(1.2297)

- egu:105042090

- Up regulated genes

c148031\_g1(0.6165)

Close
