# Supplementary material for: Physiological and transcriptomic responses of Lanzhou Lily (Lilium davidii, var. unicolor) to cold stress
Source: PLoS One. 2020 Jan 23;15(1):e0227921. doi: 10.1371/journal.pone.0227921 (PMC6977731; doi:10.1371/journal.pone.0227921)
Supplement: S1 Zip — (Zip). CK: control (20°C); LT: low temperature (4°C). (ZIP) [file pone.0227921.s011.zip › S1 Zip/src/egu00062.html]

egu00062


- egu:105052984

- Up regulated genes

c168511\_g1(1.3724)

- egu:105045727

- Up regulated genes

c162055\_g1(0.89454)

- egu:105041077

- Up regulated genes

c166224\_g1(0.80628)
- egu:105058603

- Up regulated genes

c159288\_g1(0.94417)
- egu:105042699

- Up regulated genes

c163278\_g1(2.0506)
- egu:105053416

- Up regulated genes

c161171\_g2(2.1207) c161171\_g1(1.6166)

Close
