# Supplementary material for: Physiological and transcriptomic responses of Lanzhou Lily (Lilium davidii, var. unicolor) to cold stress
Source: PLoS One. 2020 Jan 23;15(1):e0227921. doi: 10.1371/journal.pone.0227921 (PMC6977731; doi:10.1371/journal.pone.0227921)
Supplement: S1 Zip — (Zip). CK: control (20°C); LT: low temperature (4°C). (ZIP) [file pone.0227921.s011.zip › S1 Zip/src/egu00071.html]

egu00071


- egu:105045148

- Up regulated genes

c159016\_g1(2.0401)

- egu:105045148

- Up regulated genes

c159016\_g1(2.0401)

- egu:105045148

- Up regulated genes

c159016\_g1(2.0401)

- egu:105045148

- Up regulated genes

c159016\_g1(2.0401)

- egu:105045148

- Up regulated genes

c159016\_g1(2.0401)

- egu:105045148

- Up regulated genes

c159016\_g1(2.0401)

- egu:105045148

- Up regulated genes

c159016\_g1(2.0401)

- egu:105041633

- Up regulated genes

c153824\_g1(0.77094)

- egu:105041633

- Up regulated genes

c153824\_g1(0.77094)

- egu:105041633

- Up regulated genes

c153824\_g1(0.77094)

- egu:105041633

- Up regulated genes

c153824\_g1(0.77094)

- egu:105041633

- Up regulated genes

c153824\_g1(0.77094)

- egu:105041633

- Up regulated genes

c153824\_g1(0.77094)

- egu:105041633

- Up regulated genes

c153824\_g1(0.77094)

- egu:105042090

- Up regulated genes

c148031\_g1(0.6165)

Close
