# Supplementary material for: Physiological and transcriptomic responses of Lanzhou Lily (Lilium davidii, var. unicolor) to cold stress
Source: PLoS One. 2020 Jan 23;15(1):e0227921. doi: 10.1371/journal.pone.0227921 (PMC6977731; doi:10.1371/journal.pone.0227921)
Supplement: S1 Zip — (Zip). CK: control (20°C); LT: low temperature (4°C). (ZIP) [file pone.0227921.s011.zip › S1 Zip/src/egu00130.html]

egu00130


- egu:105040573

- Up regulated genes

c141199\_g1(1.1797)

- egu:105053813

- Up regulated genes

c134164\_g1(5.8972)
- egu:105039619

- Up regulated genes

c162887\_g1(0.93581)

- egu:105052944

- Up regulated genes

c137525\_g1(1.6695)

- egu:105040573

- Up regulated genes

c141199\_g1(1.1797)

- egu:105045995

- Up regulated genes

c165806\_g1(3.5125)

- egu:105052944

- Up regulated genes

c137525\_g1(1.6695)

Close
