# Supplementary material for: Physiological and transcriptomic responses of Lanzhou Lily (Lilium davidii, var. unicolor) to cold stress
Source: PLoS One. 2020 Jan 23;15(1):e0227921. doi: 10.1371/journal.pone.0227921 (PMC6977731; doi:10.1371/journal.pone.0227921)
Supplement: S1 Zip — (Zip). CK: control (20°C); LT: low temperature (4°C). (ZIP) [file pone.0227921.s011.zip › S1 Zip/src/egu00190.html]

egu00190


- egu:12079488

- Up regulated genes

c161872\_g2(1.5346)

- egu:12079446

- Up regulated genes

c174249\_g4(1.8235)

- egu:12079488

- Up regulated genes

c161872\_g2(1.5346)
- egu:12079446

- Up regulated genes

c174249\_g4(1.8235)

- egu:105045810

- Up regulated genes

c169214\_g1(0.84119)

- egu:105044465

- Up regulated genes

c154310\_g1(1.7761)
- egu:105052009

- Up regulated genes

c140802\_g1(1.7675)

- egu:105060729

- Up regulated genes

c149166\_g1(1.6337)

- egu:105052990

- Up regulated genes

c117443\_g1(0.9188)

- egu:105045810

- Up regulated genes

c169214\_g1(0.84119)

- egu:105052990

- Up regulated genes

c117443\_g1(0.9188)
- egu:105060729

- Up regulated genes

c149166\_g1(1.6337)

Close
