# Supplementary material for: Physiological and transcriptomic responses of Lanzhou Lily (Lilium davidii, var. unicolor) to cold stress
Source: PLoS One. 2020 Jan 23;15(1):e0227921. doi: 10.1371/journal.pone.0227921 (PMC6977731; doi:10.1371/journal.pone.0227921)
Supplement: S1 Zip — (Zip). CK: control (20°C); LT: low temperature (4°C). (ZIP) [file pone.0227921.s011.zip › S1 Zip/src/egu00230.html]

egu00230


- egu:105032431

- Up regulated genes

c172086\_g3(2.6529)

- egu:105036591

- Up regulated genes

c168403\_g1(1.1921)

- egu:105058802

- Up regulated genes

c169556\_g1(1.3314)

- egu:105034397

- Up regulated genes

c171631\_g6(1.0224)
- egu:105047342

- Up regulated genes

c166229\_g1(1.1519)
- egu:105060985

- Up regulated genes

c171895\_g1(0.55799)
- egu:105039834

- Up regulated genes

c107073\_g1(0.71258)
- egu:12079509

- Up regulated genes

c174522\_g5(2.3275)

- egu:105032431

- Up regulated genes

c172086\_g3(2.6529)

- egu:105035499

- Up regulated genes

c121963\_g1(0.56446)

- egu:105058802

- Up regulated genes

c169556\_g1(1.3314)

- egu:105034397

- Up regulated genes

c171631\_g6(1.0224)
- egu:105047342

- Up regulated genes

c166229\_g1(1.1519)
- egu:105060985

- Up regulated genes

c171895\_g1(0.55799)
- egu:105039834

- Up regulated genes

c107073\_g1(0.71258)
- egu:12079509

- Up regulated genes

c174522\_g5(2.3275)

- egu:105060687

- Up regulated genes

c151895\_g1(0.8502)

- egu:105035499

- Up regulated genes

c121963\_g1(0.56446)

- egu:105058982

- Up regulated genes

c156756\_g2(2.2972)
- egu:105038179

- Up regulated genes

c161769\_g1(0.81464)
- egu:105053882

- Up regulated genes

c158821\_g1(0.52215)
- egu:105035292

- Up regulated genes

c188298\_g1(2.0535)
- egu:105057280

- Up regulated genes

c172074\_g1(2.793)
- egu:105042489

- Up regulated genes

c156756\_g1(2.052)

- egu:105058982

- Up regulated genes

c156756\_g2(2.2972)
- egu:105038179

- Up regulated genes

c161769\_g1(0.81464)
- egu:105053882

- Up regulated genes

c158821\_g1(0.52215)
- egu:105035292

- Up regulated genes

c188298\_g1(2.0535)
- egu:105057280

- Up regulated genes

c172074\_g1(2.793)
- egu:105042489

- Up regulated genes

c156756\_g1(2.052)

- egu:105058982

- Up regulated genes

c156756\_g2(2.2972)
- egu:105038179

- Up regulated genes

c161769\_g1(0.81464)
- egu:105053882

- Up regulated genes

c158821\_g1(0.52215)
- egu:105035292

- Up regulated genes

c188298\_g1(2.0535)
- egu:105057280

- Up regulated genes

c172074\_g1(2.793)
- egu:105042489

- Up regulated genes

c156756\_g1(2.052)

- egu:105058982

- Up regulated genes

c156756\_g2(2.2972)
- egu:105038179

- Up regulated genes

c161769\_g1(0.81464)
- egu:105053882

- Up regulated genes

c158821\_g1(0.52215)
- egu:105035292

- Up regulated genes

c188298\_g1(2.0535)
- egu:105057280

- Up regulated genes

c172074\_g1(2.793)
- egu:105042489

- Up regulated genes

c156756\_g1(2.052)

Close
