# Supplementary material for: Physiological and transcriptomic responses of Lanzhou Lily (Lilium davidii, var. unicolor) to cold stress
Source: PLoS One. 2020 Jan 23;15(1):e0227921. doi: 10.1371/journal.pone.0227921 (PMC6977731; doi:10.1371/journal.pone.0227921)
Supplement: S1 Zip — (Zip). CK: control (20°C); LT: low temperature (4°C). (ZIP) [file pone.0227921.s011.zip › S1 Zip/src/egu00260.html]

egu00260


- egu:105042090

- Up regulated genes

c148031\_g1(0.6165)

- egu:105049537

- Up regulated genes

c134944\_g1(1.2476)

- egu:105053256

- Up regulated genes

c173425\_g1(0.91322)

- egu:105038419

- Up regulated genes

c116327\_g1(1.1367)

- egu:105033631

- Up regulated genes

c153848\_g1(1.4453)

- egu:105058884

- Up regulated genes

c166123\_g1(0.62807)

- egu:105055420

- Up regulated genes

c167137\_g1(2.6523) c168951\_g1(1.1729)
- egu:105044125

- Up regulated genes

c167006\_g1(0.74568) c170271\_g1(1.7042)

- egu:105033631

- Up regulated genes

c153848\_g1(1.4453)

- egu:105060274

- Up regulated genes

c163681\_g1(0.94659)

- egu:105056873

- Up regulated genes

c156718\_g1(0.83881)

- egu:105038209

- Up regulated genes

c119816\_g1(1.47)
- egu:105052340

- Up regulated genes

c175256\_g1(1.4216) c151470\_g2(1.2691)
- egu:105046041

- Up regulated genes

c157181\_g1(1.4428) c151470\_g3(1.9388)

Close
