# Supplementary material for: Physiological and transcriptomic responses of Lanzhou Lily (Lilium davidii, var. unicolor) to cold stress
Source: PLoS One. 2020 Jan 23;15(1):e0227921. doi: 10.1371/journal.pone.0227921 (PMC6977731; doi:10.1371/journal.pone.0227921)
Supplement: S1 Zip — (Zip). CK: control (20°C); LT: low temperature (4°C). (ZIP) [file pone.0227921.s011.zip › S1 Zip/src/egu00310.html]

egu00310


- egu:105042090

- Up regulated genes

c148031\_g1(0.6165)

- egu:105043130

- Up regulated genes

c165534\_g2(2.7786) c165534\_g1(2.2502)
- egu:105049737

- Up regulated genes

c170170\_g1(1.1388)
- egu:105045520

- Up regulated genes

c173126\_g1(0.85546)
- egu:105053701

- Up regulated genes

c159372\_g1(0.91498)
- egu:105034748

- Up regulated genes

c158212\_g1(1.1718)

- egu:105043130

- Up regulated genes

c165534\_g2(2.7786) c165534\_g1(2.2502)
- egu:105049737

- Up regulated genes

c170170\_g1(1.1388)
- egu:105045520

- Up regulated genes

c173126\_g1(0.85546)
- egu:105053701

- Up regulated genes

c159372\_g1(0.91498)
- egu:105034748

- Up regulated genes

c158212\_g1(1.1718)

- egu:105043130

- Up regulated genes

c165534\_g2(2.7786) c165534\_g1(2.2502)
- egu:105049737

- Up regulated genes

c170170\_g1(1.1388)
- egu:105045520

- Up regulated genes

c173126\_g1(0.85546)
- egu:105053701

- Up regulated genes

c159372\_g1(0.91498)
- egu:105034748

- Up regulated genes

c158212\_g1(1.1718)

- egu:105042090

- Up regulated genes

c148031\_g1(0.6165)

Close
