# Supplementary material for: Physiological and transcriptomic responses of Lanzhou Lily (Lilium davidii, var. unicolor) to cold stress
Source: PLoS One. 2020 Jan 23;15(1):e0227921. doi: 10.1371/journal.pone.0227921 (PMC6977731; doi:10.1371/journal.pone.0227921)
Supplement: S1 Zip — (Zip). CK: control (20°C); LT: low temperature (4°C). (ZIP) [file pone.0227921.s011.zip › S1 Zip/src/egu00330.html]

egu00330


- egu:105042090

- Up regulated genes

c148031\_g1(0.6165)

- egu:105050758

- Up regulated genes

c157509\_g1(2.1923)

- egu:105042090

- Up regulated genes

c148031\_g1(0.6165)

- egu:105035222

- Up regulated genes

c170577\_g2(0.66496)

- egu:105032618

- Up regulated genes

c163847\_g1(2.2781)

- egu:105035697

- Up regulated genes

c173974\_g3(2.0328)
- egu:105058904

- Up regulated genes

c149000\_g1(2.4743)
- egu:105035498

- Up regulated genes

c158241\_g1(1.7429)

- egu:105035697

- Up regulated genes

c173974\_g3(2.0328)
- egu:105058904

- Up regulated genes

c149000\_g1(2.4743)
- egu:105035498

- Up regulated genes

c158241\_g1(1.7429)

- egu:105034598

- Up regulated genes

c154095\_g1(1.2755)

Close
