# Supplementary material for: Physiological and transcriptomic responses of Lanzhou Lily (Lilium davidii, var. unicolor) to cold stress
Source: PLoS One. 2020 Jan 23;15(1):e0227921. doi: 10.1371/journal.pone.0227921 (PMC6977731; doi:10.1371/journal.pone.0227921)
Supplement: S1 Zip — (Zip). CK: control (20°C); LT: low temperature (4°C). (ZIP) [file pone.0227921.s011.zip › S1 Zip/src/egu00360.html]

egu00360


- egu:105035781

- Up regulated genes

c168304\_g1(0.73196)
- egu:105054501

- Up regulated genes

c148234\_g1(5.8458)
- egu:105055673

- Up regulated genes

c168304\_g3(5.2016) c168304\_g2(3.9853) c166080\_g1(4.7318)

- egu:105055420

- Up regulated genes

c167137\_g1(2.6523) c168951\_g1(1.1729)
- egu:105044125

- Up regulated genes

c167006\_g1(0.74568) c170271\_g1(1.7042)

- egu:105037948

- Up regulated genes

c168406\_g1(0.53391)

- egu:105045995

- Up regulated genes

c165806\_g1(3.5125)

- egu:105053813

- Up regulated genes

c134164\_g1(5.8972)
- egu:105039619

- Up regulated genes

c162887\_g1(0.93581)

Close
