# Supplementary material for: Physiological and transcriptomic responses of Lanzhou Lily (Lilium davidii, var. unicolor) to cold stress
Source: PLoS One. 2020 Jan 23;15(1):e0227921. doi: 10.1371/journal.pone.0227921 (PMC6977731; doi:10.1371/journal.pone.0227921)
Supplement: S1 Zip — (Zip). CK: control (20°C); LT: low temperature (4°C). (ZIP) [file pone.0227921.s011.zip › S1 Zip/src/egu00400.html]

egu00400


- egu:105048637

- Up regulated genes

c103295\_g1(1.3228)

- egu:105044276

- Up regulated genes

c170819\_g2(1.17)

- egu:105060274

- Up regulated genes

c163681\_g1(0.94659)

- egu:105060274

- Up regulated genes

c163681\_g1(0.94659)

- egu:105060182

- Up regulated genes

c164239\_g2(1.4555)

- egu:105040155

- Up regulated genes

c134112\_g1(0.98133)
- egu:105036609

- Up regulated genes

c129527\_g1(1.6156) c152936\_g1(2.5657)

- egu:105040155

- Up regulated genes

c134112\_g1(0.98133)
- egu:105036609

- Up regulated genes

c129527\_g1(1.6156) c152936\_g1(2.5657)

- egu:105040155

- Up regulated genes

c134112\_g1(0.98133)
- egu:105036609

- Up regulated genes

c129527\_g1(1.6156) c152936\_g1(2.5657)

- egu:105040155

- Up regulated genes

c134112\_g1(0.98133)
- egu:105036609

- Up regulated genes

c129527\_g1(1.6156) c152936\_g1(2.5657)

- egu:105041687

- Up regulated genes

c165885\_g1(0.75955)

- egu:105037948

- Up regulated genes

c168406\_g1(0.53391)

- egu:105037948

- Up regulated genes

c168406\_g1(0.53391)

- egu:105037948

- Up regulated genes

c168406\_g1(0.53391)

- egu:105037948

- Up regulated genes

c168406\_g1(0.53391)

Close
