# Supplementary material for: Physiological and transcriptomic responses of Lanzhou Lily (Lilium davidii, var. unicolor) to cold stress
Source: PLoS One. 2020 Jan 23;15(1):e0227921. doi: 10.1371/journal.pone.0227921 (PMC6977731; doi:10.1371/journal.pone.0227921)
Supplement: S1 Zip — (Zip). CK: control (20°C); LT: low temperature (4°C). (ZIP) [file pone.0227921.s011.zip › S1 Zip/src/egu00410.html]

egu00410


- egu:105045148

- Up regulated genes

c159016\_g1(2.0401)

- egu:105041902

- Up regulated genes

c151510\_g1(2.0621)

- egu:105055420

- Up regulated genes

c167137\_g1(2.6523) c168951\_g1(1.1729)
- egu:105044125

- Up regulated genes

c167006\_g1(0.74568) c170271\_g1(1.7042)

- egu:105042090

- Up regulated genes

c148031\_g1(0.6165)

- egu:105035697

- Up regulated genes

c173974\_g3(2.0328)
- egu:105058904

- Up regulated genes

c149000\_g1(2.4743)
- egu:105035498

- Up regulated genes

c158241\_g1(1.7429)

- egu:105035697

- Up regulated genes

c173974\_g3(2.0328)
- egu:105058904

- Up regulated genes

c149000\_g1(2.4743)
- egu:105035498

- Up regulated genes

c158241\_g1(1.7429)

- egu:105032618

- Up regulated genes

c163847\_g1(2.2781)

Close
