# Supplementary material for: Physiological and transcriptomic responses of Lanzhou Lily (Lilium davidii, var. unicolor) to cold stress
Source: PLoS One. 2020 Jan 23;15(1):e0227921. doi: 10.1371/journal.pone.0227921 (PMC6977731; doi:10.1371/journal.pone.0227921)
Supplement: S1 Zip — (Zip). CK: control (20°C); LT: low temperature (4°C). (ZIP) [file pone.0227921.s011.zip › S1 Zip/src/egu00460.html]

egu00460


- egu:105044445

- Up regulated genes

c168674\_g1(0.98979)

- egu:105045199

- Up regulated genes

c170749\_g4(1.3881) c170749\_g3(0.99004)
- egu:105042391

- Up regulated genes

c172256\_g4(3.2203) c155636\_g1(3.3411) c155636\_g2(3.0714)

- egu:105044445

- Up regulated genes

c168674\_g1(0.98979)

- egu:105038419

- Up regulated genes

c116327\_g1(1.1367)

- egu:105045199

- Up regulated genes

c170749\_g4(1.3881) c170749\_g3(0.99004)
- egu:105042391

- Up regulated genes

c172256\_g4(3.2203) c155636\_g1(3.3411) c155636\_g2(3.0714)

- egu:105045199

- Up regulated genes

c170749\_g4(1.3881) c170749\_g3(0.99004)
- egu:105042391

- Up regulated genes

c172256\_g4(3.2203) c155636\_g1(3.3411) c155636\_g2(3.0714)

- egu:105045199

- Up regulated genes

c170749\_g4(1.3881) c170749\_g3(0.99004)
- egu:105042391

- Up regulated genes

c172256\_g4(3.2203) c155636\_g1(3.3411) c155636\_g2(3.0714)

- egu:105045199

- Up regulated genes

c170749\_g4(1.3881) c170749\_g3(0.99004)
- egu:105042391

- Up regulated genes

c172256\_g4(3.2203) c155636\_g1(3.3411) c155636\_g2(3.0714)

Close
