# Supplementary material for: Physiological and transcriptomic responses of Lanzhou Lily (Lilium davidii, var. unicolor) to cold stress
Source: PLoS One. 2020 Jan 23;15(1):e0227921. doi: 10.1371/journal.pone.0227921 (PMC6977731; doi:10.1371/journal.pone.0227921)
Supplement: S1 Zip — (Zip). CK: control (20°C); LT: low temperature (4°C). (ZIP) [file pone.0227921.s011.zip › S1 Zip/src/egu00480.html]

egu00480


- egu:105044445

- Up regulated genes

c168674\_g1(0.98979)

- egu:105042890

- Up regulated genes

c159727\_g1(1.4737)

- egu:105044445

- Up regulated genes

c168674\_g1(0.98979)

- egu:105044445

- Up regulated genes

c168674\_g1(0.98979)

- egu:105056588

- Up regulated genes

c160646\_g1(3.2456)
- egu:105037855

- Up regulated genes

c173732\_g2(1.988) c165975\_g1(2.8565)
- egu:105058893

- Up regulated genes

c132043\_g1(1.1307)
- egu:105058473

- Up regulated genes

c163752\_g1(1.2883)
- egu:105032151

- Up regulated genes

c152293\_g1(1.5391)
- egu:105048525

- Up regulated genes

c163232\_g1(3.0773)

- egu:105032435

- Up regulated genes

c133447\_g2(0.58858)

- egu:105032431

- Up regulated genes

c172086\_g3(2.6529)

- egu:105032431

- Up regulated genes

c172086\_g3(2.6529)

Close
