# Supplementary material for: Physiological and transcriptomic responses of Lanzhou Lily (Lilium davidii, var. unicolor) to cold stress
Source: PLoS One. 2020 Jan 23;15(1):e0227921. doi: 10.1371/journal.pone.0227921 (PMC6977731; doi:10.1371/journal.pone.0227921)
Supplement: S1 Zip — (Zip). CK: control (20°C); LT: low temperature (4°C). (ZIP) [file pone.0227921.s011.zip › S1 Zip/src/egu00500.html]

egu00500


- egu:105042391

- Up regulated genes

c172256\_g4(3.2203) c155636\_g1(3.3411) c155636\_g2(3.0714)
- egu:105045199

- Up regulated genes

c170749\_g4(1.3881) c170749\_g3(0.99004)

- egu:105042391

- Up regulated genes

c172256\_g4(3.2203) c155636\_g1(3.3411) c155636\_g2(3.0714)
- egu:105045199

- Up regulated genes

c170749\_g4(1.3881) c170749\_g3(0.99004)

- egu:105054034

- Up regulated genes

c158824\_g1(0.69999)

- egu:105035371

- Up regulated genes

c133326\_g1(1.0801)

- egu:105054034

- Up regulated genes

c158824\_g1(0.69999)

- egu:105042391

- Up regulated genes

c172256\_g4(3.2203) c155636\_g1(3.3411) c155636\_g2(3.0714)
- egu:105045199

- Up regulated genes

c170749\_g4(1.3881) c170749\_g3(0.99004)

- egu:105046839

- Up regulated genes

c148722\_g1(1.2871)

- egu:105056682

- Up regulated genes

c157266\_g1(4.3702)

- egu:105049657

- Up regulated genes

c172045\_g1(3.2159) c165538\_g1(1.5736)
- egu:105050124

- Up regulated genes

c156393\_g2(0.87345)
- egu:105039053

- Up regulated genes

c156393\_g1(0.52375)

- egu:105055260

- Up regulated genes

c164911\_g1(0.86235)

- egu:105034870

- Up regulated genes

c171913\_g1(0.69643)

- egu:105060488

- Up regulated genes

c166861\_g1(0.67898)
- egu:105047182

- Up regulated genes

c166197\_g1(0.63246)

- egu:105055201

- Up regulated genes

c163448\_g1(1.366)

- egu:105034870

- Up regulated genes

c171913\_g1(0.69643)

- egu:105043204

- Up regulated genes

c159060\_g1(1.552) c79219\_g1(2.2707)
- egu:105043800

- Up regulated genes

c174721\_g1(4.5196)

- egu:105060892

- Up regulated genes

c173495\_g2(1.5327)

- egu:105058113

- Up regulated genes

c169649\_g1(0.4932)

- egu:105043204

- Up regulated genes

c159060\_g1(1.552) c79219\_g1(2.2707)
- egu:105043800

- Up regulated genes

c174721\_g1(4.5196)

- egu:105060892

- Up regulated genes

c173495\_g2(1.5327)

Close
