# Supplementary material for: Physiological and transcriptomic responses of Lanzhou Lily (Lilium davidii, var. unicolor) to cold stress
Source: PLoS One. 2020 Jan 23;15(1):e0227921. doi: 10.1371/journal.pone.0227921 (PMC6977731; doi:10.1371/journal.pone.0227921)
Supplement: S1 Zip — (Zip). CK: control (20°C); LT: low temperature (4°C). (ZIP) [file pone.0227921.s011.zip › S1 Zip/src/egu00510.html]

egu00510


- egu:105040617

- Up regulated genes

c158910\_g1(0.7222)

- egu:105044631

- Up regulated genes

c79137\_g1(0.80644)

- egu:105051251

- Up regulated genes

c134078\_g1(0.84186)

- egu:105039124

- Up regulated genes

c158181\_g1(0.64719)

- egu:105040617

- Up regulated genes

c158910\_g1(0.7222)

- egu:105040617

- Up regulated genes

c158910\_g1(0.7222)

- egu:105039124

- Up regulated genes

c158181\_g1(0.64719)

- egu:105039124

- Up regulated genes

c158181\_g1(0.64719)

- egu:105051251

- Up regulated genes

c134078\_g1(0.84186)

- egu:105046530

- Up regulated genes

c94583\_g1(0.75302)

- egu:105040617

- Up regulated genes

c158910\_g1(0.7222)

Close
