# Supplementary material for: Physiological and transcriptomic responses of Lanzhou Lily (Lilium davidii, var. unicolor) to cold stress
Source: PLoS One. 2020 Jan 23;15(1):e0227921. doi: 10.1371/journal.pone.0227921 (PMC6977731; doi:10.1371/journal.pone.0227921)
Supplement: S1 Zip — (Zip). CK: control (20°C); LT: low temperature (4°C). (ZIP) [file pone.0227921.s011.zip › S1 Zip/src/egu00520.html]

egu00520


- egu:105036046

- Up regulated genes

c172397\_g1(0.95764)

- egu:105047536

- Up regulated genes

c161991\_g1(0.72317)
- egu:105060220

- Up regulated genes

c172816\_g4(1.2894)
- egu:105042574

- Up regulated genes

c164458\_g2(2.7032) c164458\_g1(2.2821)

- egu:105035371

- Up regulated genes

c133326\_g1(1.0801)

- egu:105047536

- Up regulated genes

c161991\_g1(0.72317)
- egu:105060220

- Up regulated genes

c172816\_g4(1.2894)
- egu:105042574

- Up regulated genes

c164458\_g2(2.7032) c164458\_g1(2.2821)

- egu:105047457

- Up regulated genes

c159575\_g2(0.99141)
- egu:105040562

- Up regulated genes

c171416\_g1(0.70256)

- egu:105042572

- Up regulated genes

c163736\_g1(1.7307)

- egu:105055983

- Up regulated genes

c167554\_g2(0.95472) c122873\_g1(1.2297)

- egu:105045186

- Up regulated genes

c169469\_g1(1.9834)

- egu:105049059

- Up regulated genes

c164898\_g1(1.7421)

- egu:105053413

- Up regulated genes

c150645\_g1(0.4666)

- egu:105053413

- Up regulated genes

c150645\_g1(0.4666)

- egu:105035608

- Up regulated genes

c169422\_g1(0.77194)

- egu:105055260

- Up regulated genes

c164911\_g1(0.86235)

- egu:105047182

- Up regulated genes

c166197\_g1(0.63246)
- egu:105060488

- Up regulated genes

c166861\_g1(0.67898)

- egu:105056063

- Up regulated genes

c123004\_g1(0.93943)

- egu:105046676

- Up regulated genes

c72023\_g1(1.7637)
- egu:105057156

- Up regulated genes

c164111\_g1(1.5717)

Close
