# Supplementary material for: Physiological and transcriptomic responses of Lanzhou Lily (Lilium davidii, var. unicolor) to cold stress
Source: PLoS One. 2020 Jan 23;15(1):e0227921. doi: 10.1371/journal.pone.0227921 (PMC6977731; doi:10.1371/journal.pone.0227921)
Supplement: S1 Zip — (Zip). CK: control (20°C); LT: low temperature (4°C). (ZIP) [file pone.0227921.s011.zip › S1 Zip/src/egu00561.html]

egu00561


- egu:105035694

- Up regulated genes

c140940\_g1(2.2856)

- egu:105049423

- Up regulated genes

c164009\_g2(5.8326)

- egu:105041372

- Up regulated genes

c168575\_g1(0.56698)
- egu:105046158

- Up regulated genes

c160696\_g1(0.82836)

- egu:105042090

- Up regulated genes

c148031\_g1(0.6165)

Close
