# Supplementary material for: Physiological and transcriptomic responses of Lanzhou Lily (Lilium davidii, var. unicolor) to cold stress
Source: PLoS One. 2020 Jan 23;15(1):e0227921. doi: 10.1371/journal.pone.0227921 (PMC6977731; doi:10.1371/journal.pone.0227921)
Supplement: S1 Zip — (Zip). CK: control (20°C); LT: low temperature (4°C). (ZIP) [file pone.0227921.s011.zip › S1 Zip/src/egu00565.html]

egu00565


- egu:105033970

- Up regulated genes

c165623\_g2(1.1898)
- egu:105045139

- Up regulated genes

c133817\_g1(0.83679)
- egu:105054164

- Up regulated genes

c167245\_g1(1.5987)

- egu:105046237

- Up regulated genes

c164800\_g1(0.87603)

Close
