# Supplementary material for: Physiological and transcriptomic responses of Lanzhou Lily (Lilium davidii, var. unicolor) to cold stress
Source: PLoS One. 2020 Jan 23;15(1):e0227921. doi: 10.1371/journal.pone.0227921 (PMC6977731; doi:10.1371/journal.pone.0227921)
Supplement: S1 Zip — (Zip). CK: control (20°C); LT: low temperature (4°C). (ZIP) [file pone.0227921.s011.zip › S1 Zip/src/egu00620.html]

egu00620


- egu:105058702

- Up regulated genes

c163723\_g1(0.8624)
- egu:105043985

- Up regulated genes

c166833\_g1(0.67016)

- egu:105058982

- Up regulated genes

c156756\_g2(2.2972)
- egu:105035292

- Up regulated genes

c188298\_g1(2.0535)
- egu:105038179

- Up regulated genes

c161769\_g1(0.81464)
- egu:105057280

- Up regulated genes

c172074\_g1(2.793)
- egu:105053882

- Up regulated genes

c158821\_g1(0.52215)
- egu:105042489

- Up regulated genes

c156756\_g1(2.052)

- egu:105058543

- Up regulated genes

c145522\_g1(1.236)

- egu:105042090

- Up regulated genes

c148031\_g1(0.6165)

- egu:105041644

- Up regulated genes

c165048\_g1(0.7123)

- egu:105059487

- Up regulated genes

c162039\_g1(0.48153)

Close
