# Supplementary material for: Physiological and transcriptomic responses of Lanzhou Lily (Lilium davidii, var. unicolor) to cold stress
Source: PLoS One. 2020 Jan 23;15(1):e0227921. doi: 10.1371/journal.pone.0227921 (PMC6977731; doi:10.1371/journal.pone.0227921)
Supplement: S1 Zip — (Zip). CK: control (20°C); LT: low temperature (4°C). (ZIP) [file pone.0227921.s011.zip › S1 Zip/src/egu00710.html]

egu00710


- egu:105059450

- Up regulated genes

c170991\_g2(0.56857)

- egu:105047057

- Up regulated genes

c159520\_g1(1.375)

- egu:105059872

- Up regulated genes

c157432\_g1(0.46067)

- egu:105058702

- Up regulated genes

c163723\_g1(0.8624)
- egu:105043985

- Up regulated genes

c166833\_g1(0.67016)

- egu:105058543

- Up regulated genes

c145522\_g1(1.236)

- egu:105059450

- Up regulated genes

c170991\_g2(0.56857)

- egu:105059882

- Up regulated genes

c145285\_g1(2.0457)
- egu:105051363

- Up regulated genes

c105074\_g2(1.0862)

- egu:105058543

- Up regulated genes

c145522\_g1(1.236)

- egu:105058702

- Up regulated genes

c163723\_g1(0.8624)
- egu:105043985

- Up regulated genes

c166833\_g1(0.67016)

Close
