# Supplementary material for: Physiological and transcriptomic responses of Lanzhou Lily (Lilium davidii, var. unicolor) to cold stress
Source: PLoS One. 2020 Jan 23;15(1):e0227921. doi: 10.1371/journal.pone.0227921 (PMC6977731; doi:10.1371/journal.pone.0227921)
Supplement: S1 Zip — (Zip). CK: control (20°C); LT: low temperature (4°C). (ZIP) [file pone.0227921.s011.zip › S1 Zip/src/egu00750.html]

egu00750


- egu:105035898

- Up regulated genes

c224416\_g1(2.3586)

- egu:105047312

- Up regulated genes

c161228\_g1(2.9337) c173447\_g1(1.8723)

- egu:105047312

- Up regulated genes

c161228\_g1(2.9337) c173447\_g1(1.8723)

- egu:105047312

- Up regulated genes

c161228\_g1(2.9337) c173447\_g1(1.8723)

- egu:105053256

- Up regulated genes

c173425\_g1(0.91322)

Close
