# Supplementary material for: Physiological and transcriptomic responses of Lanzhou Lily (Lilium davidii, var. unicolor) to cold stress
Source: PLoS One. 2020 Jan 23;15(1):e0227921. doi: 10.1371/journal.pone.0227921 (PMC6977731; doi:10.1371/journal.pone.0227921)
Supplement: S1 Zip — (Zip). CK: control (20°C); LT: low temperature (4°C). (ZIP) [file pone.0227921.s011.zip › S1 Zip/src/egu00900.html]

egu00900


- egu:105034095

- Up regulated genes

c146169\_g1(0.52161)

- egu:105059274

- Up regulated genes

c161281\_g1(0.79018)

- egu:105039667

- Up regulated genes

c159165\_g1(1.3222)

- egu:105036025

- Up regulated genes

c95088\_g1(0.53613)

Close
