# Supplementary material for: Physiological and transcriptomic responses of Lanzhou Lily (Lilium davidii, var. unicolor) to cold stress
Source: PLoS One. 2020 Jan 23;15(1):e0227921. doi: 10.1371/journal.pone.0227921 (PMC6977731; doi:10.1371/journal.pone.0227921)
Supplement: S1 Zip — (Zip). CK: control (20°C); LT: low temperature (4°C). (ZIP) [file pone.0227921.s011.zip › S1 Zip/src/egu00904.html]

egu00904


- egu:105034258

- Up regulated genes

c162655\_g1(3.9328)
- egu:105043827

- Up regulated genes

c167196\_g2(2.3599)

- egu:105034258

- Up regulated genes

c162655\_g1(3.9328)
- egu:105043827

- Up regulated genes

c167196\_g2(2.3599)

- egu:105034258

- Up regulated genes

c162655\_g1(3.9328)
- egu:105043827

- Up regulated genes

c167196\_g2(2.3599)

- egu:105034258

- Up regulated genes

c162655\_g1(3.9328)
- egu:105043827

- Up regulated genes

c167196\_g2(2.3599)

Close
