# Supplementary material for: Physiological and transcriptomic responses of Lanzhou Lily (Lilium davidii, var. unicolor) to cold stress
Source: PLoS One. 2020 Jan 23;15(1):e0227921. doi: 10.1371/journal.pone.0227921 (PMC6977731; doi:10.1371/journal.pone.0227921)
Supplement: S1 Zip — (Zip). CK: control (20°C); LT: low temperature (4°C). (ZIP) [file pone.0227921.s011.zip › S1 Zip/src/egu00906.html]

egu00906


- egu:105060532

- Up regulated genes

c163397\_g1(1.016)

- egu:105060532

- Up regulated genes

c163397\_g1(1.016)

- egu:105038273

- Up regulated genes

c168301\_g1(0.69621)

- egu:105038273

- Up regulated genes

c168301\_g1(0.69621)

- egu:105033850

- Up regulated genes

c173485\_g1(1.1365)
- egu:105058241

- Up regulated genes

c154973\_g1(1.4227)

- egu:105035937

- Up regulated genes

c173509\_g1(0.94689)

- egu:105046802

- Up regulated genes

c168117\_g1(2.85) c162048\_g1(3.3425)

- egu:105046802

- Up regulated genes

c168117\_g1(2.85) c162048\_g1(3.3425)

- egu:105060532

- Up regulated genes

c163397\_g1(1.016)

- egu:105060532

- Up regulated genes

c163397\_g1(1.016)

- egu:105060532

- Up regulated genes

c163397\_g1(1.016)

Close
