# Supplementary material for: Physiological and transcriptomic responses of Lanzhou Lily (Lilium davidii, var. unicolor) to cold stress
Source: PLoS One. 2020 Jan 23;15(1):e0227921. doi: 10.1371/journal.pone.0227921 (PMC6977731; doi:10.1371/journal.pone.0227921)
Supplement: S1 Zip — (Zip). CK: control (20°C); LT: low temperature (4°C). (ZIP) [file pone.0227921.s011.zip › S1 Zip/src/egu00940.html]

egu00940


- egu:105035781

- Up regulated genes

c168304\_g1(0.73196)
- egu:105054501

- Up regulated genes

c148234\_g1(5.8458)
- egu:105055673

- Up regulated genes

c168304\_g2(3.9853) c168304\_g3(5.2016) c166080\_g1(4.7318)

- egu:105053813

- Up regulated genes

c134164\_g1(5.8972)
- egu:105039619

- Up regulated genes

c162887\_g1(0.93581)

- egu:105045199

- Up regulated genes

c170749\_g4(1.3881) c170749\_g3(0.99004)
- egu:105042391

- Up regulated genes

c155636\_g2(3.0714) c172256\_g4(3.2203) c155636\_g1(3.3411)

- egu:105050729

- Up regulated genes

c165035\_g1(1.3319)

- egu:105050729

- Up regulated genes

c165035\_g1(1.3319)

- egu:105053813

- Up regulated genes

c134164\_g1(5.8972)
- egu:105039619

- Up regulated genes

c162887\_g1(0.93581)

- egu:105053813

- Up regulated genes

c134164\_g1(5.8972)
- egu:105039619

- Up regulated genes

c162887\_g1(0.93581)

- egu:105050729

- Up regulated genes

c165035\_g1(1.3319)

- egu:105050729

- Up regulated genes

c165035\_g1(1.3319)

- egu:105050729

- Up regulated genes

c165035\_g1(1.3319)

- egu:105038037

- Up regulated genes

c172938\_g7(3.4541)
- egu:105051975

- Up regulated genes

c169830\_g1(1.0742)
- egu:105056224

- Up regulated genes

c140986\_g1(2.4063)

- egu:105037657

- Up regulated genes

c161779\_g15(2.7014) c170056\_g1(4.6538)

- egu:105037657

- Up regulated genes

c161779\_g15(2.7014) c170056\_g1(4.6538)

- egu:105037657

- Up regulated genes

c161779\_g15(2.7014) c170056\_g1(4.6538)

- egu:105037657

- Up regulated genes

c161779\_g15(2.7014) c170056\_g1(4.6538)

- egu:105037657

- Up regulated genes

c161779\_g15(2.7014) c170056\_g1(4.6538)

- egu:105038037

- Up regulated genes

c172938\_g7(3.4541)
- egu:105051975

- Up regulated genes

c169830\_g1(1.0742)
- egu:105056224

- Up regulated genes

c140986\_g1(2.4063)

- egu:105038037

- Up regulated genes

c172938\_g7(3.4541)
- egu:105051975

- Up regulated genes

c169830\_g1(1.0742)
- egu:105056224

- Up regulated genes

c140986\_g1(2.4063)

- egu:105038037

- Up regulated genes

c172938\_g7(3.4541)
- egu:105051975

- Up regulated genes

c169830\_g1(1.0742)
- egu:105056224

- Up regulated genes

c140986\_g1(2.4063)

- egu:105053813

- Up regulated genes

c134164\_g1(5.8972)
- egu:105039619

- Up regulated genes

c162887\_g1(0.93581)

- egu:105053813

- Up regulated genes

c134164\_g1(5.8972)
- egu:105039619

- Up regulated genes

c162887\_g1(0.93581)

- egu:105053813

- Up regulated genes

c134164\_g1(5.8972)
- egu:105039619

- Up regulated genes

c162887\_g1(0.93581)

- egu:105050729

- Up regulated genes

c165035\_g1(1.3319)

- egu:105045995

- Up regulated genes

c165806\_g1(3.5125)

- egu:105045995

- Up regulated genes

c165806\_g1(3.5125)

Close
