# Supplementary material for: Physiological and transcriptomic responses of Lanzhou Lily (Lilium davidii, var. unicolor) to cold stress
Source: PLoS One. 2020 Jan 23;15(1):e0227921. doi: 10.1371/journal.pone.0227921 (PMC6977731; doi:10.1371/journal.pone.0227921)
Supplement: S1 Zip — (Zip). CK: control (20°C); LT: low temperature (4°C). (ZIP) [file pone.0227921.s011.zip › S1 Zip/src/egu00941.html]

egu00941


- egu:105058232

- Up regulated genes

c146719\_g1(2.8331)

- egu:105054663

- Up regulated genes

c166729\_g1(5.4331)

- egu:105054663

- Up regulated genes

c166729\_g1(5.4331)

- egu:105035984

- Up regulated genes

c167553\_g1(2.6788)

- egu:105035984

- Up regulated genes

c167553\_g1(2.6788)

- egu:105035984

- Up regulated genes

c167553\_g1(2.6788)

- egu:105050962

- Up regulated genes

c151401\_g1(4.5093) c174513\_g3(4.5905) c146896\_g1(6.4969) c146896\_g3(7.3843) c174513\_g1(5.3633)
- egu:105036364

- Up regulated genes

c155146\_g1(7.5424)
- egu:105035716

- Up regulated genes

c169857\_g1(2.9204)

- egu:105058071

- Up regulated genes

c154616\_g1(6.2597)

- egu:105035842

- Up regulated genes

c155053\_g1(Inf)

- egu:105058232

- Up regulated genes

c146719\_g1(2.8331)

- egu:105035842

- Up regulated genes

c155053\_g1(Inf)

- egu:105058071

- Up regulated genes

c154616\_g1(6.2597)

- egu:105035984

- Up regulated genes

c167553\_g1(2.6788)

- egu:105058071

- Up regulated genes

c154616\_g1(6.2597)

- egu:105054663

- Up regulated genes

c166729\_g1(5.4331)

- egu:105058071

- Up regulated genes

c154616\_g1(6.2597)

- egu:105058071

- Up regulated genes

c154616\_g1(6.2597)

- egu:105058232

- Up regulated genes

c146719\_g1(2.8331)

- egu:105058071

- Up regulated genes

c154616\_g1(6.2597)

- egu:105035842

- Up regulated genes

c155053\_g1(Inf)

- egu:105058232

- Up regulated genes

c146719\_g1(2.8331)

- egu:105035842

- Up regulated genes

c155053\_g1(Inf)

- egu:105035842

- Up regulated genes

c155053\_g1(Inf)

- egu:105054663

- Up regulated genes

c166729\_g1(5.4331)

- egu:105050962

- Up regulated genes

c151401\_g1(4.5093) c174513\_g3(4.5905) c146896\_g1(6.4969) c146896\_g3(7.3843) c174513\_g1(5.3633)
- egu:105036364

- Up regulated genes

c155146\_g1(7.5424)
- egu:105035716

- Up regulated genes

c169857\_g1(2.9204)

- egu:105050962

- Up regulated genes

c151401\_g1(4.5093) c174513\_g3(4.5905) c146896\_g1(6.4969) c146896\_g3(7.3843) c174513\_g1(5.3633)
- egu:105036364

- Up regulated genes

c155146\_g1(7.5424)
- egu:105035716

- Up regulated genes

c169857\_g1(2.9204)

- egu:105050962

- Up regulated genes

c151401\_g1(4.5093) c174513\_g3(4.5905) c146896\_g1(6.4969) c146896\_g3(7.3843) c174513\_g1(5.3633)
- egu:105036364

- Up regulated genes

c155146\_g1(7.5424)
- egu:105035716

- Up regulated genes

c169857\_g1(2.9204)

- egu:105054663

- Up regulated genes

c166729\_g1(5.4331)

- egu:105054663

- Up regulated genes

c166729\_g1(5.4331)

- egu:105045995

- Up regulated genes

c165806\_g1(3.5125)

- egu:105050962

- Up regulated genes

c151401\_g1(4.5093) c174513\_g3(4.5905) c146896\_g1(6.4969) c146896\_g3(7.3843) c174513\_g1(5.3633)
- egu:105036364

- Up regulated genes

c155146\_g1(7.5424)
- egu:105035716

- Up regulated genes

c169857\_g1(2.9204)

Close
