# Supplementary material for: Physiological and transcriptomic responses of Lanzhou Lily (Lilium davidii, var. unicolor) to cold stress
Source: PLoS One. 2020 Jan 23;15(1):e0227921. doi: 10.1371/journal.pone.0227921 (PMC6977731; doi:10.1371/journal.pone.0227921)
Supplement: S1 Zip — (Zip). CK: control (20°C); LT: low temperature (4°C). (ZIP) [file pone.0227921.s011.zip › S1 Zip/src/egu00960.html]

egu00960


- egu:105044125

- Up regulated genes

c170271\_g1(1.7042) c167006\_g1(0.74568)
- egu:105055420

- Up regulated genes

c167137\_g1(2.6523) c168951\_g1(1.1729)

- egu:105044125

- Up regulated genes

c170271\_g1(1.7042) c167006\_g1(0.74568)
- egu:105055420

- Up regulated genes

c167137\_g1(2.6523) c168951\_g1(1.1729)

- egu:105037948

- Up regulated genes

c168406\_g1(0.53391)

Close
