# Supplementary material for: Physiological and transcriptomic responses of Lanzhou Lily (Lilium davidii, var. unicolor) to cold stress
Source: PLoS One. 2020 Jan 23;15(1):e0227921. doi: 10.1371/journal.pone.0227921 (PMC6977731; doi:10.1371/journal.pone.0227921)
Supplement: S1 Zip — (Zip). CK: control (20°C); LT: low temperature (4°C). (ZIP) [file pone.0227921.s011.zip › S1 Zip/src/egu01040.html]

egu01040


- egu:105052984

- Up regulated genes

c168511\_g1(1.3724)

- egu:105045727

- Up regulated genes

c162055\_g1(0.89454)

- egu:105041633

- Up regulated genes

c153824\_g1(0.77094)

- egu:105052984

- Up regulated genes

c168511\_g1(1.3724)

- egu:105045727

- Up regulated genes

c162055\_g1(0.89454)

- egu:105052984

- Up regulated genes

c168511\_g1(1.3724)

- egu:105045727

- Up regulated genes

c162055\_g1(0.89454)

- egu:105052984

- Up regulated genes

c168511\_g1(1.3724)

- egu:105045727

- Up regulated genes

c162055\_g1(0.89454)

- egu:105052984

- Up regulated genes

c168511\_g1(1.3724)

- egu:105045727

- Up regulated genes

c162055\_g1(0.89454)

- egu:105052984

- Up regulated genes

c168511\_g1(1.3724)

- egu:105045727

- Up regulated genes

c162055\_g1(0.89454)

- egu:105052984

- Up regulated genes

c168511\_g1(1.3724)

- egu:105045727

- Up regulated genes

c162055\_g1(0.89454)

- egu:105052984

- Up regulated genes

c168511\_g1(1.3724)

- egu:105045727

- Up regulated genes

c162055\_g1(0.89454)

- egu:105041633

- Up regulated genes

c153824\_g1(0.77094)

- egu:105052984

- Up regulated genes

c168511\_g1(1.3724)

- egu:105045727

- Up regulated genes

c162055\_g1(0.89454)

- egu:105052984

- Up regulated genes

c168511\_g1(1.3724)

- egu:105045727

- Up regulated genes

c162055\_g1(0.89454)

- egu:105052984

- Up regulated genes

c168511\_g1(1.3724)

- egu:105045727

- Up regulated genes

c162055\_g1(0.89454)

- egu:105052984

- Up regulated genes

c168511\_g1(1.3724)

- egu:105045727

- Up regulated genes

c162055\_g1(0.89454)

- egu:105052984

- Up regulated genes

c168511\_g1(1.3724)

- egu:105045727

- Up regulated genes

c162055\_g1(0.89454)

- egu:105052984

- Up regulated genes

c168511\_g1(1.3724)

- egu:105045727

- Up regulated genes

c162055\_g1(0.89454)

Close
