# Supplementary material for: Physiological and transcriptomic responses of Lanzhou Lily (Lilium davidii, var. unicolor) to cold stress
Source: PLoS One. 2020 Jan 23;15(1):e0227921. doi: 10.1371/journal.pone.0227921 (PMC6977731; doi:10.1371/journal.pone.0227921)
Supplement: S1 Zip — (Zip). CK: control (20°C); LT: low temperature (4°C). (ZIP) [file pone.0227921.s011.zip › S1 Zip/src/egu01110.html]

egu01110


- egu:105038882

- Up regulated genes

c151973\_g1(0.59053)

- egu:105043827

- Up regulated genes

c167196\_g2(2.3599)
- egu:105034258

- Up regulated genes

c162655\_g1(3.9328)

- egu:105043827

- Up regulated genes

c167196\_g2(2.3599)
- egu:105034258

- Up regulated genes

c162655\_g1(3.9328)

- egu:105045199

- Up regulated genes

c170749\_g4(1.3881) c170749\_g3(0.99004)
- egu:105042391

- Up regulated genes

c172256\_g4(3.2203) c155636\_g1(3.3411) c155636\_g2(3.0714)

- egu:105047182

- Up regulated genes

c166197\_g1(0.63246)
- egu:105060488

- Up regulated genes

c166861\_g1(0.67898)

- egu:105055201

- Up regulated genes

c163448\_g1(1.366)

- egu:105058113

- Up regulated genes

c169649\_g1(0.4932)

- egu:105058113

- Up regulated genes

c169649\_g1(0.4932)

- egu:105058113

- Up regulated genes

c169649\_g1(0.4932)

- egu:105058113

- Up regulated genes

c169649\_g1(0.4932)

- egu:105058113

- Up regulated genes

c169649\_g1(0.4932)

- egu:105058113

- Up regulated genes

c169649\_g1(0.4932)

- egu:105058113

- Up regulated genes

c169649\_g1(0.4932)

- egu:105058113

- Up regulated genes

c169649\_g1(0.4932)

- egu:105058113

- Up regulated genes

c169649\_g1(0.4932)

- egu:105058113

- Up regulated genes

c169649\_g1(0.4932)

- egu:105058113

- Up regulated genes

c169649\_g1(0.4932)

- egu:105053413

- Up regulated genes

c150645\_g1(0.4666)

- egu:105038499

- Up regulated genes

c168307\_g1(4.6883)

- egu:105036034

- Up regulated genes

c135007\_g1(0.70355)

- egu:105037948

- Up regulated genes

c168406\_g1(0.53391)

- egu:105032793

- Up regulated genes

c140061\_g1(2.9643) c155351\_g1(2.9588) c155351\_g2(2.8648)

- egu:105058884

- Up regulated genes

c166123\_g1(0.62807)

- egu:105056718

- Up regulated genes

c162165\_g1(3.3164)
- egu:105053112

- Up regulated genes

c173762\_g1(2.5689)

- egu:105056718

- Up regulated genes

c162165\_g1(3.3164)
- egu:105053112

- Up regulated genes

c173762\_g1(2.5689)

- egu:105056718

- Up regulated genes

c162165\_g1(3.3164)
- egu:105053112

- Up regulated genes

c173762\_g1(2.5689)

- egu:105056718

- Up regulated genes

c162165\_g1(3.3164)
- egu:105053112

- Up regulated genes

c173762\_g1(2.5689)

- egu:105056718

- Up regulated genes

c162165\_g1(3.3164)
- egu:105053112

- Up regulated genes

c173762\_g1(2.5689)

- egu:105056718

- Up regulated genes

c162165\_g1(3.3164)
- egu:105053112

- Up regulated genes

c173762\_g1(2.5689)

- egu:105056718

- Up regulated genes

c162165\_g1(3.3164)
- egu:105053112

- Up regulated genes

c173762\_g1(2.5689)

- egu:105056718

- Up regulated genes

c162165\_g1(3.3164)
- egu:105053112

- Up regulated genes

c173762\_g1(2.5689)

- egu:105056718

- Up regulated genes

c162165\_g1(3.3164)
- egu:105053112

- Up regulated genes

c173762\_g1(2.5689)

- egu:105056718

- Up regulated genes

c162165\_g1(3.3164)
- egu:105053112

- Up regulated genes

c173762\_g1(2.5689)

- egu:105056718

- Up regulated genes

c162165\_g1(3.3164)
- egu:105053112

- Up regulated genes

c173762\_g1(2.5689)

- egu:105039328

- Up regulated genes

c166548\_g2(0.76477)

- egu:105038559

- Up regulated genes

c172946\_g1(1.1727)

- egu:105041633

- Up regulated genes

c153824\_g1(0.77094)

- egu:105041633

- Up regulated genes

c153824\_g1(0.77094)

- egu:105041633

- Up regulated genes

c153824\_g1(0.77094)

- egu:105041633

- Up regulated genes

c153824\_g1(0.77094)

- egu:105041633

- Up regulated genes

c153824\_g1(0.77094)

- egu:105041633

- Up regulated genes

c153824\_g1(0.77094)

- egu:105041633

- Up regulated genes

c153824\_g1(0.77094)

- egu:105041633

- Up regulated genes

c153824\_g1(0.77094)

- egu:105041633

- Up regulated genes

c153824\_g1(0.77094)

- egu:105041633

- Up regulated genes

c153824\_g1(0.77094)

- egu:105041633

- Up regulated genes

c153824\_g1(0.77094)

- egu:105041633

- Up regulated genes

c153824\_g1(0.77094)

- egu:105041633

- Up regulated genes

c153824\_g1(0.77094)

- egu:105041633

- Up regulated genes

c153824\_g1(0.77094)

- egu:105041633

- Up regulated genes

c153824\_g1(0.77094)

- egu:105041633

- Up regulated genes

c153824\_g1(0.77094)

- egu:105041633

- Up regulated genes

c153824\_g1(0.77094)

- egu:105041633

- Up regulated genes

c153824\_g1(0.77094)

- egu:105041633

- Up regulated genes

c153824\_g1(0.77094)

- egu:105041633

- Up regulated genes

c153824\_g1(0.77094)

- egu:105041633

- Up regulated genes

c153824\_g1(0.77094)

- egu:105041633

- Up regulated genes

c153824\_g1(0.77094)

- egu:105041633

- Up regulated genes

c153824\_g1(0.77094)

- egu:105041633

- Up regulated genes

c153824\_g1(0.77094)

- egu:105041633

- Up regulated genes

c153824\_g1(0.77094)

- egu:105041633

- Up regulated genes

c153824\_g1(0.77094)

- egu:105041633

- Up regulated genes

c153824\_g1(0.77094)

- egu:105041633

- Up regulated genes

c153824\_g1(0.77094)

- egu:105041633

- Up regulated genes

c153824\_g1(0.77094)

- egu:105041633

- Up regulated genes

c153824\_g1(0.77094)

- egu:105041633

- Up regulated genes

c153824\_g1(0.77094)

- egu:105041633

- Up regulated genes

c153824\_g1(0.77094)

- egu:105041633

- Up regulated genes

c153824\_g1(0.77094)

- egu:105041633

- Up regulated genes

c153824\_g1(0.77094)

- egu:105041633

- Up regulated genes

c153824\_g1(0.77094)

- egu:105041633

- Up regulated genes

c153824\_g1(0.77094)

- egu:105041633

- Up regulated genes

c153824\_g1(0.77094)

- egu:105041633

- Up regulated genes

c153824\_g1(0.77094)

- egu:105041633

- Up regulated genes

c153824\_g1(0.77094)

- egu:105041633

- Up regulated genes

c153824\_g1(0.77094)

- egu:105041633

- Up regulated genes

c153824\_g1(0.77094)

- egu:105041633

- Up regulated genes

c153824\_g1(0.77094)

- egu:105041633

- Up regulated genes

c153824\_g1(0.77094)

- egu:105041633

- Up regulated genes

c153824\_g1(0.77094)

- egu:105041633

- Up regulated genes

c153824\_g1(0.77094)

- egu:105041633

- Up regulated genes

c153824\_g1(0.77094)

- egu:105041633

- Up regulated genes

c153824\_g1(0.77094)

- egu:105041633

- Up regulated genes

c153824\_g1(0.77094)

- egu:105041633

- Up regulated genes

c153824\_g1(0.77094)

- egu:105041633

- Up regulated genes

c153824\_g1(0.77094)

- egu:105041633

- Up regulated genes

c153824\_g1(0.77094)

- egu:105041633

- Up regulated genes

c153824\_g1(0.77094)

- egu:105041633

- Up regulated genes

c153824\_g1(0.77094)

- egu:105041633

- Up regulated genes

c153824\_g1(0.77094)

- egu:105041633

- Up regulated genes

c153824\_g1(0.77094)

- egu:105041633

- Up regulated genes

c153824\_g1(0.77094)

- egu:105041633

- Up regulated genes

c153824\_g1(0.77094)

- egu:105041633

- Up regulated genes

c153824\_g1(0.77094)

- egu:105041633

- Up regulated genes

c153824\_g1(0.77094)

- egu:105041633

- Up regulated genes

c153824\_g1(0.77094)

- egu:105041633

- Up regulated genes

c153824\_g1(0.77094)

- egu:105041633

- Up regulated genes

c153824\_g1(0.77094)

- egu:105041633

- Up regulated genes

c153824\_g1(0.77094)

- egu:105056718

- Up regulated genes

c162165\_g1(3.3164)
- egu:105053112

- Up regulated genes

c173762\_g1(2.5689)

- egu:105039328

- Up regulated genes

c166548\_g2(0.76477)

- egu:105054164

- Up regulated genes

c167245\_g1(1.5987)
- egu:105033970

- Up regulated genes

c165623\_g2(1.1898)
- egu:105045139

- Up regulated genes

c133817\_g1(0.83679)

- egu:105054164

- Up regulated genes

c167245\_g1(1.5987)
- egu:105033970

- Up regulated genes

c165623\_g2(1.1898)
- egu:105045139

- Up regulated genes

c133817\_g1(0.83679)

- egu:105054164

- Up regulated genes

c167245\_g1(1.5987)
- egu:105033970

- Up regulated genes

c165623\_g2(1.1898)
- egu:105045139

- Up regulated genes

c133817\_g1(0.83679)

- egu:105054164

- Up regulated genes

c167245\_g1(1.5987)
- egu:105033970

- Up regulated genes

c165623\_g2(1.1898)
- egu:105045139

- Up regulated genes

c133817\_g1(0.83679)

- egu:105054164

- Up regulated genes

c167245\_g1(1.5987)
- egu:105033970

- Up regulated genes

c165623\_g2(1.1898)
- egu:105045139

- Up regulated genes

c133817\_g1(0.83679)

- egu:105054164

- Up regulated genes

c167245\_g1(1.5987)
- egu:105033970

- Up regulated genes

c165623\_g2(1.1898)
- egu:105045139

- Up regulated genes

c133817\_g1(0.83679)

- egu:105054164

- Up regulated genes

c167245\_g1(1.5987)
- egu:105033970

- Up regulated genes

c165623\_g2(1.1898)
- egu:105045139

- Up regulated genes

c133817\_g1(0.83679)

- egu:105054164

- Up regulated genes

c167245\_g1(1.5987)
- egu:105033970

- Up regulated genes

c165623\_g2(1.1898)
- egu:105045139

- Up regulated genes

c133817\_g1(0.83679)

- egu:105054164

- Up regulated genes

c167245\_g1(1.5987)
- egu:105033970

- Up regulated genes

c165623\_g2(1.1898)
- egu:105045139

- Up regulated genes

c133817\_g1(0.83679)

- egu:105054164

- Up regulated genes

c167245\_g1(1.5987)
- egu:105033970

- Up regulated genes

c165623\_g2(1.1898)
- egu:105045139

- Up regulated genes

c133817\_g1(0.83679)

- egu:105054164

- Up regulated genes

c167245\_g1(1.5987)
- egu:105033970

- Up regulated genes

c165623\_g2(1.1898)
- egu:105045139

- Up regulated genes

c133817\_g1(0.83679)

- egu:105054164

- Up regulated genes

c167245\_g1(1.5987)
- egu:105033970

- Up regulated genes

c165623\_g2(1.1898)
- egu:105045139

- Up regulated genes

c133817\_g1(0.83679)

- egu:105054164

- Up regulated genes

c167245\_g1(1.5987)
- egu:105033970

- Up regulated genes

c165623\_g2(1.1898)
- egu:105045139

- Up regulated genes

c133817\_g1(0.83679)

- egu:105054164

- Up regulated genes

c167245\_g1(1.5987)
- egu:105033970

- Up regulated genes

c165623\_g2(1.1898)
- egu:105045139

- Up regulated genes

c133817\_g1(0.83679)

- egu:105054164

- Up regulated genes

c167245\_g1(1.5987)
- egu:105033970

- Up regulated genes

c165623\_g2(1.1898)
- egu:105045139

- Up regulated genes

c133817\_g1(0.83679)

- egu:105054164

- Up regulated genes

c167245\_g1(1.5987)
- egu:105033970

- Up regulated genes

c165623\_g2(1.1898)
- egu:105045139

- Up regulated genes

c133817\_g1(0.83679)

- egu:105054164

- Up regulated genes

c167245\_g1(1.5987)
- egu:105033970

- Up regulated genes

c165623\_g2(1.1898)
- egu:105045139

- Up regulated genes

c133817\_g1(0.83679)

- egu:105054164

- Up regulated genes

c167245\_g1(1.5987)
- egu:105033970

- Up regulated genes

c165623\_g2(1.1898)
- egu:105045139

- Up regulated genes

c133817\_g1(0.83679)

- egu:105054164

- Up regulated genes

c167245\_g1(1.5987)
- egu:105033970

- Up regulated genes

c165623\_g2(1.1898)
- egu:105045139

- Up regulated genes

c133817\_g1(0.83679)

- egu:105054164

- Up regulated genes

c167245\_g1(1.5987)
- egu:105033970

- Up regulated genes

c165623\_g2(1.1898)
- egu:105045139

- Up regulated genes

c133817\_g1(0.83679)

- egu:105054164

- Up regulated genes

c167245\_g1(1.5987)
- egu:105033970

- Up regulated genes

c165623\_g2(1.1898)
- egu:105045139

- Up regulated genes

c133817\_g1(0.83679)

- egu:105054164

- Up regulated genes

c167245\_g1(1.5987)
- egu:105033970

- Up regulated genes

c165623\_g2(1.1898)
- egu:105045139

- Up regulated genes

c133817\_g1(0.83679)

- egu:105054164

- Up regulated genes

c167245\_g1(1.5987)
- egu:105033970

- Up regulated genes

c165623\_g2(1.1898)
- egu:105045139

- Up regulated genes

c133817\_g1(0.83679)

- egu:105054164

- Up regulated genes

c167245\_g1(1.5987)
- egu:105033970

- Up regulated genes

c165623\_g2(1.1898)
- egu:105045139

- Up regulated genes

c133817\_g1(0.83679)

- egu:105054164

- Up regulated genes

c167245\_g1(1.5987)
- egu:105033970

- Up regulated genes

c165623\_g2(1.1898)
- egu:105045139

- Up regulated genes

c133817\_g1(0.83679)

- egu:105054164

- Up regulated genes

c167245\_g1(1.5987)
- egu:105033970

- Up regulated genes

c165623\_g2(1.1898)
- egu:105045139

- Up regulated genes

c133817\_g1(0.83679)

- egu:105054164

- Up regulated genes

c167245\_g1(1.5987)
- egu:105033970

- Up regulated genes

c165623\_g2(1.1898)
- egu:105045139

- Up regulated genes

c133817\_g1(0.83679)

- egu:105054164

- Up regulated genes

c167245\_g1(1.5987)
- egu:105033970

- Up regulated genes

c165623\_g2(1.1898)
- egu:105045139

- Up regulated genes

c133817\_g1(0.83679)

- egu:105054164

- Up regulated genes

c167245\_g1(1.5987)
- egu:105033970

- Up regulated genes

c165623\_g2(1.1898)
- egu:105045139

- Up regulated genes

c133817\_g1(0.83679)

- egu:105054164

- Up regulated genes

c167245\_g1(1.5987)
- egu:105033970

- Up regulated genes

c165623\_g2(1.1898)
- egu:105045139

- Up regulated genes

c133817\_g1(0.83679)

- egu:105054164

- Up regulated genes

c167245\_g1(1.5987)
- egu:105033970

- Up regulated genes

c165623\_g2(1.1898)
- egu:105045139

- Up regulated genes

c133817\_g1(0.83679)

- egu:105054164

- Up regulated genes

c167245\_g1(1.5987)
- egu:105033970

- Up regulated genes

c165623\_g2(1.1898)
- egu:105045139

- Up regulated genes

c133817\_g1(0.83679)

- egu:105054164

- Up regulated genes

c167245\_g1(1.5987)
- egu:105033970

- Up regulated genes

c165623\_g2(1.1898)
- egu:105045139

- Up regulated genes

c133817\_g1(0.83679)

- egu:105054164

- Up regulated genes

c167245\_g1(1.5987)
- egu:105033970

- Up regulated genes

c165623\_g2(1.1898)
- egu:105045139

- Up regulated genes

c133817\_g1(0.83679)

- egu:105054164

- Up regulated genes

c167245\_g1(1.5987)
- egu:105033970

- Up regulated genes

c165623\_g2(1.1898)
- egu:105045139

- Up regulated genes

c133817\_g1(0.83679)

- egu:105054164

- Up regulated genes

c167245\_g1(1.5987)
- egu:105033970

- Up regulated genes

c165623\_g2(1.1898)
- egu:105045139

- Up regulated genes

c133817\_g1(0.83679)

- egu:105054164

- Up regulated genes

c167245\_g1(1.5987)
- egu:105033970

- Up regulated genes

c165623\_g2(1.1898)
- egu:105045139

- Up regulated genes

c133817\_g1(0.83679)

- egu:105054164

- Up regulated genes

c167245\_g1(1.5987)
- egu:105033970

- Up regulated genes

c165623\_g2(1.1898)
- egu:105045139

- Up regulated genes

c133817\_g1(0.83679)

- egu:105054164

- Up regulated genes

c167245\_g1(1.5987)
- egu:105033970

- Up regulated genes

c165623\_g2(1.1898)
- egu:105045139

- Up regulated genes

c133817\_g1(0.83679)

- egu:105054164

- Up regulated genes

c167245\_g1(1.5987)
- egu:105033970

- Up regulated genes

c165623\_g2(1.1898)
- egu:105045139

- Up regulated genes

c133817\_g1(0.83679)

- egu:105054164

- Up regulated genes

c167245\_g1(1.5987)
- egu:105033970

- Up regulated genes

c165623\_g2(1.1898)
- egu:105045139

- Up regulated genes

c133817\_g1(0.83679)

- egu:105054164

- Up regulated genes

c167245\_g1(1.5987)
- egu:105033970

- Up regulated genes

c165623\_g2(1.1898)
- egu:105045139

- Up regulated genes

c133817\_g1(0.83679)

- egu:105055883

- Up regulated genes

c152294\_g1(0.45786)

- egu:105034997

- Up regulated genes

c121798\_g1(0.99412)

- egu:105034997

- Up regulated genes

c121798\_g1(0.99412)

- egu:105034997

- Up regulated genes

c121798\_g1(0.99412)

- egu:105034997

- Up regulated genes

c121798\_g1(0.99412)

- egu:105034997

- Up regulated genes

c121798\_g1(0.99412)

- egu:105034997

- Up regulated genes

c121798\_g1(0.99412)

- egu:105034997

- Up regulated genes

c121798\_g1(0.99412)

- egu:105034997

- Up regulated genes

c121798\_g1(0.99412)

- egu:105034997

- Up regulated genes

c121798\_g1(0.99412)

- egu:105034997

- Up regulated genes

c121798\_g1(0.99412)

- egu:105034997

- Up regulated genes

c121798\_g1(0.99412)

- egu:105052944

- Up regulated genes

c137525\_g1(1.6695)

- egu:105052944

- Up regulated genes

c137525\_g1(1.6695)

- egu:105052944

- Up regulated genes

c137525\_g1(1.6695)

- egu:105052944

- Up regulated genes

c137525\_g1(1.6695)

- egu:105052944

- Up regulated genes

c137525\_g1(1.6695)

- egu:105052944

- Up regulated genes

c137525\_g1(1.6695)

- egu:105052944

- Up regulated genes

c137525\_g1(1.6695)

- egu:105052944

- Up regulated genes

c137525\_g1(1.6695)

- egu:105052944

- Up regulated genes

c137525\_g1(1.6695)

- egu:105052944

- Up regulated genes

c137525\_g1(1.6695)

- egu:105052944

- Up regulated genes

c137525\_g1(1.6695)

- egu:105055939

- Up regulated genes

c156970\_g1(1.2935)

- egu:105058186

- Up regulated genes

c155751\_g1(0.98173)

- egu:105058186

- Up regulated genes

c155751\_g1(0.98173)

- egu:105058186

- Up regulated genes

c155751\_g1(0.98173)

- egu:105058186

- Up regulated genes

c155751\_g1(0.98173)

- egu:105058186

- Up regulated genes

c155751\_g1(0.98173)

- egu:105058186

- Up regulated genes

c155751\_g1(0.98173)

- egu:105058186

- Up regulated genes

c155751\_g1(0.98173)

- egu:105058186

- Up regulated genes

c155751\_g1(0.98173)

- egu:105058186

- Up regulated genes

c155751\_g1(0.98173)

- egu:105058186

- Up regulated genes

c155751\_g1(0.98173)

- egu:105058186

- Up regulated genes

c155751\_g1(0.98173)

- egu:105058186

- Up regulated genes

c155751\_g1(0.98173)

- egu:105058186

- Up regulated genes

c155751\_g1(0.98173)

- egu:105058186

- Up regulated genes

c155751\_g1(0.98173)

- egu:105058186

- Up regulated genes

c155751\_g1(0.98173)

- egu:105058186

- Up regulated genes

c155751\_g1(0.98173)

- egu:105058186

- Up regulated genes

c155751\_g1(0.98173)

- egu:105058186

- Up regulated genes

c155751\_g1(0.98173)

- egu:105058186

- Up regulated genes

c155751\_g1(0.98173)

- egu:105058186

- Up regulated genes

c155751\_g1(0.98173)

- egu:105058186

- Up regulated genes

c155751\_g1(0.98173)

- egu:105058186

- Up regulated genes

c155751\_g1(0.98173)

- egu:105058186

- Up regulated genes

c155751\_g1(0.98173)

- egu:105058186

- Up regulated genes

c155751\_g1(0.98173)

- egu:105058186

- Up regulated genes

c155751\_g1(0.98173)

- egu:105058186

- Up regulated genes

c155751\_g1(0.98173)

- egu:105058186

- Up regulated genes

c155751\_g1(0.98173)

- egu:105058186

- Up regulated genes

c155751\_g1(0.98173)

- egu:105058186

- Up regulated genes

c155751\_g1(0.98173)

- egu:105058186

- Up regulated genes

c155751\_g1(0.98173)

- egu:105058186

- Up regulated genes

c155751\_g1(0.98173)

- egu:105058186

- Up regulated genes

c155751\_g1(0.98173)

- egu:105058186

- Up regulated genes

c155751\_g1(0.98173)

- egu:105058186

- Up regulated genes

c155751\_g1(0.98173)

- egu:105058186

- Up regulated genes

c155751\_g1(0.98173)

- egu:105058186

- Up regulated genes

c155751\_g1(0.98173)

- egu:105058186

- Up regulated genes

c155751\_g1(0.98173)

- egu:105058186

- Up regulated genes

c155751\_g1(0.98173)

- egu:105058186

- Up regulated genes

c155751\_g1(0.98173)

- egu:105058186

- Up regulated genes

c155751\_g1(0.98173)

- egu:105058186

- Up regulated genes

c155751\_g1(0.98173)

- egu:105058186

- Up regulated genes

c155751\_g1(0.98173)

- egu:105041902

- Up regulated genes

c151510\_g1(2.0621)

- egu:105041902

- Up regulated genes

c151510\_g1(2.0621)

- egu:105041902

- Up regulated genes

c151510\_g1(2.0621)

- egu:105041902

- Up regulated genes

c151510\_g1(2.0621)

- egu:105041902

- Up regulated genes

c151510\_g1(2.0621)

- egu:105041902

- Up regulated genes

c151510\_g1(2.0621)

- egu:105041902

- Up regulated genes

c151510\_g1(2.0621)

- egu:105041902

- Up regulated genes

c151510\_g1(2.0621)

- egu:105041902

- Up regulated genes

c151510\_g1(2.0621)

- egu:105041902

- Up regulated genes

c151510\_g1(2.0621)

- egu:105041902

- Up regulated genes

c151510\_g1(2.0621)

- egu:105058186

- Up regulated genes

c155751\_g1(0.98173)

- egu:105045148

- Up regulated genes

c159016\_g1(2.0401)

- egu:105038419

- Up regulated genes

c116327\_g1(1.1367)

- egu:105038419

- Up regulated genes

c116327\_g1(1.1367)

- egu:105038419

- Up regulated genes

c116327\_g1(1.1367)

- egu:105038419

- Up regulated genes

c116327\_g1(1.1367)

- egu:105038419

- Up regulated genes

c116327\_g1(1.1367)

- egu:105038419

- Up regulated genes

c116327\_g1(1.1367)

- egu:105038419

- Up regulated genes

c116327\_g1(1.1367)

- egu:105038419

- Up regulated genes

c116327\_g1(1.1367)

- egu:105038419

- Up regulated genes

c116327\_g1(1.1367)

- egu:105038419

- Up regulated genes

c116327\_g1(1.1367)

- egu:105038419

- Up regulated genes

c116327\_g1(1.1367)

- egu:105033631

- Up regulated genes

c153848\_g1(1.4453)

- egu:105039187

- Up regulated genes

c157258\_g1(1.2643)

- egu:105034598

- Up regulated genes

c154095\_g1(1.2755)

- egu:105036364

- Up regulated genes

c155146\_g1(7.5424)
- egu:105050962

- Up regulated genes

c174513\_g3(4.5905) c174513\_g1(5.3633) c146896\_g1(6.4969) c146896\_g3(7.3843) c151401\_g1(4.5093)
- egu:105035716

- Up regulated genes

c169857\_g1(2.9204)

- egu:105036364

- Up regulated genes

c155146\_g1(7.5424)
- egu:105050962

- Up regulated genes

c174513\_g3(4.5905) c174513\_g1(5.3633) c146896\_g1(6.4969) c146896\_g3(7.3843) c151401\_g1(4.5093)
- egu:105035716

- Up regulated genes

c169857\_g1(2.9204)

- egu:105036364

- Up regulated genes

c155146\_g1(7.5424)
- egu:105050962

- Up regulated genes

c174513\_g3(4.5905) c174513\_g1(5.3633) c146896\_g1(6.4969) c146896\_g3(7.3843) c151401\_g1(4.5093)
- egu:105035716

- Up regulated genes

c169857\_g1(2.9204)

- egu:105036364

- Up regulated genes

c155146\_g1(7.5424)
- egu:105050962

- Up regulated genes

c174513\_g3(4.5905) c174513\_g1(5.3633) c146896\_g1(6.4969) c146896\_g3(7.3843) c151401\_g1(4.5093)
- egu:105035716

- Up regulated genes

c169857\_g1(2.9204)

- egu:105036364

- Up regulated genes

c155146\_g1(7.5424)
- egu:105050962

- Up regulated genes

c174513\_g3(4.5905) c174513\_g1(5.3633) c146896\_g1(6.4969) c146896\_g3(7.3843) c151401\_g1(4.5093)
- egu:105035716

- Up regulated genes

c169857\_g1(2.9204)

- egu:105036364

- Up regulated genes

c155146\_g1(7.5424)
- egu:105050962

- Up regulated genes

c174513\_g3(4.5905) c174513\_g1(5.3633) c146896\_g1(6.4969) c146896\_g3(7.3843) c151401\_g1(4.5093)
- egu:105035716

- Up regulated genes

c169857\_g1(2.9204)

- egu:105036364

- Up regulated genes

c155146\_g1(7.5424)
- egu:105050962

- Up regulated genes

c174513\_g3(4.5905) c174513\_g1(5.3633) c146896\_g1(6.4969) c146896\_g3(7.3843) c151401\_g1(4.5093)
- egu:105035716

- Up regulated genes

c169857\_g1(2.9204)

- egu:105036364

- Up regulated genes

c155146\_g1(7.5424)
- egu:105050962

- Up regulated genes

c174513\_g3(4.5905) c174513\_g1(5.3633) c146896\_g1(6.4969) c146896\_g3(7.3843) c151401\_g1(4.5093)
- egu:105035716

- Up regulated genes

c169857\_g1(2.9204)

- egu:105036364

- Up regulated genes

c155146\_g1(7.5424)
- egu:105050962

- Up regulated genes

c174513\_g3(4.5905) c174513\_g1(5.3633) c146896\_g1(6.4969) c146896\_g3(7.3843) c151401\_g1(4.5093)
- egu:105035716

- Up regulated genes

c169857\_g1(2.9204)

- egu:105036364

- Up regulated genes

c155146\_g1(7.5424)
- egu:105050962

- Up regulated genes

c174513\_g3(4.5905) c174513\_g1(5.3633) c146896\_g1(6.4969) c146896\_g3(7.3843) c151401\_g1(4.5093)
- egu:105035716

- Up regulated genes

c169857\_g1(2.9204)

- egu:105036364

- Up regulated genes

c155146\_g1(7.5424)
- egu:105050962

- Up regulated genes

c174513\_g3(4.5905) c174513\_g1(5.3633) c146896\_g1(6.4969) c146896\_g3(7.3843) c151401\_g1(4.5093)
- egu:105035716

- Up regulated genes

c169857\_g1(2.9204)

- egu:105050474

- Up regulated genes

c170886\_g3(0.98865)

- egu:105050474

- Up regulated genes

c170886\_g3(0.98865)

- egu:105050474

- Up regulated genes

c170886\_g3(0.98865)

- egu:105050474

- Up regulated genes

c170886\_g3(0.98865)

- egu:105050474

- Up regulated genes

c170886\_g3(0.98865)

- egu:105050474

- Up regulated genes

c170886\_g3(0.98865)

- egu:105050474

- Up regulated genes

c170886\_g3(0.98865)

- egu:105050474

- Up regulated genes

c170886\_g3(0.98865)

- egu:105050474

- Up regulated genes

c170886\_g3(0.98865)

- egu:105050474

- Up regulated genes

c170886\_g3(0.98865)

- egu:105050474

- Up regulated genes

c170886\_g3(0.98865)

- egu:105050474

- Up regulated genes

c170886\_g3(0.98865)

- egu:105050474

- Up regulated genes

c170886\_g3(0.98865)

- egu:105050474

- Up regulated genes

c170886\_g3(0.98865)

- egu:105050474

- Up regulated genes

c170886\_g3(0.98865)

- egu:105050474

- Up regulated genes

c170886\_g3(0.98865)

- egu:105050474

- Up regulated genes

c170886\_g3(0.98865)

- egu:105050474

- Up regulated genes

c170886\_g3(0.98865)

- egu:105050474

- Up regulated genes

c170886\_g3(0.98865)

- egu:105050474

- Up regulated genes

c170886\_g3(0.98865)

- egu:105050474

- Up regulated genes

c170886\_g3(0.98865)

- egu:105050474

- Up regulated genes

c170886\_g3(0.98865)

- egu:105052990

- Up regulated genes

c117443\_g1(0.9188)
- egu:105060729

- Up regulated genes

c149166\_g1(1.6337)

- egu:105052990

- Up regulated genes

c117443\_g1(0.9188)
- egu:105060729

- Up regulated genes

c149166\_g1(1.6337)

- egu:105052990

- Up regulated genes

c117443\_g1(0.9188)
- egu:105060729

- Up regulated genes

c149166\_g1(1.6337)

- egu:105052990

- Up regulated genes

c117443\_g1(0.9188)
- egu:105060729

- Up regulated genes

c149166\_g1(1.6337)

- egu:105052990

- Up regulated genes

c117443\_g1(0.9188)
- egu:105060729

- Up regulated genes

c149166\_g1(1.6337)

- egu:105052990

- Up regulated genes

c117443\_g1(0.9188)
- egu:105060729

- Up regulated genes

c149166\_g1(1.6337)

- egu:105052990

- Up regulated genes

c117443\_g1(0.9188)
- egu:105060729

- Up regulated genes

c149166\_g1(1.6337)

- egu:105052990

- Up regulated genes

c117443\_g1(0.9188)
- egu:105060729

- Up regulated genes

c149166\_g1(1.6337)

- egu:105052990

- Up regulated genes

c117443\_g1(0.9188)
- egu:105060729

- Up regulated genes

c149166\_g1(1.6337)

- egu:105052990

- Up regulated genes

c117443\_g1(0.9188)
- egu:105060729

- Up regulated genes

c149166\_g1(1.6337)

- egu:105052990

- Up regulated genes

c117443\_g1(0.9188)
- egu:105060729

- Up regulated genes

c149166\_g1(1.6337)

- egu:105052990

- Up regulated genes

c117443\_g1(0.9188)
- egu:105060729

- Up regulated genes

c149166\_g1(1.6337)

- egu:105052990

- Up regulated genes

c117443\_g1(0.9188)
- egu:105060729

- Up regulated genes

c149166\_g1(1.6337)

- egu:105052990

- Up regulated genes

c117443\_g1(0.9188)
- egu:105060729

- Up regulated genes

c149166\_g1(1.6337)

- egu:105052990

- Up regulated genes

c117443\_g1(0.9188)
- egu:105060729

- Up regulated genes

c149166\_g1(1.6337)

- egu:105052990

- Up regulated genes

c117443\_g1(0.9188)
- egu:105060729

- Up regulated genes

c149166\_g1(1.6337)

- egu:105052990

- Up regulated genes

c117443\_g1(0.9188)
- egu:105060729

- Up regulated genes

c149166\_g1(1.6337)

- egu:105052990

- Up regulated genes

c117443\_g1(0.9188)
- egu:105060729

- Up regulated genes

c149166\_g1(1.6337)

- egu:105052990

- Up regulated genes

c117443\_g1(0.9188)
- egu:105060729

- Up regulated genes

c149166\_g1(1.6337)

- egu:105052990

- Up regulated genes

c117443\_g1(0.9188)
- egu:105060729

- Up regulated genes

c149166\_g1(1.6337)

- egu:105045855

- Up regulated genes

c172556\_g1(1.8748)
- egu:105059487

- Up regulated genes

c162039\_g1(0.48153)

- egu:105058982

- Up regulated genes

c156756\_g2(2.2972)
- egu:105057280

- Up regulated genes

c172074\_g1(2.793)
- egu:105038179

- Up regulated genes

c161769\_g1(0.81464)
- egu:105042489

- Up regulated genes

c156756\_g1(2.052)
- egu:105035292

- Up regulated genes

c188298\_g1(2.0535)
- egu:105053882

- Up regulated genes

c158821\_g1(0.52215)

- egu:105053561

- Up regulated genes

c163051\_g1(1.3984)

- egu:105042090

- Up regulated genes

c148031\_g1(0.6165)

- egu:105038209

- Up regulated genes

c119816\_g1(1.47)
- egu:105052340

- Up regulated genes

c175256\_g1(1.4216) c151470\_g2(1.2691)
- egu:105046041

- Up regulated genes

c157181\_g1(1.4428) c151470\_g3(1.9388)
- egu:105056873

- Up regulated genes

c156718\_g1(0.83881)

- egu:105059872

- Up regulated genes

c157432\_g1(0.46067)

- egu:105059882

- Up regulated genes

c145285\_g1(2.0457)
- egu:105051363

- Up regulated genes

c105074\_g2(1.0862)

- egu:105039431

- Up regulated genes

c170590\_g5(2.0592) c170590\_g8(1.9734)
- egu:105060774

- Up regulated genes

c167493\_g1(1.2221)

- egu:105059350

- Up regulated genes

c155459\_g1(0.63442)

- egu:105032435

- Up regulated genes

c133447\_g2(0.58858)

- egu:105059450

- Up regulated genes

c170991\_g2(0.56857)

- egu:105059450

- Up regulated genes

c170991\_g2(0.56857)

- egu:105059450

- Up regulated genes

c170991\_g2(0.56857)

- egu:105059450

- Up regulated genes

c170991\_g2(0.56857)

- egu:105059450

- Up regulated genes

c170991\_g2(0.56857)

- egu:105059450

- Up regulated genes

c170991\_g2(0.56857)

- egu:105059450

- Up regulated genes

c170991\_g2(0.56857)

- egu:105059450

- Up regulated genes

c170991\_g2(0.56857)

- egu:105059450

- Up regulated genes

c170991\_g2(0.56857)

- egu:105059450

- Up regulated genes

c170991\_g2(0.56857)

- egu:105059450

- Up regulated genes

c170991\_g2(0.56857)

- egu:105059450

- Up regulated genes

c170991\_g2(0.56857)

- egu:105059450

- Up regulated genes

c170991\_g2(0.56857)

- egu:105059450

- Up regulated genes

c170991\_g2(0.56857)

- egu:105059450

- Up regulated genes

c170991\_g2(0.56857)

- egu:105059450

- Up regulated genes

c170991\_g2(0.56857)

- egu:105059450

- Up regulated genes

c170991\_g2(0.56857)

- egu:105059450

- Up regulated genes

c170991\_g2(0.56857)

- egu:105059450

- Up regulated genes

c170991\_g2(0.56857)

- egu:105059450

- Up regulated genes

c170991\_g2(0.56857)

- egu:105059450

- Up regulated genes

c170991\_g2(0.56857)

- egu:105059450

- Up regulated genes

c170991\_g2(0.56857)

- egu:105060382

- Up regulated genes

c162772\_g1(0.74368)

- egu:105060382

- Up regulated genes

c162772\_g1(0.74368)

- egu:105060382

- Up regulated genes

c162772\_g1(0.74368)

- egu:105060382

- Up regulated genes

c162772\_g1(0.74368)

- egu:105060382

- Up regulated genes

c162772\_g1(0.74368)

- egu:105060382

- Up regulated genes

c162772\_g1(0.74368)

- egu:105060382

- Up regulated genes

c162772\_g1(0.74368)

- egu:105060382

- Up regulated genes

c162772\_g1(0.74368)

- egu:105060382

- Up regulated genes

c162772\_g1(0.74368)

- egu:105060382

- Up regulated genes

c162772\_g1(0.74368)

- egu:105060382

- Up regulated genes

c162772\_g1(0.74368)

- egu:105060382

- Up regulated genes

c162772\_g1(0.74368)

- egu:105060382

- Up regulated genes

c162772\_g1(0.74368)

- egu:105060382

- Up regulated genes

c162772\_g1(0.74368)

- egu:105060382

- Up regulated genes

c162772\_g1(0.74368)

- egu:105060382

- Up regulated genes

c162772\_g1(0.74368)

- egu:105060382

- Up regulated genes

c162772\_g1(0.74368)

- egu:105060382

- Up regulated genes

c162772\_g1(0.74368)

- egu:105060382

- Up regulated genes

c162772\_g1(0.74368)

- egu:105060382

- Up regulated genes

c162772\_g1(0.74368)

- egu:105060382

- Up regulated genes

c162772\_g1(0.74368)

- egu:105060382

- Up regulated genes

c162772\_g1(0.74368)

- egu:105059450

- Up regulated genes

c170991\_g2(0.56857)

- egu:105059450

- Up regulated genes

c170991\_g2(0.56857)

- egu:105059450

- Up regulated genes

c170991\_g2(0.56857)

- egu:105059450

- Up regulated genes

c170991\_g2(0.56857)

- egu:105059450

- Up regulated genes

c170991\_g2(0.56857)

- egu:105059450

- Up regulated genes

c170991\_g2(0.56857)

- egu:105059450

- Up regulated genes

c170991\_g2(0.56857)

- egu:105059450

- Up regulated genes

c170991\_g2(0.56857)

- egu:105059450

- Up regulated genes

c170991\_g2(0.56857)

- egu:105059450

- Up regulated genes

c170991\_g2(0.56857)

- egu:105059450

- Up regulated genes

c170991\_g2(0.56857)

- egu:105059450

- Up regulated genes

c170991\_g2(0.56857)

- egu:105059450

- Up regulated genes

c170991\_g2(0.56857)

- egu:105059450

- Up regulated genes

c170991\_g2(0.56857)

- egu:105059450

- Up regulated genes

c170991\_g2(0.56857)

- egu:105059450

- Up regulated genes

c170991\_g2(0.56857)

- egu:105059450

- Up regulated genes

c170991\_g2(0.56857)

- egu:105059450

- Up regulated genes

c170991\_g2(0.56857)

- egu:105059450

- Up regulated genes

c170991\_g2(0.56857)

- egu:105059450

- Up regulated genes

c170991\_g2(0.56857)

- egu:105059450

- Up regulated genes

c170991\_g2(0.56857)

- egu:105059450

- Up regulated genes

c170991\_g2(0.56857)

- egu:105059450

- Up regulated genes

c170991\_g2(0.56857)

- egu:105059450

- Up regulated genes

c170991\_g2(0.56857)

- egu:105059450

- Up regulated genes

c170991\_g2(0.56857)

- egu:105059450

- Up regulated genes

c170991\_g2(0.56857)

- egu:105059450

- Up regulated genes

c170991\_g2(0.56857)

- egu:105059450

- Up regulated genes

c170991\_g2(0.56857)

- egu:105059450

- Up regulated genes

c170991\_g2(0.56857)

- egu:105059450

- Up regulated genes

c170991\_g2(0.56857)

- egu:105059450

- Up regulated genes

c170991\_g2(0.56857)

- egu:105059450

- Up regulated genes

c170991\_g2(0.56857)

- egu:105047057

- Up regulated genes

c159520\_g1(1.375)

- egu:105060182

- Up regulated genes

c164239\_g2(1.4555)

- egu:105037948

- Up regulated genes

c168406\_g1(0.53391)

- egu:105037948

- Up regulated genes

c168406\_g1(0.53391)

- egu:105060274

- Up regulated genes

c163681\_g1(0.94659)

- egu:105060274

- Up regulated genes

c163681\_g1(0.94659)

- egu:105035781

- Up regulated genes

c168304\_g1(0.73196)
- egu:105054501

- Up regulated genes

c148234\_g1(5.8458)
- egu:105055673

- Up regulated genes

c166080\_g1(4.7318) c168304\_g3(5.2016) c168304\_g2(3.9853)

- egu:105039619

- Up regulated genes

c162887\_g1(0.93581)
- egu:105053813

- Up regulated genes

c134164\_g1(5.8972)

- egu:105050729

- Up regulated genes

c165035\_g1(1.3319)

- egu:105039619

- Up regulated genes

c162887\_g1(0.93581)
- egu:105053813

- Up regulated genes

c134164\_g1(5.8972)

- egu:105050729

- Up regulated genes

c165035\_g1(1.3319)

- egu:105037657

- Up regulated genes

c161779\_g15(2.7014) c170056\_g1(4.6538)

- egu:105051975

- Up regulated genes

c169830\_g1(1.0742)
- egu:105056224

- Up regulated genes

c140986\_g1(2.4063)
- egu:105038037

- Up regulated genes

c172938\_g7(3.4541)

- egu:105045995

- Up regulated genes

c165806\_g1(3.5125)

- egu:105045199

- Up regulated genes

c170749\_g4(1.3881) c170749\_g3(0.99004)
- egu:105042391

- Up regulated genes

c172256\_g4(3.2203) c155636\_g1(3.3411) c155636\_g2(3.0714)

- egu:105039619

- Up regulated genes

c162887\_g1(0.93581)
- egu:105053813

- Up regulated genes

c134164\_g1(5.8972)

- egu:105050729

- Up regulated genes

c165035\_g1(1.3319)

- egu:105037657

- Up regulated genes

c161779\_g15(2.7014) c170056\_g1(4.6538)

- egu:105039619

- Up regulated genes

c162887\_g1(0.93581)
- egu:105053813

- Up regulated genes

c134164\_g1(5.8972)

- egu:105050729

- Up regulated genes

c165035\_g1(1.3319)

- egu:105037657

- Up regulated genes

c161779\_g15(2.7014) c170056\_g1(4.6538)

- egu:105051975

- Up regulated genes

c169830\_g1(1.0742)
- egu:105056224

- Up regulated genes

c140986\_g1(2.4063)
- egu:105038037

- Up regulated genes

c172938\_g7(3.4541)

- egu:105039619

- Up regulated genes

c162887\_g1(0.93581)
- egu:105053813

- Up regulated genes

c134164\_g1(5.8972)

- egu:105050729

- Up regulated genes

c165035\_g1(1.3319)

- egu:105037657

- Up regulated genes

c161779\_g15(2.7014) c170056\_g1(4.6538)

- egu:105039619

- Up regulated genes

c162887\_g1(0.93581)
- egu:105053813

- Up regulated genes

c134164\_g1(5.8972)

- egu:105050729

- Up regulated genes

c165035\_g1(1.3319)

- egu:105037657

- Up regulated genes

c161779\_g15(2.7014) c170056\_g1(4.6538)

- egu:105051975

- Up regulated genes

c169830\_g1(1.0742)
- egu:105056224

- Up regulated genes

c140986\_g1(2.4063)
- egu:105038037

- Up regulated genes

c172938\_g7(3.4541)

- egu:105036364

- Up regulated genes

c155146\_g1(7.5424)
- egu:105050962

- Up regulated genes

c174513\_g3(4.5905) c174513\_g1(5.3633) c146896\_g1(6.4969) c146896\_g3(7.3843) c151401\_g1(4.5093)
- egu:105035716

- Up regulated genes

c169857\_g1(2.9204)

- egu:105036364

- Up regulated genes

c155146\_g1(7.5424)
- egu:105050962

- Up regulated genes

c174513\_g3(4.5905) c174513\_g1(5.3633) c146896\_g1(6.4969) c146896\_g3(7.3843) c151401\_g1(4.5093)
- egu:105035716

- Up regulated genes

c169857\_g1(2.9204)

- egu:105036364

- Up regulated genes

c155146\_g1(7.5424)
- egu:105050962

- Up regulated genes

c174513\_g3(4.5905) c174513\_g1(5.3633) c146896\_g1(6.4969) c146896\_g3(7.3843) c151401\_g1(4.5093)
- egu:105035716

- Up regulated genes

c169857\_g1(2.9204)

- egu:105036364

- Up regulated genes

c155146\_g1(7.5424)
- egu:105050962

- Up regulated genes

c174513\_g3(4.5905) c174513\_g1(5.3633) c146896\_g1(6.4969) c146896\_g3(7.3843) c151401\_g1(4.5093)
- egu:105035716

- Up regulated genes

c169857\_g1(2.9204)

- egu:105036364

- Up regulated genes

c155146\_g1(7.5424)
- egu:105050962

- Up regulated genes

c174513\_g3(4.5905) c174513\_g1(5.3633) c146896\_g1(6.4969) c146896\_g3(7.3843) c151401\_g1(4.5093)
- egu:105035716

- Up regulated genes

c169857\_g1(2.9204)

- egu:105036364

- Up regulated genes

c155146\_g1(7.5424)
- egu:105050962

- Up regulated genes

c174513\_g3(4.5905) c174513\_g1(5.3633) c146896\_g1(6.4969) c146896\_g3(7.3843) c151401\_g1(4.5093)
- egu:105035716

- Up regulated genes

c169857\_g1(2.9204)

- egu:105036364

- Up regulated genes

c155146\_g1(7.5424)
- egu:105050962

- Up regulated genes

c174513\_g3(4.5905) c174513\_g1(5.3633) c146896\_g1(6.4969) c146896\_g3(7.3843) c151401\_g1(4.5093)
- egu:105035716

- Up regulated genes

c169857\_g1(2.9204)

- egu:105036364

- Up regulated genes

c155146\_g1(7.5424)
- egu:105050962

- Up regulated genes

c174513\_g3(4.5905) c174513\_g1(5.3633) c146896\_g1(6.4969) c146896\_g3(7.3843) c151401\_g1(4.5093)
- egu:105035716

- Up regulated genes

c169857\_g1(2.9204)

- egu:105036364

- Up regulated genes

c155146\_g1(7.5424)
- egu:105050962

- Up regulated genes

c174513\_g3(4.5905) c174513\_g1(5.3633) c146896\_g1(6.4969) c146896\_g3(7.3843) c151401\_g1(4.5093)
- egu:105035716

- Up regulated genes

c169857\_g1(2.9204)

- egu:105036364

- Up regulated genes

c155146\_g1(7.5424)
- egu:105050962

- Up regulated genes

c174513\_g3(4.5905) c174513\_g1(5.3633) c146896\_g1(6.4969) c146896\_g3(7.3843) c151401\_g1(4.5093)
- egu:105035716

- Up regulated genes

c169857\_g1(2.9204)

- egu:105036364

- Up regulated genes

c155146\_g1(7.5424)
- egu:105050962

- Up regulated genes

c174513\_g3(4.5905) c174513\_g1(5.3633) c146896\_g1(6.4969) c146896\_g3(7.3843) c151401\_g1(4.5093)
- egu:105035716

- Up regulated genes

c169857\_g1(2.9204)

- egu:105058071

- Up regulated genes

c154616\_g1(6.2597)

- egu:105054663

- Up regulated genes

c166729\_g1(5.4331)

- egu:105058071

- Up regulated genes

c154616\_g1(6.2597)

- egu:105054663

- Up regulated genes

c166729\_g1(5.4331)

- egu:105054663

- Up regulated genes

c166729\_g1(5.4331)

- egu:105058071

- Up regulated genes

c154616\_g1(6.2597)

- egu:105058071

- Up regulated genes

c154616\_g1(6.2597)

- egu:105035842

- Up regulated genes

c155053\_g1(Inf)

- egu:105035842

- Up regulated genes

c155053\_g1(Inf)

- egu:105035842

- Up regulated genes

c155053\_g1(Inf)

- egu:105035842

- Up regulated genes

c155053\_g1(Inf)

- egu:105048962

- Up regulated genes

c172129\_g1(4.8855)

- egu:105055420

- Up regulated genes

c167137\_g1(2.6523) c168951\_g1(1.1729)
- egu:105044125

- Up regulated genes

c167006\_g1(0.74568) c170271\_g1(1.7042)

- egu:105055420

- Up regulated genes

c167137\_g1(2.6523) c168951\_g1(1.1729)
- egu:105044125

- Up regulated genes

c167006\_g1(0.74568) c170271\_g1(1.7042)

- egu:105055420

- Up regulated genes

c167137\_g1(2.6523) c168951\_g1(1.1729)
- egu:105044125

- Up regulated genes

c167006\_g1(0.74568) c170271\_g1(1.7042)

- egu:105055420

- Up regulated genes

c167137\_g1(2.6523) c168951\_g1(1.1729)
- egu:105044125

- Up regulated genes

c167006\_g1(0.74568) c170271\_g1(1.7042)

- egu:105055420

- Up regulated genes

c167137\_g1(2.6523) c168951\_g1(1.1729)
- egu:105044125

- Up regulated genes

c167006\_g1(0.74568) c170271\_g1(1.7042)

- egu:105055420

- Up regulated genes

c167137\_g1(2.6523) c168951\_g1(1.1729)
- egu:105044125

- Up regulated genes

c167006\_g1(0.74568) c170271\_g1(1.7042)

- egu:105055420

- Up regulated genes

c167137\_g1(2.6523) c168951\_g1(1.1729)
- egu:105044125

- Up regulated genes

c167006\_g1(0.74568) c170271\_g1(1.7042)

- egu:105055420

- Up regulated genes

c167137\_g1(2.6523) c168951\_g1(1.1729)
- egu:105044125

- Up regulated genes

c167006\_g1(0.74568) c170271\_g1(1.7042)

- egu:105055420

- Up regulated genes

c167137\_g1(2.6523) c168951\_g1(1.1729)
- egu:105044125

- Up regulated genes

c167006\_g1(0.74568) c170271\_g1(1.7042)

- egu:105055420

- Up regulated genes

c167137\_g1(2.6523) c168951\_g1(1.1729)
- egu:105044125

- Up regulated genes

c167006\_g1(0.74568) c170271\_g1(1.7042)

- egu:105055420

- Up regulated genes

c167137\_g1(2.6523) c168951\_g1(1.1729)
- egu:105044125

- Up regulated genes

c167006\_g1(0.74568) c170271\_g1(1.7042)

- egu:105055420

- Up regulated genes

c167137\_g1(2.6523) c168951\_g1(1.1729)
- egu:105044125

- Up regulated genes

c167006\_g1(0.74568) c170271\_g1(1.7042)

- egu:105055420

- Up regulated genes

c167137\_g1(2.6523) c168951\_g1(1.1729)
- egu:105044125

- Up regulated genes

c167006\_g1(0.74568) c170271\_g1(1.7042)

- egu:105035842

- Up regulated genes

c155053\_g1(Inf)

- egu:105035984

- Up regulated genes

c167553\_g1(2.6788)

- egu:105034095

- Up regulated genes

c146169\_g1(0.52161)

- egu:105034095

- Up regulated genes

c146169\_g1(0.52161)

- egu:105034095

- Up regulated genes

c146169\_g1(0.52161)

- egu:105034095

- Up regulated genes

c146169\_g1(0.52161)

- egu:105034095

- Up regulated genes

c146169\_g1(0.52161)

- egu:105034095

- Up regulated genes

c146169\_g1(0.52161)

- egu:105034095

- Up regulated genes

c146169\_g1(0.52161)

- egu:105034095

- Up regulated genes

c146169\_g1(0.52161)

- egu:105034095

- Up regulated genes

c146169\_g1(0.52161)

- egu:105034095

- Up regulated genes

c146169\_g1(0.52161)

- egu:105034095

- Up regulated genes

c146169\_g1(0.52161)

- egu:105034095

- Up regulated genes

c146169\_g1(0.52161)

- egu:105034095

- Up regulated genes

c146169\_g1(0.52161)

- egu:105034095

- Up regulated genes

c146169\_g1(0.52161)

- egu:105034095

- Up regulated genes

c146169\_g1(0.52161)

- egu:105034095

- Up regulated genes

c146169\_g1(0.52161)

- egu:105034095

- Up regulated genes

c146169\_g1(0.52161)

- egu:105034095

- Up regulated genes

c146169\_g1(0.52161)

- egu:105034095

- Up regulated genes

c146169\_g1(0.52161)

- egu:105034095

- Up regulated genes

c146169\_g1(0.52161)

- egu:105034095

- Up regulated genes

c146169\_g1(0.52161)

- egu:105034095

- Up regulated genes

c146169\_g1(0.52161)

- egu:105053370

- Up regulated genes

c151855\_g2(2.7437)

- egu:105055939

- Up regulated genes

c156970\_g1(1.2935)

- egu:105055939

- Up regulated genes

c156970\_g1(1.2935)

- egu:105036591

- Up regulated genes

c168403\_g1(1.1921)

- egu:105042090

- Up regulated genes

c148031\_g1(0.6165)

- egu:105035984

- Up regulated genes

c167553\_g1(2.6788)

- egu:105035499

- Up regulated genes

c121963\_g1(0.56446)

- egu:105058982

- Up regulated genes

c156756\_g2(2.2972)
- egu:105057280

- Up regulated genes

c172074\_g1(2.793)
- egu:105038179

- Up regulated genes

c161769\_g1(0.81464)
- egu:105042489

- Up regulated genes

c156756\_g1(2.052)
- egu:105035292

- Up regulated genes

c188298\_g1(2.0535)
- egu:105053882

- Up regulated genes

c158821\_g1(0.52215)

- egu:105058232

- Up regulated genes

c146719\_g1(2.8331)

- egu:105058232

- Up regulated genes

c146719\_g1(2.8331)

- egu:105058232

- Up regulated genes

c146719\_g1(2.8331)

- egu:105058232

- Up regulated genes

c146719\_g1(2.8331)

- egu:105058232

- Up regulated genes

c146719\_g1(2.8331)

- egu:105058232

- Up regulated genes

c146719\_g1(2.8331)

- egu:105058232

- Up regulated genes

c146719\_g1(2.8331)

- egu:105058232

- Up regulated genes

c146719\_g1(2.8331)

- egu:105058232

- Up regulated genes

c146719\_g1(2.8331)

- egu:105058232

- Up regulated genes

c146719\_g1(2.8331)

- egu:105058232

- Up regulated genes

c146719\_g1(2.8331)

- egu:105040573

- Up regulated genes

c141199\_g1(1.1797)

- egu:105040573

- Up regulated genes

c141199\_g1(1.1797)

- egu:105057764

- Up regulated genes

c156235\_g1(1.2114)

- egu:105058186

- Up regulated genes

c155751\_g1(0.98173)

- egu:105040573

- Up regulated genes

c141199\_g1(1.1797)

- egu:105040573

- Up regulated genes

c141199\_g1(1.1797)

- egu:105038882

- Up regulated genes

c151973\_g1(0.59053)

- egu:105053391

- Up regulated genes

c114481\_g1(1.3666) c104905\_g1(0.92874)

- egu:105053391

- Up regulated genes

c114481\_g1(1.3666) c104905\_g1(0.92874)

- egu:105058186

- Up regulated genes

c155751\_g1(0.98173)

- egu:105058543

- Up regulated genes

c145522\_g1(1.236)

- egu:105058543

- Up regulated genes

c145522\_g1(1.236)

- egu:105058543

- Up regulated genes

c145522\_g1(1.236)

- egu:105058543

- Up regulated genes

c145522\_g1(1.236)

- egu:105058543

- Up regulated genes

c145522\_g1(1.236)

- egu:105058543

- Up regulated genes

c145522\_g1(1.236)

- egu:105058543

- Up regulated genes

c145522\_g1(1.236)

- egu:105058543

- Up regulated genes

c145522\_g1(1.236)

- egu:105058543

- Up regulated genes

c145522\_g1(1.236)

- egu:105058543

- Up regulated genes

c145522\_g1(1.236)

- egu:105058543

- Up regulated genes

c145522\_g1(1.236)

- egu:105041644

- Up regulated genes

c165048\_g1(0.7123)

- egu:105041644

- Up regulated genes

c165048\_g1(0.7123)

- egu:105041644

- Up regulated genes

c165048\_g1(0.7123)

- egu:105041644

- Up regulated genes

c165048\_g1(0.7123)

- egu:105041644

- Up regulated genes

c165048\_g1(0.7123)

- egu:105041644

- Up regulated genes

c165048\_g1(0.7123)

- egu:105041644

- Up regulated genes

c165048\_g1(0.7123)

- egu:105041644

- Up regulated genes

c165048\_g1(0.7123)

- egu:105041644

- Up regulated genes

c165048\_g1(0.7123)

- egu:105041644

- Up regulated genes

c165048\_g1(0.7123)

- egu:105041644

- Up regulated genes

c165048\_g1(0.7123)

- egu:105041644

- Up regulated genes

c165048\_g1(0.7123)

- egu:105041644

- Up regulated genes

c165048\_g1(0.7123)

- egu:105041644

- Up regulated genes

c165048\_g1(0.7123)

- egu:105041644

- Up regulated genes

c165048\_g1(0.7123)

- egu:105041644

- Up regulated genes

c165048\_g1(0.7123)

- egu:105041644

- Up regulated genes

c165048\_g1(0.7123)

- egu:105041644

- Up regulated genes

c165048\_g1(0.7123)

- egu:105041644

- Up regulated genes

c165048\_g1(0.7123)

- egu:105041644

- Up regulated genes

c165048\_g1(0.7123)

- egu:105041644

- Up regulated genes

c165048\_g1(0.7123)

- egu:105052944

- Up regulated genes

c137525\_g1(1.6695)

- egu:105052984

- Up regulated genes

c168511\_g1(1.3724)

- egu:105045727

- Up regulated genes

c162055\_g1(0.89454)

- egu:105042699

- Up regulated genes

c163278\_g1(2.0506)
- egu:105058603

- Up regulated genes

c159288\_g1(0.94417)
- egu:105053416

- Up regulated genes

c161171\_g1(1.6166) c161171\_g2(2.1207)
- egu:105041077

- Up regulated genes

c166224\_g1(0.80628)

- egu:105042699

- Up regulated genes

c163278\_g1(2.0506)
- egu:105058603

- Up regulated genes

c159288\_g1(0.94417)
- egu:105053416

- Up regulated genes

c161171\_g1(1.6166) c161171\_g2(2.1207)
- egu:105041077

- Up regulated genes

c166224\_g1(0.80628)

- egu:105042699

- Up regulated genes

c163278\_g1(2.0506)
- egu:105058603

- Up regulated genes

c159288\_g1(0.94417)
- egu:105053416

- Up regulated genes

c161171\_g1(1.6166) c161171\_g2(2.1207)
- egu:105041077

- Up regulated genes

c166224\_g1(0.80628)

- egu:105042699

- Up regulated genes

c163278\_g1(2.0506)
- egu:105058603

- Up regulated genes

c159288\_g1(0.94417)
- egu:105053416

- Up regulated genes

c161171\_g1(1.6166) c161171\_g2(2.1207)
- egu:105041077

- Up regulated genes

c166224\_g1(0.80628)

- egu:105042699

- Up regulated genes

c163278\_g1(2.0506)
- egu:105058603

- Up regulated genes

c159288\_g1(0.94417)
- egu:105053416

- Up regulated genes

c161171\_g1(1.6166) c161171\_g2(2.1207)
- egu:105041077

- Up regulated genes

c166224\_g1(0.80628)

- egu:105042699

- Up regulated genes

c163278\_g1(2.0506)
- egu:105058603

- Up regulated genes

c159288\_g1(0.94417)
- egu:105053416

- Up regulated genes

c161171\_g1(1.6166) c161171\_g2(2.1207)
- egu:105041077

- Up regulated genes

c166224\_g1(0.80628)

- egu:105042699

- Up regulated genes

c163278\_g1(2.0506)
- egu:105058603

- Up regulated genes

c159288\_g1(0.94417)
- egu:105053416

- Up regulated genes

c161171\_g1(1.6166) c161171\_g2(2.1207)
- egu:105041077

- Up regulated genes

c166224\_g1(0.80628)

- egu:105042699

- Up regulated genes

c163278\_g1(2.0506)
- egu:105058603

- Up regulated genes

c159288\_g1(0.94417)
- egu:105053416

- Up regulated genes

c161171\_g1(1.6166) c161171\_g2(2.1207)
- egu:105041077

- Up regulated genes

c166224\_g1(0.80628)

- egu:105042699

- Up regulated genes

c163278\_g1(2.0506)
- egu:105058603

- Up regulated genes

c159288\_g1(0.94417)
- egu:105053416

- Up regulated genes

c161171\_g1(1.6166) c161171\_g2(2.1207)
- egu:105041077

- Up regulated genes

c166224\_g1(0.80628)

- egu:105042699

- Up regulated genes

c163278\_g1(2.0506)
- egu:105058603

- Up regulated genes

c159288\_g1(0.94417)
- egu:105053416

- Up regulated genes

c161171\_g1(1.6166) c161171\_g2(2.1207)
- egu:105041077

- Up regulated genes

c166224\_g1(0.80628)

- egu:105042699

- Up regulated genes

c163278\_g1(2.0506)
- egu:105058603

- Up regulated genes

c159288\_g1(0.94417)
- egu:105053416

- Up regulated genes

c161171\_g1(1.6166) c161171\_g2(2.1207)
- egu:105041077

- Up regulated genes

c166224\_g1(0.80628)

- egu:105042699

- Up regulated genes

c163278\_g1(2.0506)
- egu:105058603

- Up regulated genes

c159288\_g1(0.94417)
- egu:105053416

- Up regulated genes

c161171\_g1(1.6166) c161171\_g2(2.1207)
- egu:105041077

- Up regulated genes

c166224\_g1(0.80628)

- egu:105060532

- Up regulated genes

c163397\_g1(1.016)

- egu:105060532

- Up regulated genes

c163397\_g1(1.016)

- egu:105060532

- Up regulated genes

c163397\_g1(1.016)

- egu:105046802

- Up regulated genes

c168117\_g1(2.85) c162048\_g1(3.3425)

- egu:105046802

- Up regulated genes

c168117\_g1(2.85) c162048\_g1(3.3425)

- egu:105046802

- Up regulated genes

c168117\_g1(2.85) c162048\_g1(3.3425)

- egu:105046802

- Up regulated genes

c168117\_g1(2.85) c162048\_g1(3.3425)

- egu:105046802

- Up regulated genes

c168117\_g1(2.85) c162048\_g1(3.3425)

- egu:105046802

- Up regulated genes

c168117\_g1(2.85) c162048\_g1(3.3425)

- egu:105046802

- Up regulated genes

c168117\_g1(2.85) c162048\_g1(3.3425)

- egu:105046802

- Up regulated genes

c168117\_g1(2.85) c162048\_g1(3.3425)

- egu:105046802

- Up regulated genes

c168117\_g1(2.85) c162048\_g1(3.3425)

- egu:105046802

- Up regulated genes

c168117\_g1(2.85) c162048\_g1(3.3425)

- egu:105046802

- Up regulated genes

c168117\_g1(2.85) c162048\_g1(3.3425)

- egu:105035937

- Up regulated genes

c173509\_g1(0.94689)

- egu:105046802

- Up regulated genes

c168117\_g1(2.85) c162048\_g1(3.3425)

- egu:105045199

- Up regulated genes

c170749\_g4(1.3881) c170749\_g3(0.99004)
- egu:105042391

- Up regulated genes

c172256\_g4(3.2203) c155636\_g1(3.3411) c155636\_g2(3.0714)

- egu:105048637

- Up regulated genes

c103295\_g1(1.3228)

- egu:105048637

- Up regulated genes

c103295\_g1(1.3228)

- egu:105048637

- Up regulated genes

c103295\_g1(1.3228)

- egu:105048637

- Up regulated genes

c103295\_g1(1.3228)

- egu:105048637

- Up regulated genes

c103295\_g1(1.3228)

- egu:105048637

- Up regulated genes

c103295\_g1(1.3228)

- egu:105048637

- Up regulated genes

c103295\_g1(1.3228)

- egu:105048637

- Up regulated genes

c103295\_g1(1.3228)

- egu:105048637

- Up regulated genes

c103295\_g1(1.3228)

- egu:105048637

- Up regulated genes

c103295\_g1(1.3228)

- egu:105048637

- Up regulated genes

c103295\_g1(1.3228)

- egu:105048637

- Up regulated genes

c103295\_g1(1.3228)

- egu:105048637

- Up regulated genes

c103295\_g1(1.3228)

- egu:105048637

- Up regulated genes

c103295\_g1(1.3228)

- egu:105048637

- Up regulated genes

c103295\_g1(1.3228)

- egu:105048637

- Up regulated genes

c103295\_g1(1.3228)

- egu:105048637

- Up regulated genes

c103295\_g1(1.3228)

- egu:105048637

- Up regulated genes

c103295\_g1(1.3228)

- egu:105048637

- Up regulated genes

c103295\_g1(1.3228)

- egu:105048637

- Up regulated genes

c103295\_g1(1.3228)

- egu:105048637

- Up regulated genes

c103295\_g1(1.3228)

- egu:105048637

- Up regulated genes

c103295\_g1(1.3228)

- egu:105045995

- Up regulated genes

c165806\_g1(3.5125)

- egu:105058232

- Up regulated genes

c146719\_g1(2.8331)

- egu:105058232

- Up regulated genes

c146719\_g1(2.8331)

- egu:105058232

- Up regulated genes

c146719\_g1(2.8331)

- egu:105058232

- Up regulated genes

c146719\_g1(2.8331)

- egu:105058232

- Up regulated genes

c146719\_g1(2.8331)

- egu:105058232

- Up regulated genes

c146719\_g1(2.8331)

- egu:105058232

- Up regulated genes

c146719\_g1(2.8331)

- egu:105058232

- Up regulated genes

c146719\_g1(2.8331)

- egu:105058232

- Up regulated genes

c146719\_g1(2.8331)

- egu:105058232

- Up regulated genes

c146719\_g1(2.8331)

- egu:105058232

- Up regulated genes

c146719\_g1(2.8331)

- egu:105040155

- Up regulated genes

c134112\_g1(0.98133)
- egu:105036609

- Up regulated genes

c129527\_g1(1.6156) c152936\_g1(2.5657)

- egu:105041687

- Up regulated genes

c165885\_g1(0.75955)

- egu:105044276

- Up regulated genes

c170819\_g2(1.17)

- egu:105059274

- Up regulated genes

c161281\_g1(0.79018)

- egu:105059274

- Up regulated genes

c161281\_g1(0.79018)

- egu:105059274

- Up regulated genes

c161281\_g1(0.79018)

- egu:105059274

- Up regulated genes

c161281\_g1(0.79018)

- egu:105059274

- Up regulated genes

c161281\_g1(0.79018)

- egu:105059274

- Up regulated genes

c161281\_g1(0.79018)

- egu:105059274

- Up regulated genes

c161281\_g1(0.79018)

- egu:105059274

- Up regulated genes

c161281\_g1(0.79018)

- egu:105059274

- Up regulated genes

c161281\_g1(0.79018)

- egu:105059274

- Up regulated genes

c161281\_g1(0.79018)

- egu:105059274

- Up regulated genes

c161281\_g1(0.79018)

- egu:105059274

- Up regulated genes

c161281\_g1(0.79018)

- egu:105041133

- Up regulated genes

c170127\_g1(0.93991)

- egu:105046237

- Up regulated genes

c164800\_g1(0.87603)

- egu:105046158

- Up regulated genes

c160696\_g1(0.82836)
- egu:105041372

- Up regulated genes

c168575\_g1(0.56698)

- egu:105046237

- Up regulated genes

c164800\_g1(0.87603)

Close
