# Supplementary material for: Physiological and transcriptomic responses of Lanzhou Lily (Lilium davidii, var. unicolor) to cold stress
Source: PLoS One. 2020 Jan 23;15(1):e0227921. doi: 10.1371/journal.pone.0227921 (PMC6977731; doi:10.1371/journal.pone.0227921)
Supplement: S1 Zip — (Zip). CK: control (20°C); LT: low temperature (4°C). (ZIP) [file pone.0227921.s011.zip › S1 Zip/src/egu03008.html]

egu03008


- egu:105043116

- Up regulated genes

c145725\_g1(1.2642)

- egu:105051885

- Up regulated genes

c159038\_g1(1.0325)

- egu:105032124

- Up regulated genes

c173393\_g1(0.72563)

- egu:105045325

- Up regulated genes

c155970\_g1(0.79014)

- egu:105053596

- Up regulated genes

c143131\_g1(0.69574)

- egu:105058749

- Up regulated genes

c141305\_g1(0.96571)

- egu:105061517

- Up regulated genes

c146280\_g1(1.4736)

- egu:105037954

- Up regulated genes

c170819\_g1(0.966)

- egu:105045184

- Up regulated genes

c168172\_g1(0.64808)

- egu:105040095

- Up regulated genes

c155498\_g1(1.124)

- egu:105035524

- Up regulated genes

c173926\_g1(0.53919)

- egu:105042460

- Up regulated genes

c160458\_g1(0.69771)

- egu:105053627

- Up regulated genes

c174699\_g1(1.5732)

- egu:105049533

- Up regulated genes

c134306\_g1(1.365)

- egu:105032038

- Up regulated genes

c165011\_g1(1.0508)

- egu:105046077

- Up regulated genes

c168781\_g1(1.9465)

- egu:105043717

- Up regulated genes

c174677\_g1(0.75762)

- egu:105051030

- Up regulated genes

c170751\_g1(1.0171)

- egu:105047430

- Up regulated genes

c173905\_g2(0.69787)

- egu:105042999

- Up regulated genes

c166893\_g1(0.99197)

- egu:105059424

- Up regulated genes

c152959\_g1(2.4409)

- egu:105059853

- Up regulated genes

c168253\_g1(0.74584)

- egu:105054755

- Up regulated genes

c172223\_g1(0.69968)

- egu:105058060

- Up regulated genes

c158781\_g1(0.86389) c164010\_g2(0.90497)

- egu:105054834

- Up regulated genes

c167244\_g1(1.0979)

Close
