# Supplementary material for: Physiological and transcriptomic responses of Lanzhou Lily (Lilium davidii, var. unicolor) to cold stress
Source: PLoS One. 2020 Jan 23;15(1):e0227921. doi: 10.1371/journal.pone.0227921 (PMC6977731; doi:10.1371/journal.pone.0227921)
Supplement: S1 Zip — (Zip). CK: control (20°C); LT: low temperature (4°C). (ZIP) [file pone.0227921.s011.zip › S1 Zip/src/egu03010.html]

egu03010


- egu:105033541

- Up regulated genes

c224168\_g1(1.1219)

- egu:105037979

- Up regulated genes

c133240\_g1(1.0982)

- egu:105033079

- Up regulated genes

c157907\_g1(0.79942)

- egu:105058445

- Up regulated genes

c148511\_g1(0.56067)

- egu:105059802

- Up regulated genes

c159076\_g1(0.56466)

- egu:105044285

- Up regulated genes

c165977\_g1(0.50788)

- egu:105059181

- Up regulated genes

c142526\_g1(0.61157)

- egu:105049840

- Up regulated genes

c145296\_g1(1.1823)

- egu:105041276

- Up regulated genes

c157164\_g3(0.77359)

- egu:105044506

- Up regulated genes

c146787\_g1(0.55291)

- egu:105055776

- Up regulated genes

c138227\_g1(0.57714)

- egu:105051463

- Up regulated genes

c13257\_g1(0.99952)

- egu:105042949

- Up regulated genes

c156834\_g1(0.89316)

- egu:105056627

- Up regulated genes

c115398\_g1(0.97441)

- egu:105048878

- Up regulated genes

c158891\_g1(0.53888)

- egu:105032842

- Up regulated genes

c131691\_g1(0.58622)

Close
