# Supplementary material for: Physiological and transcriptomic responses of Lanzhou Lily (Lilium davidii, var. unicolor) to cold stress
Source: PLoS One. 2020 Jan 23;15(1):e0227921. doi: 10.1371/journal.pone.0227921 (PMC6977731; doi:10.1371/journal.pone.0227921)
Supplement: S1 Zip — (Zip). CK: control (20°C); LT: low temperature (4°C). (ZIP) [file pone.0227921.s011.zip › S1 Zip/src/egu03013.html]

egu03013


- egu:105058622

- Up regulated genes

c169995\_g2(1.6425)

- egu:105043116

- Up regulated genes

c145725\_g1(1.2642)

- egu:105043116

- Up regulated genes

c145725\_g1(1.2642)

- egu:105047630

- Up regulated genes

c158413\_g1(1.1487)

- egu:105046248

- Up regulated genes

c155321\_g1(0.84393)

- egu:105041107

- Up regulated genes

c147937\_g1(0.82277)

- egu:105050885

- Up regulated genes

c166802\_g1(0.92581)

- egu:105035083

- Up regulated genes

c169579\_g1(0.65568)

- egu:105052955

- Up regulated genes

c174078\_g4(0.83459)

- egu:105056426

- Up regulated genes

c141122\_g1(0.77519)

- egu:105049252

- Up regulated genes

c167428\_g1(0.62145)

- egu:105043116

- Up regulated genes

c145725\_g1(1.2642)

- egu:105058399

- Up regulated genes

c158591\_g1(0.49252)

- egu:105043116

- Up regulated genes

c145725\_g1(1.2642)

- egu:105051885

- Up regulated genes

c159038\_g1(1.0325)

- egu:105035788

- Up regulated genes

c837\_g1(2.7557)
- egu:105051987

- Up regulated genes

c72097\_g1(2.4728)

- egu:105048848

- Up regulated genes

c171650\_g1(0.85464)
- egu:105056837

- Up regulated genes

c168229\_g1(0.95832)
- egu:105045408

- Up regulated genes

c169217\_g3(3.3357)
- egu:105056889

- Up regulated genes

c147291\_g1(0.6749)

- egu:105046005

- Up regulated genes

c167332\_g2(1.578)

- egu:105050873

- Up regulated genes

c164695\_g7(0.7308)

- egu:105056865

- Up regulated genes

c172809\_g1(0.52567)

- egu:105040546

- Up regulated genes

c165981\_g1(1.1327)

Close
