# Supplementary material for: Physiological and transcriptomic responses of Lanzhou Lily (Lilium davidii, var. unicolor) to cold stress
Source: PLoS One. 2020 Jan 23;15(1):e0227921. doi: 10.1371/journal.pone.0227921 (PMC6977731; doi:10.1371/journal.pone.0227921)
Supplement: S1 Zip — (Zip). CK: control (20°C); LT: low temperature (4°C). (ZIP) [file pone.0227921.s011.zip › S1 Zip/src/egu03015.html]

egu03015


- egu:105034835

- Up regulated genes

c174709\_g3(1.1991)

- egu:105050127

- Up regulated genes

c172927\_g1(0.95322)

- egu:105031982

- Up regulated genes

c169759\_g1(2.8311)

- egu:105042580

- Up regulated genes

c172184\_g1(0.7712)

- egu:105034445

- Up regulated genes

c167848\_g1(5.5328)

- egu:105041936

- Up regulated genes

c163585\_g1(0.97647)

- egu:105032926

- Up regulated genes

c146246\_g2(0.88742)
- egu:105039096

- Up regulated genes

c52599\_g1(0.67814)
- egu:105040379

- Up regulated genes

c139798\_g1(0.54797)

- egu:105049603

- Up regulated genes

c154451\_g2(1.266)

- egu:105060065

- Up regulated genes

c141841\_g1(1.9579)

- egu:105057634

- Up regulated genes

c143034\_g2(0.7777)

Close
