# Supplementary material for: Physiological and transcriptomic responses of Lanzhou Lily (Lilium davidii, var. unicolor) to cold stress
Source: PLoS One. 2020 Jan 23;15(1):e0227921. doi: 10.1371/journal.pone.0227921 (PMC6977731; doi:10.1371/journal.pone.0227921)
Supplement: S1 Zip — (Zip). CK: control (20°C); LT: low temperature (4°C). (ZIP) [file pone.0227921.s011.zip › S1 Zip/src/egu03018.html]

egu03018


- egu:105053561

- Up regulated genes

c163051\_g1(1.3984)

- egu:105058802

- Up regulated genes

c169556\_g1(1.3314)

- egu:105034266

- Up regulated genes

c167106\_g2(1.2941)

- egu:105044893

- Up regulated genes

c162676\_g1(0.80939)

- egu:105046443

- Up regulated genes

c156321\_g2(1.0418)

- egu:105033956

- Up regulated genes

c156792\_g1(1.1601)

- egu:105055716

- Up regulated genes

c152859\_g1(1.8131)
- egu:105044967

- Up regulated genes

c165391\_g1(1.5813)

- egu:105035524

- Up regulated genes

c173926\_g1(0.53919)

- egu:105058802

- Up regulated genes

c169556\_g1(1.3314)

- egu:105060774

- Up regulated genes

c167493\_g1(1.2221)

- egu:105053561

- Up regulated genes

c163051\_g1(1.3984)

Close
