# Supplementary material for: Physiological and transcriptomic responses of Lanzhou Lily (Lilium davidii, var. unicolor) to cold stress
Source: PLoS One. 2020 Jan 23;15(1):e0227921. doi: 10.1371/journal.pone.0227921 (PMC6977731; doi:10.1371/journal.pone.0227921)
Supplement: S1 Zip — (Zip). CK: control (20°C); LT: low temperature (4°C). (ZIP) [file pone.0227921.s011.zip › S1 Zip/src/egu03020.html]

egu03020


- egu:12079509

- Up regulated genes

c174522\_g5(2.3275)

- egu:105060985

- Up regulated genes

c171895\_g1(0.55799)

- egu:105034397

- Up regulated genes

c171631\_g6(1.0224)

- egu:105047342

- Up regulated genes

c166229\_g1(1.1519)

- egu:105039834

- Up regulated genes

c107073\_g1(0.71258)

Close
