# Supplementary material for: Physiological and transcriptomic responses of Lanzhou Lily (Lilium davidii, var. unicolor) to cold stress
Source: PLoS One. 2020 Jan 23;15(1):e0227921. doi: 10.1371/journal.pone.0227921 (PMC6977731; doi:10.1371/journal.pone.0227921)
Supplement: S1 Zip — (Zip). CK: control (20°C); LT: low temperature (4°C). (ZIP) [file pone.0227921.s011.zip › S1 Zip/src/egu03040.html]

egu03040


- egu:105050579

- Up regulated genes

c173699\_g2(1.4387)

- egu:105060553

- Up regulated genes

c172808\_g4(1.337)

- egu:105058818

- Up regulated genes

c160307\_g1(0.67216)
- egu:105052957

- Up regulated genes

c167816\_g1(0.86521)

- egu:105058570

- Up regulated genes

c174104\_g1(0.84253)
- egu:105045020

- Up regulated genes

c165965\_g3(0.98965)

- egu:105055498

- Up regulated genes

c164662\_g3(1.0862)

- egu:105047594

- Up regulated genes

c167228\_g1(0.92338)
- egu:105048592

- Up regulated genes

c162711\_g2(0.96416)
- egu:105038935

- Up regulated genes

c162615\_g1(1.1043)
- egu:105061041

- Up regulated genes

c162711\_g1(0.50382)
- egu:105053664

- Up regulated genes

c164201\_g2(0.56767)

- egu:105045761

- Up regulated genes

c161568\_g3(1.0524)

- egu:105056954

- Up regulated genes

c141698\_g1(1.7769) c141698\_g2(1.4654)
- egu:105045690

- Up regulated genes

c163181\_g5(1.0715)

- egu:105048227

- Up regulated genes

c168415\_g1(0.60255)
- egu:105045262

- Up regulated genes

c165278\_g1(1.3099)

- egu:105058479

- Up regulated genes

c139076\_g1(0.48883)

- egu:105053817

- Up regulated genes

c173165\_g1(1.1456)

- egu:105048742

- Up regulated genes

c155331\_g2(1.4797)

- egu:105060553

- Up regulated genes

c172808\_g4(1.337)

Close
