# Supplementary material for: Physiological and transcriptomic responses of Lanzhou Lily (Lilium davidii, var. unicolor) to cold stress
Source: PLoS One. 2020 Jan 23;15(1):e0227921. doi: 10.1371/journal.pone.0227921 (PMC6977731; doi:10.1371/journal.pone.0227921)
Supplement: S1 Zip — (Zip). CK: control (20°C); LT: low temperature (4°C). (ZIP) [file pone.0227921.s011.zip › S1 Zip/src/egu03410.html]

egu03410


- egu:105057784

- Up regulated genes

c167546\_g1(0.77257)

- egu:105057784

- Up regulated genes

c167546\_g1(0.77257)

- egu:105046296

- Up regulated genes

c160857\_g1(1.4726)

- egu:105046296

- Up regulated genes

c160857\_g1(1.4726)

- egu:105046296

- Up regulated genes

c160857\_g1(1.4726)

- egu:105051450

- Up regulated genes

c173164\_g1(0.9552)

- egu:105057784

- Up regulated genes

c167546\_g1(0.77257)

- egu:105057784

- Up regulated genes

c167546\_g1(0.77257)

- egu:105046296

- Up regulated genes

c160857\_g1(1.4726)

Close
