# Supplementary material for: Physiological and transcriptomic responses of Lanzhou Lily (Lilium davidii, var. unicolor) to cold stress
Source: PLoS One. 2020 Jan 23;15(1):e0227921. doi: 10.1371/journal.pone.0227921 (PMC6977731; doi:10.1371/journal.pone.0227921)
Supplement: S1 Zip — (Zip). CK: control (20°C); LT: low temperature (4°C). (ZIP) [file pone.0227921.s011.zip › S1 Zip/src/egu03420.html]

egu03420


- egu:105057784

- Up regulated genes

c167546\_g1(0.77257)

- egu:105036598

- Up regulated genes

c169225\_g4(1.1113)

- egu:105046296

- Up regulated genes

c160857\_g1(1.4726)

- egu:105040945

- Up regulated genes

c139006\_g1(0.70521)
- egu:105044402

- Up regulated genes

c168792\_g1(0.98868)

- egu:105051054

- Up regulated genes

c172314\_g3(1.0316)

Close
