# Supplementary material for: Physiological and transcriptomic responses of Lanzhou Lily (Lilium davidii, var. unicolor) to cold stress
Source: PLoS One. 2020 Jan 23;15(1):e0227921. doi: 10.1371/journal.pone.0227921 (PMC6977731; doi:10.1371/journal.pone.0227921)
Supplement: S2 Zip — (Zip). CK: control (20°C); LT: low temperature (4°C). (ZIP) [file pone.0227921.s012.zip › S2 Zip/LTvsCK_DOWN/LTvsCK_DOWN.html]

Pathway Enrichment

  

# The most enriched pathway terms

Statistic method: hypergeometric test

FDR correction method: Benjamini and Hochberg

| Term | Sample number | Background number | P-value | Corrected P-value | Gene\_id | KEGG\_ID/KO | Entrez ID | Gene name |
| --- | --- | --- | --- | --- | --- | --- | --- | --- |
| Photosynthesis | 30 | 82 | 1.452222128e-08 | 1.62648878336e-06 | c158839\_g1 c134148\_g1 c237127\_g1 c84948\_g1 c237057\_g1 c42744\_g1 c71692\_g1 c152881\_g1 c121701\_g1 c153649\_g1 c121911\_g1 c76799\_g1 c153193\_g1 c152833\_g1 c106880\_g1 c121905\_g1 c198283\_g1 c158296\_g1 c143903\_g1 c123692\_g1 c84926\_g1 c140011\_g1 c156755\_g1 c76010\_g1 c122782\_g1 c149607\_g1 c48634\_g1 c151822\_g1 c164923\_g1 c131998\_g1 | egu:105033747 egu:105037794 egu:105043122 egu:105055143 egu:105049872 egu:105038285 egu:105037273 egu:105044486 egu:105046198 egu:105046700 egu:12079457 egu:105056235 egu:105053658 egu:105033023 egu:105047072 egu:105033747 egu:105046752 egu:105034183 egu:105049540 egu:105038844 egu:105046935 egu:105037935 egu:105056630 egu:105051755 egu:105034502 egu:105044080 egu:105044241 egu:105054056 egu:12079475 egu:105055013 | 105033747 105037794 105043122 105055143 105049872 105038285 105037273 105044486 105046198 105046700 12079457 105056235 105053658 105033023 105047072 105033747 105046752 105034183 105049540 105038844 105046935 105037935 105056630 105051755 105034502 105044080 105044241 105054056 12079475 105055013 |  |
| Photosynthesis - antenna proteins | 13 | 20 | 1.64311740823e-06 | 9.20145748611e-05 | c139230\_g1 c115377\_g1 c174985\_g1 c155535\_g1 c211152\_g1 c149445\_g1 c153860\_g1 c115694\_g1 c145751\_g1 c152435\_g1 c164577\_g1 c157047\_g1 c149517\_g1 | egu:105053065 egu:105032174 egu:105051572 egu:105032321 egu:105032432 egu:105058558 egu:105058244 egu:105046981 egu:105046981 egu:105039517 egu:105035084 egu:105058393 egu:105033408 | 105053065 105032174 105051572 105032321 105032432 105058558 105058244 105046981 105046981 105039517 105035084 105058393 105033408 |  |
| Porphyrin and chlorophyll metabolism | 18 | 50 | 1.32748654706e-05 | 0.00049559497757 | c168519\_g1 c169028\_g1 c185147\_g2 c167947\_g1 c133188\_g1 c134612\_g1 c170780\_g1 c71809\_g1 c155686\_g1 c157850\_g1 c71670\_g1 c161205\_g1 c123480\_g1 c165450\_g1 c167743\_g1 c152307\_g1 c166557\_g2 c166557\_g1 | egu:105040768 egu:105057582 egu:105036097 egu:105054529 egu:105040656 egu:105035618 egu:105054529 egu:105049221 egu:105044579 egu:105035555 egu:105037930 egu:105052855 egu:105035938 egu:105044798 egu:105051026 egu:105059913 egu:105058545 egu:105058545 | 105040768 105057582 105036097 105054529 105040656 105035618 105054529 105049221 105044579 105035555 105037930 105052855 105035938 105044798 105051026 105059913 105058545 105058545 |  |
| Carbon fixation in photosynthetic organisms | 24 | 89 | 3.09333159827e-05 | 0.000866132847516 | c121900\_g1 c174574\_g3 c173582\_g1 c198353\_g1 c169723\_g1 c144640\_g1 c158889\_g1 c162112\_g2 c159323\_g1 c154502\_g4 c155247\_g1 c168133\_g3 c169641\_g1 c173703\_g2 c164307\_g1 c160123\_g1 c104889\_g2 c104889\_g1 c154303\_g1 c170804\_g2 c170857\_g1 c162034\_g1 c147541\_g1 c71483\_g1 | egu:105042746 egu:105054530 egu:105056157 egu:105059611 egu:105038099 egu:105051883 egu:105046280 egu:105050625 egu:105048107 egu:105035321 egu:105048825 egu:105048437 egu:105055679 egu:105034557 egu:105043976 egu:105060347 egu:105054530 egu:105034557 egu:105032039 egu:105048474 egu:105038009 egu:105056168 egu:105057517 egu:105049882 | 105042746 105054530 105056157 105059611 105038099 105051883 105046280 105050625 105048107 105035321 105048825 105048437 105055679 105034557 105043976 105060347 105054530 105034557 105032039 105048474 105038009 105056168 105057517 105049882 |  |
| Ubiquinone and other terpenoid-quinone biosynthesis | 9 | 36 | 0.0138737390701 | 0.274520863623 | c156623\_g1 c185151\_g1 c154629\_g1 c165685\_g1 c159709\_g1 c166982\_g1 c152469\_g1 c152224\_g1 c173971\_g3 | egu:105040851 egu:105040940 egu:105047162 egu:105041933 egu:105046456 egu:105053482 egu:105035064 egu:105055609 egu:105060927 | 105040851 105040940 105047162 105041933 105046456 105053482 105035064 105055609 105060927 |  |
| Fatty acid elongation | 8 | 30 | 0.0147064748369 | 0.274520863623 | c151794\_g1 c2875\_g1 c2875\_g2 c150017\_g1 c148702\_g1 c134603\_g2 c164323\_g1 c164323\_g2 | egu:105040213 egu:105042530 egu:105047853 egu:105041077 egu:105044215 egu:105039895 egu:105048315 egu:105047165 | 105040213 105042530 105047853 105041077 105044215 105039895 105048315 105047165 |  |
| Starch and sucrose metabolism | 34 | 243 | 0.0249327019242 | 0.398923230787 | c171202\_g1 c156351\_g3 c172165\_g1 c159732\_g1 c171119\_g1 c173060\_g2 c227079\_g1 c164821\_g1 c133070\_g1 c173942\_g4 c167282\_g1 c132815\_g1 c152658\_g1 c169016\_g1 c171033\_g3 c156351\_g1 c20180\_g1 c156351\_g5 c143878\_g1 c146228\_g1 c104638\_g1 c170497\_g1 c98676\_g1 c170862\_g1 c169825\_g1 c168535\_g1 c172683\_g1 c156351\_g6 c166462\_g1 c174706\_g1 c163786\_g2 c150214\_g1 c172732\_g1 c163786\_g1 | egu:105053029 egu:105042109 egu:105039195 egu:105038864 egu:105047967 egu:105042390 egu:105058120 egu:105044229 egu:105060694 egu:105044265 egu:105050772 egu:105041065 egu:105049809 egu:105042381 egu:105045835 egu:105048485 egu:105032401 egu:105048485 egu:105056575 egu:105045201 egu:105040139 egu:105056534 egu:105060491 egu:105049657 egu:105047967 egu:105041389 egu:105033733 egu:105053174 egu:105043601 egu:105034542 egu:105052110 egu:105044696 egu:105042109 egu:105052110 | 105053029 105042109 105039195 105038864 105047967 105042390 105058120 105044229 105060694 105044265 105050772 105041065 105049809 105042381 105045835 105048485 105032401 105048485 105056575 105045201 105040139 105056534 105060491 105049657 105047967 105041389 105033733 105053174 105043601 105034542 105052110 105044696 105042109 105052110 |  |
| Metabolic pathways | 232 | 2161 | 0.0298806303676 | 0.418328825146 | c171202\_g1 c132497\_g1 c198353\_g1 c133188\_g1 c134612\_g1 c134148\_g1 c237127\_g1 c163865\_g3 c134153\_g1 c160287\_g1 c158303\_g2 c165968\_g2 c152469\_g1 c162112\_g2 c163450\_g1 c164810\_g1 c153649\_g1 c85645\_g1 c173942\_g4 c173664\_g2 c164754\_g1 c167282\_g1 c173864\_g1 c152833\_g1 c143120\_g2 c113371\_g2 c169016\_g1 c171033\_g3 c156351\_g1 c168470\_g1 c156351\_g5 c143903\_g1 c123692\_g1 c167954\_g1 c132652\_g1 c71809\_g1 c104638\_g1 c167743\_g1 c171016\_g1 c137214\_g2 c141783\_g1 c104889\_g2 c104889\_g1 c154303\_g1 c162118\_g1 c157047\_g1 c173971\_g3 c170240\_g1 c151227\_g1 c185147\_g2 c170804\_g2 c164923\_g1 c156623\_g1 c165685\_g1 c166462\_g1 c98584\_g1 c152294\_g2 c164585\_g7 c160082\_g2 c159141\_g1 c166072\_g1 c158088\_g1 c104600\_g1 c154629\_g1 c164015\_g2 c153630\_g1 c169929\_g1 c171137\_g1 c152881\_g1 c147908\_g1 c173060\_g2 c152658\_g1 c168329\_g1 c169028\_g1 c173363\_g5 c154502\_g4 c133070\_g1 c155535\_g1 c157850\_g1 c163701\_g1 c106880\_g1 c173703\_g2 c156465\_g1 c165472\_g1 c152224\_g1 c167963\_g1 c20180\_g1 c156209\_g1 c155686\_g1 c164784\_g1 c132393\_g1 c152651\_g1 c163023\_g1 c146228\_g1 c71483\_g1 c169665\_g2 c160123\_g1 c121900\_g1 c174574\_g3 c145751\_g1 c163317\_g1 c48670\_g1 c164585\_g11 c154527\_g1 c140011\_g1 c170862\_g1 c158576\_g4 c84926\_g1 c166380\_g2 c131939\_g1 c161205\_g1 c174660\_g1 c166827\_g1 c169731\_g1 c164577\_g1 c163786\_g2 c150214\_g1 c163786\_g1 c159963\_g1 c48634\_g1 c144640\_g1 c153259\_g1 c167873\_g1 c166497\_g1 c151091\_g1 c166749\_g1 c121911\_g1 c166374\_g1 c168133\_g3 c154844\_g1 c170620\_g6 c158839\_g1 c167947\_g1 c157388\_g1 c153193\_g1 c166378\_g2 c147467\_g1 c134759\_g1 c105237\_g1 c172165\_g1 c135610\_g1 c169514\_g5 c164307\_g1 c174800\_g1 c159032\_g1 c164707\_g1 c121905\_g1 c171119\_g1 c162518\_g1 c152279\_g1 c169641\_g1 c170780\_g1 c161796\_g1 c166982\_g1 c149607\_g1 c115694\_g1 c168498\_g1 c165450\_g1 c156755\_g1 c173984\_g2 c168210\_g7 c168210\_g6 c151822\_g1 c172683\_g1 c162034\_g1 c147541\_g1 c157902\_g1 c71670\_g1 c153585\_g1 c172732\_g1 c101133\_g1 c173582\_g1 c158106\_g1 c174985\_g1 c185151\_g1 c169723\_g1 c224136\_g1 c147420\_g1 c164056\_g1 c71692\_g1 c153334\_g1 c237057\_g1 c156351\_g3 c133760\_g1 c158889\_g1 c152482\_g1 c152607\_g1 c121701\_g1 c166557\_g2 c166557\_g1 c159804\_g1 c132118\_g1 c154382\_g1 c164821\_g1 c155247\_g1 c134111\_g1 c162392\_g1 c211844\_g1 c159323\_g1 c166298\_g1 c150506\_g1 c152307\_g1 c171137\_g4 c115377\_g1 c198283\_g1 c168519\_g1 c168243\_g1 c166358\_g1 c124333\_g1 c172387\_g1 c131998\_g1 c123480\_g1 c168878\_g2 c155451\_g1 c155055\_g1 c170497\_g1 c122896\_g1 c76010\_g1 c170857\_g1 c143298\_g1 c169825\_g1 c168535\_g1 c146595\_g1 c156351\_g6 c171508\_g2 c171508\_g1 c113031\_g1 c163774\_g1 c174706\_g1 c131571\_g1 c171087\_g1 c123366\_g1 | egu:105053029 egu:105059577 egu:105059611 egu:105040656 egu:105035618 egu:105037794 egu:105043122 egu:105048612 egu:105043499 egu:105050888 egu:105033675 egu:105044935 egu:105035064 egu:105050625 egu:105046522 egu:105052573 egu:105046700 egu:105049380 egu:105044265 egu:105050243 egu:105053059 egu:105050772 egu:105055141 egu:105033023 egu:105032337 egu:105034969 egu:105042381 egu:105045835 egu:105048485 egu:105053765 egu:105048485 egu:105049540 egu:105038844 egu:105047663 egu:105050388 egu:105049221 egu:105040139 egu:105051026 egu:105045448 egu:105038852 egu:105033284 egu:105054530 egu:105034557 egu:105032039 egu:105043957 egu:105058393 egu:105060927 egu:105052168 egu:105054950 egu:105036097 egu:105048474 egu:12079475 egu:105040851 egu:105041933 egu:105043601 egu:105056213 egu:105055883 egu:105052170 egu:105033054 egu:105058326 egu:105038832 egu:105057795 egu:105033050 egu:105047162 egu:105045732 egu:105051428 egu:105048467 egu:105059466 egu:105044486 egu:105039298 egu:105042390 egu:105049809 egu:105041662 egu:105057582 egu:12079461 egu:105035321 egu:105060694 egu:105032321 egu:105035555 egu:105035926 egu:105047072 egu:105034557 egu:105043485 egu:105037657 egu:105055609 egu:105059048 egu:105032401 egu:105044629 egu:105044579 egu:105040461 egu:105051539 egu:105043746 egu:105055246 egu:105045201 egu:105049882 egu:105044713 egu:105060347 egu:105042746 egu:105054530 egu:105046981 egu:105056567 egu:105053770 egu:12079395 egu:105036212 egu:105037935 egu:105049657 egu:105055982 egu:105046935 egu:105060549 egu:105038325 egu:105052855 egu:105047063 egu:105057722 egu:105039221 egu:105035084 egu:105052110 egu:105044696 egu:105052110 egu:105033796 egu:105044241 egu:105051883 egu:105054827 egu:105032148 egu:105035100 egu:105034893 egu:105035252 egu:12079457 egu:105034612 egu:105048437 egu:105055979 egu:105039067 egu:105033747 egu:105054529 egu:105052174 egu:105053658 egu:105059182 egu:105043191 egu:105050897 egu:105059645 egu:105039195 egu:105044125 egu:105048729 egu:105043976 egu:105055783 egu:105058894 egu:105035680 egu:105033747 egu:105047967 egu:105037896 egu:105051936 egu:105055679 egu:105054529 egu:105049214 egu:105053482 egu:105044080 egu:105046981 egu:105057579 egu:105044798 egu:105056630 egu:105041599 egu:105043452 egu:105043452 egu:105054056 egu:105033733 egu:105056168 egu:105057517 egu:105047380 egu:105037930 egu:105039235 egu:105042109 egu:105042952 egu:105056157 egu:105051928 egu:105051572 egu:105040940 egu:105038099 egu:105060163 egu:105044732 egu:105036454 egu:105037273 egu:105050202 egu:105049872 egu:105042109 egu:105036939 egu:105046280 egu:105046559 egu:105043264 egu:105046198 egu:105058545 egu:105058545 egu:105036836 egu:105034995 egu:105051526 egu:105044229 egu:105048825 egu:105056640 egu:105041436 egu:105041694 egu:105048107 egu:105057721 egu:105043430 egu:105059913 egu:105059466 egu:105032174 egu:105046752 egu:105040768 egu:105049020 egu:105046827 egu:105055151 egu:105044978 egu:105055013 egu:105035938 egu:105056476 egu:105050983 egu:105049274 egu:105056534 egu:105046147 egu:105051755 egu:105038009 egu:105041725 egu:105047967 egu:105041389 egu:105057316 egu:105053174 egu:105052170 egu:105052170 egu:105061169 egu:105056650 egu:105034542 egu:105034341 egu:105044348 egu:105060320 | 105053029 105059577 105059611 105040656 105035618 105037794 105043122 105048612 105043499 105050888 105033675 105044935 105035064 105050625 105046522 105052573 105046700 105049380 105044265 105050243 105053059 105050772 105055141 105033023 105032337 105034969 105042381 105045835 105048485 105053765 105048485 105049540 105038844 105047663 105050388 105049221 105040139 105051026 105045448 105038852 105033284 105054530 105034557 105032039 105043957 105058393 105060927 105052168 105054950 105036097 105048474 12079475 105040851 105041933 105043601 105056213 105055883 105052170 105033054 105058326 105038832 105057795 105033050 105047162 105045732 105051428 105048467 105059466 105044486 105039298 105042390 105049809 105041662 105057582 12079461 105035321 105060694 105032321 105035555 105035926 105047072 105034557 105043485 105037657 105055609 105059048 105032401 105044629 105044579 105040461 105051539 105043746 105055246 105045201 105049882 105044713 105060347 105042746 105054530 105046981 105056567 105053770 12079395 105036212 105037935 105049657 105055982 105046935 105060549 105038325 105052855 105047063 105057722 105039221 105035084 105052110 105044696 105052110 105033796 105044241 105051883 105054827 105032148 105035100 105034893 105035252 12079457 105034612 105048437 105055979 105039067 105033747 105054529 105052174 105053658 105059182 105043191 105050897 105059645 105039195 105044125 105048729 105043976 105055783 105058894 105035680 105033747 105047967 105037896 105051936 105055679 105054529 105049214 105053482 105044080 105046981 105057579 105044798 105056630 105041599 105043452 105043452 105054056 105033733 105056168 105057517 105047380 105037930 105039235 105042109 105042952 105056157 105051928 105051572 105040940 105038099 105060163 105044732 105036454 105037273 105050202 105049872 105042109 105036939 105046280 105046559 105043264 105046198 105058545 105058545 105036836 105034995 105051526 105044229 105048825 105056640 105041436 105041694 105048107 105057721 105043430 105059913 105059466 105032174 105046752 105040768 105049020 105046827 105055151 105044978 105055013 105035938 105056476 105050983 105049274 105056534 105046147 105051755 105038009 105041725 105047967 105041389 105057316 105053174 105052170 105052170 105061169 105056650 105034542 105034341 105044348 105060320 |  |
| Fatty acid biosynthesis | 11 | 59 | 0.038031460833 | 0.473280401478 | c154844\_g1 c153334\_g1 c104546\_g1 c155055\_g1 c152279\_g1 c164754\_g1 c153259\_g1 c172387\_g1 c159141\_g1 c169731\_g1 c147420\_g1 | egu:105055979 egu:105050202 egu:105049664 egu:105049274 egu:105051936 egu:105053059 egu:105054827 egu:105044978 egu:105058326 egu:105039221 egu:105044732 | 105055979 105050202 105049664 105049274 105051936 105053059 105054827 105044978 105058326 105039221 105044732 |  |
| Glyoxylate and dicarboxylate metabolism | 13 | 83 | 0.071388870122 | 0.799555345366 | c153630\_g1 c137804\_g1 c132497\_g1 c167954\_g1 c132393\_g1 c158088\_g1 c168133\_g3 c157902\_g1 c163701\_g1 c169641\_g1 c71483\_g1 c48670\_g1 c147908\_g1 | egu:105051428 egu:105057601 egu:105059577 egu:105047663 egu:105051539 egu:105057795 egu:105048437 egu:105047380 egu:105035926 egu:105055679 egu:105049882 egu:105053770 egu:105039298 | 105051428 105057601 105059577 105047663 105051539 105057795 105048437 105047380 105035926 105055679 105049882 105053770 105039298 |  |
| Lipoic acid metabolism | 2 | 5 | 0.118083808798 | 0.911723784586 | c164707\_g1 c163865\_g3 | egu:105035680 egu:105048612 | 105035680 105048612 |  |
| Tropane, piperidine and pyridine alkaloid biosynthesis | 4 | 18 | 0.118170865888 | 0.911723784586 | c185151\_g1 c171087\_g1 c159323\_g1 c135610\_g1 | egu:105040940 egu:105044348 egu:105048107 egu:105044125 | 105040940 105044348 105048107 105044125 |  |
| Carbon metabolism | 38 | 322 | 0.120045739881 | 0.911723784586 | c121900\_g1 c104889\_g1 c173582\_g1 c198353\_g1 c169723\_g1 c144640\_g1 c153259\_g1 c158889\_g1 c162112\_g2 c159323\_g1 c153630\_g1 c154844\_g1 c155055\_g1 c154502\_g4 c155247\_g1 c147908\_g1 c168133\_g3 c163701\_g1 c169641\_g1 c173703\_g2 c164307\_g1 c167954\_g1 c132393\_g1 c162392\_g1 c164784\_g1 c113371\_g2 c160123\_g1 c104889\_g2 c174574\_g3 c154303\_g1 c48670\_g1 c170804\_g2 c170857\_g1 c162034\_g1 c133070\_g1 c147541\_g1 c157902\_g1 c71483\_g1 | egu:105042746 egu:105034557 egu:105056157 egu:105059611 egu:105038099 egu:105051883 egu:105054827 egu:105046280 egu:105050625 egu:105048107 egu:105051428 egu:105055979 egu:105049274 egu:105035321 egu:105048825 egu:105039298 egu:105048437 egu:105035926 egu:105055679 egu:105034557 egu:105043976 egu:105047663 egu:105051539 egu:105041436 egu:105040461 egu:105034969 egu:105060347 egu:105054530 egu:105054530 egu:105032039 egu:105053770 egu:105048474 egu:105038009 egu:105056168 egu:105060694 egu:105057517 egu:105047380 egu:105049882 | 105042746 105034557 105056157 105059611 105038099 105051883 105054827 105046280 105050625 105048107 105051428 105055979 105049274 105035321 105048825 105039298 105048437 105035926 105055679 105034557 105043976 105047663 105051539 105041436 105040461 105034969 105060347 105054530 105054530 105032039 105053770 105048474 105038009 105056168 105060694 105057517 105047380 105049882 |  |
| Riboflavin metabolism | 3 | 12 | 0.135744242674 | 0.911723784586 | c166380\_g2 c154382\_g1 c164015\_g2 | egu:105060549 egu:105051526 egu:105045732 | 105060549 105051526 105045732 |  |
| Regulation of autophagy | 7 | 43 | 0.139600711328 | 0.911723784586 | c146711\_g1 c162703\_g1 c163487\_g1 c145728\_g1 c140985\_g1 c173419\_g5 c167169\_g2 | egu:105058890 egu:105061507 egu:105043253 egu:105058890 egu:105058890 egu:105058890 egu:105061507 | 105058890 105061507 105043253 105058890 105058890 105058890 105061507 |  |
| Ribosome | 43 | 377 | 0.144885021146 | 0.911723784586 | c142600\_g1 c154494\_g1 c27497\_g1 c163280\_g1 c156427\_g1 c157065\_g1 c158737\_g2 c178004\_g1 c151905\_g1 c158858\_g1 c155204\_g1 c141711\_g1 c161542\_g1 c172832\_g1 c145214\_g1 c106647\_g1 c143129\_g1 c160468\_g1 c224200\_g1 c94348\_g1 c116569\_g1 c160112\_g1 c141240\_g1 c168022\_g1 c142521\_g1 c163095\_g3 c134157\_g1 c138659\_g1 c161109\_g1 c161348\_g1 c132904\_g1 c159369\_g1 c48496\_g1 c150484\_g1 c132540\_g1 c140525\_g1 c147799\_g1 c134374\_g1 c156664\_g1 c101971\_g1 c131320\_g1 c143884\_g1 c133119\_g1 | egu:105047611 egu:105036935 egu:105052661 egu:105052800 egu:105059074 egu:105034390 egu:105032797 egu:105032412 egu:105035316 egu:105060920 egu:105032412 egu:105048529 egu:105044181 egu:105045120 egu:105034395 egu:105053938 egu:105056818 egu:105041074 egu:105059189 egu:105048206 egu:105046043 egu:105041287 egu:105048988 egu:105044673 egu:105034999 egu:105055772 egu:105045280 egu:105041319 egu:105060039 egu:105061188 egu:105036502 egu:105061575 egu:105033340 egu:105040763 egu:105034754 egu:105042443 egu:105047719 egu:105040137 egu:105057725 egu:105039272 egu:12079413 egu:105043313 egu:105044970 | 105047611 105036935 105052661 105052800 105059074 105034390 105032797 105032412 105035316 105060920 105032412 105048529 105044181 105045120 105034395 105053938 105056818 105041074 105059189 105048206 105046043 105041287 105048988 105044673 105034999 105055772 105045280 105041319 105060039 105061188 105036502 105061575 105033340 105040763 105034754 105042443 105047719 105040137 105057725 105039272 12079413 105043313 105044970 |  |
| Biosynthesis of secondary metabolites | 124 | 1184 | 0.15297999015 | 0.911723784586 | c132497\_g1 c198353\_g1 c133188\_g1 c134612\_g1 c165685\_g1 c158303\_g2 c165968\_g2 c152469\_g1 c148702\_g1 c167963\_g1 c85645\_g1 c173942\_g4 c153630\_g1 c113371\_g2 c169016\_g1 c171033\_g3 c164902\_g1 c168470\_g1 c162392\_g1 c71809\_g1 c104638\_g1 c167743\_g1 c171016\_g1 c137214\_g2 c141783\_g1 c104889\_g2 c104889\_g1 c154303\_g1 c162118\_g1 c173971\_g3 c151227\_g1 c185147\_g2 c170804\_g2 c156623\_g1 c152294\_g2 c166072\_g1 c104600\_g1 c154629\_g1 c162034\_g1 c147908\_g1 c173060\_g2 c168329\_g1 c169028\_g1 c155055\_g1 c154502\_g4 c133070\_g1 c157850\_g1 c160710\_g1 c163701\_g1 c132652\_g1 c173703\_g2 c165472\_g1 c152224\_g1 c156209\_g1 c164784\_g1 c146228\_g1 c71483\_g1 c155686\_g1 c174574\_g3 c166497\_g1 c48670\_g1 c158576\_g4 c161205\_g1 c174706\_g1 c134153\_g1 c144640\_g1 c2875\_g1 c153259\_g1 c2875\_g2 c166374\_g1 c154844\_g1 c167947\_g1 c157388\_g1 c147467\_g1 c105237\_g1 c135610\_g1 c166982\_g1 c159032\_g1 c162518\_g1 c169641\_g1 c170780\_g1 c165450\_g1 c174739\_g1 c164015\_g2 c147541\_g1 c157902\_g1 c71670\_g1 c101133\_g1 c173582\_g1 c185151\_g1 c224136\_g1 c164056\_g1 c152607\_g1 c166557\_g2 c166557\_g1 c162112\_g2 c159804\_g1 c154382\_g1 c164821\_g1 c155247\_g1 c168243\_g1 c159323\_g1 c150017\_g1 c166298\_g1 c152307\_g1 c151794\_g1 c134111\_g1 c168519\_g1 c166358\_g1 c124333\_g1 c170240\_g1 c166380\_g2 c123480\_g1 c134603\_g2 c164323\_g1 c164323\_g2 c122896\_g1 c170857\_g1 c146595\_g1 c113031\_g1 c163774\_g1 c12992\_g1 c131571\_g1 c171087\_g1 | egu:105059577 egu:105059611 egu:105040656 egu:105035618 egu:105041933 egu:105033675 egu:105044935 egu:105035064 egu:105044215 egu:105059048 egu:105049380 egu:105044265 egu:105051428 egu:105034969 egu:105042381 egu:105045835 egu:105033309 egu:105053765 egu:105041436 egu:105049221 egu:105040139 egu:105051026 egu:105045448 egu:105038852 egu:105033284 egu:105054530 egu:105034557 egu:105032039 egu:105043957 egu:105060927 egu:105054950 egu:105036097 egu:105048474 egu:105040851 egu:105055883 egu:105038832 egu:105033050 egu:105047162 egu:105056168 egu:105039298 egu:105042390 egu:105041662 egu:105057582 egu:105049274 egu:105035321 egu:105060694 egu:105035555 egu:105034750 egu:105035926 egu:105050388 egu:105034557 egu:105037657 egu:105055609 egu:105044629 egu:105040461 egu:105045201 egu:105049882 egu:105044579 egu:105054530 egu:105035100 egu:105053770 egu:105055982 egu:105052855 egu:105034542 egu:105043499 egu:105051883 egu:105042530 egu:105054827 egu:105047853 egu:105034612 egu:105055979 egu:105054529 egu:105052174 egu:105043191 egu:105059645 egu:105044125 egu:105053482 egu:105058894 egu:105037896 egu:105055679 egu:105054529 egu:105044798 egu:105034750 egu:105045732 egu:105057517 egu:105047380 egu:105037930 egu:105042952 egu:105056157 egu:105040940 egu:105060163 egu:105036454 egu:105043264 egu:105058545 egu:105058545 egu:105050625 egu:105036836 egu:105051526 egu:105044229 egu:105048825 egu:105049020 egu:105048107 egu:105041077 egu:105057721 egu:105059913 egu:105040213 egu:105056640 egu:105040768 egu:105046827 egu:105055151 egu:105052168 egu:105060549 egu:105035938 egu:105039895 egu:105048315 egu:105047165 egu:105046147 egu:105038009 egu:105057316 egu:105061169 egu:105056650 egu:105052307 egu:105034341 egu:105044348 | 105059577 105059611 105040656 105035618 105041933 105033675 105044935 105035064 105044215 105059048 105049380 105044265 105051428 105034969 105042381 105045835 105033309 105053765 105041436 105049221 105040139 105051026 105045448 105038852 105033284 105054530 105034557 105032039 105043957 105060927 105054950 105036097 105048474 105040851 105055883 105038832 105033050 105047162 105056168 105039298 105042390 105041662 105057582 105049274 105035321 105060694 105035555 105034750 105035926 105050388 105034557 105037657 105055609 105044629 105040461 105045201 105049882 105044579 105054530 105035100 105053770 105055982 105052855 105034542 105043499 105051883 105042530 105054827 105047853 105034612 105055979 105054529 105052174 105043191 105059645 105044125 105053482 105058894 105037896 105055679 105054529 105044798 105034750 105045732 105057517 105047380 105037930 105042952 105056157 105040940 105060163 105036454 105043264 105058545 105058545 105050625 105036836 105051526 105044229 105048825 105049020 105048107 105041077 105057721 105059913 105040213 105056640 105040768 105046827 105055151 105052168 105060549 105035938 105039895 105048315 105047165 105046147 105038009 105057316 105061169 105056650 105052307 105034341 105044348 |  |
| Amino sugar and nucleotide sugar metabolism | 20 | 161 | 0.157189739349 | 0.911723784586 | c170497\_g1 c98676\_g1 c133303\_g2 c159732\_g1 c132815\_g1 c168210\_g7 c168210\_g6 c133070\_g1 c157388\_g1 c151091\_g1 c143878\_g1 c104638\_g1 c149405\_g1 c160287\_g1 c158447\_g1 c20180\_g1 c150506\_g1 c152658\_g1 c123366\_g1 c227079\_g1 | egu:105056534 egu:105060491 egu:105040562 egu:105038864 egu:105041065 egu:105043452 egu:105043452 egu:105060694 egu:105052174 egu:105034893 egu:105056575 egu:105040139 egu:105054161 egu:105050888 egu:105039344 egu:105032401 egu:105043430 egu:105049809 egu:105060320 egu:105058120 | 105056534 105060491 105040562 105038864 105041065 105043452 105043452 105060694 105052174 105034893 105056575 105040139 105054161 105050888 105039344 105032401 105043430 105049809 105060320 105058120 |  |
| ABC transporters | 5 | 29 | 0.16850708953 | 0.911723784586 | c169285\_g2 c161768\_g1 c157873\_g1 c169285\_g1 c173719\_g3 | egu:105055560 egu:105040552 egu:105056548 egu:105059124 egu:105052956 | 105055560 105040552 105056548 105059124 105052956 |  |
| Glycolysis / Gluconeogenesis | 19 | 154 | 0.17113734825 | 0.911723784586 | c134153\_g1 c154502\_g4 c173582\_g1 c162392\_g1 c133070\_g1 c198353\_g1 c104889\_g1 c162034\_g1 c144640\_g1 c170857\_g1 c104889\_g2 c174574\_g3 c113371\_g2 c173703\_g2 c162112\_g2 c166374\_g1 c170804\_g2 c132652\_g1 c85645\_g1 | egu:105043499 egu:105035321 egu:105056157 egu:105041436 egu:105060694 egu:105059611 egu:105034557 egu:105056168 egu:105051883 egu:105038009 egu:105054530 egu:105054530 egu:105034969 egu:105034557 egu:105050625 egu:105034612 egu:105048474 egu:105050388 egu:105049380 | 105043499 105035321 105056157 105041436 105060694 105059611 105034557 105056168 105051883 105038009 105054530 105054530 105034969 105034557 105050625 105034612 105048474 105050388 105049380 |  |
| Fructose and mannose metabolism | 12 | 90 | 0.173382016851 | 0.911723784586 | c85645\_g1 c154502\_g4 c198353\_g1 c170857\_g1 c168210\_g6 c144640\_g1 c170804\_g2 c168210\_g7 c157388\_g1 c162112\_g2 c166827\_g1 c152658\_g1 | egu:105049380 egu:105035321 egu:105059611 egu:105038009 egu:105043452 egu:105051883 egu:105048474 egu:105043452 egu:105052174 egu:105050625 egu:105057722 egu:105049809 | 105049380 105035321 105059611 105038009 105043452 105051883 105048474 105043452 105052174 105050625 105057722 105049809 |  |
| Nitrogen metabolism | 6 | 38 | 0.179088600544 | 0.911723784586 | c137804\_g1 c142597\_g1 c115915\_g1 c185512\_g1 c158088\_g1 c145787\_g1 | egu:105057601 egu:105052122 egu:105048068 egu:105033813 egu:105057795 egu:105048068 | 105057601 105052122 105048068 105033813 105057795 105048068 |  |
| Tyrosine metabolism | 6 | 39 | 0.192572875335 | 0.937746175544 | c159338\_g1 c162392\_g1 c185151\_g1 c135610\_g1 c159323\_g1 c166374\_g1 | egu:105050858 egu:105041436 egu:105040940 egu:105044125 egu:105048107 egu:105034612 | 105050858 105041436 105040940 105044125 105048107 105034612 |  |
| Fatty acid metabolism | 11 | 85 | 0.210000291051 | 0.980001358238 | c154844\_g1 c153334\_g1 c159141\_g1 c155055\_g1 c152279\_g1 c164754\_g1 c153259\_g1 c172387\_g1 c137480\_g1 c169731\_g1 c147420\_g1 | egu:105055979 egu:105050202 egu:105058326 egu:105049274 egu:105051936 egu:105053059 egu:105054827 egu:105044978 egu:105061227 egu:105039221 egu:105044732 | 105055979 105050202 105058326 105049274 105051936 105053059 105054827 105044978 105061227 105039221 105044732 |  |
| Glycine, serine and threonine metabolism | 10 | 79 | 0.242803954937 | 0.999827344552 | c135610\_g1 c132497\_g1 c168243\_g1 c71483\_g1 c157902\_g1 c113031\_g1 c163701\_g1 c133760\_g1 c48670\_g1 c147908\_g1 | egu:105044125 egu:105059577 egu:105049020 egu:105049882 egu:105047380 egu:105061169 egu:105035926 egu:105036939 egu:105053770 egu:105039298 | 105044125 105059577 105049020 105049882 105047380 105061169 105035926 105036939 105053770 105039298 |  |
| Carotenoid biosynthesis | 5 | 34 | 0.247834292732 | 0.999827344552 | c166298\_g1 c137214\_g2 c166072\_g1 c159032\_g1 c163774\_g1 | egu:105057721 egu:105038852 egu:105038832 egu:105058894 egu:105056650 | 105057721 105038852 105038832 105058894 105056650 |  |
| Cyanoamino acid metabolism | 6 | 43 | 0.249925226192 | 0.999827344552 | c171033\_g3 c164821\_g1 c173942\_g4 c146228\_g1 c174706\_g1 c173060\_g2 | egu:105045835 egu:105044229 egu:105044265 egu:105045201 egu:105034542 egu:105042390 | 105045835 105044229 105044265 105045201 105034542 105042390 |  |
| Pentose phosphate pathway | 10 | 80 | 0.253647891142 | 0.999827344552 | c85645\_g1 c154502\_g4 c170804\_g2 c155247\_g1 c144640\_g1 c133070\_g1 c147541\_g1 c170857\_g1 c154303\_g1 c162112\_g2 | egu:105049380 egu:105035321 egu:105048474 egu:105048825 egu:105051883 egu:105060694 egu:105057517 egu:105038009 egu:105032039 egu:105050625 | 105049380 105035321 105048474 105048825 105051883 105060694 105057517 105038009 105032039 105050625 |  |
| Glycosaminoglycan degradation | 3 | 18 | 0.273333766396 | 0.999827344552 | c151091\_g1 c160082\_g2 c168498\_g1 | egu:105034893 egu:105033054 egu:105057579 | 105034893 105033054 105057579 |  |
| Aminoacyl-tRNA biosynthesis | 11 | 94 | 0.302803434027 | 0.999827344552 | c159463\_g1 c162191\_g1 c132118\_g1 c169070\_g1 c95934\_g1 c163858\_g1 c161964\_g1 c161205\_g1 c163317\_g1 c174800\_g1 c162019\_g1 | egu:105054784 egu:105045796 egu:105034995 egu:105041858 egu:105047446 egu:105055562 egu:105039813 egu:105052855 egu:105056567 egu:105055783 egu:105050113 | 105054784 105045796 105034995 105041858 105047446 105055562 105039813 105052855 105056567 105055783 105050113 |  |
| Pyruvate metabolism | 12 | 104 | 0.306244965711 | 0.999827344552 | c154844\_g1 c134153\_g1 c155055\_g1 c142621\_g1 c169641\_g1 c169723\_g1 c153259\_g1 c104889\_g2 c174574\_g3 c113371\_g2 c173703\_g2 c104889\_g1 | egu:105055979 egu:105043499 egu:105049274 egu:105034407 egu:105055679 egu:105038099 egu:105054827 egu:105054530 egu:105054530 egu:105034969 egu:105034557 egu:105034557 | 105055979 105043499 105049274 105034407 105055679 105038099 105054827 105054530 105054530 105034969 105034557 105034557 |  |
| Glycosphingolipid biosynthesis - globo series | 2 | 11 | 0.313583950845 | 0.999827344552 | c165411\_g1 c151091\_g1 | egu:105035858 egu:105034893 | 105035858 105034893 |  |
| Sphingolipid metabolism | 5 | 39 | 0.334503205847 | 0.999827344552 | c165411\_g1 c143298\_g1 c173984\_g2 c211844\_g1 c143120\_g2 | egu:105035858 egu:105041725 egu:105041599 egu:105041694 egu:105032337 | 105035858 105041725 105041599 105041694 105032337 |  |
| Monobactam biosynthesis | 2 | 12 | 0.346563372314 | 0.999827344552 | c168243\_g1 c113031\_g1 | egu:105049020 egu:105061169 | 105049020 105061169 |  |
| Vitamin B6 metabolism | 2 | 12 | 0.346563372314 | 0.999827344552 | c156465\_g1 c160669\_g1 | egu:105043485 egu:105050909 | 105043485 105050909 |  |
| Other glycan degradation | 3 | 21 | 0.347059904943 | 0.999827344552 | c151091\_g1 c173576\_g2 c162152\_g2 | egu:105034893 egu:105044052 egu:105060392 | 105034893 105044052 105060392 |  |
| Isoquinoline alkaloid biosynthesis | 3 | 21 | 0.347059904943 | 0.999827344552 | c185151\_g1 c159323\_g1 c135610\_g1 | egu:105040940 egu:105048107 egu:105044125 | 105040940 105048107 105044125 |  |
| Nicotinate and nicotinamide metabolism | 3 | 22 | 0.371583043567 | 0.999827344552 | c169825\_g1 c171119\_g1 c105731\_g1 | egu:105047967 egu:105047967 egu:105061383 | 105047967 105047967 105061383 |  |
| Selenocompound metabolism | 3 | 23 | 0.395925186971 | 0.999827344552 | c126847\_g1 c163308\_g1 c134111\_g1 | egu:105051560 egu:105057591 egu:105056640 | 105051560 105057591 105056640 |  |
| Glycosphingolipid biosynthesis - ganglio series | 1 | 5 | 0.420310777019 | 0.999827344552 | c151091\_g1 | egu:105034893 | 105034893 |  |
| Lysine biosynthesis | 2 | 15 | 0.441642429155 | 0.999827344552 | c168243\_g1 c113031\_g1 | egu:105049020 egu:105061169 | 105049020 105061169 |  |
| Phenylalanine, tyrosine and tryptophan biosynthesis | 6 | 56 | 0.454370073369 | 0.999827344552 | c185151\_g1 c166358\_g1 c104600\_g1 c164056\_g1 c141783\_g1 c159323\_g1 | egu:105040940 egu:105046827 egu:105033050 egu:105036454 egu:105033284 egu:105048107 | 105040940 105046827 105033050 105036454 105033284 105048107 |  |
| Glycerolipid metabolism | 8 | 77 | 0.460767964706 | 0.999827344552 | c167963\_g1 c134153\_g1 c158576\_g4 c122861\_g1 c159963\_g1 c165411\_g1 c162118\_g1 c147908\_g1 | egu:105059048 egu:105043499 egu:105055982 egu:105041806 egu:105033796 egu:105035858 egu:105043957 egu:105039298 | 105059048 105043499 105055982 105041806 105033796 105035858 105043957 105039298 |  |
| Biotin metabolism | 3 | 26 | 0.467114927622 | 0.999827344552 | c152279\_g1 c153334\_g1 c164754\_g1 | egu:105051936 egu:105050202 egu:105053059 | 105051936 105050202 105053059 |  |
| Sesquiterpenoid and triterpenoid biosynthesis | 1 | 6 | 0.470681938389 | 0.999827344552 | c152294\_g2 | egu:105055883 | 105055883 |  |
| Arginine and proline metabolism | 6 | 58 | 0.485620215701 | 0.999827344552 | c134153\_g1 c170240\_g1 c124333\_g1 c98584\_g1 c152607\_g1 c159323\_g1 | egu:105043499 egu:105052168 egu:105055151 egu:105056213 egu:105043264 egu:105048107 | 105043499 105052168 105055151 105056213 105043264 105048107 |  |
| Fatty acid degradation | 5 | 48 | 0.492708376244 | 0.999827344552 | c166374\_g1 c172387\_g1 c134153\_g1 c169731\_g1 c162392\_g1 | egu:105034612 egu:105044978 egu:105043499 egu:105039221 egu:105041436 | 105034612 105044978 105043499 105039221 105041436 |  |
| Cysteine and methionine metabolism | 11 | 114 | 0.528089081771 | 0.999827344552 | c134111\_g1 c185151\_g1 c164784\_g1 c168243\_g1 c146595\_g1 c113031\_g1 c167873\_g1 c169641\_g1 c165968\_g2 c168878\_g2 c159323\_g1 | egu:105056640 egu:105040940 egu:105040461 egu:105049020 egu:105057316 egu:105061169 egu:105032148 egu:105055679 egu:105044935 egu:105056476 egu:105048107 | 105056640 105040940 105040461 105049020 105057316 105061169 105032148 105055679 105044935 105056476 105048107 |  |
| Arginine biosynthesis | 4 | 40 | 0.538802082963 | 0.999827344552 | c71483\_g1 c152607\_g1 c159323\_g1 c158088\_g1 | egu:105049882 egu:105043264 egu:105048107 egu:105057795 | 105049882 105043264 105048107 105057795 |  |
| Phenylalanine metabolism | 4 | 40 | 0.538802082963 | 0.999827344552 | c185151\_g1 c159338\_g1 c135610\_g1 c159323\_g1 | egu:105040940 egu:105050858 egu:105044125 egu:105048107 | 105040940 105050858 105044125 105048107 |  |
| One carbon pool by folate | 2 | 19 | 0.555454671981 | 0.999827344552 | c162191\_g1 c163701\_g1 | egu:105045796 egu:105035926 | 105045796 105035926 |  |
| SNARE interactions in vesicular transport | 5 | 52 | 0.558841164917 | 0.999827344552 | c157909\_g1 c146978\_g1 c171131\_g2 c158471\_g1 c237311\_g1 | egu:105060841 egu:105034960 egu:105035227 egu:105036838 egu:105041420 | 105060841 105034960 105035227 105036838 105041420 |  |
| Stilbenoid, diarylheptanoid and gingerol biosynthesis | 2 | 20 | 0.581216822005 | 0.999827344552 | c171016\_g1 c168470\_g1 | egu:105045448 egu:105053765 | 105045448 105053765 |  |
| Oxidative phosphorylation | 15 | 163 | 0.588225948418 | 0.999827344552 | c158106\_g1 c143903\_g1 c158605\_g1 c127506\_g1 c172680\_g2 c166378\_g2 c169665\_g2 c152833\_g1 c174660\_g1 c169929\_g1 c153585\_g1 c152482\_g1 c121911\_g1 c121701\_g1 c164585\_g11 | egu:105051928 egu:105049540 egu:105060092 egu:105043730 egu:105052943 egu:105059182 egu:105044713 egu:105033023 egu:105047063 egu:105048467 egu:105039235 egu:105046559 egu:12079457 egu:105046198 egu:12079395 | 105051928 105049540 105060092 105043730 105052943 105059182 105044713 105033023 105047063 105048467 105039235 105046559 12079457 105046198 12079395 |  |
| Pentose and glucuronate interconversions | 8 | 87 | 0.589819599176 | 0.999827344552 | c171202\_g1 c161417\_g4 c198267\_g1 c155247\_g1 c172683\_g1 c134153\_g1 c149552\_g1 c166827\_g1 | egu:105053029 egu:105051305 egu:105043158 egu:105048825 egu:105033733 egu:105043499 egu:105053626 egu:105057722 | 105053029 105051305 105043158 105048825 105033733 105043499 105053626 105057722 |  |
| Folate biosynthesis | 2 | 22 | 0.629389290315 | 0.999827344552 | c134759\_g1 c153180\_g1 | egu:105050897 egu:105049360 | 105050897 105049360 |  |
| Butanoate metabolism | 2 | 22 | 0.629389290315 | 0.999827344552 | c167954\_g1 c132393\_g1 | egu:105047663 egu:105051539 | 105047663 105051539 |  |
| Glycosylphosphatidylinositol(GPI)-anchor biosynthesis | 2 | 23 | 0.651807224815 | 0.999827344552 | c171137\_g4 c171137\_g1 | egu:105059466 egu:105059466 | 105059466 105059466 |  |
| Propanoate metabolism | 3 | 35 | 0.652903559906 | 0.999827344552 | c153259\_g1 c154844\_g1 c155055\_g1 | egu:105054827 egu:105055979 egu:105049274 | 105054827 105055979 105049274 |  |
| Glutathione metabolism | 9 | 105 | 0.666884921064 | 0.999827344552 | c152562\_g1 c146721\_g1 c160383\_g1 c84836\_g1 c149412\_g1 c124333\_g1 c140974\_g1 c168222\_g2 c160398\_g1 | egu:105057765 egu:105051809 egu:105053549 egu:105055154 egu:105055090 egu:105055151 egu:105055154 egu:105046783 egu:105032151 | 105057765 105051809 105053549 105055154 105055090 105055151 105055154 105046783 105032151 |  |
| Citrate cycle (TCA cycle) | 6 | 71 | 0.669026780721 | 0.999827344552 | c174574\_g3 c113371\_g2 c104889\_g2 c104889\_g1 c169641\_g1 c173703\_g2 | egu:105054530 egu:105034969 egu:105054530 egu:105034557 egu:105055679 egu:105034557 | 105054530 105034969 105054530 105034557 105055679 105034557 |  |
| Ascorbate and aldarate metabolism | 4 | 48 | 0.672537361699 | 0.999827344552 | c134153\_g1 c146721\_g1 c160383\_g1 c149412\_g1 | egu:105043499 egu:105051809 egu:105053549 egu:105055090 | 105043499 105051809 105053549 105055090 |  |
| Circadian rhythm - plant | 5 | 60 | 0.67651164716 | 0.999827344552 | c150398\_g4 c114233\_g1 c154458\_g1 c170910\_g1 c153783\_g1 | egu:105054641 egu:105051422 egu:105036385 egu:105044676 egu:105054824 | 105054641 105051422 105036385 105044676 105054824 |  |
| Thiamine metabolism | 1 | 12 | 0.693253734273 | 0.999827344552 | c166497\_g1 | egu:105035100 | 105035100 |  |
| Galactose metabolism | 5 | 62 | 0.702410645217 | 0.999827344552 | c165411\_g1 c167034\_g2 c150506\_g1 c163786\_g2 c163786\_g1 | egu:105035858 egu:105057305 egu:105043430 egu:105052110 egu:105052110 | 105035858 105057305 105043430 105052110 105052110 |  |
| Pyrimidine metabolism | 11 | 134 | 0.724263491025 | 0.999827344552 | c164585\_g7 c170620\_g6 c173363\_g5 c171508\_g1 c105731\_g1 c173864\_g1 c131939\_g1 c171508\_g2 c163308\_g1 c131571\_g1 c164810\_g1 | egu:105052170 egu:105039067 egu:12079461 egu:105052170 egu:105061383 egu:105055141 egu:105038325 egu:105052170 egu:105057591 egu:105034341 egu:105052573 | 105052170 105039067 12079461 105052170 105061383 105055141 105038325 105052170 105057591 105034341 105052573 |  |
| beta-Alanine metabolism | 3 | 41 | 0.748206894076 | 0.999827344552 | c98584\_g1 c134153\_g1 c135610\_g1 | egu:105056213 egu:105043499 egu:105044125 | 105056213 105043499 105044125 |  |
| mRNA surveillance pathway | 12 | 149 | 0.751617351224 | 0.999827344552 | c158775\_g1 c153337\_g3 c155560\_g1 c145727\_g1 c224125\_g1 c116624\_g1 c155315\_g1 c121960\_g1 c148939\_g1 c852\_g1 c168902\_g1 c165735\_g1 | egu:105056201 egu:105046347 egu:105051301 egu:105038511 egu:105035556 egu:105039096 egu:105036286 egu:105035877 egu:105041328 egu:105048719 egu:105050147 egu:105056201 | 105056201 105046347 105051301 105038511 105035556 105039096 105036286 105035877 105041328 105048719 105050147 105056201 |  |
| Diterpenoid biosynthesis | 2 | 29 | 0.764203835611 | 0.999827344552 | c155288\_g1 c159595\_g1 | egu:105032920 egu:105032920 | 105032920 105032920 |  |
| Sulfur relay system | 1 | 15 | 0.766510210804 | 0.999827344552 | c134759\_g1 | egu:105050897 | 105050897 |  |
| Arachidonic acid metabolism | 1 | 15 | 0.766510210804 | 0.999827344552 | c152562\_g1 | egu:105057765 | 105057765 |  |
| Biosynthesis of amino acids | 24 | 290 | 0.774605598236 | 0.999827344552 | c173582\_g1 c198353\_g1 c185151\_g1 c158088\_g1 c144640\_g1 c164056\_g1 c165968\_g2 c162112\_g2 c152607\_g1 c159323\_g1 c155247\_g1 c168243\_g1 c134111\_g1 c164784\_g1 c166358\_g1 c170240\_g1 c141783\_g1 c154303\_g1 c104600\_g1 c170804\_g2 c162034\_g1 c147541\_g1 c113031\_g1 c71483\_g1 | egu:105056157 egu:105059611 egu:105040940 egu:105057795 egu:105051883 egu:105036454 egu:105044935 egu:105050625 egu:105043264 egu:105048107 egu:105048825 egu:105049020 egu:105056640 egu:105040461 egu:105046827 egu:105052168 egu:105033284 egu:105032039 egu:105033050 egu:105048474 egu:105056168 egu:105057517 egu:105061169 egu:105049882 | 105056157 105059611 105040940 105057795 105051883 105036454 105044935 105050625 105043264 105048107 105048825 105049020 105056640 105040461 105046827 105052168 105033284 105032039 105033050 105048474 105056168 105057517 105061169 105049882 |  |
| Sulfur metabolism | 3 | 43 | 0.774914626897 | 0.999827344552 | c164784\_g1 c163450\_g1 c134111\_g1 | egu:105040461 egu:105046522 egu:105056640 | 105040461 105046522 105056640 |  |
| Lysine degradation | 2 | 30 | 0.779529487834 | 0.999827344552 | c134153\_g1 c158087\_g1 | egu:105043499 egu:105043774 | 105043499 105043774 |  |
| Phosphatidylinositol signaling system | 5 | 71 | 0.800797367546 | 0.999827344552 | c169514\_g5 c158576\_g4 c155451\_g1 c161796\_g1 c132911\_g1 | egu:105048729 egu:105055982 egu:105050983 egu:105049214 egu:105048683 | 105048729 105055982 105050983 105049214 105048683 |  |
| Phenylpropanoid biosynthesis | 13 | 168 | 0.801495081405 | 0.999827344552 | c101133\_g1 c151227\_g1 c168470\_g1 c156209\_g1 c164821\_g1 c171033\_g3 c173942\_g4 c146228\_g1 c147467\_g1 c171016\_g1 c174706\_g1 c165472\_g1 c173060\_g2 | egu:105042952 egu:105054950 egu:105053765 egu:105044629 egu:105044229 egu:105045835 egu:105044265 egu:105045201 egu:105043191 egu:105045448 egu:105034542 egu:105037657 egu:105042390 | 105042952 105054950 105053765 105044629 105044229 105045835 105044265 105045201 105043191 105045448 105034542 105037657 105042390 |  |
| Histidine metabolism | 1 | 17 | 0.805354448196 | 0.999827344552 | c134153\_g1 | egu:105043499 | 105043499 |  |
| AGE-RAGE signaling pathway in diabetic complications | 1 | 17 | 0.805354448196 | 0.999827344552 | c80110\_g1 | egu:105032201 | 105032201 |  |
| Pantothenate and CoA biosynthesis | 2 | 32 | 0.807576213012 | 0.999827344552 | c169825\_g1 c171119\_g1 | egu:105047967 egu:105047967 | 105047967 105047967 |  |
| Ether lipid metabolism | 2 | 32 | 0.807576213012 | 0.999827344552 | c158303\_g2 c224136\_g1 | egu:105033675 egu:105060163 | 105033675 105060163 |  |
| 2-Oxocarboxylic acid metabolism | 4 | 60 | 0.818212954935 | 0.999827344552 | c71483\_g1 c168243\_g1 c159323\_g1 c113031\_g1 | egu:105049882 egu:105049020 egu:105048107 egu:105061169 | 105049882 105049020 105048107 105061169 |  |
| Protein export | 4 | 60 | 0.818212954935 | 0.999827344552 | c112651\_g1 c132523\_g1 c170040\_g1 c123568\_g1 | egu:105050132 egu:105055789 egu:105038252 egu:105044437 | 105050132 105055789 105038252 105044437 |  |
| Terpenoid backbone biosynthesis | 4 | 61 | 0.827504843969 | 0.999827344552 | c166497\_g1 c122896\_g1 c166557\_g2 c166557\_g1 | egu:105035100 egu:105046147 egu:105058545 egu:105058545 | 105035100 105046147 105058545 105058545 |  |
| Phagosome | 8 | 118 | 0.865974236888 | 0.999827344552 | c141394\_g1 c141394\_g3 c158106\_g1 c27236\_g1 c157909\_g1 c141160\_g2 c167530\_g1 c80110\_g1 | egu:105059836 egu:105045457 egu:105051928 egu:105038582 egu:105060841 egu:105043351 egu:105049829 egu:105032201 | 105059836 105045457 105051928 105038582 105060841 105043351 105049829 105032201 |  |
| Zeatin biosynthesis | 1 | 22 | 0.876510345645 | 0.999827344552 | c105237\_g1 | egu:105059645 | 105059645 |  |
| Glycerophospholipid metabolism | 7 | 112 | 0.901241015432 | 0.999827344552 | c167963\_g1 c158576\_g4 c173664\_g2 c224136\_g1 c158303\_g2 c12992\_g1 c162118\_g1 | egu:105059048 egu:105055982 egu:105050243 egu:105060163 egu:105033675 egu:105052307 egu:105043957 | 105059048 105055982 105050243 105060163 105033675 105052307 105043957 |  |
| Alanine, aspartate and glutamate metabolism | 3 | 57 | 0.903060652271 | 0.999827344552 | c71483\_g1 c159323\_g1 c158088\_g1 | egu:105049882 egu:105048107 egu:105057795 | 105049882 105048107 105057795 |  |
| Tryptophan metabolism | 2 | 42 | 0.9052011996 | 0.999827344552 | c134153\_g1 c154527\_g1 | egu:105043499 egu:105036212 | 105043499 105036212 |  |
| Inositol phosphate metabolism | 4 | 72 | 0.905913712276 | 0.999827344552 | c169514\_g5 c161796\_g1 c155451\_g1 c198353\_g1 | egu:105048729 egu:105049214 egu:105050983 egu:105059611 | 105048729 105049214 105050983 105059611 |  |
| alpha-Linolenic acid metabolism | 3 | 58 | 0.909019847187 | 0.999827344552 | c164902\_g1 c168329\_g1 c154400\_g1 | egu:105033309 egu:105041662 egu:105056286 | 105033309 105041662 105056286 |  |
| RNA polymerase | 2 | 45 | 0.923903011849 | 0.999827344552 | c173864\_g1 c173363\_g5 | egu:105055141 egu:12079461 | 105055141 12079461 |  |
| Cutin, suberine and wax biosynthesis | 1 | 28 | 0.928486279249 | 0.999827344552 | c140465\_g1 | egu:105036883 | 105036883 |  |
| Plant hormone signal transduction | 20 | 288 | 0.936152724388 | 0.999827344552 | c71549\_g1 c164712\_g1 c156359\_g1 c137332\_g1 c93769\_g1 c173216\_g1 c166932\_g3 c157547\_g1 c133669\_g1 c164642\_g1 c150424\_g3 c150424\_g2 c164908\_g1 c153105\_g1 c78828\_g1 c158461\_g1 c163658\_g1 c174483\_g5 c151241\_g1 c131808\_g1 | egu:105048010 egu:105033626 egu:105039360 egu:105044738 egu:105042455 egu:105032733 egu:105040625 egu:105042455 egu:105035706 egu:105052996 egu:105032733 egu:105032733 egu:105034824 egu:105042180 egu:105038391 egu:105035543 egu:105041587 egu:105059948 egu:105060959 egu:105051690 | 105048010 105033626 105039360 105044738 105042455 105032733 105040625 105042455 105035706 105052996 105032733 105032733 105034824 105042180 105038391 105035543 105041587 105059948 105060959 105051690 |  |
| Mismatch repair | 2 | 48 | 0.939085500862 | 0.999827344552 | c154238\_g2 c133317\_g1 | egu:105040286 egu:105047264 | 105040286 105047264 |  |
| Valine, leucine and isoleucine degradation | 2 | 48 | 0.939085500862 | 0.999827344552 | c134153\_g1 c152651\_g1 | egu:105043499 egu:105043746 | 105043499 105043746 |  |
| Peroxisome | 6 | 113 | 0.952693066214 | 0.999827344552 | c151165\_g1 c140758\_g1 c172387\_g1 c160079\_g1 c169731\_g1 c152778\_g1 | egu:105059069 egu:105052909 egu:105044978 egu:105044749 egu:105039221 egu:105060838 | 105059069 105052909 105044978 105044749 105039221 105060838 |  |
| Homologous recombination | 2 | 52 | 0.954900742686 | 0.999827344552 | c169317\_g2 c133317\_g1 | egu:105051907 egu:105047264 | 105051907 105047264 |  |
| RNA degradation | 7 | 130 | 0.959129526023 | 0.999827344552 | c121960\_g1 c165808\_g2 c168902\_g1 c159391\_g1 c159391\_g2 c132311\_g1 c166194\_g5 | egu:105035877 egu:105053252 egu:105050147 egu:105040019 egu:105040019 egu:105050714 egu:105060207 | 105035877 105053252 105050147 105040019 105040019 105050714 105060207 |  |
| Flavonoid biosynthesis | 2 | 54 | 0.96125328318 | 0.999827344552 | c171016\_g1 c168470\_g1 | egu:105045448 egu:105053765 | 105045448 105053765 |  |
| Ubiquitin mediated proteolysis | 9 | 159 | 0.961396170634 | 0.999827344552 | c163535\_g1 c154764\_g1 c153783\_g1 c170244\_g1 c167530\_g4 c165970\_g3 c156645\_g1 c173888\_g1 c27396\_g2 | egu:105057710 egu:105056281 egu:105054824 egu:105048331 egu:105055896 egu:105038577 egu:105055105 egu:105041586 egu:105040742 | 105057710 105056281 105054824 105048331 105055896 105038577 105055105 105041586 105040742 |  |
| Biosynthesis of unsaturated fatty acids | 1 | 36 | 0.965494347728 | 0.999827344552 | c137480\_g1 | egu:105061227 | 105061227 |  |
| Endocytosis | 11 | 197 | 0.976430035809 | 0.999827344552 | c168291\_g1 c224136\_g1 c216457\_g1 c165983\_g1 c171234\_g3 c168008\_g1 c157769\_g1 c157769\_g2 c169514\_g5 c164929\_g1 c171346\_g1 | egu:105032379 egu:105060163 egu:105033827 egu:105033827 egu:105033417 egu:105038113 egu:105056969 egu:105044452 egu:105048729 egu:105061464 egu:105056954 | 105032379 105060163 105033827 105033827 105033417 105038113 105056969 105044452 105048729 105061464 105056954 |  |
| Protein processing in endoplasmic reticulum | 15 | 257 | 0.981727829648 | 0.999827344552 | c168095\_g1 c159704\_g1 c154764\_g1 c172850\_g2 c167026\_g8 c140690\_g1 c165970\_g3 c156645\_g1 c114581\_g1 c152923\_g1 c151183\_g1 c167530\_g1 c150102\_g1 c27396\_g2 c171346\_g1 | egu:105050569 egu:105042748 egu:105056281 egu:105050569 egu:105056346 egu:105038834 egu:105038577 egu:105055105 egu:105047872 egu:105042852 egu:105053788 egu:105049829 egu:105042707 egu:105040742 egu:105056954 | 105050569 105042748 105056281 105050569 105056346 105038834 105038577 105055105 105047872 105042852 105053788 105049829 105042707 105040742 105056954 |  |
| Steroid biosynthesis | 1 | 45 | 0.984809016322 | 0.999827344552 | c152294\_g2 | egu:105055883 | 105055883 |  |
| Nucleotide excision repair | 2 | 70 | 0.988835328092 | 0.999827344552 | c154764\_g1 c133317\_g1 | egu:105056281 egu:105047264 | 105056281 105047264 |  |
| N-Glycan biosynthesis | 1 | 55 | 0.99389915201 | 0.999827344552 | c163023\_g1 | egu:105055246 | 105055246 |  |
| Purine metabolism | 8 | 181 | 0.994304164147 | 0.999827344552 | c166749\_g1 c171119\_g1 c173363\_g5 c162518\_g1 c169825\_g1 c144707\_g1 c173864\_g1 c131571\_g1 | egu:105035252 egu:105047967 egu:12079461 egu:105037896 egu:105047967 egu:105046045 egu:105055141 egu:105034341 | 105035252 105047967 12079461 105037896 105047967 105046045 105055141 105034341 |  |
| DNA replication | 1 | 59 | 0.995765298395 | 0.999827344552 | c133317\_g1 | egu:105047264 | 105047264 |  |
| Plant-pathogen interaction | 9 | 220 | 0.998526763613 | 0.999827344552 | c159963\_g1 c27081\_g1 c163371\_g1 c134621\_g1 c174341\_g1 c132911\_g1 c164943\_g2 c168884\_g1 c147918\_g3 | egu:105033796 egu:105057238 egu:105052035 egu:105048941 egu:105055268 egu:105048683 egu:105046686 egu:105052035 egu:105052456 | 105033796 105057238 105052035 105048941 105055268 105048683 105046686 105052035 105052456 |  |
| RNA transport | 8 | 205 | 0.99861301854 | 0.999827344552 | c121960\_g1 c145727\_g1 c144240\_g2 c162257\_g1 c168902\_g1 c852\_g1 c144240\_g1 c166848\_g1 | egu:105035877 egu:105038511 egu:105032882 egu:105037047 egu:105050147 egu:105048719 egu:105032882 egu:105056889 | 105035877 105038511 105032882 105037047 105050147 105048719 105032882 105056889 |  |
| Spliceosome | 9 | 227 | 0.999026133755 | 0.999827344552 | c160059\_g1 c83498\_g1 c157385\_g2 c168382\_g1 c170687\_g2 c155072\_g1 c174892\_g3 c171346\_g1 c174892\_g1 | egu:105043591 egu:105053459 egu:105058417 egu:105043591 egu:105049382 egu:105038221 egu:105039206 egu:105056954 egu:105039206 | 105043591 105053459 105058417 105043591 105049382 105038221 105039206 105056954 105039206 |  |
| Ribosome biogenesis in eukaryotes | 1 | 94 | 0.999827344552 | 0.999827344552 | c150398\_g4 | egu:105054641 | 105054641 |  |
