# Supplementary material for: Physiological and transcriptomic responses of Lanzhou Lily (Lilium davidii, var. unicolor) to cold stress
Source: PLoS One. 2020 Jan 23;15(1):e0227921. doi: 10.1371/journal.pone.0227921 (PMC6977731; doi:10.1371/journal.pone.0227921)
Supplement: S2 Zip — (Zip). CK: control (20°C); LT: low temperature (4°C). (ZIP) [file pone.0227921.s012.zip › S2 Zip/LTvsCK_DOWN/src/egu00010.html]

egu00010


- egu:105050625

- Down regulated genes

c162112\_g2(-1.4107)
- egu:105051883

- Down regulated genes

c144640\_g1(-0.76162)
- egu:105048474

- Down regulated genes

c170804\_g2(-1.4555)

- egu:105043499

- Down regulated genes

c134153\_g1(-0.99629)

- egu:105041436

- Down regulated genes

c162392\_g1(-1.1453)
- egu:105034612

- Down regulated genes

c166374\_g1(-0.65476)

- egu:105034969

- Down regulated genes

c113371\_g2(-0.54621)

- egu:105034969

- Down regulated genes

c113371\_g2(-0.54621)

- egu:105059611

- Down regulated genes

c198353\_g1(-0.79652)

- egu:105038009

- Down regulated genes

c170857\_g1(-0.78343)
- egu:105035321

- Down regulated genes

c154502\_g4(-0.97348)

- egu:105060694

- Down regulated genes

c133070\_g1(-0.6949)

- egu:105060694

- Down regulated genes

c133070\_g1(-0.6949)

- egu:105060694

- Down regulated genes

c133070\_g1(-0.6949)

- egu:105050388

- Down regulated genes

c132652\_g1(-0.67228)

- egu:105056157

- Down regulated genes

c173582\_g1(-1.1307)
- egu:105056168

- Down regulated genes

c162034\_g1(-0.8811)

- egu:105054530

- Down regulated genes

c104889\_g2(-1.7233) c174574\_g3(-2.5042)
- egu:105034557

- Down regulated genes

c104889\_g1(-1.1155) c173703\_g2(-1.3642)

- egu:105049380

- Down regulated genes

c85645\_g1(-1.342)

Close
