# Supplementary material for: Physiological and transcriptomic responses of Lanzhou Lily (Lilium davidii, var. unicolor) to cold stress
Source: PLoS One. 2020 Jan 23;15(1):e0227921. doi: 10.1371/journal.pone.0227921 (PMC6977731; doi:10.1371/journal.pone.0227921)
Supplement: S2 Zip — (Zip). CK: control (20°C); LT: low temperature (4°C). (ZIP) [file pone.0227921.s012.zip › S2 Zip/LTvsCK_DOWN/src/egu00020.html]

egu00020


- egu:105055679

- Down regulated genes

c169641\_g1(-1.9236)

- egu:105034969

- Down regulated genes

c113371\_g2(-0.54621)

- egu:105034969

- Down regulated genes

c113371\_g2(-0.54621)

- egu:105034557

- Down regulated genes

c104889\_g1(-1.1155) c173703\_g2(-1.3642)
- egu:105054530

- Down regulated genes

c174574\_g3(-2.5042) c104889\_g2(-1.7233)

Close
