# Supplementary material for: Physiological and transcriptomic responses of Lanzhou Lily (Lilium davidii, var. unicolor) to cold stress
Source: PLoS One. 2020 Jan 23;15(1):e0227921. doi: 10.1371/journal.pone.0227921 (PMC6977731; doi:10.1371/journal.pone.0227921)
Supplement: S2 Zip — (Zip). CK: control (20°C); LT: low temperature (4°C). (ZIP) [file pone.0227921.s012.zip › S2 Zip/LTvsCK_DOWN/src/egu00040.html]

egu00040


- egu:105033733

- Down regulated genes

c172683\_g1(-0.95612)

- egu:105048825

- Down regulated genes

c155247\_g1(-0.65624)

- egu:105057722

- Down regulated genes

c166827\_g1(-0.85432)

- egu:105033733

- Down regulated genes

c172683\_g1(-0.95612)

- egu:105053029

- Down regulated genes

c171202\_g1(-0.90388)

- egu:105043158

- Down regulated genes

c198267\_g1(-2.7159)
- egu:105053626

- Down regulated genes

c149552\_g1(-3.8561)
- egu:105051305

- Down regulated genes

c161417\_g4(-1.3673)

Close
