# Supplementary material for: Physiological and transcriptomic responses of Lanzhou Lily (Lilium davidii, var. unicolor) to cold stress
Source: PLoS One. 2020 Jan 23;15(1):e0227921. doi: 10.1371/journal.pone.0227921 (PMC6977731; doi:10.1371/journal.pone.0227921)
Supplement: S2 Zip — (Zip). CK: control (20°C); LT: low temperature (4°C). (ZIP) [file pone.0227921.s012.zip › S2 Zip/LTvsCK_DOWN/src/egu00051.html]

egu00051


- egu:105057722

- Down regulated genes

c166827\_g1(-0.85432)

- egu:105049809

- Down regulated genes

c152658\_g1(-0.62512)

- egu:105052174

- Down regulated genes

c157388\_g1(-1.0608)

- egu:105049380

- Down regulated genes

c85645\_g1(-1.342)

- egu:105059611

- Down regulated genes

c198353\_g1(-0.79652)

- egu:105050625

- Down regulated genes

c162112\_g2(-1.4107)
- egu:105051883

- Down regulated genes

c144640\_g1(-0.76162)
- egu:105048474

- Down regulated genes

c170804\_g2(-1.4555)

- egu:105050625

- Down regulated genes

c162112\_g2(-1.4107)
- egu:105051883

- Down regulated genes

c144640\_g1(-0.76162)
- egu:105048474

- Down regulated genes

c170804\_g2(-1.4555)

- egu:105038009

- Down regulated genes

c170857\_g1(-0.78343)
- egu:105035321

- Down regulated genes

c154502\_g4(-0.97348)

- egu:105043452

- Down regulated genes

c168210\_g6(-0.94846) c168210\_g7(-1.4775)

Close
