# Supplementary material for: Physiological and transcriptomic responses of Lanzhou Lily (Lilium davidii, var. unicolor) to cold stress
Source: PLoS One. 2020 Jan 23;15(1):e0227921. doi: 10.1371/journal.pone.0227921 (PMC6977731; doi:10.1371/journal.pone.0227921)
Supplement: S2 Zip — (Zip). CK: control (20°C); LT: low temperature (4°C). (ZIP) [file pone.0227921.s012.zip › S2 Zip/LTvsCK_DOWN/src/egu00052.html]

egu00052


- egu:105035858

- Down regulated genes

c165411\_g1(-0.78931)

- egu:105035858

- Down regulated genes

c165411\_g1(-0.78931)

- egu:105052110

- Down regulated genes

c163786\_g2(-2.2044) c163786\_g1(-2.5261)

- egu:105035858

- Down regulated genes

c165411\_g1(-0.78931)

- egu:105052110

- Down regulated genes

c163786\_g2(-2.2044) c163786\_g1(-2.5261)

- egu:105035858

- Down regulated genes

c165411\_g1(-0.78931)

- egu:105035858

- Down regulated genes

c165411\_g1(-0.78931)

- egu:105035858

- Down regulated genes

c165411\_g1(-0.78931)

- egu:105035858

- Down regulated genes

c165411\_g1(-0.78931)

- egu:105043430

- Down regulated genes

c150506\_g1(-0.88937)

- egu:105057305

- Down regulated genes

c167034\_g2(-2.694)

- egu:105035858

- Down regulated genes

c165411\_g1(-0.78931)

Close
