# Supplementary material for: Physiological and transcriptomic responses of Lanzhou Lily (Lilium davidii, var. unicolor) to cold stress
Source: PLoS One. 2020 Jan 23;15(1):e0227921. doi: 10.1371/journal.pone.0227921 (PMC6977731; doi:10.1371/journal.pone.0227921)
Supplement: S2 Zip — (Zip). CK: control (20°C); LT: low temperature (4°C). (ZIP) [file pone.0227921.s012.zip › S2 Zip/LTvsCK_DOWN/src/egu00053.html]

egu00053


- egu:105043499

- Down regulated genes

c134153\_g1(-0.99629)

- egu:105055090

- Down regulated genes

c149412\_g1(-1.9114)
- egu:105053549

- Down regulated genes

c160383\_g1(-1.0134)
- egu:105051809

- Down regulated genes

c146721\_g1(-0.5903)

Close
