# Supplementary material for: Physiological and transcriptomic responses of Lanzhou Lily (Lilium davidii, var. unicolor) to cold stress
Source: PLoS One. 2020 Jan 23;15(1):e0227921. doi: 10.1371/journal.pone.0227921 (PMC6977731; doi:10.1371/journal.pone.0227921)
Supplement: S2 Zip — (Zip). CK: control (20°C); LT: low temperature (4°C). (ZIP) [file pone.0227921.s012.zip › S2 Zip/LTvsCK_DOWN/src/egu00061.html]

egu00061


- egu:105049664

- Down regulated genes

c104546\_g1(-0.87572)

- egu:105050202

- Down regulated genes

c153334\_g1(-1.2745)

- egu:105051936

- Down regulated genes

c152279\_g1(-0.6466)

- egu:105053059

- Down regulated genes

c164754\_g1(-0.77314)

- egu:105049664

- Down regulated genes

c104546\_g1(-0.87572)

- egu:105049664

- Down regulated genes

c104546\_g1(-0.87572)

- egu:105053059

- Down regulated genes

c164754\_g1(-0.77314)

- egu:105053059

- Down regulated genes

c164754\_g1(-0.77314)

- egu:105053059

- Down regulated genes

c164754\_g1(-0.77314)

- egu:105053059

- Down regulated genes

c164754\_g1(-0.77314)

- egu:105053059

- Down regulated genes

c164754\_g1(-0.77314)

- egu:105053059

- Down regulated genes

c164754\_g1(-0.77314)

- egu:105050202

- Down regulated genes

c153334\_g1(-1.2745)

- egu:105050202

- Down regulated genes

c153334\_g1(-1.2745)

- egu:105050202

- Down regulated genes

c153334\_g1(-1.2745)

- egu:105050202

- Down regulated genes

c153334\_g1(-1.2745)

- egu:105050202

- Down regulated genes

c153334\_g1(-1.2745)

- egu:105050202

- Down regulated genes

c153334\_g1(-1.2745)

- egu:105050202

- Down regulated genes

c153334\_g1(-1.2745)

- egu:105044732

- Down regulated genes

c147420\_g1(-0.9469)

- egu:105055979

- Down regulated genes

c154844\_g1(-1.0304)
- egu:105054827

- Down regulated genes

c153259\_g1(-0.59995)
- egu:105049274

- Down regulated genes

c155055\_g1(-0.67731)

- egu:105053059

- Down regulated genes

c164754\_g1(-0.77314)

- egu:105058326

- Down regulated genes

c159141\_g1(-0.52345)

- egu:105051936

- Down regulated genes

c152279\_g1(-0.6466)

- egu:105051936

- Down regulated genes

c152279\_g1(-0.6466)

- egu:105051936

- Down regulated genes

c152279\_g1(-0.6466)

- egu:105051936

- Down regulated genes

c152279\_g1(-0.6466)

- egu:105051936

- Down regulated genes

c152279\_g1(-0.6466)

- egu:105051936

- Down regulated genes

c152279\_g1(-0.6466)

- egu:105051936

- Down regulated genes

c152279\_g1(-0.6466)

- egu:105044978

- Down regulated genes

c172387\_g1(-1.0816)
- egu:105039221

- Down regulated genes

c169731\_g1(-0.99073)

Close
