# Supplementary material for: Physiological and transcriptomic responses of Lanzhou Lily (Lilium davidii, var. unicolor) to cold stress
Source: PLoS One. 2020 Jan 23;15(1):e0227921. doi: 10.1371/journal.pone.0227921 (PMC6977731; doi:10.1371/journal.pone.0227921)
Supplement: S2 Zip — (Zip). CK: control (20°C); LT: low temperature (4°C). (ZIP) [file pone.0227921.s012.zip › S2 Zip/LTvsCK_DOWN/src/egu00062.html]

egu00062


- egu:105039895

- Down regulated genes

c134603\_g2(-2.1499)
- egu:105040213

- Down regulated genes

c151794\_g1(-1.5653)
- egu:105048315

- Down regulated genes

c164323\_g1(-1.7412)
- egu:105041077

- Down regulated genes

c150017\_g1(-1.0519)
- egu:105044215

- Down regulated genes

c148702\_g1(-0.95101)
- egu:105047165

- Down regulated genes

c164323\_g2(-1.7478)
- egu:105047853

- Down regulated genes

c2875\_g2(-1.116)
- egu:105042530

- Down regulated genes

c2875\_g1(-1.3428)

Close
