# Supplementary material for: Physiological and transcriptomic responses of Lanzhou Lily (Lilium davidii, var. unicolor) to cold stress
Source: PLoS One. 2020 Jan 23;15(1):e0227921. doi: 10.1371/journal.pone.0227921 (PMC6977731; doi:10.1371/journal.pone.0227921)
Supplement: S2 Zip — (Zip). CK: control (20°C); LT: low temperature (4°C). (ZIP) [file pone.0227921.s012.zip › S2 Zip/LTvsCK_DOWN/src/egu00130.html]

egu00130


- egu:105047162

- Down regulated genes

c154629\_g1(-1.3269)

- egu:105060927

- Down regulated genes

c173971\_g3(-1.266)

- egu:105053482

- Down regulated genes

c166982\_g1(-0.61184)

- egu:105055609

- Down regulated genes

c152224\_g1(-0.46463)

- egu:105055609

- Down regulated genes

c152224\_g1(-0.46463)

- egu:105040940

- Down regulated genes

c185151\_g1(-2.1044)

- egu:105035064

- Down regulated genes

c152469\_g1(-0.94741)

- egu:105035064

- Down regulated genes

c152469\_g1(-0.94741)

- egu:105040851

- Down regulated genes

c156623\_g1(-0.57504)

- egu:105041933

- Down regulated genes

c165685\_g1(-0.59226)

- egu:105041933

- Down regulated genes

c165685\_g1(-0.59226)

- egu:105040851

- Down regulated genes

c156623\_g1(-0.57504)

- egu:105035064

- Down regulated genes

c152469\_g1(-0.94741)

- egu:105046456

- Down regulated genes

c159709\_g1(-0.7001)

- egu:105040851

- Down regulated genes

c156623\_g1(-0.57504)

- egu:105040851

- Down regulated genes

c156623\_g1(-0.57504)

- egu:105055609

- Down regulated genes

c152224\_g1(-0.46463)

Close
