# Supplementary material for: Physiological and transcriptomic responses of Lanzhou Lily (Lilium davidii, var. unicolor) to cold stress
Source: PLoS One. 2020 Jan 23;15(1):e0227921. doi: 10.1371/journal.pone.0227921 (PMC6977731; doi:10.1371/journal.pone.0227921)
Supplement: S2 Zip — (Zip). CK: control (20°C); LT: low temperature (4°C). (ZIP) [file pone.0227921.s012.zip › S2 Zip/LTvsCK_DOWN/src/egu00190.html]

egu00190


- egu:105044713

- Down regulated genes

c169665\_g2(-0.62762)

- egu:105046559

- Down regulated genes

c152482\_g1(-0.5397)

- egu:12079395

- Down regulated genes

c164585\_g11(-0.57784)

- egu:105046559

- Down regulated genes

c152482\_g1(-0.5397)
- egu:105044713

- Down regulated genes

c169665\_g2(-0.62762)

- egu:105046559

- Down regulated genes

c152482\_g1(-0.5397)
- egu:12079395

- Down regulated genes

c164585\_g11(-0.57784)
- egu:105044713

- Down regulated genes

c169665\_g2(-0.62762)

- egu:105059182

- Down regulated genes

c166378\_g2(-0.50334)

- egu:105051928

- Down regulated genes

c158106\_g1(-0.5161)
- egu:105049540

- Down regulated genes

c143903\_g1(-1.0137)
- egu:12079457

- Down regulated genes

c121911\_g1(-1.8109)
- egu:105039235

- Down regulated genes

c153585\_g1(-0.58043)
- egu:105047063

- Down regulated genes

c174660\_g1(-1.8074)
- egu:105048467

- Down regulated genes

c169929\_g1(-0.73841)
- egu:105033023

- Down regulated genes

c152833\_g1(-0.5869)
- egu:105046198

- Down regulated genes

c121701\_g1(-0.80225)

- egu:105043730

- Down regulated genes

c127506\_g1(-2.1748)
- egu:105052943

- Down regulated genes

c172680\_g2(-1.5009)

- egu:105060092

- Down regulated genes

c158605\_g1(-0.92593)

- egu:105059182

- Down regulated genes

c166378\_g2(-0.50334)

- egu:12079457

- Down regulated genes

c121911\_g1(-1.8109)

- egu:105033023

- Down regulated genes

c152833\_g1(-0.5869)
- egu:105046198

- Down regulated genes

c121701\_g1(-0.80225)

- egu:105049540

- Down regulated genes

c143903\_g1(-1.0137)

- egu:105047063

- Down regulated genes

c174660\_g1(-1.8074)
- egu:105048467

- Down regulated genes

c169929\_g1(-0.73841)

- egu:105039235

- Down regulated genes

c153585\_g1(-0.58043)

- egu:105051928

- Down regulated genes

c158106\_g1(-0.5161)

Close
