# Supplementary material for: Physiological and transcriptomic responses of Lanzhou Lily (Lilium davidii, var. unicolor) to cold stress
Source: PLoS One. 2020 Jan 23;15(1):e0227921. doi: 10.1371/journal.pone.0227921 (PMC6977731; doi:10.1371/journal.pone.0227921)
Supplement: S2 Zip — (Zip). CK: control (20°C); LT: low temperature (4°C). (ZIP) [file pone.0227921.s012.zip › S2 Zip/LTvsCK_DOWN/src/egu00195.html]

egu00195


- egu:105044241

- Down regulated genes

c48634\_g1(-1.5024)

- egu:105049540

- Down regulated genes

c143903\_g1(-1.0137)

- egu:105056235

- Down regulated genes

c76799\_g1(-0.75248)

- egu:105053658

- Down regulated genes

c153193\_g1(-1.0859)

- egu:105037935

- Down regulated genes

c140011\_g1(-0.56891)

- egu:105044486

- Down regulated genes

c152881\_g1(-1.1873)
- egu:105033747

- Down regulated genes

c158839\_g1(-1.1123) c121905\_g1(-0.91269)

- egu:105044080

- Down regulated genes

c149607\_g1(-0.75073)

- egu:105033023

- Down regulated genes

c152833\_g1(-0.5869)
- egu:105046198

- Down regulated genes

c121701\_g1(-0.80225)

- egu:12079457

- Down regulated genes

c121911\_g1(-1.8109)

- egu:105049872

- Down regulated genes

c237057\_g1(-1.2689)

- egu:12079475

- Down regulated genes

c164923\_g1(-0.71883)

- egu:105049872

- Down regulated genes

c237057\_g1(-1.2689)

- egu:105055143

- Down regulated genes

c84948\_g1(-0.64176)

- egu:105034183

- Down regulated genes

c158296\_g1(-1.6347)
- egu:105034502

- Down regulated genes

c122782\_g1(-0.98115)

- egu:105044080

- Down regulated genes

c149607\_g1(-0.75073)

- egu:105055013

- Down regulated genes

c131998\_g1(-1.5727)

- egu:105043122

- Down regulated genes

c237127\_g1(-1.2003)

- egu:105046752

- Down regulated genes

c198283\_g1(-2.0199)

- egu:105037273

- Down regulated genes

c71692\_g1(-1.4099)

- egu:105046700

- Down regulated genes

c153649\_g1(-1.9264)

- egu:105038844

- Down regulated genes

c123692\_g1(-1.2337)

- egu:105051755

- Down regulated genes

c76010\_g1(-2.4661)

- egu:105047072

- Down regulated genes

c106880\_g1(-1.1832)

- egu:105037794

- Down regulated genes

c134148\_g1(-1.0932)

- egu:105054056

- Down regulated genes

c151822\_g1(-0.88802)

- egu:105056630

- Down regulated genes

c156755\_g1(-1.6191)
- egu:105046935

- Down regulated genes

c84926\_g1(-1.0798)

- egu:105049540

- Down regulated genes

c143903\_g1(-1.0137)
- egu:105033023

- Down regulated genes

c152833\_g1(-0.5869)
- egu:12079457

- Down regulated genes

c121911\_g1(-1.8109)
- egu:105046198

- Down regulated genes

c121701\_g1(-0.80225)

- egu:105038285

- Down regulated genes

c42744\_g1(-1.6943)

Close
