# Supplementary material for: Physiological and transcriptomic responses of Lanzhou Lily (Lilium davidii, var. unicolor) to cold stress
Source: PLoS One. 2020 Jan 23;15(1):e0227921. doi: 10.1371/journal.pone.0227921 (PMC6977731; doi:10.1371/journal.pone.0227921)
Supplement: S2 Zip — (Zip). CK: control (20°C); LT: low temperature (4°C). (ZIP) [file pone.0227921.s012.zip › S2 Zip/LTvsCK_DOWN/src/egu00196.html]

egu00196


- egu:105058558

- Down regulated genes

c149445\_g1(-2.7255)

- egu:105039517

- Down regulated genes

c152435\_g1(-1.4959)

- egu:105053065

- Down regulated genes

c139230\_g1(-1.8832)
- egu:105032432

- Down regulated genes

c211152\_g1(-1.8371)

- egu:105033408

- Down regulated genes

c149517\_g1(-1.8365)

- egu:105058393

- Down regulated genes

c157047\_g1(-2.1391)

- egu:105051572

- Down regulated genes

c174985\_g1(-1.6435)

- egu:105046981

- Down regulated genes

c115694\_g1(-1.7963) c145751\_g1(-2.4189)

- egu:105032321

- Down regulated genes

c155535\_g1(-3.3911)

- egu:105035084

- Down regulated genes

c164577\_g1(-0.9564)

- egu:105032174

- Down regulated genes

c115377\_g1(-4.7458)

- egu:105058244

- Down regulated genes

c153860\_g1(-1.2806)

Close
