# Supplementary material for: Physiological and transcriptomic responses of Lanzhou Lily (Lilium davidii, var. unicolor) to cold stress
Source: PLoS One. 2020 Jan 23;15(1):e0227921. doi: 10.1371/journal.pone.0227921 (PMC6977731; doi:10.1371/journal.pone.0227921)
Supplement: S2 Zip — (Zip). CK: control (20°C); LT: low temperature (4°C). (ZIP) [file pone.0227921.s012.zip › S2 Zip/LTvsCK_DOWN/src/egu00220.html]

egu00220


- egu:105057795

- Down regulated genes

c158088\_g1(-1.3722)

- egu:105043264

- Down regulated genes

c152607\_g1(-0.78732)

- egu:105048107

- Down regulated genes

c159323\_g1(-1.3206)

- egu:105049882

- Down regulated genes

c71483\_g1(-0.61029)

Close
