# Supplementary material for: Physiological and transcriptomic responses of Lanzhou Lily (Lilium davidii, var. unicolor) to cold stress
Source: PLoS One. 2020 Jan 23;15(1):e0227921. doi: 10.1371/journal.pone.0227921 (PMC6977731; doi:10.1371/journal.pone.0227921)
Supplement: S2 Zip — (Zip). CK: control (20°C); LT: low temperature (4°C). (ZIP) [file pone.0227921.s012.zip › S2 Zip/LTvsCK_DOWN/src/egu00230.html]

egu00230


- egu:105055141

- Down regulated genes

c173864\_g1(-0.67726)
- egu:12079461

- Down regulated genes

c173363\_g5(-2.3147)

- egu:105034341

- Down regulated genes

c131571\_g1(-0.92365)

- egu:105034341

- Down regulated genes

c131571\_g1(-0.92365)

- egu:105037896

- Down regulated genes

c162518\_g1(-0.68546)

- egu:105034341

- Down regulated genes

c131571\_g1(-0.92365)

- egu:105035252

- Down regulated genes

c166749\_g1(-0.50829)

- egu:105034341

- Down regulated genes

c131571\_g1(-0.92365)

- egu:105035252

- Down regulated genes

c166749\_g1(-0.50829)

- egu:105034341

- Down regulated genes

c131571\_g1(-0.92365)

- egu:105055141

- Down regulated genes

c173864\_g1(-0.67726)
- egu:12079461

- Down regulated genes

c173363\_g5(-2.3147)

- egu:105037896

- Down regulated genes

c162518\_g1(-0.68546)

- egu:105034341

- Down regulated genes

c131571\_g1(-0.92365)

- egu:105046045

- Down regulated genes

c144707\_g1(-0.60282)

- egu:105047967

- Down regulated genes

c171119\_g1(-1.2334) c169825\_g1(-0.83271)

- egu:105047967

- Down regulated genes

c171119\_g1(-1.2334) c169825\_g1(-0.83271)

Close
