# Supplementary material for: Physiological and transcriptomic responses of Lanzhou Lily (Lilium davidii, var. unicolor) to cold stress
Source: PLoS One. 2020 Jan 23;15(1):e0227921. doi: 10.1371/journal.pone.0227921 (PMC6977731; doi:10.1371/journal.pone.0227921)
Supplement: S2 Zip — (Zip). CK: control (20°C); LT: low temperature (4°C). (ZIP) [file pone.0227921.s012.zip › S2 Zip/LTvsCK_DOWN/src/egu00240.html]

egu00240


- egu:105038325

- Down regulated genes

c131939\_g1(-0.51701)

- egu:105055141

- Down regulated genes

c173864\_g1(-0.67726)
- egu:12079461

- Down regulated genes

c173363\_g5(-2.3147)

- egu:105034341

- Down regulated genes

c131571\_g1(-0.92365)

- egu:105039067

- Down regulated genes

c170620\_g6(-3.9832)
- egu:105052573

- Down regulated genes

c164810\_g1(-0.76842)

- egu:105052170

- Down regulated genes

c164585\_g7(-0.91856) c171508\_g1(-2.5119) c171508\_g2(-2.6276)

- egu:105061383

- Down regulated genes

c105731\_g1(-0.74343)

- egu:105055141

- Down regulated genes

c173864\_g1(-0.67726)
- egu:12079461

- Down regulated genes

c173363\_g5(-2.3147)

- egu:105034341

- Down regulated genes

c131571\_g1(-0.92365)

- egu:105034341

- Down regulated genes

c131571\_g1(-0.92365)

- egu:105034341

- Down regulated genes

c131571\_g1(-0.92365)

- egu:105034341

- Down regulated genes

c131571\_g1(-0.92365)

- egu:105034341

- Down regulated genes

c131571\_g1(-0.92365)

Close
