# Supplementary material for: Physiological and transcriptomic responses of Lanzhou Lily (Lilium davidii, var. unicolor) to cold stress
Source: PLoS One. 2020 Jan 23;15(1):e0227921. doi: 10.1371/journal.pone.0227921 (PMC6977731; doi:10.1371/journal.pone.0227921)
Supplement: S2 Zip — (Zip). CK: control (20°C); LT: low temperature (4°C). (ZIP) [file pone.0227921.s012.zip › S2 Zip/LTvsCK_DOWN/src/egu00260.html]

egu00260


- egu:105036939

- Down regulated genes

c133760\_g1(-0.58953)

- egu:105039298

- Down regulated genes

c147908\_g1(-0.62718)

- egu:105047380

- Down regulated genes

c157902\_g1(-0.69904)
- egu:105053770

- Down regulated genes

c48670\_g1(-0.6809)

- egu:105049020

- Down regulated genes

c168243\_g1(-0.66676)

- egu:105049882

- Down regulated genes

c71483\_g1(-0.61029)

- egu:105061169

- Down regulated genes

c113031\_g1(-1.0116)

- egu:105044125

- Down regulated genes

c135610\_g1(-0.95193)

- egu:105035926

- Down regulated genes

c163701\_g1(-1.0664)

- egu:105049882

- Down regulated genes

c71483\_g1(-0.61029)

- egu:105059577

- Down regulated genes

c132497\_g1(-1.3633)

Close
