# Supplementary material for: Physiological and transcriptomic responses of Lanzhou Lily (Lilium davidii, var. unicolor) to cold stress
Source: PLoS One. 2020 Jan 23;15(1):e0227921. doi: 10.1371/journal.pone.0227921 (PMC6977731; doi:10.1371/journal.pone.0227921)
Supplement: S2 Zip — (Zip). CK: control (20°C); LT: low temperature (4°C). (ZIP) [file pone.0227921.s012.zip › S2 Zip/LTvsCK_DOWN/src/egu00270.html]

egu00270


- egu:105040940

- Down regulated genes

c185151\_g1(-2.1044)

- egu:105044935

- Down regulated genes

c165968\_g2(-0.60953)

- egu:105056476

- Down regulated genes

c168878\_g2(-5.0724)
- egu:105032148

- Down regulated genes

c167873\_g1(-0.68048)

- egu:105057316

- Down regulated genes

c146595\_g1(-1.2756)

- egu:105056640

- Down regulated genes

c134111\_g1(-0.84087)

- egu:105040461

- Down regulated genes

c164784\_g1(-0.84892)

- egu:105048107

- Down regulated genes

c159323\_g1(-1.3206)

- egu:105048107

- Down regulated genes

c159323\_g1(-1.3206)

- egu:105048107

- Down regulated genes

c159323\_g1(-1.3206)

- egu:105056640

- Down regulated genes

c134111\_g1(-0.84087)

- egu:105056640

- Down regulated genes

c134111\_g1(-0.84087)

- egu:105056640

- Down regulated genes

c134111\_g1(-0.84087)

- egu:105056640

- Down regulated genes

c134111\_g1(-0.84087)

- egu:105061169

- Down regulated genes

c113031\_g1(-1.0116)

- egu:105049020

- Down regulated genes

c168243\_g1(-0.66676)

- egu:105055679

- Down regulated genes

c169641\_g1(-1.9236)

Close
