# Supplementary material for: Physiological and transcriptomic responses of Lanzhou Lily (Lilium davidii, var. unicolor) to cold stress
Source: PLoS One. 2020 Jan 23;15(1):e0227921. doi: 10.1371/journal.pone.0227921 (PMC6977731; doi:10.1371/journal.pone.0227921)
Supplement: S2 Zip — (Zip). CK: control (20°C); LT: low temperature (4°C). (ZIP) [file pone.0227921.s012.zip › S2 Zip/LTvsCK_DOWN/src/egu00330.html]

egu00330


- egu:105043264

- Down regulated genes

c152607\_g1(-0.78732)

- egu:105043499

- Down regulated genes

c134153\_g1(-0.99629)

- egu:105043499

- Down regulated genes

c134153\_g1(-0.99629)

- egu:105056213

- Down regulated genes

c98584\_g1(-1.187)

- egu:105056213

- Down regulated genes

c98584\_g1(-1.187)

- egu:105052168

- Down regulated genes

c170240\_g1(-0.60615)

- egu:105052168

- Down regulated genes

c170240\_g1(-0.60615)

- egu:105048107

- Down regulated genes

c159323\_g1(-1.3206)

- egu:105055151

- Down regulated genes

c124333\_g1(-3.6104)

Close
