# Supplementary material for: Physiological and transcriptomic responses of Lanzhou Lily (Lilium davidii, var. unicolor) to cold stress
Source: PLoS One. 2020 Jan 23;15(1):e0227921. doi: 10.1371/journal.pone.0227921 (PMC6977731; doi:10.1371/journal.pone.0227921)
Supplement: S2 Zip — (Zip). CK: control (20°C); LT: low temperature (4°C). (ZIP) [file pone.0227921.s012.zip › S2 Zip/LTvsCK_DOWN/src/egu00350.html]

egu00350


- egu:105048107

- Down regulated genes

c159323\_g1(-1.3206)

- egu:105050858

- Down regulated genes

c159338\_g1(-1.0938)

- egu:105040940

- Down regulated genes

c185151\_g1(-2.1044)

- egu:105044125

- Down regulated genes

c135610\_g1(-0.95193)

- egu:105041436

- Down regulated genes

c162392\_g1(-1.1453)
- egu:105034612

- Down regulated genes

c166374\_g1(-0.65476)

- egu:105044125

- Down regulated genes

c135610\_g1(-0.95193)

Close
