# Supplementary material for: Physiological and transcriptomic responses of Lanzhou Lily (Lilium davidii, var. unicolor) to cold stress
Source: PLoS One. 2020 Jan 23;15(1):e0227921. doi: 10.1371/journal.pone.0227921 (PMC6977731; doi:10.1371/journal.pone.0227921)
Supplement: S2 Zip — (Zip). CK: control (20°C); LT: low temperature (4°C). (ZIP) [file pone.0227921.s012.zip › S2 Zip/LTvsCK_DOWN/src/egu00400.html]

egu00400


- egu:105033284

- Down regulated genes

c141783\_g1(-1.1992)

- egu:105046827

- Down regulated genes

c166358\_g1(-1.0276)

- egu:105046827

- Down regulated genes

c166358\_g1(-1.0276)

- egu:105040940

- Down regulated genes

c185151\_g1(-2.1044)

- egu:105046827

- Down regulated genes

c166358\_g1(-1.0276)

- egu:105046827

- Down regulated genes

c166358\_g1(-1.0276)

- egu:105040940

- Down regulated genes

c185151\_g1(-2.1044)

- egu:105036454

- Down regulated genes

c164056\_g1(-0.83646)

- egu:105048107

- Down regulated genes

c159323\_g1(-1.3206)

- egu:105048107

- Down regulated genes

c159323\_g1(-1.3206)

- egu:105033050

- Down regulated genes

c104600\_g1(-0.82634)

Close
