# Supplementary material for: Physiological and transcriptomic responses of Lanzhou Lily (Lilium davidii, var. unicolor) to cold stress
Source: PLoS One. 2020 Jan 23;15(1):e0227921. doi: 10.1371/journal.pone.0227921 (PMC6977731; doi:10.1371/journal.pone.0227921)
Supplement: S2 Zip — (Zip). CK: control (20°C); LT: low temperature (4°C). (ZIP) [file pone.0227921.s012.zip › S2 Zip/LTvsCK_DOWN/src/egu00460.html]

egu00460


- egu:105042390

- Down regulated genes

c173060\_g2(-1.2692)
- egu:105045201

- Down regulated genes

c146228\_g1(-0.90789)
- egu:105034542

- Down regulated genes

c174706\_g1(-0.9061)
- egu:105044265

- Down regulated genes

c173942\_g4(-2.7483)
- egu:105045835

- Down regulated genes

c171033\_g3(-0.62809)
- egu:105044229

- Down regulated genes

c164821\_g1(-2.5552)

- egu:105042390

- Down regulated genes

c173060\_g2(-1.2692)
- egu:105045201

- Down regulated genes

c146228\_g1(-0.90789)
- egu:105034542

- Down regulated genes

c174706\_g1(-0.9061)
- egu:105044265

- Down regulated genes

c173942\_g4(-2.7483)
- egu:105045835

- Down regulated genes

c171033\_g3(-0.62809)
- egu:105044229

- Down regulated genes

c164821\_g1(-2.5552)

- egu:105042390

- Down regulated genes

c173060\_g2(-1.2692)
- egu:105045201

- Down regulated genes

c146228\_g1(-0.90789)
- egu:105034542

- Down regulated genes

c174706\_g1(-0.9061)
- egu:105044265

- Down regulated genes

c173942\_g4(-2.7483)
- egu:105045835

- Down regulated genes

c171033\_g3(-0.62809)
- egu:105044229

- Down regulated genes

c164821\_g1(-2.5552)

- egu:105042390

- Down regulated genes

c173060\_g2(-1.2692)
- egu:105045201

- Down regulated genes

c146228\_g1(-0.90789)
- egu:105034542

- Down regulated genes

c174706\_g1(-0.9061)
- egu:105044265

- Down regulated genes

c173942\_g4(-2.7483)
- egu:105045835

- Down regulated genes

c171033\_g3(-0.62809)
- egu:105044229

- Down regulated genes

c164821\_g1(-2.5552)

- egu:105042390

- Down regulated genes

c173060\_g2(-1.2692)
- egu:105045201

- Down regulated genes

c146228\_g1(-0.90789)
- egu:105034542

- Down regulated genes

c174706\_g1(-0.9061)
- egu:105044265

- Down regulated genes

c173942\_g4(-2.7483)
- egu:105045835

- Down regulated genes

c171033\_g3(-0.62809)
- egu:105044229

- Down regulated genes

c164821\_g1(-2.5552)

Close
