# Supplementary material for: Physiological and transcriptomic responses of Lanzhou Lily (Lilium davidii, var. unicolor) to cold stress
Source: PLoS One. 2020 Jan 23;15(1):e0227921. doi: 10.1371/journal.pone.0227921 (PMC6977731; doi:10.1371/journal.pone.0227921)
Supplement: S2 Zip — (Zip). CK: control (20°C); LT: low temperature (4°C). (ZIP) [file pone.0227921.s012.zip › S2 Zip/LTvsCK_DOWN/src/egu00500.html]

egu00500


- egu:105045201

- Down regulated genes

c146228\_g1(-0.90789)
- egu:105045835

- Down regulated genes

c171033\_g3(-0.62809)
- egu:105042390

- Down regulated genes

c173060\_g2(-1.2692)
- egu:105044265

- Down regulated genes

c173942\_g4(-2.7483)
- egu:105044229

- Down regulated genes

c164821\_g1(-2.5552)
- egu:105034542

- Down regulated genes

c174706\_g1(-0.9061)

- egu:105045201

- Down regulated genes

c146228\_g1(-0.90789)
- egu:105045835

- Down regulated genes

c171033\_g3(-0.62809)
- egu:105042390

- Down regulated genes

c173060\_g2(-1.2692)
- egu:105044265

- Down regulated genes

c173942\_g4(-2.7483)
- egu:105044229

- Down regulated genes

c164821\_g1(-2.5552)
- egu:105034542

- Down regulated genes

c174706\_g1(-0.9061)

- egu:105052110

- Down regulated genes

c163786\_g2(-2.2044) c163786\_g1(-2.5261)

- egu:105049809

- Down regulated genes

c152658\_g1(-0.62512)

- egu:105052110

- Down regulated genes

c163786\_g2(-2.2044) c163786\_g1(-2.5261)

- egu:105045201

- Down regulated genes

c146228\_g1(-0.90789)
- egu:105045835

- Down regulated genes

c171033\_g3(-0.62809)
- egu:105042390

- Down regulated genes

c173060\_g2(-1.2692)
- egu:105044265

- Down regulated genes

c173942\_g4(-2.7483)
- egu:105044229

- Down regulated genes

c164821\_g1(-2.5552)
- egu:105034542

- Down regulated genes

c174706\_g1(-0.9061)

- egu:105044696

- Down regulated genes

c150214\_g1(-2.8126)

- egu:105049657

- Down regulated genes

c170862\_g1(-2.0698)

- egu:105047967

- Down regulated genes

c171119\_g1(-1.2334) c169825\_g1(-0.83271)

- egu:105042109

- Down regulated genes

c156351\_g3(-2.0957) c172732\_g1(-1.1841)
- egu:105050772

- Down regulated genes

c167282\_g1(-1.1978)
- egu:105048485

- Down regulated genes

c156351\_g1(-2.1618) c156351\_g5(-2.335)
- egu:105053174

- Down regulated genes

c156351\_g6(-3.3926)
- egu:105041389

- Down regulated genes

c168535\_g1(-0.98516)
- egu:105039195

- Down regulated genes

c172165\_g1(-2.7115)

- egu:105040139

- Down regulated genes

c104638\_g1(-0.74546)

- egu:105060694

- Down regulated genes

c133070\_g1(-0.6949)

- egu:105042109

- Down regulated genes

c156351\_g3(-2.0957) c172732\_g1(-1.1841)
- egu:105050772

- Down regulated genes

c167282\_g1(-1.1978)
- egu:105048485

- Down regulated genes

c156351\_g1(-2.1618) c156351\_g5(-2.335)
- egu:105043601

- Down regulated genes

c166462\_g1(-1.9811)
- egu:105053174

- Down regulated genes

c156351\_g6(-3.3926)
- egu:105041389

- Down regulated genes

c168535\_g1(-0.98516)
- egu:105039195

- Down regulated genes

c172165\_g1(-2.7115)

- egu:105042381

- Down regulated genes

c169016\_g1(-0.80681)

- egu:105044696

- Down regulated genes

c150214\_g1(-2.8126)

Close
