# Supplementary material for: Physiological and transcriptomic responses of Lanzhou Lily (Lilium davidii, var. unicolor) to cold stress
Source: PLoS One. 2020 Jan 23;15(1):e0227921. doi: 10.1371/journal.pone.0227921 (PMC6977731; doi:10.1371/journal.pone.0227921)
Supplement: S2 Zip — (Zip). CK: control (20°C); LT: low temperature (4°C). (ZIP) [file pone.0227921.s012.zip › S2 Zip/LTvsCK_DOWN/src/egu00520.html]

egu00520


- egu:105034893

- Down regulated genes

c151091\_g1(-0.88978)

- egu:105060320

- Down regulated genes

c123366\_g1(-0.81618)

- egu:105049809

- Down regulated genes

c152658\_g1(-0.62512)

- egu:105060320

- Down regulated genes

c123366\_g1(-0.81618)

- egu:105054161

- Down regulated genes

c149405\_g1(-0.52082)

- egu:105054161

- Down regulated genes

c149405\_g1(-0.52082)

- egu:105039344

- Down regulated genes

c158447\_g1(-0.88527)

- egu:105040562

- Down regulated genes

c133303\_g2(-0.9279)

- egu:105043430

- Down regulated genes

c150506\_g1(-0.88937)

- egu:105052174

- Down regulated genes

c157388\_g1(-1.0608)

- egu:105043452

- Down regulated genes

c168210\_g7(-1.4775) c168210\_g6(-0.94846)

- egu:105050888

- Down regulated genes

c160287\_g1(-1.0826)

- egu:105060694

- Down regulated genes

c133070\_g1(-0.6949)

- egu:105032401

- Down regulated genes

c20180\_g1(-1.6143)

- egu:105040139

- Down regulated genes

c104638\_g1(-0.74546)

- egu:105060491

- Down regulated genes

c98676\_g1(-1.4797)
- egu:105038864

- Down regulated genes

c159732\_g1(-0.61507)
- egu:105056575

- Down regulated genes

c143878\_g1(-0.636)
- egu:105058120

- Down regulated genes

c227079\_g1(-4.742)

- egu:105056534

- Down regulated genes

c170497\_g1(-1.2834)

Close
