# Supplementary material for: Physiological and transcriptomic responses of Lanzhou Lily (Lilium davidii, var. unicolor) to cold stress
Source: PLoS One. 2020 Jan 23;15(1):e0227921. doi: 10.1371/journal.pone.0227921 (PMC6977731; doi:10.1371/journal.pone.0227921)
Supplement: S2 Zip — (Zip). CK: control (20°C); LT: low temperature (4°C). (ZIP) [file pone.0227921.s012.zip › S2 Zip/LTvsCK_DOWN/src/egu00531.html]

egu00531


- egu:105033054

- Down regulated genes

c160082\_g2(-1.7354)

- egu:105034893

- Down regulated genes

c151091\_g1(-0.88978)

- egu:105057579

- Down regulated genes

c168498\_g1(-1.4493)

- egu:105034893

- Down regulated genes

c151091\_g1(-0.88978)

- egu:105057579

- Down regulated genes

c168498\_g1(-1.4493)

- egu:105057579

- Down regulated genes

c168498\_g1(-1.4493)

- egu:105033054

- Down regulated genes

c160082\_g2(-1.7354)

- egu:105034893

- Down regulated genes

c151091\_g1(-0.88978)

- egu:105034893

- Down regulated genes

c151091\_g1(-0.88978)

Close
