# Supplementary material for: Physiological and transcriptomic responses of Lanzhou Lily (Lilium davidii, var. unicolor) to cold stress
Source: PLoS One. 2020 Jan 23;15(1):e0227921. doi: 10.1371/journal.pone.0227921 (PMC6977731; doi:10.1371/journal.pone.0227921)
Supplement: S2 Zip — (Zip). CK: control (20°C); LT: low temperature (4°C). (ZIP) [file pone.0227921.s012.zip › S2 Zip/LTvsCK_DOWN/src/egu00561.html]

egu00561


- egu:105041806

- Down regulated genes

c122861\_g1(-0.81837)

- egu:105043957

- Down regulated genes

c162118\_g1(-0.64519)
- egu:105059048

- Down regulated genes

c167963\_g1(-0.67401)

- egu:105033796

- Down regulated genes

c159963\_g1(-1.0636)

- egu:105043499

- Down regulated genes

c134153\_g1(-0.99629)

- egu:105039298

- Down regulated genes

c147908\_g1(-0.62718)

- egu:105035858

- Down regulated genes

c165411\_g1(-0.78931)

- egu:105055982

- Down regulated genes

c158576\_g4(-2.0092)

Close
