# Supplementary material for: Physiological and transcriptomic responses of Lanzhou Lily (Lilium davidii, var. unicolor) to cold stress
Source: PLoS One. 2020 Jan 23;15(1):e0227921. doi: 10.1371/journal.pone.0227921 (PMC6977731; doi:10.1371/journal.pone.0227921)
Supplement: S2 Zip — (Zip). CK: control (20°C); LT: low temperature (4°C). (ZIP) [file pone.0227921.s012.zip › S2 Zip/LTvsCK_DOWN/src/egu00562.html]

egu00562


- egu:105050983

- Down regulated genes

c155451\_g1(-0.59584)

- egu:105050983

- Down regulated genes

c155451\_g1(-0.59584)

- egu:105050983

- Down regulated genes

c155451\_g1(-0.59584)

- egu:105049214

- Down regulated genes

c161796\_g1(-0.80588)

- egu:105049214

- Down regulated genes

c161796\_g1(-0.80588)

- egu:105048729

- Down regulated genes

c169514\_g5(-1.5841)

- egu:105049214

- Down regulated genes

c161796\_g1(-0.80588)

- egu:105059611

- Down regulated genes

c198353\_g1(-0.79652)

Close
