# Supplementary material for: Physiological and transcriptomic responses of Lanzhou Lily (Lilium davidii, var. unicolor) to cold stress
Source: PLoS One. 2020 Jan 23;15(1):e0227921. doi: 10.1371/journal.pone.0227921 (PMC6977731; doi:10.1371/journal.pone.0227921)
Supplement: S2 Zip — (Zip). CK: control (20°C); LT: low temperature (4°C). (ZIP) [file pone.0227921.s012.zip › S2 Zip/LTvsCK_DOWN/src/egu00564.html]

egu00564


- egu:105050243

- Down regulated genes

c173664\_g2(-0.82721)

- egu:105050243

- Down regulated genes

c173664\_g2(-0.82721)

- egu:105055982

- Down regulated genes

c158576\_g4(-2.0092)

- egu:105060163

- Down regulated genes

c224136\_g1(-0.74326)
- egu:105033675

- Down regulated genes

c158303\_g2(-0.80153)

- egu:105060163

- Down regulated genes

c224136\_g1(-0.74326)
- egu:105033675

- Down regulated genes

c158303\_g2(-0.80153)

- egu:105043957

- Down regulated genes

c162118\_g1(-0.64519)
- egu:105059048

- Down regulated genes

c167963\_g1(-0.67401)

- egu:105052307

- Down regulated genes

c12992\_g1(-1.8958)

- egu:105059048

- Down regulated genes

c167963\_g1(-0.67401)

Close
