# Supplementary material for: Physiological and transcriptomic responses of Lanzhou Lily (Lilium davidii, var. unicolor) to cold stress
Source: PLoS One. 2020 Jan 23;15(1):e0227921. doi: 10.1371/journal.pone.0227921 (PMC6977731; doi:10.1371/journal.pone.0227921)
Supplement: S2 Zip — (Zip). CK: control (20°C); LT: low temperature (4°C). (ZIP) [file pone.0227921.s012.zip › S2 Zip/LTvsCK_DOWN/src/egu00600.html]

egu00600


- egu:105041599

- Down regulated genes

c173984\_g2(-2.8573)

- egu:105041599

- Down regulated genes

c173984\_g2(-2.8573)

- egu:105041725

- Down regulated genes

c143298\_g1(-2.182)
- egu:105041694

- Down regulated genes

c211844\_g1(-1.0079)

- egu:105041725

- Down regulated genes

c143298\_g1(-2.182)
- egu:105041694

- Down regulated genes

c211844\_g1(-1.0079)

- egu:105035858

- Down regulated genes

c165411\_g1(-0.78931)

- egu:105041599

- Down regulated genes

c173984\_g2(-2.8573)

- egu:105032337

- Down regulated genes

c143120\_g2(-1.313)

- egu:105032337

- Down regulated genes

c143120\_g2(-1.313)

Close
