# Supplementary material for: Physiological and transcriptomic responses of Lanzhou Lily (Lilium davidii, var. unicolor) to cold stress
Source: PLoS One. 2020 Jan 23;15(1):e0227921. doi: 10.1371/journal.pone.0227921 (PMC6977731; doi:10.1371/journal.pone.0227921)
Supplement: S2 Zip — (Zip). CK: control (20°C); LT: low temperature (4°C). (ZIP) [file pone.0227921.s012.zip › S2 Zip/LTvsCK_DOWN/src/egu00630.html]

egu00630


- egu:105047663

- Down regulated genes

c167954\_g1(-0.54023)
- egu:105051539

- Down regulated genes

c132393\_g1(-0.51805)

- egu:105055679

- Down regulated genes

c169641\_g1(-1.9236)

- egu:105039298

- Down regulated genes

c147908\_g1(-0.62718)

- egu:105048437

- Down regulated genes

c168133\_g3(-1.5362)

- egu:105051428

- Down regulated genes

c153630\_g1(-1.15)

- egu:105047380

- Down regulated genes

c157902\_g1(-0.69904)
- egu:105053770

- Down regulated genes

c48670\_g1(-0.6809)

- egu:105035926

- Down regulated genes

c163701\_g1(-1.0664)
- egu:105059577

- Down regulated genes

c132497\_g1(-1.3633)

- egu:105049882

- Down regulated genes

c71483\_g1(-0.61029)

- egu:105057601

- Down regulated genes

c137804\_g1(-0.76992)

- egu:105057795

- Down regulated genes

c158088\_g1(-1.3722)

Close
