# Supplementary material for: Physiological and transcriptomic responses of Lanzhou Lily (Lilium davidii, var. unicolor) to cold stress
Source: PLoS One. 2020 Jan 23;15(1):e0227921. doi: 10.1371/journal.pone.0227921 (PMC6977731; doi:10.1371/journal.pone.0227921)
Supplement: S2 Zip — (Zip). CK: control (20°C); LT: low temperature (4°C). (ZIP) [file pone.0227921.s012.zip › S2 Zip/LTvsCK_DOWN/src/egu00670.html]

egu00670


- egu:105035926

- Down regulated genes

c163701\_g1(-1.0664)

- egu:105045796

- Down regulated genes

c162191\_g1(-0.70144)

- egu:105035926

- Down regulated genes

c163701\_g1(-1.0664)

Close
