# Supplementary material for: Physiological and transcriptomic responses of Lanzhou Lily (Lilium davidii, var. unicolor) to cold stress
Source: PLoS One. 2020 Jan 23;15(1):e0227921. doi: 10.1371/journal.pone.0227921 (PMC6977731; doi:10.1371/journal.pone.0227921)
Supplement: S2 Zip — (Zip). CK: control (20°C); LT: low temperature (4°C). (ZIP) [file pone.0227921.s012.zip › S2 Zip/LTvsCK_DOWN/src/egu00710.html]

egu00710


- egu:105051883

- Down regulated genes

c144640\_g1(-0.76162)
- egu:105050625

- Down regulated genes

c162112\_g2(-1.4107)
- egu:105048474

- Down regulated genes

c170804\_g2(-1.4555)

- egu:105046280

- Down regulated genes

c158889\_g1(-0.8565)

- egu:105057517

- Down regulated genes

c147541\_g1(-1.0111)
- egu:105032039

- Down regulated genes

c154303\_g1(-1.424)

- egu:105043976

- Down regulated genes

c164307\_g1(-0.79941)

- egu:105048825

- Down regulated genes

c155247\_g1(-0.65624)

- egu:105038009

- Down regulated genes

c170857\_g1(-0.78343)
- egu:105035321

- Down regulated genes

c154502\_g4(-0.97348)

- egu:105051883

- Down regulated genes

c144640\_g1(-0.76162)
- egu:105050625

- Down regulated genes

c162112\_g2(-1.4107)
- egu:105048474

- Down regulated genes

c170804\_g2(-1.4555)

- egu:105059611

- Down regulated genes

c198353\_g1(-0.79652)

- egu:105042746

- Down regulated genes

c121900\_g1(-1.4168)
- egu:105060347

- Down regulated genes

c160123\_g1(-1.0927)

- egu:105056168

- Down regulated genes

c162034\_g1(-0.8811)
- egu:105056157

- Down regulated genes

c173582\_g1(-1.1307)

- egu:105048437

- Down regulated genes

c168133\_g3(-1.5362)

- egu:105038099

- Down regulated genes

c169723\_g1(-0.94359)

- egu:105055679

- Down regulated genes

c169641\_g1(-1.9236)

- egu:105048107

- Down regulated genes

c159323\_g1(-1.3206)

- egu:105055679

- Down regulated genes

c169641\_g1(-1.9236)

- egu:105049882

- Down regulated genes

c71483\_g1(-0.61029)

- egu:105049882

- Down regulated genes

c71483\_g1(-0.61029)

- egu:105038099

- Down regulated genes

c169723\_g1(-0.94359)

- egu:105054530

- Down regulated genes

c174574\_g3(-2.5042) c104889\_g2(-1.7233)
- egu:105034557

- Down regulated genes

c173703\_g2(-1.3642) c104889\_g1(-1.1155)

Close
