# Supplementary material for: Physiological and transcriptomic responses of Lanzhou Lily (Lilium davidii, var. unicolor) to cold stress
Source: PLoS One. 2020 Jan 23;15(1):e0227921. doi: 10.1371/journal.pone.0227921 (PMC6977731; doi:10.1371/journal.pone.0227921)
Supplement: S2 Zip — (Zip). CK: control (20°C); LT: low temperature (4°C). (ZIP) [file pone.0227921.s012.zip › S2 Zip/LTvsCK_DOWN/src/egu00740.html]

egu00740


- egu:105060549

- Down regulated genes

c166380\_g2(-0.48408)
- egu:105045732

- Down regulated genes

c164015\_g2(-1.0982)

- egu:105060549

- Down regulated genes

c166380\_g2(-0.48408)
- egu:105045732

- Down regulated genes

c164015\_g2(-1.0982)

- egu:105051526

- Down regulated genes

c154382\_g1(-0.80992)

Close
