# Supplementary material for: Physiological and transcriptomic responses of Lanzhou Lily (Lilium davidii, var. unicolor) to cold stress
Source: PLoS One. 2020 Jan 23;15(1):e0227921. doi: 10.1371/journal.pone.0227921 (PMC6977731; doi:10.1371/journal.pone.0227921)
Supplement: S2 Zip — (Zip). CK: control (20°C); LT: low temperature (4°C). (ZIP) [file pone.0227921.s012.zip › S2 Zip/LTvsCK_DOWN/src/egu00860.html]

egu00860


- egu:105036097

- Down regulated genes

c185147\_g2(-1.0982)

- egu:105036097

- Down regulated genes

c185147\_g2(-1.0982)

- egu:105036097

- Down regulated genes

c185147\_g2(-1.0982)

- egu:105059913

- Down regulated genes

c152307\_g1(-0.77696)

- egu:105044579

- Down regulated genes

c155686\_g1(-0.65921)

- egu:105035938

- Down regulated genes

c123480\_g1(-1.3599)

- egu:105057582

- Down regulated genes

c169028\_g1(-0.70656)
- egu:105040656

- Down regulated genes

c133188\_g1(-0.87542)

- egu:105035555

- Down regulated genes

c157850\_g1(-0.78203)

- egu:105044798

- Down regulated genes

c165450\_g1(-1.0571)
- egu:105040768

- Down regulated genes

c168519\_g1(-1.1377)

- egu:105044798

- Down regulated genes

c165450\_g1(-1.0571)
- egu:105040768

- Down regulated genes

c168519\_g1(-1.1377)

- egu:105049221

- Down regulated genes

c71809\_g1(-1.2195)

- egu:105051026

- Down regulated genes

c167743\_g1(-0.91681)

- egu:105054529

- Down regulated genes

c167947\_g1(-2.8328) c170780\_g1(-0.95455)

- egu:105052855

- Down regulated genes

c161205\_g1(-0.80345)

- egu:105037930

- Down regulated genes

c71670\_g1(-0.58079)

- egu:105044579

- Down regulated genes

c155686\_g1(-0.65921)

- egu:105035618

- Down regulated genes

c134612\_g1(-0.62277)

- egu:105035618

- Down regulated genes

c134612\_g1(-0.62277)

- egu:105037930

- Down regulated genes

c71670\_g1(-0.58079)

- egu:105044579

- Down regulated genes

c155686\_g1(-0.65921)

- egu:105044579

- Down regulated genes

c155686\_g1(-0.65921)

- egu:105058545

- Down regulated genes

c166557\_g2(-1.5787) c166557\_g1(-1.7086)

- egu:105058545

- Down regulated genes

c166557\_g2(-1.5787) c166557\_g1(-1.7086)

- egu:105035555

- Down regulated genes

c157850\_g1(-0.78203)

Close
