# Supplementary material for: Physiological and transcriptomic responses of Lanzhou Lily (Lilium davidii, var. unicolor) to cold stress
Source: PLoS One. 2020 Jan 23;15(1):e0227921. doi: 10.1371/journal.pone.0227921 (PMC6977731; doi:10.1371/journal.pone.0227921)
Supplement: S2 Zip — (Zip). CK: control (20°C); LT: low temperature (4°C). (ZIP) [file pone.0227921.s012.zip › S2 Zip/LTvsCK_DOWN/src/egu00900.html]

egu00900


- egu:105035100

- Down regulated genes

c166497\_g1(-1.4686)

- egu:105046147

- Down regulated genes

c122896\_g1(-0.75457)

- egu:105046147

- Down regulated genes

c122896\_g1(-0.75457)

- egu:105058545

- Down regulated genes

c166557\_g2(-1.5787) c166557\_g1(-1.7086)

Close
