# Supplementary material for: Physiological and transcriptomic responses of Lanzhou Lily (Lilium davidii, var. unicolor) to cold stress
Source: PLoS One. 2020 Jan 23;15(1):e0227921. doi: 10.1371/journal.pone.0227921 (PMC6977731; doi:10.1371/journal.pone.0227921)
Supplement: S2 Zip — (Zip). CK: control (20°C); LT: low temperature (4°C). (ZIP) [file pone.0227921.s012.zip › S2 Zip/LTvsCK_DOWN/src/egu00906.html]

egu00906


- egu:105057721

- Down regulated genes

c166298\_g1(-0.77074)

- egu:105038832

- Down regulated genes

c166072\_g1(-1.2063)

- egu:105038832

- Down regulated genes

c166072\_g1(-1.2063)

- egu:105057721

- Down regulated genes

c166298\_g1(-0.77074)

- egu:105056650

- Down regulated genes

c163774\_g1(-0.59036)

- egu:105056650

- Down regulated genes

c163774\_g1(-0.59036)

- egu:105058894

- Down regulated genes

c159032\_g1(-1.1839)

- egu:105058894

- Down regulated genes

c159032\_g1(-1.1839)

- egu:105056650

- Down regulated genes

c163774\_g1(-0.59036)

- egu:105056650

- Down regulated genes

c163774\_g1(-0.59036)

- egu:105038852

- Down regulated genes

c137214\_g2(-0.74276)

- egu:105058894

- Down regulated genes

c159032\_g1(-1.1839)

- egu:105058894

- Down regulated genes

c159032\_g1(-1.1839)

Close
