# Supplementary material for: Physiological and transcriptomic responses of Lanzhou Lily (Lilium davidii, var. unicolor) to cold stress
Source: PLoS One. 2020 Jan 23;15(1):e0227921. doi: 10.1371/journal.pone.0227921 (PMC6977731; doi:10.1371/journal.pone.0227921)
Supplement: S2 Zip — (Zip). CK: control (20°C); LT: low temperature (4°C). (ZIP) [file pone.0227921.s012.zip › S2 Zip/LTvsCK_DOWN/src/egu00910.html]

egu00910


- egu:105048068

- Down regulated genes

c115915\_g1(-1.2309) c145787\_g1(-1.3529)
- egu:105052122

- Down regulated genes

c142597\_g1(-0.90706)

- egu:105033813

- Down regulated genes

c185512\_g1(-0.64118)

- egu:105033813

- Down regulated genes

c185512\_g1(-0.64118)

- egu:105057795

- Down regulated genes

c158088\_g1(-1.3722)

- egu:105057601

- Down regulated genes

c137804\_g1(-0.76992)

Close
