# Supplementary material for: Physiological and transcriptomic responses of Lanzhou Lily (Lilium davidii, var. unicolor) to cold stress
Source: PLoS One. 2020 Jan 23;15(1):e0227921. doi: 10.1371/journal.pone.0227921 (PMC6977731; doi:10.1371/journal.pone.0227921)
Supplement: S2 Zip — (Zip). CK: control (20°C); LT: low temperature (4°C). (ZIP) [file pone.0227921.s012.zip › S2 Zip/LTvsCK_DOWN/src/egu00940.html]

egu00940


- egu:105042390

- Down regulated genes

c173060\_g2(-1.2692)
- egu:105045201

- Down regulated genes

c146228\_g1(-0.90789)
- egu:105034542

- Down regulated genes

c174706\_g1(-0.9061)
- egu:105044265

- Down regulated genes

c173942\_g4(-2.7483)
- egu:105045835

- Down regulated genes

c171033\_g3(-0.62809)
- egu:105044229

- Down regulated genes

c164821\_g1(-2.5552)

- egu:105045448

- Down regulated genes

c171016\_g1(-1.8154)
- egu:105053765

- Down regulated genes

c168470\_g1(-3.8645)

- egu:105045448

- Down regulated genes

c171016\_g1(-1.8154)
- egu:105053765

- Down regulated genes

c168470\_g1(-3.8645)

- egu:105045448

- Down regulated genes

c171016\_g1(-1.8154)
- egu:105053765

- Down regulated genes

c168470\_g1(-3.8645)

- egu:105054950

- Down regulated genes

c151227\_g1(-Inf)

- egu:105054950

- Down regulated genes

c151227\_g1(-Inf)

- egu:105045448

- Down regulated genes

c171016\_g1(-1.8154)
- egu:105053765

- Down regulated genes

c168470\_g1(-3.8645)

- egu:105042952

- Down regulated genes

c101133\_g1(-6.3517)
- egu:105043191

- Down regulated genes

c147467\_g1(-2.0691)
- egu:105044629

- Down regulated genes

c156209\_g1(-1.6383)

- egu:105037657

- Down regulated genes

c165472\_g1(-1.2074)

- egu:105037657

- Down regulated genes

c165472\_g1(-1.2074)

- egu:105037657

- Down regulated genes

c165472\_g1(-1.2074)

- egu:105037657

- Down regulated genes

c165472\_g1(-1.2074)

- egu:105037657

- Down regulated genes

c165472\_g1(-1.2074)

- egu:105042952

- Down regulated genes

c101133\_g1(-6.3517)
- egu:105043191

- Down regulated genes

c147467\_g1(-2.0691)
- egu:105044629

- Down regulated genes

c156209\_g1(-1.6383)

- egu:105042952

- Down regulated genes

c101133\_g1(-6.3517)
- egu:105043191

- Down regulated genes

c147467\_g1(-2.0691)
- egu:105044629

- Down regulated genes

c156209\_g1(-1.6383)

- egu:105042952

- Down regulated genes

c101133\_g1(-6.3517)
- egu:105043191

- Down regulated genes

c147467\_g1(-2.0691)
- egu:105044629

- Down regulated genes

c156209\_g1(-1.6383)

- egu:105054950

- Down regulated genes

c151227\_g1(-Inf)

- egu:105054950

- Down regulated genes

c151227\_g1(-Inf)

- egu:105054950

- Down regulated genes

c151227\_g1(-Inf)

- egu:105054950

- Down regulated genes

c151227\_g1(-Inf)

Close
