# Supplementary material for: Physiological and transcriptomic responses of Lanzhou Lily (Lilium davidii, var. unicolor) to cold stress
Source: PLoS One. 2020 Jan 23;15(1):e0227921. doi: 10.1371/journal.pone.0227921 (PMC6977731; doi:10.1371/journal.pone.0227921)
Supplement: S2 Zip — (Zip). CK: control (20°C); LT: low temperature (4°C). (ZIP) [file pone.0227921.s012.zip › S2 Zip/LTvsCK_DOWN/src/egu00970.html]

egu00970


- egu:105041858

- Down regulated genes

c169070\_g1(-1.1156)

- egu:105055562

- Down regulated genes

c163858\_g1(-0.60352)

- egu:105039813

- Down regulated genes

c161964\_g1(-1.1747)

- egu:105054784

- Down regulated genes

c159463\_g1(-0.71686)
- egu:105050113

- Down regulated genes

c162019\_g1(-1.0066)

- egu:105047446

- Down regulated genes

c95934\_g1(-1.0804)

- egu:105045796

- Down regulated genes

c162191\_g1(-0.70144)

- egu:105052855

- Down regulated genes

c161205\_g1(-0.80345)

- egu:105056567

- Down regulated genes

c163317\_g1(-0.67501)
- egu:105055783

- Down regulated genes

c174800\_g1(-0.86302)
- egu:105034995

- Down regulated genes

c132118\_g1(-0.62889)

- egu:105056567

- Down regulated genes

c163317\_g1(-0.67501)
- egu:105055783

- Down regulated genes

c174800\_g1(-0.86302)
- egu:105034995

- Down regulated genes

c132118\_g1(-0.62889)

Close
