# Supplementary material for: Physiological and transcriptomic responses of Lanzhou Lily (Lilium davidii, var. unicolor) to cold stress
Source: PLoS One. 2020 Jan 23;15(1):e0227921. doi: 10.1371/journal.pone.0227921 (PMC6977731; doi:10.1371/journal.pone.0227921)
Supplement: S2 Zip — (Zip). CK: control (20°C); LT: low temperature (4°C). (ZIP) [file pone.0227921.s012.zip › S2 Zip/LTvsCK_DOWN/src/egu01110.html]

egu01110


- egu:105040768

- Down regulated genes

c168519\_g1(-1.1377)
- egu:105044798

- Down regulated genes

c165450\_g1(-1.0571)

- egu:105035555

- Down regulated genes

c157850\_g1(-0.78203)

- egu:105058545

- Down regulated genes

c166557\_g2(-1.5787) c166557\_g1(-1.7086)

- egu:105044579

- Down regulated genes

c155686\_g1(-0.65921)

- egu:105045201

- Down regulated genes

c146228\_g1(-0.90789)
- egu:105044229

- Down regulated genes

c164821\_g1(-2.5552)
- egu:105045835

- Down regulated genes

c171033\_g3(-0.62809)
- egu:105042390

- Down regulated genes

c173060\_g2(-1.2692)
- egu:105034542

- Down regulated genes

c174706\_g1(-0.9061)
- egu:105044265

- Down regulated genes

c173942\_g4(-2.7483)

- egu:105040139

- Down regulated genes

c104638\_g1(-0.74546)

- egu:105042381

- Down regulated genes

c169016\_g1(-0.80681)

- egu:105060549

- Down regulated genes

c166380\_g2(-0.48408)
- egu:105045732

- Down regulated genes

c164015\_g2(-1.0982)

- egu:105060549

- Down regulated genes

c166380\_g2(-0.48408)
- egu:105045732

- Down regulated genes

c164015\_g2(-1.0982)

- egu:105060549

- Down regulated genes

c166380\_g2(-0.48408)
- egu:105045732

- Down regulated genes

c164015\_g2(-1.0982)

- egu:105060549

- Down regulated genes

c166380\_g2(-0.48408)
- egu:105045732

- Down regulated genes

c164015\_g2(-1.0982)

- egu:105060549

- Down regulated genes

c166380\_g2(-0.48408)
- egu:105045732

- Down regulated genes

c164015\_g2(-1.0982)

- egu:105060549

- Down regulated genes

c166380\_g2(-0.48408)
- egu:105045732

- Down regulated genes

c164015\_g2(-1.0982)

- egu:105060549

- Down regulated genes

c166380\_g2(-0.48408)
- egu:105045732

- Down regulated genes

c164015\_g2(-1.0982)

- egu:105060549

- Down regulated genes

c166380\_g2(-0.48408)
- egu:105045732

- Down regulated genes

c164015\_g2(-1.0982)

- egu:105060549

- Down regulated genes

c166380\_g2(-0.48408)
- egu:105045732

- Down regulated genes

c164015\_g2(-1.0982)

- egu:105060549

- Down regulated genes

c166380\_g2(-0.48408)
- egu:105045732

- Down regulated genes

c164015\_g2(-1.0982)

- egu:105060549

- Down regulated genes

c166380\_g2(-0.48408)
- egu:105045732

- Down regulated genes

c164015\_g2(-1.0982)

- egu:105060549

- Down regulated genes

c166380\_g2(-0.48408)
- egu:105045732

- Down regulated genes

c164015\_g2(-1.0982)

- egu:105060549

- Down regulated genes

c166380\_g2(-0.48408)
- egu:105045732

- Down regulated genes

c164015\_g2(-1.0982)

- egu:105060549

- Down regulated genes

c166380\_g2(-0.48408)
- egu:105045732

- Down regulated genes

c164015\_g2(-1.0982)

- egu:105060549

- Down regulated genes

c166380\_g2(-0.48408)
- egu:105045732

- Down regulated genes

c164015\_g2(-1.0982)

- egu:105060549

- Down regulated genes

c166380\_g2(-0.48408)
- egu:105045732

- Down regulated genes

c164015\_g2(-1.0982)

- egu:105060549

- Down regulated genes

c166380\_g2(-0.48408)
- egu:105045732

- Down regulated genes

c164015\_g2(-1.0982)

- egu:105060549

- Down regulated genes

c166380\_g2(-0.48408)
- egu:105045732

- Down regulated genes

c164015\_g2(-1.0982)

- egu:105060549

- Down regulated genes

c166380\_g2(-0.48408)
- egu:105045732

- Down regulated genes

c164015\_g2(-1.0982)

- egu:105060549

- Down regulated genes

c166380\_g2(-0.48408)
- egu:105045732

- Down regulated genes

c164015\_g2(-1.0982)

- egu:105060549

- Down regulated genes

c166380\_g2(-0.48408)
- egu:105045732

- Down regulated genes

c164015\_g2(-1.0982)

- egu:105060549

- Down regulated genes

c166380\_g2(-0.48408)
- egu:105045732

- Down regulated genes

c164015\_g2(-1.0982)

- egu:105051526

- Down regulated genes

c154382\_g1(-0.80992)

- egu:105051526

- Down regulated genes

c154382\_g1(-0.80992)

- egu:105051526

- Down regulated genes

c154382\_g1(-0.80992)

- egu:105051526

- Down regulated genes

c154382\_g1(-0.80992)

- egu:105051526

- Down regulated genes

c154382\_g1(-0.80992)

- egu:105051526

- Down regulated genes

c154382\_g1(-0.80992)

- egu:105051526

- Down regulated genes

c154382\_g1(-0.80992)

- egu:105051526

- Down regulated genes

c154382\_g1(-0.80992)

- egu:105051526

- Down regulated genes

c154382\_g1(-0.80992)

- egu:105051526

- Down regulated genes

c154382\_g1(-0.80992)

- egu:105051526

- Down regulated genes

c154382\_g1(-0.80992)

- egu:105051526

- Down regulated genes

c154382\_g1(-0.80992)

- egu:105052174

- Down regulated genes

c157388\_g1(-1.0608)

- egu:105033050

- Down regulated genes

c104600\_g1(-0.82634)

- egu:105033050

- Down regulated genes

c104600\_g1(-0.82634)

- egu:105033050

- Down regulated genes

c104600\_g1(-0.82634)

- egu:105033050

- Down regulated genes

c104600\_g1(-0.82634)

- egu:105033050

- Down regulated genes

c104600\_g1(-0.82634)

- egu:105033050

- Down regulated genes

c104600\_g1(-0.82634)

- egu:105033050

- Down regulated genes

c104600\_g1(-0.82634)

- egu:105033050

- Down regulated genes

c104600\_g1(-0.82634)

- egu:105033050

- Down regulated genes

c104600\_g1(-0.82634)

- egu:105033050

- Down regulated genes

c104600\_g1(-0.82634)

- egu:105033050

- Down regulated genes

c104600\_g1(-0.82634)

- egu:105040461

- Down regulated genes

c164784\_g1(-0.84892)

- egu:105041662

- Down regulated genes

c168329\_g1(-0.67831)

- egu:105033309

- Down regulated genes

c164902\_g1(-2.7757)

- egu:105033675

- Down regulated genes

c158303\_g2(-0.80153)
- egu:105060163

- Down regulated genes

c224136\_g1(-0.74326)

- egu:105033675

- Down regulated genes

c158303\_g2(-0.80153)
- egu:105060163

- Down regulated genes

c224136\_g1(-0.74326)

- egu:105033675

- Down regulated genes

c158303\_g2(-0.80153)
- egu:105060163

- Down regulated genes

c224136\_g1(-0.74326)

- egu:105033675

- Down regulated genes

c158303\_g2(-0.80153)
- egu:105060163

- Down regulated genes

c224136\_g1(-0.74326)

- egu:105033675

- Down regulated genes

c158303\_g2(-0.80153)
- egu:105060163

- Down regulated genes

c224136\_g1(-0.74326)

- egu:105033675

- Down regulated genes

c158303\_g2(-0.80153)
- egu:105060163

- Down regulated genes

c224136\_g1(-0.74326)

- egu:105033675

- Down regulated genes

c158303\_g2(-0.80153)
- egu:105060163

- Down regulated genes

c224136\_g1(-0.74326)

- egu:105033675

- Down regulated genes

c158303\_g2(-0.80153)
- egu:105060163

- Down regulated genes

c224136\_g1(-0.74326)

- egu:105033675

- Down regulated genes

c158303\_g2(-0.80153)
- egu:105060163

- Down regulated genes

c224136\_g1(-0.74326)

- egu:105033675

- Down regulated genes

c158303\_g2(-0.80153)
- egu:105060163

- Down regulated genes

c224136\_g1(-0.74326)

- egu:105033675

- Down regulated genes

c158303\_g2(-0.80153)
- egu:105060163

- Down regulated genes

c224136\_g1(-0.74326)

- egu:105033675

- Down regulated genes

c158303\_g2(-0.80153)
- egu:105060163

- Down regulated genes

c224136\_g1(-0.74326)

- egu:105033675

- Down regulated genes

c158303\_g2(-0.80153)
- egu:105060163

- Down regulated genes

c224136\_g1(-0.74326)

- egu:105033675

- Down regulated genes

c158303\_g2(-0.80153)
- egu:105060163

- Down regulated genes

c224136\_g1(-0.74326)

- egu:105033675

- Down regulated genes

c158303\_g2(-0.80153)
- egu:105060163

- Down regulated genes

c224136\_g1(-0.74326)

- egu:105033675

- Down regulated genes

c158303\_g2(-0.80153)
- egu:105060163

- Down regulated genes

c224136\_g1(-0.74326)

- egu:105033675

- Down regulated genes

c158303\_g2(-0.80153)
- egu:105060163

- Down regulated genes

c224136\_g1(-0.74326)

- egu:105033675

- Down regulated genes

c158303\_g2(-0.80153)
- egu:105060163

- Down regulated genes

c224136\_g1(-0.74326)

- egu:105033675

- Down regulated genes

c158303\_g2(-0.80153)
- egu:105060163

- Down regulated genes

c224136\_g1(-0.74326)

- egu:105033675

- Down regulated genes

c158303\_g2(-0.80153)
- egu:105060163

- Down regulated genes

c224136\_g1(-0.74326)

- egu:105033675

- Down regulated genes

c158303\_g2(-0.80153)
- egu:105060163

- Down regulated genes

c224136\_g1(-0.74326)

- egu:105052307

- Down regulated genes

c12992\_g1(-1.8958)

- egu:105033675

- Down regulated genes

c158303\_g2(-0.80153)
- egu:105060163

- Down regulated genes

c224136\_g1(-0.74326)

- egu:105033675

- Down regulated genes

c158303\_g2(-0.80153)
- egu:105060163

- Down regulated genes

c224136\_g1(-0.74326)

- egu:105033675

- Down regulated genes

c158303\_g2(-0.80153)
- egu:105060163

- Down regulated genes

c224136\_g1(-0.74326)

- egu:105033675

- Down regulated genes

c158303\_g2(-0.80153)
- egu:105060163

- Down regulated genes

c224136\_g1(-0.74326)

- egu:105033675

- Down regulated genes

c158303\_g2(-0.80153)
- egu:105060163

- Down regulated genes

c224136\_g1(-0.74326)

- egu:105033675

- Down regulated genes

c158303\_g2(-0.80153)
- egu:105060163

- Down regulated genes

c224136\_g1(-0.74326)

- egu:105033675

- Down regulated genes

c158303\_g2(-0.80153)
- egu:105060163

- Down regulated genes

c224136\_g1(-0.74326)

- egu:105033675

- Down regulated genes

c158303\_g2(-0.80153)
- egu:105060163

- Down regulated genes

c224136\_g1(-0.74326)

- egu:105033675

- Down regulated genes

c158303\_g2(-0.80153)
- egu:105060163

- Down regulated genes

c224136\_g1(-0.74326)

- egu:105033675

- Down regulated genes

c158303\_g2(-0.80153)
- egu:105060163

- Down regulated genes

c224136\_g1(-0.74326)

- egu:105033675

- Down regulated genes

c158303\_g2(-0.80153)
- egu:105060163

- Down regulated genes

c224136\_g1(-0.74326)

- egu:105033675

- Down regulated genes

c158303\_g2(-0.80153)
- egu:105060163

- Down regulated genes

c224136\_g1(-0.74326)

- egu:105033675

- Down regulated genes

c158303\_g2(-0.80153)
- egu:105060163

- Down regulated genes

c224136\_g1(-0.74326)

- egu:105033675

- Down regulated genes

c158303\_g2(-0.80153)
- egu:105060163

- Down regulated genes

c224136\_g1(-0.74326)

- egu:105033675

- Down regulated genes

c158303\_g2(-0.80153)
- egu:105060163

- Down regulated genes

c224136\_g1(-0.74326)

- egu:105033675

- Down regulated genes

c158303\_g2(-0.80153)
- egu:105060163

- Down regulated genes

c224136\_g1(-0.74326)

- egu:105033675

- Down regulated genes

c158303\_g2(-0.80153)
- egu:105060163

- Down regulated genes

c224136\_g1(-0.74326)

- egu:105033675

- Down regulated genes

c158303\_g2(-0.80153)
- egu:105060163

- Down regulated genes

c224136\_g1(-0.74326)

- egu:105033675

- Down regulated genes

c158303\_g2(-0.80153)
- egu:105060163

- Down regulated genes

c224136\_g1(-0.74326)

- egu:105033675

- Down regulated genes

c158303\_g2(-0.80153)
- egu:105060163

- Down regulated genes

c224136\_g1(-0.74326)

- egu:105033675

- Down regulated genes

c158303\_g2(-0.80153)
- egu:105060163

- Down regulated genes

c224136\_g1(-0.74326)

- egu:105034750

- Down regulated genes

c160710\_g1(-0.80243) c174739\_g1(-2.4705)

- egu:105034750

- Down regulated genes

c160710\_g1(-0.80243) c174739\_g1(-2.4705)

- egu:105034750

- Down regulated genes

c160710\_g1(-0.80243) c174739\_g1(-2.4705)

- egu:105034750

- Down regulated genes

c160710\_g1(-0.80243) c174739\_g1(-2.4705)

- egu:105034750

- Down regulated genes

c160710\_g1(-0.80243) c174739\_g1(-2.4705)

- egu:105034750

- Down regulated genes

c160710\_g1(-0.80243) c174739\_g1(-2.4705)

- egu:105034750

- Down regulated genes

c160710\_g1(-0.80243) c174739\_g1(-2.4705)

- egu:105034750

- Down regulated genes

c160710\_g1(-0.80243) c174739\_g1(-2.4705)

- egu:105034750

- Down regulated genes

c160710\_g1(-0.80243) c174739\_g1(-2.4705)

- egu:105034750

- Down regulated genes

c160710\_g1(-0.80243) c174739\_g1(-2.4705)

- egu:105034750

- Down regulated genes

c160710\_g1(-0.80243) c174739\_g1(-2.4705)

- egu:105034750

- Down regulated genes

c160710\_g1(-0.80243) c174739\_g1(-2.4705)

- egu:105034750

- Down regulated genes

c160710\_g1(-0.80243) c174739\_g1(-2.4705)

- egu:105034750

- Down regulated genes

c160710\_g1(-0.80243) c174739\_g1(-2.4705)

- egu:105034750

- Down regulated genes

c160710\_g1(-0.80243) c174739\_g1(-2.4705)

- egu:105034750

- Down regulated genes

c160710\_g1(-0.80243) c174739\_g1(-2.4705)

- egu:105034750

- Down regulated genes

c160710\_g1(-0.80243) c174739\_g1(-2.4705)

- egu:105034750

- Down regulated genes

c160710\_g1(-0.80243) c174739\_g1(-2.4705)

- egu:105034750

- Down regulated genes

c160710\_g1(-0.80243) c174739\_g1(-2.4705)

- egu:105034750

- Down regulated genes

c160710\_g1(-0.80243) c174739\_g1(-2.4705)

- egu:105034750

- Down regulated genes

c160710\_g1(-0.80243) c174739\_g1(-2.4705)

- egu:105034750

- Down regulated genes

c160710\_g1(-0.80243) c174739\_g1(-2.4705)

- egu:105034750

- Down regulated genes

c160710\_g1(-0.80243) c174739\_g1(-2.4705)

- egu:105034750

- Down regulated genes

c160710\_g1(-0.80243) c174739\_g1(-2.4705)

- egu:105034750

- Down regulated genes

c160710\_g1(-0.80243) c174739\_g1(-2.4705)

- egu:105034750

- Down regulated genes

c160710\_g1(-0.80243) c174739\_g1(-2.4705)

- egu:105034750

- Down regulated genes

c160710\_g1(-0.80243) c174739\_g1(-2.4705)

- egu:105034750

- Down regulated genes

c160710\_g1(-0.80243) c174739\_g1(-2.4705)

- egu:105034750

- Down regulated genes

c160710\_g1(-0.80243) c174739\_g1(-2.4705)

- egu:105034750

- Down regulated genes

c160710\_g1(-0.80243) c174739\_g1(-2.4705)

- egu:105034750

- Down regulated genes

c160710\_g1(-0.80243) c174739\_g1(-2.4705)

- egu:105055883

- Down regulated genes

c152294\_g2(-1.0254)

- egu:105041933

- Down regulated genes

c165685\_g1(-0.59226)

- egu:105041933

- Down regulated genes

c165685\_g1(-0.59226)

- egu:105041933

- Down regulated genes

c165685\_g1(-0.59226)

- egu:105041933

- Down regulated genes

c165685\_g1(-0.59226)

- egu:105041933

- Down regulated genes

c165685\_g1(-0.59226)

- egu:105041933

- Down regulated genes

c165685\_g1(-0.59226)

- egu:105041933

- Down regulated genes

c165685\_g1(-0.59226)

- egu:105041933

- Down regulated genes

c165685\_g1(-0.59226)

- egu:105041933

- Down regulated genes

c165685\_g1(-0.59226)

- egu:105041933

- Down regulated genes

c165685\_g1(-0.59226)

- egu:105041933

- Down regulated genes

c165685\_g1(-0.59226)

- egu:105041933

- Down regulated genes

c165685\_g1(-0.59226)

- egu:105041933

- Down regulated genes

c165685\_g1(-0.59226)

- egu:105041933

- Down regulated genes

c165685\_g1(-0.59226)

- egu:105041933

- Down regulated genes

c165685\_g1(-0.59226)

- egu:105041933

- Down regulated genes

c165685\_g1(-0.59226)

- egu:105041933

- Down regulated genes

c165685\_g1(-0.59226)

- egu:105041933

- Down regulated genes

c165685\_g1(-0.59226)

- egu:105041933

- Down regulated genes

c165685\_g1(-0.59226)

- egu:105041933

- Down regulated genes

c165685\_g1(-0.59226)

- egu:105041933

- Down regulated genes

c165685\_g1(-0.59226)

- egu:105041933

- Down regulated genes

c165685\_g1(-0.59226)

- egu:105052168

- Down regulated genes

c170240\_g1(-0.60615)

- egu:105052168

- Down regulated genes

c170240\_g1(-0.60615)

- egu:105052168

- Down regulated genes

c170240\_g1(-0.60615)

- egu:105052168

- Down regulated genes

c170240\_g1(-0.60615)

- egu:105052168

- Down regulated genes

c170240\_g1(-0.60615)

- egu:105052168

- Down regulated genes

c170240\_g1(-0.60615)

- egu:105052168

- Down regulated genes

c170240\_g1(-0.60615)

- egu:105052168

- Down regulated genes

c170240\_g1(-0.60615)

- egu:105052168

- Down regulated genes

c170240\_g1(-0.60615)

- egu:105052168

- Down regulated genes

c170240\_g1(-0.60615)

- egu:105052168

- Down regulated genes

c170240\_g1(-0.60615)

- egu:105036836

- Down regulated genes

c159804\_g1(-0.77973)

- egu:105036836

- Down regulated genes

c159804\_g1(-0.77973)

- egu:105036836

- Down regulated genes

c159804\_g1(-0.77973)

- egu:105036836

- Down regulated genes

c159804\_g1(-0.77973)

- egu:105036836

- Down regulated genes

c159804\_g1(-0.77973)

- egu:105036836

- Down regulated genes

c159804\_g1(-0.77973)

- egu:105036836

- Down regulated genes

c159804\_g1(-0.77973)

- egu:105036836

- Down regulated genes

c159804\_g1(-0.77973)

- egu:105036836

- Down regulated genes

c159804\_g1(-0.77973)

- egu:105036836

- Down regulated genes

c159804\_g1(-0.77973)

- egu:105036836

- Down regulated genes

c159804\_g1(-0.77973)

- egu:105036836

- Down regulated genes

c159804\_g1(-0.77973)

- egu:105036836

- Down regulated genes

c159804\_g1(-0.77973)

- egu:105036836

- Down regulated genes

c159804\_g1(-0.77973)

- egu:105036836

- Down regulated genes

c159804\_g1(-0.77973)

- egu:105036836

- Down regulated genes

c159804\_g1(-0.77973)

- egu:105036836

- Down regulated genes

c159804\_g1(-0.77973)

- egu:105036836

- Down regulated genes

c159804\_g1(-0.77973)

- egu:105036836

- Down regulated genes

c159804\_g1(-0.77973)

- egu:105036836

- Down regulated genes

c159804\_g1(-0.77973)

- egu:105036836

- Down regulated genes

c159804\_g1(-0.77973)

- egu:105036836

- Down regulated genes

c159804\_g1(-0.77973)

- egu:105051428

- Down regulated genes

c153630\_g1(-1.15)

- egu:105049882

- Down regulated genes

c71483\_g1(-0.61029)

- egu:105049882

- Down regulated genes

c71483\_g1(-0.61029)

- egu:105049882

- Down regulated genes

c71483\_g1(-0.61029)

- egu:105049882

- Down regulated genes

c71483\_g1(-0.61029)

- egu:105049882

- Down regulated genes

c71483\_g1(-0.61029)

- egu:105049882

- Down regulated genes

c71483\_g1(-0.61029)

- egu:105049882

- Down regulated genes

c71483\_g1(-0.61029)

- egu:105049882

- Down regulated genes

c71483\_g1(-0.61029)

- egu:105049882

- Down regulated genes

c71483\_g1(-0.61029)

- egu:105049882

- Down regulated genes

c71483\_g1(-0.61029)

- egu:105049882

- Down regulated genes

c71483\_g1(-0.61029)

- egu:105049882

- Down regulated genes

c71483\_g1(-0.61029)

- egu:105049882

- Down regulated genes

c71483\_g1(-0.61029)

- egu:105049882

- Down regulated genes

c71483\_g1(-0.61029)

- egu:105049882

- Down regulated genes

c71483\_g1(-0.61029)

- egu:105049882

- Down regulated genes

c71483\_g1(-0.61029)

- egu:105049882

- Down regulated genes

c71483\_g1(-0.61029)

- egu:105049882

- Down regulated genes

c71483\_g1(-0.61029)

- egu:105049882

- Down regulated genes

c71483\_g1(-0.61029)

- egu:105049882

- Down regulated genes

c71483\_g1(-0.61029)

- egu:105049882

- Down regulated genes

c71483\_g1(-0.61029)

- egu:105049882

- Down regulated genes

c71483\_g1(-0.61029)

- egu:105053770

- Down regulated genes

c48670\_g1(-0.6809)
- egu:105047380

- Down regulated genes

c157902\_g1(-0.69904)

- egu:105039298

- Down regulated genes

c147908\_g1(-0.62718)

- egu:105035926

- Down regulated genes

c163701\_g1(-1.0664)
- egu:105059577

- Down regulated genes

c132497\_g1(-1.3633)

- egu:105059645

- Down regulated genes

c105237\_g1(-1.2193)

- egu:105059645

- Down regulated genes

c105237\_g1(-1.2193)

- egu:105059645

- Down regulated genes

c105237\_g1(-1.2193)

- egu:105059645

- Down regulated genes

c105237\_g1(-1.2193)

- egu:105059645

- Down regulated genes

c105237\_g1(-1.2193)

- egu:105059645

- Down regulated genes

c105237\_g1(-1.2193)

- egu:105059645

- Down regulated genes

c105237\_g1(-1.2193)

- egu:105059645

- Down regulated genes

c105237\_g1(-1.2193)

- egu:105059645

- Down regulated genes

c105237\_g1(-1.2193)

- egu:105059645

- Down regulated genes

c105237\_g1(-1.2193)

- egu:105059645

- Down regulated genes

c105237\_g1(-1.2193)

- egu:105056640

- Down regulated genes

c134111\_g1(-0.84087)

- egu:105057316

- Down regulated genes

c146595\_g1(-1.2756)

- egu:105034969

- Down regulated genes

c113371\_g2(-0.54621)

- egu:105043499

- Down regulated genes

c134153\_g1(-0.99629)

- egu:105041436

- Down regulated genes

c162392\_g1(-1.1453)
- egu:105034612

- Down regulated genes

c166374\_g1(-0.65476)

- egu:105034557

- Down regulated genes

c104889\_g1(-1.1155) c173703\_g2(-1.3642)
- egu:105054530

- Down regulated genes

c104889\_g2(-1.7233) c174574\_g3(-2.5042)

- egu:105034557

- Down regulated genes

c104889\_g1(-1.1155) c173703\_g2(-1.3642)
- egu:105054530

- Down regulated genes

c104889\_g2(-1.7233) c174574\_g3(-2.5042)

- egu:105034557

- Down regulated genes

c104889\_g1(-1.1155) c173703\_g2(-1.3642)
- egu:105054530

- Down regulated genes

c104889\_g2(-1.7233) c174574\_g3(-2.5042)

- egu:105034557

- Down regulated genes

c104889\_g1(-1.1155) c173703\_g2(-1.3642)
- egu:105054530

- Down regulated genes

c104889\_g2(-1.7233) c174574\_g3(-2.5042)

- egu:105034557

- Down regulated genes

c104889\_g1(-1.1155) c173703\_g2(-1.3642)
- egu:105054530

- Down regulated genes

c104889\_g2(-1.7233) c174574\_g3(-2.5042)

- egu:105034557

- Down regulated genes

c104889\_g1(-1.1155) c173703\_g2(-1.3642)
- egu:105054530

- Down regulated genes

c104889\_g2(-1.7233) c174574\_g3(-2.5042)

- egu:105034557

- Down regulated genes

c104889\_g1(-1.1155) c173703\_g2(-1.3642)
- egu:105054530

- Down regulated genes

c104889\_g2(-1.7233) c174574\_g3(-2.5042)

- egu:105034557

- Down regulated genes

c104889\_g1(-1.1155) c173703\_g2(-1.3642)
- egu:105054530

- Down regulated genes

c104889\_g2(-1.7233) c174574\_g3(-2.5042)

- egu:105034557

- Down regulated genes

c104889\_g1(-1.1155) c173703\_g2(-1.3642)
- egu:105054530

- Down regulated genes

c104889\_g2(-1.7233) c174574\_g3(-2.5042)

- egu:105034557

- Down regulated genes

c104889\_g1(-1.1155) c173703\_g2(-1.3642)
- egu:105054530

- Down regulated genes

c104889\_g2(-1.7233) c174574\_g3(-2.5042)

- egu:105034557

- Down regulated genes

c104889\_g1(-1.1155) c173703\_g2(-1.3642)
- egu:105054530

- Down regulated genes

c104889\_g2(-1.7233) c174574\_g3(-2.5042)

- egu:105056168

- Down regulated genes

c162034\_g1(-0.8811)
- egu:105056157

- Down regulated genes

c173582\_g1(-1.1307)

- egu:105050625

- Down regulated genes

c162112\_g2(-1.4107)
- egu:105048474

- Down regulated genes

c170804\_g2(-1.4555)
- egu:105051883

- Down regulated genes

c144640\_g1(-0.76162)

- egu:105050625

- Down regulated genes

c162112\_g2(-1.4107)
- egu:105048474

- Down regulated genes

c170804\_g2(-1.4555)
- egu:105051883

- Down regulated genes

c144640\_g1(-0.76162)

- egu:105050625

- Down regulated genes

c162112\_g2(-1.4107)
- egu:105048474

- Down regulated genes

c170804\_g2(-1.4555)
- egu:105051883

- Down regulated genes

c144640\_g1(-0.76162)

- egu:105050625

- Down regulated genes

c162112\_g2(-1.4107)
- egu:105048474

- Down regulated genes

c170804\_g2(-1.4555)
- egu:105051883

- Down regulated genes

c144640\_g1(-0.76162)

- egu:105050625

- Down regulated genes

c162112\_g2(-1.4107)
- egu:105048474

- Down regulated genes

c170804\_g2(-1.4555)
- egu:105051883

- Down regulated genes

c144640\_g1(-0.76162)

- egu:105050625

- Down regulated genes

c162112\_g2(-1.4107)
- egu:105048474

- Down regulated genes

c170804\_g2(-1.4555)
- egu:105051883

- Down regulated genes

c144640\_g1(-0.76162)

- egu:105050625

- Down regulated genes

c162112\_g2(-1.4107)
- egu:105048474

- Down regulated genes

c170804\_g2(-1.4555)
- egu:105051883

- Down regulated genes

c144640\_g1(-0.76162)

- egu:105050625

- Down regulated genes

c162112\_g2(-1.4107)
- egu:105048474

- Down regulated genes

c170804\_g2(-1.4555)
- egu:105051883

- Down regulated genes

c144640\_g1(-0.76162)

- egu:105050625

- Down regulated genes

c162112\_g2(-1.4107)
- egu:105048474

- Down regulated genes

c170804\_g2(-1.4555)
- egu:105051883

- Down regulated genes

c144640\_g1(-0.76162)

- egu:105050625

- Down regulated genes

c162112\_g2(-1.4107)
- egu:105048474

- Down regulated genes

c170804\_g2(-1.4555)
- egu:105051883

- Down regulated genes

c144640\_g1(-0.76162)

- egu:105050625

- Down regulated genes

c162112\_g2(-1.4107)
- egu:105048474

- Down regulated genes

c170804\_g2(-1.4555)
- egu:105051883

- Down regulated genes

c144640\_g1(-0.76162)

- egu:105050625

- Down regulated genes

c162112\_g2(-1.4107)
- egu:105048474

- Down regulated genes

c170804\_g2(-1.4555)
- egu:105051883

- Down regulated genes

c144640\_g1(-0.76162)

- egu:105050625

- Down regulated genes

c162112\_g2(-1.4107)
- egu:105048474

- Down regulated genes

c170804\_g2(-1.4555)
- egu:105051883

- Down regulated genes

c144640\_g1(-0.76162)

- egu:105050625

- Down regulated genes

c162112\_g2(-1.4107)
- egu:105048474

- Down regulated genes

c170804\_g2(-1.4555)
- egu:105051883

- Down regulated genes

c144640\_g1(-0.76162)

- egu:105050625

- Down regulated genes

c162112\_g2(-1.4107)
- egu:105048474

- Down regulated genes

c170804\_g2(-1.4555)
- egu:105051883

- Down regulated genes

c144640\_g1(-0.76162)

- egu:105050625

- Down regulated genes

c162112\_g2(-1.4107)
- egu:105048474

- Down regulated genes

c170804\_g2(-1.4555)
- egu:105051883

- Down regulated genes

c144640\_g1(-0.76162)

- egu:105050625

- Down regulated genes

c162112\_g2(-1.4107)
- egu:105048474

- Down regulated genes

c170804\_g2(-1.4555)
- egu:105051883

- Down regulated genes

c144640\_g1(-0.76162)

- egu:105050625

- Down regulated genes

c162112\_g2(-1.4107)
- egu:105048474

- Down regulated genes

c170804\_g2(-1.4555)
- egu:105051883

- Down regulated genes

c144640\_g1(-0.76162)

- egu:105050625

- Down regulated genes

c162112\_g2(-1.4107)
- egu:105048474

- Down regulated genes

c170804\_g2(-1.4555)
- egu:105051883

- Down regulated genes

c144640\_g1(-0.76162)

- egu:105050625

- Down regulated genes

c162112\_g2(-1.4107)
- egu:105048474

- Down regulated genes

c170804\_g2(-1.4555)
- egu:105051883

- Down regulated genes

c144640\_g1(-0.76162)

- egu:105050625

- Down regulated genes

c162112\_g2(-1.4107)
- egu:105048474

- Down regulated genes

c170804\_g2(-1.4555)
- egu:105051883

- Down regulated genes

c144640\_g1(-0.76162)

- egu:105050625

- Down regulated genes

c162112\_g2(-1.4107)
- egu:105048474

- Down regulated genes

c170804\_g2(-1.4555)
- egu:105051883

- Down regulated genes

c144640\_g1(-0.76162)

- egu:105038009

- Down regulated genes

c170857\_g1(-0.78343)
- egu:105035321

- Down regulated genes

c154502\_g4(-0.97348)
- egu:105049380

- Down regulated genes

c85645\_g1(-1.342)

- egu:105060694

- Down regulated genes

c133070\_g1(-0.6949)

- egu:105050388

- Down regulated genes

c132652\_g1(-0.67228)
- egu:105060694

- Down regulated genes

c133070\_g1(-0.6949)

- egu:105048825

- Down regulated genes

c155247\_g1(-0.65624)

- egu:105060694

- Down regulated genes

c133070\_g1(-0.6949)

- egu:105060694

- Down regulated genes

c133070\_g1(-0.6949)

- egu:105060694

- Down regulated genes

c133070\_g1(-0.6949)

- egu:105060694

- Down regulated genes

c133070\_g1(-0.6949)

- egu:105060694

- Down regulated genes

c133070\_g1(-0.6949)

- egu:105060694

- Down regulated genes

c133070\_g1(-0.6949)

- egu:105060694

- Down regulated genes

c133070\_g1(-0.6949)

- egu:105060694

- Down regulated genes

c133070\_g1(-0.6949)

- egu:105060694

- Down regulated genes

c133070\_g1(-0.6949)

- egu:105060694

- Down regulated genes

c133070\_g1(-0.6949)

- egu:105060694

- Down regulated genes

c133070\_g1(-0.6949)

- egu:105057517

- Down regulated genes

c147541\_g1(-1.0111)
- egu:105032039

- Down regulated genes

c154303\_g1(-1.424)

- egu:105040940

- Down regulated genes

c185151\_g1(-2.1044)
- egu:105048107

- Down regulated genes

c159323\_g1(-1.3206)

- egu:105040940

- Down regulated genes

c185151\_g1(-2.1044)
- egu:105048107

- Down regulated genes

c159323\_g1(-1.3206)

- egu:105033284

- Down regulated genes

c141783\_g1(-1.1992)

- egu:105037657

- Down regulated genes

c165472\_g1(-1.2074)

- egu:105044629

- Down regulated genes

c156209\_g1(-1.6383)
- egu:105042952

- Down regulated genes

c101133\_g1(-6.3517)
- egu:105043191

- Down regulated genes

c147467\_g1(-2.0691)

- egu:105045201

- Down regulated genes

c146228\_g1(-0.90789)
- egu:105044229

- Down regulated genes

c164821\_g1(-2.5552)
- egu:105045835

- Down regulated genes

c171033\_g3(-0.62809)
- egu:105042390

- Down regulated genes

c173060\_g2(-1.2692)
- egu:105034542

- Down regulated genes

c174706\_g1(-0.9061)
- egu:105044265

- Down regulated genes

c173942\_g4(-2.7483)

- egu:105037657

- Down regulated genes

c165472\_g1(-1.2074)

- egu:105045448

- Down regulated genes

c171016\_g1(-1.8154)
- egu:105053765

- Down regulated genes

c168470\_g1(-3.8645)

- egu:105045448

- Down regulated genes

c171016\_g1(-1.8154)
- egu:105053765

- Down regulated genes

c168470\_g1(-3.8645)

- egu:105045448

- Down regulated genes

c171016\_g1(-1.8154)
- egu:105053765

- Down regulated genes

c168470\_g1(-3.8645)

- egu:105045448

- Down regulated genes

c171016\_g1(-1.8154)
- egu:105053765

- Down regulated genes

c168470\_g1(-3.8645)

- egu:105045448

- Down regulated genes

c171016\_g1(-1.8154)
- egu:105053765

- Down regulated genes

c168470\_g1(-3.8645)

- egu:105045448

- Down regulated genes

c171016\_g1(-1.8154)
- egu:105053765

- Down regulated genes

c168470\_g1(-3.8645)

- egu:105045448

- Down regulated genes

c171016\_g1(-1.8154)
- egu:105053765

- Down regulated genes

c168470\_g1(-3.8645)

- egu:105045448

- Down regulated genes

c171016\_g1(-1.8154)
- egu:105053765

- Down regulated genes

c168470\_g1(-3.8645)

- egu:105045448

- Down regulated genes

c171016\_g1(-1.8154)
- egu:105053765

- Down regulated genes

c168470\_g1(-3.8645)

- egu:105045448

- Down regulated genes

c171016\_g1(-1.8154)
- egu:105053765

- Down regulated genes

c168470\_g1(-3.8645)

- egu:105045448

- Down regulated genes

c171016\_g1(-1.8154)
- egu:105053765

- Down regulated genes

c168470\_g1(-3.8645)

- egu:105045448

- Down regulated genes

c171016\_g1(-1.8154)
- egu:105053765

- Down regulated genes

c168470\_g1(-3.8645)

- egu:105045448

- Down regulated genes

c171016\_g1(-1.8154)
- egu:105053765

- Down regulated genes

c168470\_g1(-3.8645)

- egu:105045448

- Down regulated genes

c171016\_g1(-1.8154)
- egu:105053765

- Down regulated genes

c168470\_g1(-3.8645)

- egu:105045448

- Down regulated genes

c171016\_g1(-1.8154)
- egu:105053765

- Down regulated genes

c168470\_g1(-3.8645)

- egu:105045448

- Down regulated genes

c171016\_g1(-1.8154)
- egu:105053765

- Down regulated genes

c168470\_g1(-3.8645)

- egu:105045448

- Down regulated genes

c171016\_g1(-1.8154)
- egu:105053765

- Down regulated genes

c168470\_g1(-3.8645)

- egu:105045448

- Down regulated genes

c171016\_g1(-1.8154)
- egu:105053765

- Down regulated genes

c168470\_g1(-3.8645)

- egu:105045448

- Down regulated genes

c171016\_g1(-1.8154)
- egu:105053765

- Down regulated genes

c168470\_g1(-3.8645)

- egu:105045448

- Down regulated genes

c171016\_g1(-1.8154)
- egu:105053765

- Down regulated genes

c168470\_g1(-3.8645)

- egu:105045448

- Down regulated genes

c171016\_g1(-1.8154)
- egu:105053765

- Down regulated genes

c168470\_g1(-3.8645)

- egu:105045448

- Down regulated genes

c171016\_g1(-1.8154)
- egu:105053765

- Down regulated genes

c168470\_g1(-3.8645)

- egu:105045448

- Down regulated genes

c171016\_g1(-1.8154)
- egu:105053765

- Down regulated genes

c168470\_g1(-3.8645)

- egu:105045448

- Down regulated genes

c171016\_g1(-1.8154)
- egu:105053765

- Down regulated genes

c168470\_g1(-3.8645)

- egu:105045448

- Down regulated genes

c171016\_g1(-1.8154)
- egu:105053765

- Down regulated genes

c168470\_g1(-3.8645)

- egu:105045448

- Down regulated genes

c171016\_g1(-1.8154)
- egu:105053765

- Down regulated genes

c168470\_g1(-3.8645)

- egu:105045448

- Down regulated genes

c171016\_g1(-1.8154)
- egu:105053765

- Down regulated genes

c168470\_g1(-3.8645)

- egu:105045448

- Down regulated genes

c171016\_g1(-1.8154)
- egu:105053765

- Down regulated genes

c168470\_g1(-3.8645)

- egu:105045448

- Down regulated genes

c171016\_g1(-1.8154)
- egu:105053765

- Down regulated genes

c168470\_g1(-3.8645)

- egu:105045448

- Down regulated genes

c171016\_g1(-1.8154)
- egu:105053765

- Down regulated genes

c168470\_g1(-3.8645)

- egu:105045448

- Down regulated genes

c171016\_g1(-1.8154)
- egu:105053765

- Down regulated genes

c168470\_g1(-3.8645)

- egu:105045448

- Down regulated genes

c171016\_g1(-1.8154)
- egu:105053765

- Down regulated genes

c168470\_g1(-3.8645)

- egu:105045448

- Down regulated genes

c171016\_g1(-1.8154)
- egu:105053765

- Down regulated genes

c168470\_g1(-3.8645)

- egu:105045448

- Down regulated genes

c171016\_g1(-1.8154)
- egu:105053765

- Down regulated genes

c168470\_g1(-3.8645)

- egu:105045448

- Down regulated genes

c171016\_g1(-1.8154)
- egu:105053765

- Down regulated genes

c168470\_g1(-3.8645)

- egu:105045448

- Down regulated genes

c171016\_g1(-1.8154)
- egu:105053765

- Down regulated genes

c168470\_g1(-3.8645)

- egu:105045448

- Down regulated genes

c171016\_g1(-1.8154)
- egu:105053765

- Down regulated genes

c168470\_g1(-3.8645)

- egu:105045448

- Down regulated genes

c171016\_g1(-1.8154)
- egu:105053765

- Down regulated genes

c168470\_g1(-3.8645)

- egu:105045448

- Down regulated genes

c171016\_g1(-1.8154)
- egu:105053765

- Down regulated genes

c168470\_g1(-3.8645)

- egu:105045448

- Down regulated genes

c171016\_g1(-1.8154)
- egu:105053765

- Down regulated genes

c168470\_g1(-3.8645)

- egu:105045448

- Down regulated genes

c171016\_g1(-1.8154)
- egu:105053765

- Down regulated genes

c168470\_g1(-3.8645)

- egu:105045448

- Down regulated genes

c171016\_g1(-1.8154)
- egu:105053765

- Down regulated genes

c168470\_g1(-3.8645)

- egu:105045448

- Down regulated genes

c171016\_g1(-1.8154)
- egu:105053765

- Down regulated genes

c168470\_g1(-3.8645)

- egu:105045448

- Down regulated genes

c171016\_g1(-1.8154)
- egu:105053765

- Down regulated genes

c168470\_g1(-3.8645)

- egu:105054950

- Down regulated genes

c151227\_g1(-Inf)

- egu:105037657

- Down regulated genes

c165472\_g1(-1.2074)

- egu:105054950

- Down regulated genes

c151227\_g1(-Inf)

- egu:105054950

- Down regulated genes

c151227\_g1(-Inf)

- egu:105044629

- Down regulated genes

c156209\_g1(-1.6383)
- egu:105042952

- Down regulated genes

c101133\_g1(-6.3517)
- egu:105043191

- Down regulated genes

c147467\_g1(-2.0691)

- egu:105037657

- Down regulated genes

c165472\_g1(-1.2074)

- egu:105054950

- Down regulated genes

c151227\_g1(-Inf)

- egu:105037657

- Down regulated genes

c165472\_g1(-1.2074)

- egu:105054950

- Down regulated genes

c151227\_g1(-Inf)

- egu:105054950

- Down regulated genes

c151227\_g1(-Inf)

- egu:105044629

- Down regulated genes

c156209\_g1(-1.6383)
- egu:105042952

- Down regulated genes

c101133\_g1(-6.3517)
- egu:105043191

- Down regulated genes

c147467\_g1(-2.0691)

- egu:105044125

- Down regulated genes

c135610\_g1(-0.95193)

- egu:105044125

- Down regulated genes

c135610\_g1(-0.95193)

- egu:105044125

- Down regulated genes

c135610\_g1(-0.95193)

- egu:105044125

- Down regulated genes

c135610\_g1(-0.95193)

- egu:105044125

- Down regulated genes

c135610\_g1(-0.95193)

- egu:105044125

- Down regulated genes

c135610\_g1(-0.95193)

- egu:105044125

- Down regulated genes

c135610\_g1(-0.95193)

- egu:105044125

- Down regulated genes

c135610\_g1(-0.95193)

- egu:105044125

- Down regulated genes

c135610\_g1(-0.95193)

- egu:105044125

- Down regulated genes

c135610\_g1(-0.95193)

- egu:105044125

- Down regulated genes

c135610\_g1(-0.95193)

- egu:105048107

- Down regulated genes

c159323\_g1(-1.3206)

- egu:105048107

- Down regulated genes

c159323\_g1(-1.3206)

- egu:105048107

- Down regulated genes

c159323\_g1(-1.3206)

- egu:105048107

- Down regulated genes

c159323\_g1(-1.3206)

- egu:105048107

- Down regulated genes

c159323\_g1(-1.3206)

- egu:105048107

- Down regulated genes

c159323\_g1(-1.3206)

- egu:105048107

- Down regulated genes

c159323\_g1(-1.3206)

- egu:105048107

- Down regulated genes

c159323\_g1(-1.3206)

- egu:105048107

- Down regulated genes

c159323\_g1(-1.3206)

- egu:105048107

- Down regulated genes

c159323\_g1(-1.3206)

- egu:105048107

- Down regulated genes

c159323\_g1(-1.3206)

- egu:105043264

- Down regulated genes

c152607\_g1(-0.78732)

- egu:105043264

- Down regulated genes

c152607\_g1(-0.78732)

- egu:105043264

- Down regulated genes

c152607\_g1(-0.78732)

- egu:105043264

- Down regulated genes

c152607\_g1(-0.78732)

- egu:105043264

- Down regulated genes

c152607\_g1(-0.78732)

- egu:105043264

- Down regulated genes

c152607\_g1(-0.78732)

- egu:105043264

- Down regulated genes

c152607\_g1(-0.78732)

- egu:105043264

- Down regulated genes

c152607\_g1(-0.78732)

- egu:105043264

- Down regulated genes

c152607\_g1(-0.78732)

- egu:105043264

- Down regulated genes

c152607\_g1(-0.78732)

- egu:105043264

- Down regulated genes

c152607\_g1(-0.78732)

- egu:105043264

- Down regulated genes

c152607\_g1(-0.78732)

- egu:105043264

- Down regulated genes

c152607\_g1(-0.78732)

- egu:105043264

- Down regulated genes

c152607\_g1(-0.78732)

- egu:105043264

- Down regulated genes

c152607\_g1(-0.78732)

- egu:105043264

- Down regulated genes

c152607\_g1(-0.78732)

- egu:105043264

- Down regulated genes

c152607\_g1(-0.78732)

- egu:105043264

- Down regulated genes

c152607\_g1(-0.78732)

- egu:105043264

- Down regulated genes

c152607\_g1(-0.78732)

- egu:105043264

- Down regulated genes

c152607\_g1(-0.78732)

- egu:105043264

- Down regulated genes

c152607\_g1(-0.78732)

- egu:105055151

- Down regulated genes

c124333\_g1(-3.6104)

- egu:105044125

- Down regulated genes

c135610\_g1(-0.95193)

- egu:105044348

- Down regulated genes

c171087\_g1(-1.2705)

- egu:105049020

- Down regulated genes

c168243\_g1(-0.66676)

- egu:105061169

- Down regulated genes

c113031\_g1(-1.0116)

- egu:105044125

- Down regulated genes

c135610\_g1(-0.95193)

- egu:105037896

- Down regulated genes

c162518\_g1(-0.68546)

- egu:105034341

- Down regulated genes

c131571\_g1(-0.92365)

- egu:105052855

- Down regulated genes

c161205\_g1(-0.80345)

- egu:105054529

- Down regulated genes

c167947\_g1(-2.8328) c170780\_g1(-0.95455)

- egu:105051026

- Down regulated genes

c167743\_g1(-0.91681)

- egu:105035064

- Down regulated genes

c152469\_g1(-0.94741)

- egu:105035064

- Down regulated genes

c152469\_g1(-0.94741)

- egu:105060927

- Down regulated genes

c173971\_g3(-1.266)

- egu:105047162

- Down regulated genes

c154629\_g1(-1.3269)

- egu:105041933

- Down regulated genes

c165685\_g1(-0.59226)

- egu:105041933

- Down regulated genes

c165685\_g1(-0.59226)

- egu:105041933

- Down regulated genes

c165685\_g1(-0.59226)

- egu:105041933

- Down regulated genes

c165685\_g1(-0.59226)

- egu:105041933

- Down regulated genes

c165685\_g1(-0.59226)

- egu:105041933

- Down regulated genes

c165685\_g1(-0.59226)

- egu:105041933

- Down regulated genes

c165685\_g1(-0.59226)

- egu:105041933

- Down regulated genes

c165685\_g1(-0.59226)

- egu:105041933

- Down regulated genes

c165685\_g1(-0.59226)

- egu:105041933

- Down regulated genes

c165685\_g1(-0.59226)

- egu:105041933

- Down regulated genes

c165685\_g1(-0.59226)

- egu:105041933

- Down regulated genes

c165685\_g1(-0.59226)

- egu:105035064

- Down regulated genes

c152469\_g1(-0.94741)

- egu:105035064

- Down regulated genes

c152469\_g1(-0.94741)

- egu:105037930

- Down regulated genes

c71670\_g1(-0.58079)

- egu:105037930

- Down regulated genes

c71670\_g1(-0.58079)

- egu:105037930

- Down regulated genes

c71670\_g1(-0.58079)

- egu:105037930

- Down regulated genes

c71670\_g1(-0.58079)

- egu:105037930

- Down regulated genes

c71670\_g1(-0.58079)

- egu:105037930

- Down regulated genes

c71670\_g1(-0.58079)

- egu:105037930

- Down regulated genes

c71670\_g1(-0.58079)

- egu:105037930

- Down regulated genes

c71670\_g1(-0.58079)

- egu:105037930

- Down regulated genes

c71670\_g1(-0.58079)

- egu:105037930

- Down regulated genes

c71670\_g1(-0.58079)

- egu:105037930

- Down regulated genes

c71670\_g1(-0.58079)

- egu:105037930

- Down regulated genes

c71670\_g1(-0.58079)

- egu:105037930

- Down regulated genes

c71670\_g1(-0.58079)

- egu:105037930

- Down regulated genes

c71670\_g1(-0.58079)

- egu:105037930

- Down regulated genes

c71670\_g1(-0.58079)

- egu:105037930

- Down regulated genes

c71670\_g1(-0.58079)

- egu:105037930

- Down regulated genes

c71670\_g1(-0.58079)

- egu:105037930

- Down regulated genes

c71670\_g1(-0.58079)

- egu:105037930

- Down regulated genes

c71670\_g1(-0.58079)

- egu:105037930

- Down regulated genes

c71670\_g1(-0.58079)

- egu:105037930

- Down regulated genes

c71670\_g1(-0.58079)

- egu:105037930

- Down regulated genes

c71670\_g1(-0.58079)

- egu:105037930

- Down regulated genes

c71670\_g1(-0.58079)

- egu:105037930

- Down regulated genes

c71670\_g1(-0.58079)

- egu:105037930

- Down regulated genes

c71670\_g1(-0.58079)

- egu:105037930

- Down regulated genes

c71670\_g1(-0.58079)

- egu:105037930

- Down regulated genes

c71670\_g1(-0.58079)

- egu:105037930

- Down regulated genes

c71670\_g1(-0.58079)

- egu:105037930

- Down regulated genes

c71670\_g1(-0.58079)

- egu:105037930

- Down regulated genes

c71670\_g1(-0.58079)

- egu:105037930

- Down regulated genes

c71670\_g1(-0.58079)

- egu:105037930

- Down regulated genes

c71670\_g1(-0.58079)

- egu:105037930

- Down regulated genes

c71670\_g1(-0.58079)

- egu:105037930

- Down regulated genes

c71670\_g1(-0.58079)

- egu:105037930

- Down regulated genes

c71670\_g1(-0.58079)

- egu:105037930

- Down regulated genes

c71670\_g1(-0.58079)

- egu:105037930

- Down regulated genes

c71670\_g1(-0.58079)

- egu:105037930

- Down regulated genes

c71670\_g1(-0.58079)

- egu:105037930

- Down regulated genes

c71670\_g1(-0.58079)

- egu:105037930

- Down regulated genes

c71670\_g1(-0.58079)

- egu:105037930

- Down regulated genes

c71670\_g1(-0.58079)

- egu:105037930

- Down regulated genes

c71670\_g1(-0.58079)

- egu:105049221

- Down regulated genes

c71809\_g1(-1.2195)

- egu:105040768

- Down regulated genes

c168519\_g1(-1.1377)
- egu:105044798

- Down regulated genes

c165450\_g1(-1.0571)

- egu:105059913

- Down regulated genes

c152307\_g1(-0.77696)

- egu:105035555

- Down regulated genes

c157850\_g1(-0.78203)

- egu:105057582

- Down regulated genes

c169028\_g1(-0.70656)
- egu:105040656

- Down regulated genes

c133188\_g1(-0.87542)

- egu:105035938

- Down regulated genes

c123480\_g1(-1.3599)

- egu:105036097

- Down regulated genes

c185147\_g2(-1.0982)

- egu:105036097

- Down regulated genes

c185147\_g2(-1.0982)

- egu:105036097

- Down regulated genes

c185147\_g2(-1.0982)

- egu:105044579

- Down regulated genes

c155686\_g1(-0.65921)

- egu:105035618

- Down regulated genes

c134612\_g1(-0.62277)

- egu:105044579

- Down regulated genes

c155686\_g1(-0.65921)

- egu:105035618

- Down regulated genes

c134612\_g1(-0.62277)

- egu:105035618

- Down regulated genes

c134612\_g1(-0.62277)

- egu:105035618

- Down regulated genes

c134612\_g1(-0.62277)

- egu:105035618

- Down regulated genes

c134612\_g1(-0.62277)

- egu:105035618

- Down regulated genes

c134612\_g1(-0.62277)

- egu:105035618

- Down regulated genes

c134612\_g1(-0.62277)

- egu:105035618

- Down regulated genes

c134612\_g1(-0.62277)

- egu:105035618

- Down regulated genes

c134612\_g1(-0.62277)

- egu:105035618

- Down regulated genes

c134612\_g1(-0.62277)

- egu:105035618

- Down regulated genes

c134612\_g1(-0.62277)

- egu:105035618

- Down regulated genes

c134612\_g1(-0.62277)

- egu:105035618

- Down regulated genes

c134612\_g1(-0.62277)

- egu:105035618

- Down regulated genes

c134612\_g1(-0.62277)

- egu:105035618

- Down regulated genes

c134612\_g1(-0.62277)

- egu:105035618

- Down regulated genes

c134612\_g1(-0.62277)

- egu:105035618

- Down regulated genes

c134612\_g1(-0.62277)

- egu:105035618

- Down regulated genes

c134612\_g1(-0.62277)

- egu:105035618

- Down regulated genes

c134612\_g1(-0.62277)

- egu:105035618

- Down regulated genes

c134612\_g1(-0.62277)

- egu:105035618

- Down regulated genes

c134612\_g1(-0.62277)

- egu:105035618

- Down regulated genes

c134612\_g1(-0.62277)

- egu:105044935

- Down regulated genes

c165968\_g2(-0.60953)

- egu:105055679

- Down regulated genes

c169641\_g1(-1.9236)

- egu:105055679

- Down regulated genes

c169641\_g1(-1.9236)

- egu:105055679

- Down regulated genes

c169641\_g1(-1.9236)

- egu:105055679

- Down regulated genes

c169641\_g1(-1.9236)

- egu:105055679

- Down regulated genes

c169641\_g1(-1.9236)

- egu:105055679

- Down regulated genes

c169641\_g1(-1.9236)

- egu:105055679

- Down regulated genes

c169641\_g1(-1.9236)

- egu:105055679

- Down regulated genes

c169641\_g1(-1.9236)

- egu:105055679

- Down regulated genes

c169641\_g1(-1.9236)

- egu:105055679

- Down regulated genes

c169641\_g1(-1.9236)

- egu:105055679

- Down regulated genes

c169641\_g1(-1.9236)

- egu:105059611

- Down regulated genes

c198353\_g1(-0.79652)

- egu:105049274

- Down regulated genes

c155055\_g1(-0.67731)
- egu:105055979

- Down regulated genes

c154844\_g1(-1.0304)
- egu:105054827

- Down regulated genes

c153259\_g1(-0.59995)

- egu:105049274

- Down regulated genes

c155055\_g1(-0.67731)
- egu:105055979

- Down regulated genes

c154844\_g1(-1.0304)
- egu:105054827

- Down regulated genes

c153259\_g1(-0.59995)

- egu:105049274

- Down regulated genes

c155055\_g1(-0.67731)
- egu:105055979

- Down regulated genes

c154844\_g1(-1.0304)
- egu:105054827

- Down regulated genes

c153259\_g1(-0.59995)

- egu:105049274

- Down regulated genes

c155055\_g1(-0.67731)
- egu:105055979

- Down regulated genes

c154844\_g1(-1.0304)
- egu:105054827

- Down regulated genes

c153259\_g1(-0.59995)

- egu:105049274

- Down regulated genes

c155055\_g1(-0.67731)
- egu:105055979

- Down regulated genes

c154844\_g1(-1.0304)
- egu:105054827

- Down regulated genes

c153259\_g1(-0.59995)

- egu:105049274

- Down regulated genes

c155055\_g1(-0.67731)
- egu:105055979

- Down regulated genes

c154844\_g1(-1.0304)
- egu:105054827

- Down regulated genes

c153259\_g1(-0.59995)

- egu:105049274

- Down regulated genes

c155055\_g1(-0.67731)
- egu:105055979

- Down regulated genes

c154844\_g1(-1.0304)
- egu:105054827

- Down regulated genes

c153259\_g1(-0.59995)

- egu:105049274

- Down regulated genes

c155055\_g1(-0.67731)
- egu:105055979

- Down regulated genes

c154844\_g1(-1.0304)
- egu:105054827

- Down regulated genes

c153259\_g1(-0.59995)

- egu:105049274

- Down regulated genes

c155055\_g1(-0.67731)
- egu:105055979

- Down regulated genes

c154844\_g1(-1.0304)
- egu:105054827

- Down regulated genes

c153259\_g1(-0.59995)

- egu:105049274

- Down regulated genes

c155055\_g1(-0.67731)
- egu:105055979

- Down regulated genes

c154844\_g1(-1.0304)
- egu:105054827

- Down regulated genes

c153259\_g1(-0.59995)

- egu:105049274

- Down regulated genes

c155055\_g1(-0.67731)
- egu:105055979

- Down regulated genes

c154844\_g1(-1.0304)
- egu:105054827

- Down regulated genes

c153259\_g1(-0.59995)

- egu:105049274

- Down regulated genes

c155055\_g1(-0.67731)
- egu:105055979

- Down regulated genes

c154844\_g1(-1.0304)
- egu:105054827

- Down regulated genes

c153259\_g1(-0.59995)

- egu:105049274

- Down regulated genes

c155055\_g1(-0.67731)
- egu:105055979

- Down regulated genes

c154844\_g1(-1.0304)
- egu:105054827

- Down regulated genes

c153259\_g1(-0.59995)

- egu:105049274

- Down regulated genes

c155055\_g1(-0.67731)
- egu:105055979

- Down regulated genes

c154844\_g1(-1.0304)
- egu:105054827

- Down regulated genes

c153259\_g1(-0.59995)

- egu:105049274

- Down regulated genes

c155055\_g1(-0.67731)
- egu:105055979

- Down regulated genes

c154844\_g1(-1.0304)
- egu:105054827

- Down regulated genes

c153259\_g1(-0.59995)

- egu:105049274

- Down regulated genes

c155055\_g1(-0.67731)
- egu:105055979

- Down regulated genes

c154844\_g1(-1.0304)
- egu:105054827

- Down regulated genes

c153259\_g1(-0.59995)

- egu:105049274

- Down regulated genes

c155055\_g1(-0.67731)
- egu:105055979

- Down regulated genes

c154844\_g1(-1.0304)
- egu:105054827

- Down regulated genes

c153259\_g1(-0.59995)

- egu:105049274

- Down regulated genes

c155055\_g1(-0.67731)
- egu:105055979

- Down regulated genes

c154844\_g1(-1.0304)
- egu:105054827

- Down regulated genes

c153259\_g1(-0.59995)

- egu:105049274

- Down regulated genes

c155055\_g1(-0.67731)
- egu:105055979

- Down regulated genes

c154844\_g1(-1.0304)
- egu:105054827

- Down regulated genes

c153259\_g1(-0.59995)

- egu:105049274

- Down regulated genes

c155055\_g1(-0.67731)
- egu:105055979

- Down regulated genes

c154844\_g1(-1.0304)
- egu:105054827

- Down regulated genes

c153259\_g1(-0.59995)

- egu:105049274

- Down regulated genes

c155055\_g1(-0.67731)
- egu:105055979

- Down regulated genes

c154844\_g1(-1.0304)
- egu:105054827

- Down regulated genes

c153259\_g1(-0.59995)

- egu:105055609

- Down regulated genes

c152224\_g1(-0.46463)

- egu:105040851

- Down regulated genes

c156623\_g1(-0.57504)

- egu:105040851

- Down regulated genes

c156623\_g1(-0.57504)

- egu:105040851

- Down regulated genes

c156623\_g1(-0.57504)

- egu:105040851

- Down regulated genes

c156623\_g1(-0.57504)

- egu:105040851

- Down regulated genes

c156623\_g1(-0.57504)

- egu:105040851

- Down regulated genes

c156623\_g1(-0.57504)

- egu:105040851

- Down regulated genes

c156623\_g1(-0.57504)

- egu:105040851

- Down regulated genes

c156623\_g1(-0.57504)

- egu:105040851

- Down regulated genes

c156623\_g1(-0.57504)

- egu:105040851

- Down regulated genes

c156623\_g1(-0.57504)

- egu:105040851

- Down regulated genes

c156623\_g1(-0.57504)

- egu:105040851

- Down regulated genes

c156623\_g1(-0.57504)

- egu:105048315

- Down regulated genes

c164323\_g1(-1.7412)
- egu:105044215

- Down regulated genes

c148702\_g1(-0.95101)
- egu:105040213

- Down regulated genes

c151794\_g1(-1.5653)
- egu:105042530

- Down regulated genes

c2875\_g1(-1.3428)
- egu:105041077

- Down regulated genes

c150017\_g1(-1.0519)
- egu:105039895

- Down regulated genes

c134603\_g2(-2.1499)
- egu:105047165

- Down regulated genes

c164323\_g2(-1.7478)
- egu:105047853

- Down regulated genes

c2875\_g2(-1.116)

- egu:105048315

- Down regulated genes

c164323\_g1(-1.7412)
- egu:105044215

- Down regulated genes

c148702\_g1(-0.95101)
- egu:105040213

- Down regulated genes

c151794\_g1(-1.5653)
- egu:105042530

- Down regulated genes

c2875\_g1(-1.3428)
- egu:105041077

- Down regulated genes

c150017\_g1(-1.0519)
- egu:105039895

- Down regulated genes

c134603\_g2(-2.1499)
- egu:105047165

- Down regulated genes

c164323\_g2(-1.7478)
- egu:105047853

- Down regulated genes

c2875\_g2(-1.116)

- egu:105048315

- Down regulated genes

c164323\_g1(-1.7412)
- egu:105044215

- Down regulated genes

c148702\_g1(-0.95101)
- egu:105040213

- Down regulated genes

c151794\_g1(-1.5653)
- egu:105042530

- Down regulated genes

c2875\_g1(-1.3428)
- egu:105041077

- Down regulated genes

c150017\_g1(-1.0519)
- egu:105039895

- Down regulated genes

c134603\_g2(-2.1499)
- egu:105047165

- Down regulated genes

c164323\_g2(-1.7478)
- egu:105047853

- Down regulated genes

c2875\_g2(-1.116)

- egu:105048315

- Down regulated genes

c164323\_g1(-1.7412)
- egu:105044215

- Down regulated genes

c148702\_g1(-0.95101)
- egu:105040213

- Down regulated genes

c151794\_g1(-1.5653)
- egu:105042530

- Down regulated genes

c2875\_g1(-1.3428)
- egu:105041077

- Down regulated genes

c150017\_g1(-1.0519)
- egu:105039895

- Down regulated genes

c134603\_g2(-2.1499)
- egu:105047165

- Down regulated genes

c164323\_g2(-1.7478)
- egu:105047853

- Down regulated genes

c2875\_g2(-1.116)

- egu:105048315

- Down regulated genes

c164323\_g1(-1.7412)
- egu:105044215

- Down regulated genes

c148702\_g1(-0.95101)
- egu:105040213

- Down regulated genes

c151794\_g1(-1.5653)
- egu:105042530

- Down regulated genes

c2875\_g1(-1.3428)
- egu:105041077

- Down regulated genes

c150017\_g1(-1.0519)
- egu:105039895

- Down regulated genes

c134603\_g2(-2.1499)
- egu:105047165

- Down regulated genes

c164323\_g2(-1.7478)
- egu:105047853

- Down regulated genes

c2875\_g2(-1.116)

- egu:105048315

- Down regulated genes

c164323\_g1(-1.7412)
- egu:105044215

- Down regulated genes

c148702\_g1(-0.95101)
- egu:105040213

- Down regulated genes

c151794\_g1(-1.5653)
- egu:105042530

- Down regulated genes

c2875\_g1(-1.3428)
- egu:105041077

- Down regulated genes

c150017\_g1(-1.0519)
- egu:105039895

- Down regulated genes

c134603\_g2(-2.1499)
- egu:105047165

- Down regulated genes

c164323\_g2(-1.7478)
- egu:105047853

- Down regulated genes

c2875\_g2(-1.116)

- egu:105048315

- Down regulated genes

c164323\_g1(-1.7412)
- egu:105044215

- Down regulated genes

c148702\_g1(-0.95101)
- egu:105040213

- Down regulated genes

c151794\_g1(-1.5653)
- egu:105042530

- Down regulated genes

c2875\_g1(-1.3428)
- egu:105041077

- Down regulated genes

c150017\_g1(-1.0519)
- egu:105039895

- Down regulated genes

c134603\_g2(-2.1499)
- egu:105047165

- Down regulated genes

c164323\_g2(-1.7478)
- egu:105047853

- Down regulated genes

c2875\_g2(-1.116)

- egu:105048315

- Down regulated genes

c164323\_g1(-1.7412)
- egu:105044215

- Down regulated genes

c148702\_g1(-0.95101)
- egu:105040213

- Down regulated genes

c151794\_g1(-1.5653)
- egu:105042530

- Down regulated genes

c2875\_g1(-1.3428)
- egu:105041077

- Down regulated genes

c150017\_g1(-1.0519)
- egu:105039895

- Down regulated genes

c134603\_g2(-2.1499)
- egu:105047165

- Down regulated genes

c164323\_g2(-1.7478)
- egu:105047853

- Down regulated genes

c2875\_g2(-1.116)

- egu:105048315

- Down regulated genes

c164323\_g1(-1.7412)
- egu:105044215

- Down regulated genes

c148702\_g1(-0.95101)
- egu:105040213

- Down regulated genes

c151794\_g1(-1.5653)
- egu:105042530

- Down regulated genes

c2875\_g1(-1.3428)
- egu:105041077

- Down regulated genes

c150017\_g1(-1.0519)
- egu:105039895

- Down regulated genes

c134603\_g2(-2.1499)
- egu:105047165

- Down regulated genes

c164323\_g2(-1.7478)
- egu:105047853

- Down regulated genes

c2875\_g2(-1.116)

- egu:105048315

- Down regulated genes

c164323\_g1(-1.7412)
- egu:105044215

- Down regulated genes

c148702\_g1(-0.95101)
- egu:105040213

- Down regulated genes

c151794\_g1(-1.5653)
- egu:105042530

- Down regulated genes

c2875\_g1(-1.3428)
- egu:105041077

- Down regulated genes

c150017\_g1(-1.0519)
- egu:105039895

- Down regulated genes

c134603\_g2(-2.1499)
- egu:105047165

- Down regulated genes

c164323\_g2(-1.7478)
- egu:105047853

- Down regulated genes

c2875\_g2(-1.116)

- egu:105048315

- Down regulated genes

c164323\_g1(-1.7412)
- egu:105044215

- Down regulated genes

c148702\_g1(-0.95101)
- egu:105040213

- Down regulated genes

c151794\_g1(-1.5653)
- egu:105042530

- Down regulated genes

c2875\_g1(-1.3428)
- egu:105041077

- Down regulated genes

c150017\_g1(-1.0519)
- egu:105039895

- Down regulated genes

c134603\_g2(-2.1499)
- egu:105047165

- Down regulated genes

c164323\_g2(-1.7478)
- egu:105047853

- Down regulated genes

c2875\_g2(-1.116)

- egu:105048315

- Down regulated genes

c164323\_g1(-1.7412)
- egu:105044215

- Down regulated genes

c148702\_g1(-0.95101)
- egu:105040213

- Down regulated genes

c151794\_g1(-1.5653)
- egu:105042530

- Down regulated genes

c2875\_g1(-1.3428)
- egu:105041077

- Down regulated genes

c150017\_g1(-1.0519)
- egu:105039895

- Down regulated genes

c134603\_g2(-2.1499)
- egu:105047165

- Down regulated genes

c164323\_g2(-1.7478)
- egu:105047853

- Down regulated genes

c2875\_g2(-1.116)

- egu:105056650

- Down regulated genes

c163774\_g1(-0.59036)

- egu:105056650

- Down regulated genes

c163774\_g1(-0.59036)

- egu:105038852

- Down regulated genes

c137214\_g2(-0.74276)

- egu:105058894

- Down regulated genes

c159032\_g1(-1.1839)

- egu:105058894

- Down regulated genes

c159032\_g1(-1.1839)

- egu:105056650

- Down regulated genes

c163774\_g1(-0.59036)

- egu:105056650

- Down regulated genes

c163774\_g1(-0.59036)

- egu:105057721

- Down regulated genes

c166298\_g1(-0.77074)

- egu:105038832

- Down regulated genes

c166072\_g1(-1.2063)

- egu:105038832

- Down regulated genes

c166072\_g1(-1.2063)

- egu:105038832

- Down regulated genes

c166072\_g1(-1.2063)

- egu:105038832

- Down regulated genes

c166072\_g1(-1.2063)

- egu:105038832

- Down regulated genes

c166072\_g1(-1.2063)

- egu:105038832

- Down regulated genes

c166072\_g1(-1.2063)

- egu:105038832

- Down regulated genes

c166072\_g1(-1.2063)

- egu:105038832

- Down regulated genes

c166072\_g1(-1.2063)

- egu:105038832

- Down regulated genes

c166072\_g1(-1.2063)

- egu:105038832

- Down regulated genes

c166072\_g1(-1.2063)

- egu:105038832

- Down regulated genes

c166072\_g1(-1.2063)

- egu:105038832

- Down regulated genes

c166072\_g1(-1.2063)

- egu:105038832

- Down regulated genes

c166072\_g1(-1.2063)

- egu:105038832

- Down regulated genes

c166072\_g1(-1.2063)

- egu:105038832

- Down regulated genes

c166072\_g1(-1.2063)

- egu:105038832

- Down regulated genes

c166072\_g1(-1.2063)

- egu:105038832

- Down regulated genes

c166072\_g1(-1.2063)

- egu:105038832

- Down regulated genes

c166072\_g1(-1.2063)

- egu:105038832

- Down regulated genes

c166072\_g1(-1.2063)

- egu:105038832

- Down regulated genes

c166072\_g1(-1.2063)

- egu:105038832

- Down regulated genes

c166072\_g1(-1.2063)

- egu:105057721

- Down regulated genes

c166298\_g1(-0.77074)

- egu:105038832

- Down regulated genes

c166072\_g1(-1.2063)

- egu:105038832

- Down regulated genes

c166072\_g1(-1.2063)

- egu:105038832

- Down regulated genes

c166072\_g1(-1.2063)

- egu:105038832

- Down regulated genes

c166072\_g1(-1.2063)

- egu:105038832

- Down regulated genes

c166072\_g1(-1.2063)

- egu:105038832

- Down regulated genes

c166072\_g1(-1.2063)

- egu:105038832

- Down regulated genes

c166072\_g1(-1.2063)

- egu:105038832

- Down regulated genes

c166072\_g1(-1.2063)

- egu:105038832

- Down regulated genes

c166072\_g1(-1.2063)

- egu:105038832

- Down regulated genes

c166072\_g1(-1.2063)

- egu:105038832

- Down regulated genes

c166072\_g1(-1.2063)

- egu:105038832

- Down regulated genes

c166072\_g1(-1.2063)

- egu:105038832

- Down regulated genes

c166072\_g1(-1.2063)

- egu:105038832

- Down regulated genes

c166072\_g1(-1.2063)

- egu:105038832

- Down regulated genes

c166072\_g1(-1.2063)

- egu:105038832

- Down regulated genes

c166072\_g1(-1.2063)

- egu:105038832

- Down regulated genes

c166072\_g1(-1.2063)

- egu:105038832

- Down regulated genes

c166072\_g1(-1.2063)

- egu:105038832

- Down regulated genes

c166072\_g1(-1.2063)

- egu:105038832

- Down regulated genes

c166072\_g1(-1.2063)

- egu:105038832

- Down regulated genes

c166072\_g1(-1.2063)

- egu:105058545

- Down regulated genes

c166557\_g2(-1.5787) c166557\_g1(-1.7086)

- egu:105058545

- Down regulated genes

c166557\_g2(-1.5787) c166557\_g1(-1.7086)

- egu:105058545

- Down regulated genes

c166557\_g2(-1.5787) c166557\_g1(-1.7086)

- egu:105058545

- Down regulated genes

c166557\_g2(-1.5787) c166557\_g1(-1.7086)

- egu:105058545

- Down regulated genes

c166557\_g2(-1.5787) c166557\_g1(-1.7086)

- egu:105058545

- Down regulated genes

c166557\_g2(-1.5787) c166557\_g1(-1.7086)

- egu:105058545

- Down regulated genes

c166557\_g2(-1.5787) c166557\_g1(-1.7086)

- egu:105058545

- Down regulated genes

c166557\_g2(-1.5787) c166557\_g1(-1.7086)

- egu:105058545

- Down regulated genes

c166557\_g2(-1.5787) c166557\_g1(-1.7086)

- egu:105058545

- Down regulated genes

c166557\_g2(-1.5787) c166557\_g1(-1.7086)

- egu:105058545

- Down regulated genes

c166557\_g2(-1.5787) c166557\_g1(-1.7086)

- egu:105045201

- Down regulated genes

c146228\_g1(-0.90789)
- egu:105044229

- Down regulated genes

c164821\_g1(-2.5552)
- egu:105045835

- Down regulated genes

c171033\_g3(-0.62809)
- egu:105042390

- Down regulated genes

c173060\_g2(-1.2692)
- egu:105034542

- Down regulated genes

c174706\_g1(-0.9061)
- egu:105044265

- Down regulated genes

c173942\_g4(-2.7483)

- egu:105053482

- Down regulated genes

c166982\_g1(-0.61184)

- egu:105053482

- Down regulated genes

c166982\_g1(-0.61184)

- egu:105053482

- Down regulated genes

c166982\_g1(-0.61184)

- egu:105053482

- Down regulated genes

c166982\_g1(-0.61184)

- egu:105053482

- Down regulated genes

c166982\_g1(-0.61184)

- egu:105053482

- Down regulated genes

c166982\_g1(-0.61184)

- egu:105053482

- Down regulated genes

c166982\_g1(-0.61184)

- egu:105053482

- Down regulated genes

c166982\_g1(-0.61184)

- egu:105053482

- Down regulated genes

c166982\_g1(-0.61184)

- egu:105053482

- Down regulated genes

c166982\_g1(-0.61184)

- egu:105053482

- Down regulated genes

c166982\_g1(-0.61184)

- egu:105053482

- Down regulated genes

c166982\_g1(-0.61184)

- egu:105053482

- Down regulated genes

c166982\_g1(-0.61184)

- egu:105053482

- Down regulated genes

c166982\_g1(-0.61184)

- egu:105053482

- Down regulated genes

c166982\_g1(-0.61184)

- egu:105053482

- Down regulated genes

c166982\_g1(-0.61184)

- egu:105053482

- Down regulated genes

c166982\_g1(-0.61184)

- egu:105053482

- Down regulated genes

c166982\_g1(-0.61184)

- egu:105053482

- Down regulated genes

c166982\_g1(-0.61184)

- egu:105053482

- Down regulated genes

c166982\_g1(-0.61184)

- egu:105053482

- Down regulated genes

c166982\_g1(-0.61184)

- egu:105046827

- Down regulated genes

c166358\_g1(-1.0276)

- egu:105036454

- Down regulated genes

c164056\_g1(-0.83646)

- egu:105055609

- Down regulated genes

c152224\_g1(-0.46463)

- egu:105040851

- Down regulated genes

c156623\_g1(-0.57504)

- egu:105040851

- Down regulated genes

c156623\_g1(-0.57504)

- egu:105040851

- Down regulated genes

c156623\_g1(-0.57504)

- egu:105040851

- Down regulated genes

c156623\_g1(-0.57504)

- egu:105040851

- Down regulated genes

c156623\_g1(-0.57504)

- egu:105040851

- Down regulated genes

c156623\_g1(-0.57504)

- egu:105040851

- Down regulated genes

c156623\_g1(-0.57504)

- egu:105040851

- Down regulated genes

c156623\_g1(-0.57504)

- egu:105040851

- Down regulated genes

c156623\_g1(-0.57504)

- egu:105040851

- Down regulated genes

c156623\_g1(-0.57504)

- egu:105040851

- Down regulated genes

c156623\_g1(-0.57504)

- egu:105040851

- Down regulated genes

c156623\_g1(-0.57504)

- egu:105046147

- Down regulated genes

c122896\_g1(-0.75457)

- egu:105046147

- Down regulated genes

c122896\_g1(-0.75457)

- egu:105046147

- Down regulated genes

c122896\_g1(-0.75457)

- egu:105046147

- Down regulated genes

c122896\_g1(-0.75457)

- egu:105046147

- Down regulated genes

c122896\_g1(-0.75457)

- egu:105046147

- Down regulated genes

c122896\_g1(-0.75457)

- egu:105046147

- Down regulated genes

c122896\_g1(-0.75457)

- egu:105046147

- Down regulated genes

c122896\_g1(-0.75457)

- egu:105046147

- Down regulated genes

c122896\_g1(-0.75457)

- egu:105046147

- Down regulated genes

c122896\_g1(-0.75457)

- egu:105046147

- Down regulated genes

c122896\_g1(-0.75457)

- egu:105046147

- Down regulated genes

c122896\_g1(-0.75457)

- egu:105046147

- Down regulated genes

c122896\_g1(-0.75457)

- egu:105046147

- Down regulated genes

c122896\_g1(-0.75457)

- egu:105046147

- Down regulated genes

c122896\_g1(-0.75457)

- egu:105046147

- Down regulated genes

c122896\_g1(-0.75457)

- egu:105046147

- Down regulated genes

c122896\_g1(-0.75457)

- egu:105046147

- Down regulated genes

c122896\_g1(-0.75457)

- egu:105046147

- Down regulated genes

c122896\_g1(-0.75457)

- egu:105046147

- Down regulated genes

c122896\_g1(-0.75457)

- egu:105046147

- Down regulated genes

c122896\_g1(-0.75457)

- egu:105046147

- Down regulated genes

c122896\_g1(-0.75457)

- egu:105046147

- Down regulated genes

c122896\_g1(-0.75457)

- egu:105046147

- Down regulated genes

c122896\_g1(-0.75457)

- egu:105035100

- Down regulated genes

c166497\_g1(-1.4686)

- egu:105035100

- Down regulated genes

c166497\_g1(-1.4686)

- egu:105035100

- Down regulated genes

c166497\_g1(-1.4686)

- egu:105035100

- Down regulated genes

c166497\_g1(-1.4686)

- egu:105035100

- Down regulated genes

c166497\_g1(-1.4686)

- egu:105035100

- Down regulated genes

c166497\_g1(-1.4686)

- egu:105035100

- Down regulated genes

c166497\_g1(-1.4686)

- egu:105035100

- Down regulated genes

c166497\_g1(-1.4686)

- egu:105035100

- Down regulated genes

c166497\_g1(-1.4686)

- egu:105035100

- Down regulated genes

c166497\_g1(-1.4686)

- egu:105035100

- Down regulated genes

c166497\_g1(-1.4686)

- egu:105035100

- Down regulated genes

c166497\_g1(-1.4686)

- egu:105035100

- Down regulated genes

c166497\_g1(-1.4686)

- egu:105035100

- Down regulated genes

c166497\_g1(-1.4686)

- egu:105035100

- Down regulated genes

c166497\_g1(-1.4686)

- egu:105035100

- Down regulated genes

c166497\_g1(-1.4686)

- egu:105035100

- Down regulated genes

c166497\_g1(-1.4686)

- egu:105035100

- Down regulated genes

c166497\_g1(-1.4686)

- egu:105035100

- Down regulated genes

c166497\_g1(-1.4686)

- egu:105035100

- Down regulated genes

c166497\_g1(-1.4686)

- egu:105035100

- Down regulated genes

c166497\_g1(-1.4686)

- egu:105035100

- Down regulated genes

c166497\_g1(-1.4686)

- egu:105035100

- Down regulated genes

c166497\_g1(-1.4686)

- egu:105035100

- Down regulated genes

c166497\_g1(-1.4686)

- egu:105035100

- Down regulated genes

c166497\_g1(-1.4686)

- egu:105035100

- Down regulated genes

c166497\_g1(-1.4686)

- egu:105035100

- Down regulated genes

c166497\_g1(-1.4686)

- egu:105035100

- Down regulated genes

c166497\_g1(-1.4686)

- egu:105035100

- Down regulated genes

c166497\_g1(-1.4686)

- egu:105035100

- Down regulated genes

c166497\_g1(-1.4686)

- egu:105035100

- Down regulated genes

c166497\_g1(-1.4686)

- egu:105035100

- Down regulated genes

c166497\_g1(-1.4686)

- egu:105035100

- Down regulated genes

c166497\_g1(-1.4686)

- egu:105035100

- Down regulated genes

c166497\_g1(-1.4686)

- egu:105035100

- Down regulated genes

c166497\_g1(-1.4686)

- egu:105035100

- Down regulated genes

c166497\_g1(-1.4686)

- egu:105035100

- Down regulated genes

c166497\_g1(-1.4686)

- egu:105035100

- Down regulated genes

c166497\_g1(-1.4686)

- egu:105035100

- Down regulated genes

c166497\_g1(-1.4686)

- egu:105035100

- Down regulated genes

c166497\_g1(-1.4686)

- egu:105035100

- Down regulated genes

c166497\_g1(-1.4686)

- egu:105035100

- Down regulated genes

c166497\_g1(-1.4686)

- egu:105059048

- Down regulated genes

c167963\_g1(-0.67401)

- egu:105055982

- Down regulated genes

c158576\_g4(-2.0092)

- egu:105043957

- Down regulated genes

c162118\_g1(-0.64519)
- egu:105059048

- Down regulated genes

c167963\_g1(-0.67401)

- egu:105043957

- Down regulated genes

c162118\_g1(-0.64519)
- egu:105059048

- Down regulated genes

c167963\_g1(-0.67401)

- egu:105043957

- Down regulated genes

c162118\_g1(-0.64519)
- egu:105059048

- Down regulated genes

c167963\_g1(-0.67401)

- egu:105043957

- Down regulated genes

c162118\_g1(-0.64519)
- egu:105059048

- Down regulated genes

c167963\_g1(-0.67401)

- egu:105043957

- Down regulated genes

c162118\_g1(-0.64519)
- egu:105059048

- Down regulated genes

c167963\_g1(-0.67401)

- egu:105043957

- Down regulated genes

c162118\_g1(-0.64519)
- egu:105059048

- Down regulated genes

c167963\_g1(-0.67401)

- egu:105043957

- Down regulated genes

c162118\_g1(-0.64519)
- egu:105059048

- Down regulated genes

c167963\_g1(-0.67401)

- egu:105043957

- Down regulated genes

c162118\_g1(-0.64519)
- egu:105059048

- Down regulated genes

c167963\_g1(-0.67401)

- egu:105043957

- Down regulated genes

c162118\_g1(-0.64519)
- egu:105059048

- Down regulated genes

c167963\_g1(-0.67401)

- egu:105043957

- Down regulated genes

c162118\_g1(-0.64519)
- egu:105059048

- Down regulated genes

c167963\_g1(-0.67401)

- egu:105043957

- Down regulated genes

c162118\_g1(-0.64519)
- egu:105059048

- Down regulated genes

c167963\_g1(-0.67401)

Close
