# Supplementary material for: Physiological and transcriptomic responses of Lanzhou Lily (Lilium davidii, var. unicolor) to cold stress
Source: PLoS One. 2020 Jan 23;15(1):e0227921. doi: 10.1371/journal.pone.0227921 (PMC6977731; doi:10.1371/journal.pone.0227921)
Supplement: S2 Zip — (Zip). CK: control (20°C); LT: low temperature (4°C). (ZIP) [file pone.0227921.s012.zip › S2 Zip/LTvsCK_DOWN/src/egu02010.html]

egu02010


- egu:105056548

- Down regulated genes

c157873\_g1(-0.89734)
- egu:105059124

- Down regulated genes

c169285\_g1(-1.4103)
- egu:105040552

- Down regulated genes

c161768\_g1(-1.1189)
- egu:105055560

- Down regulated genes

c169285\_g2(-1.2694)

- egu:105052956

- Down regulated genes

c173719\_g3(-1.3909)

Close
