# Supplementary material for: Physiological and transcriptomic responses of Lanzhou Lily (Lilium davidii, var. unicolor) to cold stress
Source: PLoS One. 2020 Jan 23;15(1):e0227921. doi: 10.1371/journal.pone.0227921 (PMC6977731; doi:10.1371/journal.pone.0227921)
Supplement: S2 Zip — (Zip). CK: control (20°C); LT: low temperature (4°C). (ZIP) [file pone.0227921.s012.zip › S2 Zip/LTvsCK_DOWN/src/egu03010.html]

egu03010


- egu:105032412

- Down regulated genes

c178004\_g1(-1.2941) c155204\_g1(-1.6155)

- egu:105045280

- Down regulated genes

c134157\_g1(-0.54254)

- egu:105044673

- Down regulated genes

c168022\_g1(-0.86799)

- egu:105036502

- Down regulated genes

c132904\_g1(-0.80137)

- egu:105041287

- Down regulated genes

c160112\_g1(-0.81377)

- egu:105052800

- Down regulated genes

c163280\_g1(-1.1067)

- egu:105043313

- Down regulated genes

c143884\_g1(-0.92949)

- egu:105034395

- Down regulated genes

c145214\_g1(-0.95484)

- egu:105041074

- Down regulated genes

c160468\_g1(-0.8155)

- egu:105040137

- Down regulated genes

c134374\_g1(-1.4146)

- egu:105034390

- Down regulated genes

c157065\_g1(-0.78293)

- egu:105052661

- Down regulated genes

c27497\_g1(-0.86786)

- egu:105056818

- Down regulated genes

c143129\_g1(-1.1394)

- egu:105061188

- Down regulated genes

c161348\_g1(-0.90125)

- egu:105039272

- Down regulated genes

c101971\_g1(-1.4075)

- egu:105044181

- Down regulated genes

c161542\_g1(-2.178)

- egu:105055772

- Down regulated genes

c163095\_g3(-1.4321)

- egu:105035316

- Down regulated genes

c151905\_g1(-1.2279)

- egu:105059074

- Down regulated genes

c156427\_g1(-1.4622)

- egu:105048206

- Down regulated genes

c94348\_g1(-1.1872)
- egu:105036935

- Down regulated genes

c154494\_g1(-0.67899)

- egu:105046043

- Down regulated genes

c116569\_g1(-1.1318)

- egu:105053938

- Down regulated genes

c106647\_g1(-1.4165)

- egu:12079413

- Down regulated genes

c131320\_g1(-1.0757)

- egu:105040763

- Down regulated genes

c150484\_g1(-1.2259)

- egu:105060920

- Down regulated genes

c158858\_g1(-0.56843)
- egu:105048988

- Down regulated genes

c141240\_g1(-0.99676)

- egu:105059189

- Down regulated genes

c224200\_g1(-1.0692)

- egu:105048529

- Down regulated genes

c141711\_g1(-0.7314)

- egu:105033340

- Down regulated genes

c48496\_g1(-1.1351)

- egu:105045120

- Down regulated genes

c172832\_g1(-1.2197)

- egu:105061575

- Down regulated genes

c159369\_g1(-1.4106)

- egu:105060039

- Down regulated genes

c161109\_g1(-1.0826)

- egu:105032797

- Down regulated genes

c158737\_g2(-0.99467)

- egu:105041319

- Down regulated genes

c138659\_g1(-1.068)

- egu:105034754

- Down regulated genes

c132540\_g1(-0.89075)

- egu:105047719

- Down regulated genes

c147799\_g1(-0.69882)
- egu:105044970

- Down regulated genes

c133119\_g1(-0.95577)

- egu:105047611

- Down regulated genes

c142600\_g1(-0.92123)

- egu:105042443

- Down regulated genes

c140525\_g1(-0.9854)

- egu:105034999

- Down regulated genes

c142521\_g1(-0.69455)

- egu:105057725

- Down regulated genes

c156664\_g1(-0.74444)

Close
