# Supplementary material for: Physiological and transcriptomic responses of Lanzhou Lily (Lilium davidii, var. unicolor) to cold stress
Source: PLoS One. 2020 Jan 23;15(1):e0227921. doi: 10.1371/journal.pone.0227921 (PMC6977731; doi:10.1371/journal.pone.0227921)
Supplement: S2 Zip — (Zip). CK: control (20°C); LT: low temperature (4°C). (ZIP) [file pone.0227921.s012.zip › S2 Zip/LTvsCK_DOWN/src/egu03013.html]

egu03013


- egu:105038511

- Down regulated genes

c145727\_g1(-0.90197)

- egu:105048719

- Down regulated genes

c852\_g1(-0.93812)

- egu:105056889

- Down regulated genes

c166848\_g1(-1.1895)

- egu:105037047

- Down regulated genes

c162257\_g1(-0.67237)

- egu:105035877

- Down regulated genes

c121960\_g1(-0.97761)
- egu:105050147

- Down regulated genes

c168902\_g1(-0.49457)

- egu:105032882

- Down regulated genes

c144240\_g2(-1.675) c144240\_g1(-1.7804)

Close
