# Supplementary material for: Physiological and transcriptomic responses of Lanzhou Lily (Lilium davidii, var. unicolor) to cold stress
Source: PLoS One. 2020 Jan 23;15(1):e0227921. doi: 10.1371/journal.pone.0227921 (PMC6977731; doi:10.1371/journal.pone.0227921)
Supplement: S2 Zip — (Zip). CK: control (20°C); LT: low temperature (4°C). (ZIP) [file pone.0227921.s012.zip › S2 Zip/LTvsCK_DOWN/src/egu03015.html]

egu03015


- egu:105038511

- Down regulated genes

c145727\_g1(-0.90197)

- egu:105048719

- Down regulated genes

c852\_g1(-0.93812)

- egu:105041328

- Down regulated genes

c148939\_g1(-0.6269)

- egu:105036286

- Down regulated genes

c155315\_g1(-0.73698)

- egu:105051301

- Down regulated genes

c155560\_g1(-0.65087)

- egu:105050147

- Down regulated genes

c168902\_g1(-0.49457)
- egu:105035877

- Down regulated genes

c121960\_g1(-0.97761)

- egu:105039096

- Down regulated genes

c116624\_g1(-0.60134)

- egu:105050147

- Down regulated genes

c168902\_g1(-0.49457)
- egu:105035877

- Down regulated genes

c121960\_g1(-0.97761)

- egu:105035556

- Down regulated genes

c224125\_g1(-0.72358)
- egu:105056201

- Down regulated genes

c158775\_g1(-0.7853) c165735\_g1(-0.63084)

- egu:105046347

- Down regulated genes

c153337\_g3(-0.9011)

Close
