# Supplementary material for: Physiological and transcriptomic responses of Lanzhou Lily (Lilium davidii, var. unicolor) to cold stress
Source: PLoS One. 2020 Jan 23;15(1):e0227921. doi: 10.1371/journal.pone.0227921 (PMC6977731; doi:10.1371/journal.pone.0227921)
Supplement: S2 Zip — (Zip). CK: control (20°C); LT: low temperature (4°C). (ZIP) [file pone.0227921.s012.zip › S2 Zip/LTvsCK_DOWN/src/egu03018.html]

egu03018


- egu:105040019

- Down regulated genes

c159391\_g1(-0.99017) c159391\_g2(-0.72882)

- egu:105060207

- Down regulated genes

c166194\_g5(-2.2507)
- egu:105050714

- Down regulated genes

c132311\_g1(-0.88251)

- egu:105053252

- Down regulated genes

c165808\_g2(-3.6165)

- egu:105050147

- Down regulated genes

c168902\_g1(-0.49457)
- egu:105035877

- Down regulated genes

c121960\_g1(-0.97761)

Close
