# Supplementary material for: Physiological and transcriptomic responses of Lanzhou Lily (Lilium davidii, var. unicolor) to cold stress
Source: PLoS One. 2020 Jan 23;15(1):e0227921. doi: 10.1371/journal.pone.0227921 (PMC6977731; doi:10.1371/journal.pone.0227921)
Supplement: S2 Zip — (Zip). CK: control (20°C); LT: low temperature (4°C). (ZIP) [file pone.0227921.s012.zip › S2 Zip/LTvsCK_DOWN/src/egu03040.html]

egu03040


- egu:105043591

- Down regulated genes

c160059\_g1(-0.97862) c168382\_g1(-0.66)

- egu:105039206

- Down regulated genes

c174892\_g3(-0.63271) c174892\_g1(-0.58327)

- egu:105053459

- Down regulated genes

c83498\_g1(-1.8097)

- egu:105049382

- Down regulated genes

c170687\_g2(-0.72556)

- egu:105058417

- Down regulated genes

c157385\_g2(-0.53356)

- egu:105056954

- Down regulated genes

c171346\_g1(-0.97358)

- egu:105053459

- Down regulated genes

c83498\_g1(-1.8097)

- egu:105038221

- Down regulated genes

c155072\_g1(-0.51816)

Close
