# Supplementary material for: Physiological and transcriptomic responses of Lanzhou Lily (Lilium davidii, var. unicolor) to cold stress
Source: PLoS One. 2020 Jan 23;15(1):e0227921. doi: 10.1371/journal.pone.0227921 (PMC6977731; doi:10.1371/journal.pone.0227921)
Supplement: S2 Zip — (Zip). CK: control (20°C); LT: low temperature (4°C). (ZIP) [file pone.0227921.s012.zip › S2 Zip/LTvsCK_DOWN/src/egu03060.html]

egu03060


- egu:105044437

- Down regulated genes

c123568\_g1(-0.7811)

- egu:105055789

- Down regulated genes

c132523\_g1(-0.91304)

- egu:105038252

- Down regulated genes

c170040\_g1(-0.79192)

- egu:105038252

- Down regulated genes

c170040\_g1(-0.79192)

- egu:105050132

- Down regulated genes

c112651\_g1(-1.074)

Close
