# Supplementary material for: Physiological and transcriptomic responses of Lanzhou Lily (Lilium davidii, var. unicolor) to cold stress
Source: PLoS One. 2020 Jan 23;15(1):e0227921. doi: 10.1371/journal.pone.0227921 (PMC6977731; doi:10.1371/journal.pone.0227921)
Supplement: S1 Zip — (Zip). CK: control (20°C); LT: low temperature (4°C). (ZIP) [file pone.0227921.s011.zip › S1 Zip/src/egu04070.html]

egu04070


- egu:105057333

- Up regulated genes

c101051\_g1(1.3264) c166855\_g1(2.4733)

- egu:105048729

- Up regulated genes

c162261\_g1(0.7909)

- egu:105056668

- Up regulated genes

c152208\_g2(0.80623)

- egu:105056668

- Up regulated genes

c152208\_g2(0.80623)

- egu:105036034

- Up regulated genes

c135007\_g1(0.70355)

- egu:105036034

- Up regulated genes

c135007\_g1(0.70355)

- egu:105036034

- Up regulated genes

c135007\_g1(0.70355)

- egu:105055908

- Up regulated genes

c104329\_g1(0.68484)

Close
