# Supplementary material for: Physiological and transcriptomic responses of Lanzhou Lily (Lilium davidii, var. unicolor) to cold stress
Source: PLoS One. 2020 Jan 23;15(1):e0227921. doi: 10.1371/journal.pone.0227921 (PMC6977731; doi:10.1371/journal.pone.0227921)
Supplement: S1 Zip — (Zip). CK: control (20°C); LT: low temperature (4°C). (ZIP) [file pone.0227921.s011.zip › S1 Zip/src/egu04075.html]

egu04075


- egu:105055597

- Up regulated genes

c142840\_g2(0.93965)

- egu:105032167

- Up regulated genes

c164022\_g1(0.71943) c160379\_g1(1.3305)

- egu:105061364

- Up regulated genes

c142660\_g1(0.76411)

- egu:105043078

- Up regulated genes

c113301\_g1(1.0603)

- egu:105040420

- Up regulated genes

c172818\_g4(2.4838)
- egu:105055850

- Up regulated genes

c146349\_g1(0.6884)
- egu:105032276

- Up regulated genes

c170101\_g1(4.1204)
- egu:105040525

- Up regulated genes

c164404\_g1(0.77771)
- egu:105046557

- Up regulated genes

c147714\_g1(0.84432)
- egu:105059132

- Up regulated genes

c150290\_g1(0.73548)

- egu:105042113

- Up regulated genes

c174574\_g2(0.51986)

- egu:105052243

- Up regulated genes

c87233\_g1(2.4494)

- egu:105056896

- Up regulated genes

c162356\_g1(2.1166) c96913\_g1(2.5475)
- egu:105043050

- Up regulated genes

c159509\_g4(2.2838)
- egu:105057151

- Up regulated genes

c156282\_g1(0.85087)

- egu:105043791

- Up regulated genes

c147612\_g1(1.832)

- egu:105059035

- Up regulated genes

c76349\_g1(1.0227)

- egu:105060874

- Up regulated genes

c157415\_g1(0.93474)

- egu:105033019

- Up regulated genes

c162387\_g1(1.3545)

- egu:105045549

- Up regulated genes

c163360\_g1(2.2574)

- egu:105051062

- Up regulated genes

c163407\_g1(2.1752)
- egu:105058608

- Up regulated genes

c152357\_g1(0.45351)
- egu:105035162

- Up regulated genes

c138921\_g1(0.77683)

- egu:105041307

- Up regulated genes

c132627\_g1(0.90883) c103312\_g1(1.1975) c157217\_g1(0.53617)

- egu:105048226

- Up regulated genes

c169345\_g1(0.8931)

- egu:105032733

- Up regulated genes

c154443\_g1(0.91637)

- egu:105052748

- Up regulated genes

c146802\_g3(1.2826)
- egu:105038536

- Up regulated genes

c158029\_g1(1.65)

- egu:105050174

- Up regulated genes

c155948\_g1(0.98648)

- egu:105035319

- Up regulated genes

c149381\_g1(0.68976)

- egu:105046997

- Up regulated genes

c165448\_g1(0.62187)

- egu:105040420

- Up regulated genes

c172818\_g4(2.4838)
- egu:105055850

- Up regulated genes

c146349\_g1(0.6884)
- egu:105032276

- Up regulated genes

c170101\_g1(4.1204)
- egu:105040525

- Up regulated genes

c164404\_g1(0.77771)
- egu:105046557

- Up regulated genes

c147714\_g1(0.84432)
- egu:105059132

- Up regulated genes

c150290\_g1(0.73548)

- egu:105043524

- Up regulated genes

c167605\_g2(0.94617) c138925\_g1(2.444)

- egu:105044079

- Up regulated genes

c140543\_g1(0.81986)

- egu:105060907

- Up regulated genes

c146567\_g1(0.66219)

Close
