# Supplementary material for: Physiological and transcriptomic responses of Lanzhou Lily (Lilium davidii, var. unicolor) to cold stress
Source: PLoS One. 2020 Jan 23;15(1):e0227921. doi: 10.1371/journal.pone.0227921 (PMC6977731; doi:10.1371/journal.pone.0227921)
Supplement: S1 Zip — (Zip). CK: control (20°C); LT: low temperature (4°C). (ZIP) [file pone.0227921.s011.zip › S1 Zip/src/egu04120.html]

egu04120


- egu:105045214

- Up regulated genes

c114328\_g1(1.1213)

- egu:105043171

- Up regulated genes

c169528\_g2(2.7047)

- egu:105045873

- Up regulated genes

c164685\_g2(0.51641)

- egu:105044042

- Up regulated genes

c168179\_g1(0.47487)

Close
