# Supplementary material for: Physiological and transcriptomic responses of Lanzhou Lily (Lilium davidii, var. unicolor) to cold stress
Source: PLoS One. 2020 Jan 23;15(1):e0227921. doi: 10.1371/journal.pone.0227921 (PMC6977731; doi:10.1371/journal.pone.0227921)
Supplement: S1 Zip — (Zip). CK: control (20°C); LT: low temperature (4°C). (ZIP) [file pone.0227921.s011.zip › S1 Zip/src/egu04141.html]

egu04141


- egu:105050980

- Up regulated genes

c160425\_g1(0.79127)

- egu:105046530

- Up regulated genes

c94583\_g1(0.75302)

- egu:105038626

- Up regulated genes

c171446\_g2(0.83336) c153315\_g2(1.5473)

- egu:105060095

- Up regulated genes

c147952\_g2(1.3844)

- egu:105056954

- Up regulated genes

c141698\_g2(1.4654) c141698\_g1(1.7769)
- egu:105045690

- Up regulated genes

c163181\_g5(1.0715)

- egu:105056954

- Up regulated genes

c141698\_g2(1.4654) c141698\_g1(1.7769)
- egu:105045690

- Up regulated genes

c163181\_g5(1.0715)

- egu:105051770

- Up regulated genes

c171451\_g1(0.47898)

- egu:105052833

- Up regulated genes

c161184\_g2(0.63645)

- egu:105054741

- Up regulated genes

c172657\_g2(1.5914)

- egu:105057396

- Up regulated genes

c159142\_g2(0.60085)

- egu:105053689

- Up regulated genes

c159312\_g1(1.1391)

- egu:105042707

- Up regulated genes

c141977\_g1(3.4707)

- egu:105041025

- Up regulated genes

c143179\_g1(0.69021)

Close
