# Supplementary material for: Physiological and transcriptomic responses of Lanzhou Lily (Lilium davidii, var. unicolor) to cold stress
Source: PLoS One. 2020 Jan 23;15(1):e0227921. doi: 10.1371/journal.pone.0227921 (PMC6977731; doi:10.1371/journal.pone.0227921)
Supplement: S1 Zip — (Zip). CK: control (20°C); LT: low temperature (4°C). (ZIP) [file pone.0227921.s011.zip › S1 Zip/src/egu04144.html]

egu04144


- egu:105056954

- Up regulated genes

c141698\_g1(1.7769) c141698\_g2(1.4654)
- egu:105045690

- Up regulated genes

c163181\_g5(1.0715)

- egu:105052346

- Up regulated genes

c147063\_g1(0.76359)

- egu:105032610

- Up regulated genes

c165298\_g1(1.0114)
- egu:105049593

- Up regulated genes

c105465\_g1(0.80573)
- egu:105043229

- Up regulated genes

c162333\_g2(1.1056)

- egu:105045676

- Up regulated genes

c161639\_g1(2.6308)

- egu:105033970

- Up regulated genes

c165623\_g2(1.1898)
- egu:105045139

- Up regulated genes

c133817\_g1(0.83679)
- egu:105054164

- Up regulated genes

c167245\_g1(1.5987)

- egu:105033970

- Up regulated genes

c165623\_g2(1.1898)
- egu:105045139

- Up regulated genes

c133817\_g1(0.83679)
- egu:105054164

- Up regulated genes

c167245\_g1(1.5987)

- egu:105048729

- Up regulated genes

c162261\_g1(0.7909)

- egu:105048729

- Up regulated genes

c162261\_g1(0.7909)

- egu:105039433

- Up regulated genes

c173571\_g2(0.61422)

- egu:105033970

- Up regulated genes

c165623\_g2(1.1898)
- egu:105045139

- Up regulated genes

c133817\_g1(0.83679)
- egu:105054164

- Up regulated genes

c167245\_g1(1.5987)

- egu:105044044

- Up regulated genes

c142610\_g1(2.458) c142610\_g2(1.8907)

- egu:105032610

- Up regulated genes

c165298\_g1(1.0114)
- egu:105049593

- Up regulated genes

c105465\_g1(0.80573)
- egu:105043229

- Up regulated genes

c162333\_g2(1.1056)

- egu:105058367

- Up regulated genes

c173338\_g6(0.59266)
- egu:105038113

- Up regulated genes

c171004\_g1(0.51841)

Close
