# Supplementary material for: Physiological and transcriptomic responses of Lanzhou Lily (Lilium davidii, var. unicolor) to cold stress
Source: PLoS One. 2020 Jan 23;15(1):e0227921. doi: 10.1371/journal.pone.0227921 (PMC6977731; doi:10.1371/journal.pone.0227921)
Supplement: S1 Zip — (Zip). CK: control (20°C); LT: low temperature (4°C). (ZIP) [file pone.0227921.s011.zip › S1 Zip/src/egu04145.html]

egu04145


- egu:105059030

- Up regulated genes

c163575\_g2(1.5296)
- egu:105054846

- Up regulated genes

c149749\_g2(0.85695)
- egu:105042885

- Up regulated genes

c157053\_g1(2.7231)

- egu:105045810

- Up regulated genes

c169214\_g1(0.84119)

- egu:105045810

- Up regulated genes

c169214\_g1(0.84119)

- egu:105056668

- Up regulated genes

c152208\_g2(0.80623)

- egu:105050980

- Up regulated genes

c160425\_g1(0.79127)

- egu:105045810

- Up regulated genes

c169214\_g1(0.84119)

Close
