# Supplementary material for: Physiological and transcriptomic responses of Lanzhou Lily (Lilium davidii, var. unicolor) to cold stress
Source: PLoS One. 2020 Jan 23;15(1):e0227921. doi: 10.1371/journal.pone.0227921 (PMC6977731; doi:10.1371/journal.pone.0227921)
Supplement: S1 Zip — (Zip). CK: control (20°C); LT: low temperature (4°C). (ZIP) [file pone.0227921.s011.zip › S1 Zip/src/egu04626.html]

egu04626


- egu:105050781

- Up regulated genes

c147877\_g2(1.4098)

- egu:105044758

- Up regulated genes

c166605\_g3(1.2517)

- egu:105057333

- Up regulated genes

c101051\_g1(1.3264) c166855\_g1(2.4733)
- egu:105046031

- Up regulated genes

c156536\_g2(0.8528)
- egu:105052456

- Up regulated genes

c147918\_g2(0.79224)
- egu:105050612

- Up regulated genes

c174710\_g5(0.60072)
- egu:105034665

- Up regulated genes

c152058\_g1(1.67)
- egu:105057654

- Up regulated genes

c113196\_g1(0.58318)

- egu:105048941

- Up regulated genes

c154003\_g1(0.60818)

- egu:105048941

- Up regulated genes

c154003\_g1(0.60818)

- egu:105051034

- Up regulated genes

c153491\_g1(0.88576)

- egu:105047519

- Up regulated genes

c158996\_g1(0.91481)
- egu:105060351

- Up regulated genes

c166423\_g2(0.87115)

- egu:105047530

- Up regulated genes

c161556\_g2(0.58324)
- egu:105052035

- Up regulated genes

c163371\_g2(1.3489)
- egu:105046864

- Up regulated genes

c158259\_g1(0.86799)

- egu:105054741

- Up regulated genes

c172657\_g2(1.5914)

- egu:105057222

- Up regulated genes

c158416\_g1(1.2656)

Close
