# Supplementary material for: Physiological and transcriptomic responses of Lanzhou Lily (Lilium davidii, var. unicolor) to cold stress
Source: PLoS One. 2020 Jan 23;15(1):e0227921. doi: 10.1371/journal.pone.0227921 (PMC6977731; doi:10.1371/journal.pone.0227921)
Supplement: S1 Zip — (Zip). CK: control (20°C); LT: low temperature (4°C). (ZIP) [file pone.0227921.s011.zip › S1 Zip/src/egu04712.html]

egu04712


- egu:105035907

- Up regulated genes

c169999\_g5(2.0624)

- egu:105040294

- Up regulated genes

c161583\_g1(1.9184)

- egu:105041113

- Up regulated genes

c174283\_g3(7.128)

- egu:105040294

- Up regulated genes

c161583\_g1(1.9184)

- egu:105036385

- Up regulated genes

c171563\_g1(1.6467)

- egu:105040294

- Up regulated genes

c161583\_g1(1.9184)

- egu:105045005

- Up regulated genes

c164886\_g1(0.87098)

- egu:105036385

- Up regulated genes

c171563\_g1(1.6467)

- egu:105040294

- Up regulated genes

c161583\_g1(1.9184)

- egu:105050962

- Up regulated genes

c151401\_g1(4.5093) c146896\_g1(6.4969) c146896\_g3(7.3843) c174513\_g3(4.5905) c174513\_g1(5.3633)
- egu:105036364

- Up regulated genes

c155146\_g1(7.5424)
- egu:105035716

- Up regulated genes

c169857\_g1(2.9204)

- egu:105059424

- Up regulated genes

c152959\_g1(2.4409)

- egu:105047555

- Up regulated genes

c171275\_g1(2.4877)

- egu:105040182

- Up regulated genes

c186285\_g1(2.6295) c121976\_g1(3.6373)

- egu:105045005

- Up regulated genes

c164886\_g1(0.87098)

- egu:105040132

- Up regulated genes

c170144\_g1(0.85396)

- egu:105040132

- Up regulated genes

c170144\_g1(0.85396)

Close
