# Supplementary material for: Physiological and transcriptomic responses of Lanzhou Lily (Lilium davidii, var. unicolor) to cold stress
Source: PLoS One. 2020 Jan 23;15(1):e0227921. doi: 10.1371/journal.pone.0227921 (PMC6977731; doi:10.1371/journal.pone.0227921)
Supplement: S2 Zip — (Zip). CK: control (20°C); LT: low temperature (4°C). (ZIP) [file pone.0227921.s012.zip › S2 Zip/LTvsCK_DOWN/src/egu03440.html]

egu03440


- egu:105051907

- Down regulated genes

c169317\_g2(-1.0868)

- egu:105047264

- Down regulated genes

c133317\_g1(-3.1033)

- egu:105051907

- Down regulated genes

c169317\_g2(-1.0868)

Close
