# Supplementary material for: Physiological and transcriptomic responses of Lanzhou Lily (Lilium davidii, var. unicolor) to cold stress
Source: PLoS One. 2020 Jan 23;15(1):e0227921. doi: 10.1371/journal.pone.0227921 (PMC6977731; doi:10.1371/journal.pone.0227921)
Supplement: S2 Zip — (Zip). CK: control (20°C); LT: low temperature (4°C). (ZIP) [file pone.0227921.s012.zip › S2 Zip/LTvsCK_DOWN/src/egu04075.html]

egu04075


- egu:105040625

- Down regulated genes

c166932\_g3(-0.58771)

- egu:105042180

- Down regulated genes

c153105\_g1(-1.212)
- egu:105035543

- Down regulated genes

c158461\_g1(-1.4814)

- egu:105052996

- Down regulated genes

c164642\_g1(-2.2913)

- egu:105044738

- Down regulated genes

c137332\_g1(-1.5195)
- egu:105048010

- Down regulated genes

c71549\_g1(-1.4756)
- egu:105039360

- Down regulated genes

c156359\_g1(-0.72568)

- egu:105038391

- Down regulated genes

c78828\_g1(-4.9012)

- egu:105060959

- Down regulated genes

c151241\_g1(-4.9273)

- egu:105041587

- Down regulated genes

c163658\_g1(-0.65321)

- egu:105032733

- Down regulated genes

c173216\_g1(-1.0906) c150424\_g3(-1.9215) c150424\_g2(-2.0457)
- egu:105059948

- Down regulated genes

c174483\_g5(-0.60715)

- egu:105033626

- Down regulated genes

c164712\_g1(-0.6386)

- egu:105042180

- Down regulated genes

c153105\_g1(-1.212)
- egu:105035543

- Down regulated genes

c158461\_g1(-1.4814)

- egu:105034824

- Down regulated genes

c164908\_g1(-1.7606)
- egu:105042455

- Down regulated genes

c93769\_g1(-3.3405) c157547\_g1(-3.448)
- egu:105035706

- Down regulated genes

c133669\_g1(-1.4547)

Close
