# Supplementary material for: Physiological and transcriptomic responses of Lanzhou Lily (Lilium davidii, var. unicolor) to cold stress
Source: PLoS One. 2020 Jan 23;15(1):e0227921. doi: 10.1371/journal.pone.0227921 (PMC6977731; doi:10.1371/journal.pone.0227921)
Supplement: S2 Zip — (Zip). CK: control (20°C); LT: low temperature (4°C). (ZIP) [file pone.0227921.s012.zip › S2 Zip/LTvsCK_DOWN/src/egu04120.html]

egu04120


- egu:105056281

- Down regulated genes

c154764\_g1(-1.4115)

- egu:105056281

- Down regulated genes

c154764\_g1(-1.4115)

- egu:105056281

- Down regulated genes

c154764\_g1(-1.4115)

- egu:105056281

- Down regulated genes

c154764\_g1(-1.4115)

- egu:105054824

- Down regulated genes

c153783\_g1(-0.80958)

- egu:105055105

- Down regulated genes

c156645\_g1(-1.4217)

- egu:105055105

- Down regulated genes

c156645\_g1(-1.4217)

- egu:105056281

- Down regulated genes

c154764\_g1(-1.4115)

- egu:105041586

- Down regulated genes

c173888\_g1(-0.82868)
- egu:105057710

- Down regulated genes

c163535\_g1(-0.99217)

- egu:105054824

- Down regulated genes

c153783\_g1(-0.80958)

- egu:105040742

- Down regulated genes

c27396\_g2(-0.71877)

- egu:105055896

- Down regulated genes

c167530\_g4(-1.1042)

- egu:105038577

- Down regulated genes

c165970\_g3(-0.64957)

- egu:105048331

- Down regulated genes

c170244\_g1(-1.0817)

Close
