# Supplementary material for: Physiological and transcriptomic responses of Lanzhou Lily (Lilium davidii, var. unicolor) to cold stress
Source: PLoS One. 2020 Jan 23;15(1):e0227921. doi: 10.1371/journal.pone.0227921 (PMC6977731; doi:10.1371/journal.pone.0227921)
Supplement: S2 Zip — (Zip). CK: control (20°C); LT: low temperature (4°C). (ZIP) [file pone.0227921.s012.zip › S2 Zip/LTvsCK_DOWN/src/egu04130.html]

egu04130


- egu:105034960

- Down regulated genes

c146978\_g1(-0.8999)

- egu:105035227

- Down regulated genes

c171131\_g2(-0.51467)

- egu:105041420

- Down regulated genes

c237311\_g1(-0.60205)

- egu:105036838

- Down regulated genes

c158471\_g1(-0.57035)

Close
