# Supplementary material for: Physiological and transcriptomic responses of Lanzhou Lily (Lilium davidii, var. unicolor) to cold stress
Source: PLoS One. 2020 Jan 23;15(1):e0227921. doi: 10.1371/journal.pone.0227921 (PMC6977731; doi:10.1371/journal.pone.0227921)
Supplement: S2 Zip — (Zip). CK: control (20°C); LT: low temperature (4°C). (ZIP) [file pone.0227921.s012.zip › S2 Zip/LTvsCK_DOWN/src/egu04141.html]

egu04141


- egu:105049829

- Down regulated genes

c167530\_g1(-0.90984)

- egu:105042748

- Down regulated genes

c159704\_g1(-0.70866)

- egu:105056954

- Down regulated genes

c171346\_g1(-0.97358)

- egu:105056281

- Down regulated genes

c154764\_g1(-1.4115)

- egu:105040742

- Down regulated genes

c27396\_g2(-0.71877)

- egu:105056954

- Down regulated genes

c171346\_g1(-0.97358)

- egu:105038577

- Down regulated genes

c165970\_g3(-0.64957)

- egu:105040742

- Down regulated genes

c27396\_g2(-0.71877)

- egu:105055105

- Down regulated genes

c156645\_g1(-1.4217)

- egu:105053788

- Down regulated genes

c151183\_g1(-0.61249)
- egu:105042852

- Down regulated genes

c152923\_g1(-0.92216)

- egu:105038577

- Down regulated genes

c165970\_g3(-0.64957)

- egu:105047872

- Down regulated genes

c114581\_g1(-4.1318)
- egu:105050569

- Down regulated genes

c168095\_g1(-2.747) c172850\_g2(-1.9951)
- egu:105042707

- Down regulated genes

c150102\_g1(-1.9773)

- egu:105056346

- Down regulated genes

c167026\_g8(-2.4761)

- egu:105038834

- Down regulated genes

c140690\_g1(-0.90709)

Close
