# Supplementary material for: Physiological and transcriptomic responses of Lanzhou Lily (Lilium davidii, var. unicolor) to cold stress
Source: PLoS One. 2020 Jan 23;15(1):e0227921. doi: 10.1371/journal.pone.0227921 (PMC6977731; doi:10.1371/journal.pone.0227921)
Supplement: S2 Zip — (Zip). CK: control (20°C); LT: low temperature (4°C). (ZIP) [file pone.0227921.s012.zip › S2 Zip/LTvsCK_DOWN/src/egu04144.html]

egu04144


- egu:105056954

- Down regulated genes

c171346\_g1(-0.97358)

- egu:105033417

- Down regulated genes

c171234\_g3(-0.71198)

- egu:105060163

- Down regulated genes

c224136\_g1(-0.74326)

- egu:105060163

- Down regulated genes

c224136\_g1(-0.74326)

- egu:105048729

- Down regulated genes

c169514\_g5(-1.5841)

- egu:105048729

- Down regulated genes

c169514\_g5(-1.5841)

- egu:105032379

- Down regulated genes

c168291\_g1(-0.5719)
- egu:105056969

- Down regulated genes

c157769\_g1(-0.53548)
- egu:105044452

- Down regulated genes

c157769\_g2(-0.68511)
- egu:105033827

- Down regulated genes

c216457\_g1(-2.3078) c165983\_g1(-0.77155)

- egu:105060163

- Down regulated genes

c224136\_g1(-0.74326)

- egu:105061464

- Down regulated genes

c164929\_g1(-0.6533)

- egu:105038113

- Down regulated genes

c168008\_g1(-2.732)

Close
