# Supplementary material for: Physiological and transcriptomic responses of Lanzhou Lily (Lilium davidii, var. unicolor) to cold stress
Source: PLoS One. 2020 Jan 23;15(1):e0227921. doi: 10.1371/journal.pone.0227921 (PMC6977731; doi:10.1371/journal.pone.0227921)
Supplement: S2 Zip — (Zip). CK: control (20°C); LT: low temperature (4°C). (ZIP) [file pone.0227921.s012.zip › S2 Zip/LTvsCK_DOWN/src/egu04145.html]

egu04145


- egu:105032201

- Down regulated genes

c80110\_g1(-1.6082)

- egu:105032201

- Down regulated genes

c80110\_g1(-1.6082)

- egu:105059836

- Down regulated genes

c141394\_g1(-1.4363)
- egu:105045457

- Down regulated genes

c141394\_g3(-1.5135)

- egu:105051928

- Down regulated genes

c158106\_g1(-0.5161)

- egu:105051928

- Down regulated genes

c158106\_g1(-0.5161)

- egu:105049829

- Down regulated genes

c167530\_g1(-0.90984)

- egu:105038582

- Down regulated genes

c27236\_g1(-1.1457)
- egu:105043351

- Down regulated genes

c141160\_g2(-1.3871)

- egu:105051928

- Down regulated genes

c158106\_g1(-0.5161)

Close
