# Supplementary material for: Physiological and transcriptomic responses of Lanzhou Lily (Lilium davidii, var. unicolor) to cold stress
Source: PLoS One. 2020 Jan 23;15(1):e0227921. doi: 10.1371/journal.pone.0227921 (PMC6977731; doi:10.1371/journal.pone.0227921)
Supplement: S2 Zip — (Zip). CK: control (20°C); LT: low temperature (4°C). (ZIP) [file pone.0227921.s012.zip › S2 Zip/LTvsCK_DOWN/src/egu04146.html]

egu04146


- egu:105044978

- Down regulated genes

c172387\_g1(-1.0816)
- egu:105039221

- Down regulated genes

c169731\_g1(-0.99073)

- egu:105059069

- Down regulated genes

c151165\_g1(-0.8279)
- egu:105060838

- Down regulated genes

c152778\_g1(-0.93334)

- egu:105044749

- Down regulated genes

c160079\_g1(-0.86492)

- egu:105052909

- Down regulated genes

c140758\_g1(-0.5625)

Close
