# Supplementary material for: Physiological and transcriptomic responses of Lanzhou Lily (Lilium davidii, var. unicolor) to cold stress
Source: PLoS One. 2020 Jan 23;15(1):e0227921. doi: 10.1371/journal.pone.0227921 (PMC6977731; doi:10.1371/journal.pone.0227921)
Supplement: S2 Zip — (Zip). CK: control (20°C); LT: low temperature (4°C). (ZIP) [file pone.0227921.s012.zip › S2 Zip/LTvsCK_DOWN/src/egu04626.html]

egu04626


- egu:105046686

- Down regulated genes

c164943\_g2(-0.853)

- egu:105057238

- Down regulated genes

c27081\_g1(-0.75075)
- egu:105048683

- Down regulated genes

c132911\_g1(-1.0956)
- egu:105052456

- Down regulated genes

c147918\_g3(-0.97921)

- egu:105048941

- Down regulated genes

c134621\_g1(-0.83855)

- egu:105048941

- Down regulated genes

c134621\_g1(-0.83855)

- egu:105033796

- Down regulated genes

c159963\_g1(-1.0636)

- egu:105055268

- Down regulated genes

c174341\_g1(-0.78788)
- egu:105052035

- Down regulated genes

c163371\_g1(-1.0977) c168884\_g1(-0.58797)

Close
