# Supplementary material for: Physiological and transcriptomic responses of Lanzhou Lily (Lilium davidii, var. unicolor) to cold stress
Source: PLoS One. 2020 Jan 23;15(1):e0227921. doi: 10.1371/journal.pone.0227921 (PMC6977731; doi:10.1371/journal.pone.0227921)
Supplement: S2 Zip — (Zip). CK: control (20°C); LT: low temperature (4°C). (ZIP) [file pone.0227921.s012.zip › S2 Zip/LTvsCK_DOWN/src/egu04712.html]

egu04712


- egu:105044676

- Down regulated genes

c170910\_g1(-1.4213)

- egu:105051422

- Down regulated genes

c114233\_g1(-Inf)

- egu:105054824

- Down regulated genes

c153783\_g1(-0.80958)

- egu:105036385

- Down regulated genes

c154458\_g1(-1.097)

- egu:105036385

- Down regulated genes

c154458\_g1(-1.097)

- egu:105054641

- Down regulated genes

c150398\_g4(-0.9785)

- egu:105054824

- Down regulated genes

c153783\_g1(-0.80958)

Close
